# Supplementary material for: Effectiveness of evidence based mental health apps on user health outcome: A systematic literature review
Source: PLoS One. 2025 Mar 25;20(3):e0319983. doi: 10.1371/journal.pone.0319983 (PMC11936281; doi:10.1371/journal.pone.0319983)
Supplement: S2 File — (DOCX) [file pone.0319983.s002.docx]

Search: **((Depression OR anxiety OR suicid*) AND (Mobile OR app OR smartphone)) NOT (sleep OR alcohol OR drugs OR addiction OR tobacco)** Filters: **English, from 2013 - 2023** Sort by: **Publication Date**

((("depressed"[All Fields] OR "depression"[MeSH Terms] OR "depression"[All Fields] OR "depressions"[All Fields] OR "depression s"[All Fields] OR "depressive disorder"[MeSH Terms] OR ("depressive"[All Fields] AND "disorder"[All Fields]) OR "depressive disorder"[All Fields] OR "depressivity"[All Fields] OR "depressive"[All Fields] OR "depressively"[All Fields] OR "depressiveness"[All Fields] OR "depressives"[All Fields] OR ("anxiety"[MeSH Terms] OR "anxiety"[All Fields] OR "anxieties"[All Fields] OR "anxiety s"[All Fields]) OR ("suicid"[All Fields] OR "suicidal ideation"[MeSH Terms] OR ("suicidal"[All Fields] AND "ideation"[All Fields]) OR "suicidal ideation"[All Fields] OR "suicidality"[All Fields] OR "suicidal"[All Fields] OR "suicidally"[All Fields] OR "suicidals"[All Fields] OR "suicide"[MeSH Terms] OR "suicide"[All Fields] OR "suicides"[All Fields] OR "suicide s"[All Fields] OR "suicided"[All Fields] OR "suiciders"[All Fields])) AND ("mobile"[All Fields] OR "mobiles"[All Fields] OR ("australas plant pathol"[Journal] OR "app"[All Fields]) OR ("smartphone"[MeSH Terms] OR "smartphone"[All Fields] OR "smartphones"[All Fields] OR "smartphone s"[All Fields]))) NOT ("sleep"[MeSH Terms] OR "sleep"[All Fields] OR "sleeping"[All Fields] OR "sleeps"[All Fields] OR "sleep s"[All Fields] OR ("alcohol s"[All Fields] OR "alcoholate"[All Fields] OR "alcoholates"[All Fields] OR "alcohols"[MeSH Terms] OR "alcohols"[All Fields] OR "ethanol"[MeSH Terms] OR "ethanol"[All Fields] OR "alcohol"[All Fields]) OR ("drug s"[All Fields] OR "pharmaceutical preparations"[MeSH Terms] OR ("pharmaceutical"[All Fields] AND "preparations"[All Fields]) OR "pharmaceutical preparations"[All Fields] OR "drugs"[All Fields]) OR ("addict"[All Fields] OR "addict s"[All Fields] OR "addicted"[All Fields] OR "addicting"[All Fields] OR "addiction s"[All Fields] OR "addictive"[All Fields] OR "addictiveness"[All Fields] OR "addictives"[All Fields] OR "addicts"[All Fields] OR "behavior, addictive"[MeSH Terms] OR ("behavior"[All Fields] AND "addictive"[All Fields]) OR "addictive behavior"[All Fields] OR "addiction"[All Fields] OR "addictions"[All Fields]) OR ("tobacco products"[MeSH Terms] OR ("tobacco"[All Fields] AND "products"[All Fields]) OR "tobacco products"[All Fields] OR "tobacco"[All Fields] OR "nicotiana"[MeSH Terms] OR "nicotiana"[All Fields] OR "tobacco s"[All Fields] OR "tobaccos"[All Fields]))) AND ((english[Filter]) AND (2013:2023[pdat]))
