# Supplementary material for: Effectiveness of evidence based mental health apps on user health outcome: A systematic literature review
Source: PLoS One. 2025 Mar 25;20(3):e0319983. doi: 10.1371/journal.pone.0319983 (PMC11936281; doi:10.1371/journal.pone.0319983)
Supplement: S3 File — (DOCX) [file pone.0319983.s003.docx]

Mixed Methods Appraisal Tool (MMAT), version 2018

| **Study** | **Category of study designs** | **Methodological quality criteria** | **Responses** | | | |
| --- | --- | --- | --- | --- | --- | --- |
|  |  |  | Yes | No | Can’t tell | Comments |
| Kageyama et al., 2021 | Screening questions (for all types) | S1. Are there clear research questions? |  |  |  |  |
|  |  | S2. Do the collected data allow to address the research questions? |  |  |  |  |
|  | **2. Quantitative randomized controlled trials** | 2.1. Is randomization appropriately performed? |  |  |  |  |
|  |  | 2.2. Are the groups comparable at baseline? |  |  |  |  |
|  |  | 2.3. Are there complete outcome data? |  |  |  |  |
|  |  | 2.4. Are outcome assessors blinded to the intervention provided? |  |  |  |  |
|  |  | 2.5 Did the participants adhere to the assigned intervention? |  |  |  |  |

| **Study** | **Category of study designs** | **Methodological quality criteria** | **Responses** | | | |
| --- | --- | --- | --- | --- | --- | --- |
|  |  |  | Yes | No | Can’t tell | Comments |
| McCloud et al., 2020 | Screening questions (for all types) | S1. Are there clear research questions? |  |  |  |  |
|  |  | S2. Do the collected data allow to address the research questions? |  |  |  |  |
|  | **2. Quantitative randomized controlled trials** | 2.1. Is randomization appropriately performed? |  |  |  |  |
|  |  | 2.2. Are the groups comparable at baseline? |  |  |  |  |
|  |  | 2.3. Are there complete outcome data? |  |  |  |  |
|  |  | 2.4. Are outcome assessors blinded to the intervention provided? |  |  |  |  |
|  |  | 2.5 Did the participants adhere to the assigned intervention? |  |  |  |  |

| **Study** | **Category of study designs** | **Methodological quality criteria** | **Responses** | | | |
| --- | --- | --- | --- | --- | --- | --- |
|  |  |  | Yes | No | Can’t tell | Comments |
| Franklin et al., 2016 | Screening questions (for all types) | S1. Are there clear research questions? |  |  |  |  |
|  |  | S2. Do the collected data allow to address the research questions? |  |  |  |  |
|  | **2. Quantitative randomized controlled trials** | 2.1. Is randomization appropriately performed? |  |  |  |  |
|  |  | 2.2. Are the groups comparable at baseline? |  |  |  |  |
|  |  | 2.3. Are there complete outcome data? |  |  |  |  |
|  |  | 2.4. Are outcome assessors blinded to the intervention provided? |  |  |  |  |
|  |  | 2.5 Did the participants adhere to the assigned intervention? |  |  |  |  |

| **Study** | **Category of study designs** | **Methodological quality criteria** | **Responses** | | | |
| --- | --- | --- | --- | --- | --- | --- |
|  |  |  | Yes | No | Can’t tell | Comments |
| Liao et al., 2022 | Screening questions (for all types) | S1. Are there clear research questions? |  |  |  |  |
|  |  | S2. Do the collected data allow to address the research questions? |  |  |  |  |
|  | **2. Quantitative randomized controlled trials** | 2.1. Is randomization appropriately performed? |  |  |  |  |
|  |  | 2.2. Are the groups comparable at baseline? |  |  |  |  |
|  |  | 2.3. Are there complete outcome data? |  |  |  |  |
|  |  | 2.4. Are outcome assessors blinded to the intervention provided? |  |  |  |  |
|  |  | 2.5 Did the participants adhere to the assigned intervention? |  |  |  |  |

| **Study** | **Category of study designs** | **Methodological quality criteria** | **Responses** | | | |
| --- | --- | --- | --- | --- | --- | --- |
|  |  |  | Yes | No | Can’t tell | Comments |
| Sano et al., 2022 | Screening questions (for all types) | S1. Are there clear research questions? |  |  |  |  |
|  |  | S2. Do the collected data allow to address the research questions? |  |  |  |  |
|  | **2. Quantitative randomized controlled trials** | 2.1. Is randomization appropriately performed? |  |  |  |  |
|  |  | 2.2. Are the groups comparable at baseline? |  |  |  |  |
|  |  | 2.3. Are there complete outcome data? |  |  |  |  |
|  |  | 2.4. Are outcome assessors blinded to the intervention provided? |  |  |  | The study does not specify if the outcome assessors were blinded. |
|  |  | 2.5 Did the participants adhere to the assigned intervention? |  |  |  |  |

| **Study** | **Category of study designs** | **Methodological quality criteria** | **Responses** | | | |
| --- | --- | --- | --- | --- | --- | --- |
|  |  |  | Yes | No | Can’t tell | Comments |
| Dennis-Tiwary et al., 2016 | Screening questions (for all types) | S1. Are there clear research questions? |  |  |  |  |
|  |  | S2. Do the collected data allow to address the research questions? |  |  |  |  |
|  | **2. Quantitative randomized controlled trials** | 2.1. Is randomization appropriately performed? |  |  |  |  |
|  |  | 2.2. Are the groups comparable at baseline? |  |  |  |  |
|  |  | 2.3. Are there complete outcome data? |  |  |  | Doesn’t mention |
|  |  | 2.4. Are outcome assessors blinded to the intervention provided? |  |  |  |  |
|  |  | 2.5 Did the participants adhere to the assigned intervention? |  |  |  |  |

| **Study** | **Category of study designs** | **Methodological quality criteria** | **Responses** | | | |
| --- | --- | --- | --- | --- | --- | --- |
|  |  |  | Yes | No | Can’t tell | Comments |
| Tighe et al., 2017 | Screening questions (for all types) | S1. Are there clear research questions? |  |  |  |  |
|  |  | S2. Do the collected data allow to address the research questions? |  |  |  |  |
|  | **2. Quantitative randomized controlled trials** | 2.1. Is randomization appropriately performed? |  |  |  |  |
|  |  | 2.2. Are the groups comparable at baseline? |  |  |  |  |
|  |  | 2.3. Are there complete outcome data? |  |  |  |  |
|  |  | 2.4. Are outcome assessors blinded to the intervention provided? |  |  |  |  |
|  |  | 2.5 Did the participants adhere to the assigned intervention? |  |  |  |  |

| **Study** | **Category of study designs** | **Methodological quality criteria** | **Responses** | | | |
| --- | --- | --- | --- | --- | --- | --- |
|  |  |  | Yes | No | Can’t tell | Comments |
| Arean et al., 2016 | Screening questions (for all types) | S1. Are there clear research questions? |  |  |  |  |
|  |  | S2. Do the collected data allow to address the research questions? |  |  |  |  |
|  | **2. Quantitative randomized controlled trials** | 2.1. Is randomization appropriately performed? |  |  |  |  |
|  |  | 2.2. Are the groups comparable at baseline? |  |  |  |  |
|  |  | 2.3. Are there complete outcome data? |  |  |  |  |
|  |  | 2.4. Are outcome assessors blinded to the intervention provided? |  |  |  |  |
|  |  | 2.5 Did the participants adhere to the assigned intervention? |  |  |  |  |

| **Study** | **Category of study designs** | **Methodological quality criteria** | **Responses** | | | |
| --- | --- | --- | --- | --- | --- | --- |
|  |  |  | Yes | No | Can’t tell | Comments |
| Flett et al., 2019 | Screening questions (for all types) | S1. Are there clear research questions? |  |  |  |  |
|  |  | S2. Do the collected data allow to address the research questions? |  |  |  |  |
|  | **2. Quantitative randomized controlled trials** | 2.1. Is randomization appropriately performed? |  |  |  |  |
|  |  | 2.2. Are the groups comparable at baseline? |  |  |  |  |
|  |  | 2.3. Are there complete outcome data? |  |  |  |  |
|  |  | 2.4. Are outcome assessors blinded to the intervention provided? |  |  |  |  |
|  |  | 2.5 Did the participants adhere to the assigned intervention? |  |  |  |  |

| **Study** | **Category of study designs** | **Methodological quality criteria** | **Responses** | | | |
| --- | --- | --- | --- | --- | --- | --- |
|  |  |  | Yes | No | Can’t tell | Comments |
| O'Donnell et al., 2023 | Screening questions (for all types) | S1. Are there clear research questions? |  |  |  |  |
|  |  | S2. Do the collected data allow to address the research questions? |  |  |  |  |
|  | **2. Quantitative randomized controlled trials** | 2.1. Is randomization appropriately performed? |  |  |  |  |
|  |  | 2.2. Are the groups comparable at baseline? |  |  |  |  |
|  |  | 2.3. Are there complete outcome data? |  |  |  |  |
|  |  | 2.4. Are outcome assessors blinded to the intervention provided? |  |  |  |  |
|  |  | 2.5 Did the participants adhere to the assigned intervention? |  |  |  |  |

| **Study** | **Category of study designs** | **Methodological quality criteria** | **Responses** | | | |
| --- | --- | --- | --- | --- | --- | --- |
|  |  |  | Yes | No | Can’t tell | Comments |
| Fish et al., 2019 | Screening questions (for all types) | S1. Are there clear research questions? |  |  |  |  |
|  |  | S2. Do the collected data allow to address the research questions? |  |  |  |  |
|  | **2. Quantitative randomized controlled trials** | 2.1. Is randomization appropriately performed? |  |  |  |  |
|  |  | 2.2. Are the groups comparable at baseline? |  |  |  |  |
|  |  | 2.3. Are there complete outcome data? |  |  |  |  |
|  |  | 2.4. Are outcome assessors blinded to the intervention provided? |  |  |  |  |
|  |  | 2.5 Did the participants adhere to the assigned intervention? |  |  |  |  |

| **Study** | **Category of study designs** | **Methodological quality criteria** | **Responses** | | | |
| --- | --- | --- | --- | --- | --- | --- |
|  |  |  | Yes | No | Can’t tell | Comments |
| (Bell et al., 2023) | Screening questions (for all types) | S1. Are there clear research questions? |  |  |  |  |
|  |  | S2. Do the data collected allow you to address the research questions? |  |  |  |  |
|  | **2. Quantitative randomized controlled trials** | 2.1. Is randomization appropriately performed? |  |  |  |  |
|  |  | 2.2. Are the groups comparable at baseline? |  |  |  |  |
|  |  | 2.3. Is there complete outcome data? |  |  |  |  |
|  |  | 2.4. Are outcome assessors blinded to the intervention provided? |  |  |  |  |
|  |  | 2.5 Did the participants adhere to the assigned intervention? |  |  |  |  |

| **Study** | **Category of study designs** | **Methodological quality criteria** | **Responses** | | | |
| --- | --- | --- | --- | --- | --- | --- |
|  |  |  | Yes | No | Can’t tell | Comments |
| (Kusumadewi et al., 2023) | Screening questions (for all types) | S1. Are there clear research questions? |  |  |  |  |
|  |  | S2. Do the collected data allow to address the research questions? |  |  |  |  |
|  | **2. Quantitative randomized controlled trials** | 2.1. Is randomization appropriately performed? |  |  |  |  |
|  |  | 2.2. Are the groups comparable at baseline? |  |  |  |  |
|  |  | 2.3. Are there complete outcome data? |  |  |  |  |
|  |  | 2.4. Are outcome assessors blinded to the intervention provided? |  |  |  |  |
|  |  | 2.5 Did the participants adhere to the assigned intervention? |  |  |  | The document does not provide specific details on the adherence rates of participants to the assigned interventions. |

| **Study** | **Category of study designs** | **Methodological quality criteria** | **Responses** | | | |
| --- | --- | --- | --- | --- | --- | --- |
|  |  |  | Yes | No | Can’t tell | Comments |
| (Kirykowicz et al., 2023) | Screening questions (for all types) | S1. Are there clear research questions? |  |  |  |  |
|  |  | S2. Do the collected data allow to address the research questions? |  |  |  |  |
|  | **2. Quantitative randomized controlled trials** | 2.1. Is randomization appropriately performed? |  |  |  |  |
|  |  | 2.2. Are the groups comparable at baseline? |  |  |  |  |
|  |  | 2.3. Are there complete outcome data? |  |  |  | The transcript does not provide explicit information on whether the outcome data is complete for all participants. |
|  |  | 2.4. Are outcome assessors blinded to the intervention provided? |  |  |  | It is unclear if blinding was implemented in this study. |
|  |  | 2.5 Did the participants adhere to the assigned intervention? |  |  |  |  |

| **Study** | **Category of study designs** | **Methodological quality criteria** | **Responses** | | | |
| --- | --- | --- | --- | --- | --- | --- |
|  |  |  | Yes | No | Can’t tell | Comments |
| (Liu et al., 2023) | Screening questions (for all types) | S1. Are there clear research questions? |  |  |  |  |
|  |  | S2. Do the collected data allow to address the research questions? |  |  |  |  |
|  | **2. Quantitative randomized controlled trials** | 2.1. Is randomization appropriately performed? |  |  |  |  |
|  |  | 2.2. Are the groups comparable at baseline? |  |  |  |  |
|  |  | 2.3. Are there complete outcome data? |  |  |  |  |
|  |  | 2.4. Are outcome assessors blinded to the intervention provided? |  |  |  | The document does not provide explicit information on whether the outcome assessors were blinded to the intervention provided. |
|  |  | 2.5 Did the participants adhere to the assigned intervention? |  |  |  |  |

| **Study** | **Category of study designs** | **Methodological quality criteria** | **Responses** | | | |
| --- | --- | --- | --- | --- | --- | --- |
|  |  |  | Yes | No | Can’t tell | Comments |
| (Tan et al., 2023) | Screening questions (for all types) | S1. Are there clear research questions? |  |  |  |  |
|  |  | S2. Do the collected data allow to address the research questions? |  |  |  |  |
|  | **2. Quantitative randomized controlled trials** | 2.1. Is randomization appropriately performed? |  |  |  |  |
|  |  | 2.2. Are the groups comparable at baseline? |  |  |  |  |
|  |  | 2.3. Are there complete outcome data? |  |  |  |  |
|  |  | 2.4. Are outcome assessors blinded to the intervention provided? |  |  |  |  |
|  |  | 2.5 Did the participants adhere to the assigned intervention? |  |  |  | The document does not provide specific details on the adherence of participants to the assigned intervention. |

| **Study** | **Category of study designs** | **Methodological quality criteria** | **Responses** | | | |
| --- | --- | --- | --- | --- | --- | --- |
|  |  |  | Yes | No | Can’t tell | Comments |
| (Deady et al., 2023) | Screening questions (for all types) | S1. Are there clear research questions? |  |  |  |  |
|  |  | S2. Do the collected data allow to address the research questions? |  |  |  |  |
|  | **2. Quantitative randomized controlled trials** | 2.1. Is randomization appropriately performed? |  |  |  |  |
|  |  | 2.2. Are the groups comparable at baseline? |  |  |  |  |
|  |  | 2.3. Are there complete outcome data? |  |  |  |  |
|  |  | 2.4. Are outcome assessors blinded to the intervention provided? |  |  |  |  |
|  |  | 2.5 Did the participants adhere to the assigned intervention? |  |  |  |  |

| **Study** | **Category of study designs** | **Methodological quality criteria** | **Responses** | | | |
| --- | --- | --- | --- | --- | --- | --- |
|  |  |  | Yes | No | Can’t tell | Comments |
| (Schwob and Newman, 2023) | Screening questions (for all types) | S1. Are there clear research questions? |  |  |  |  |
|  |  | S2. Do the collected data allow to address the research questions? |  |  |  |  |
|  | **2. Quantitative randomized controlled trials** | 2.1. Is randomization appropriately performed? |  |  |  |  |
|  |  | 2.2. Are the groups comparable at baseline? |  |  |  |  |
|  |  | 2.3. Are there complete outcome data? |  |  |  |  |
|  |  | 2.4. Are outcome assessors blinded to the intervention provided? |  |  |  |  |
|  |  | 2.5 Did the participants adhere to the assigned intervention? |  |  |  |  |

| **Study** | **Category of study designs** | **Methodological quality criteria** | **Responses** | | | |
| --- | --- | --- | --- | --- | --- | --- |
|  |  |  | Yes | No | Can’t tell | Comments |
| (Ejiri et al., 2023) | Screening questions (for all types) | S1. Are there clear research questions? |  |  |  |  |
|  |  | S2. Do the collected data allow to address the research questions? |  |  |  |  |
|  | **2. Quantitative randomized controlled trials** | 2.1. Is randomization appropriately performed? |  |  |  |  |
|  |  | 2.2. Are the groups comparable at baseline? |  |  |  |  |
|  |  | 2.3. Are there complete outcome data? |  |  |  |  |
|  |  | 2.4. Are outcome assessors blinded to the intervention provided? |  |  |  |  |
|  |  | 2.5 Did the participants adhere to the assigned intervention? |  |  |  |  |

| **Study** | **Category of study designs** | **Methodological quality criteria** | **Responses** | | | |
| --- | --- | --- | --- | --- | --- | --- |
|  |  |  | Yes | No | Can’t tell | Comments |
| (Boden et al., 2023) | Screening questions (for all types) | S1. Are there clear research questions? |  |  |  |  |
|  |  | S2. Do the collected data allow to address the research questions? |  |  |  |  |
|  | **2. Quantitative randomized controlled trials** | 2.1. Is randomization appropriately performed? |  |  |  |  |
|  |  | 2.2. Are the groups comparable at baseline? |  |  |  |  |
|  |  | 2.3. Are there complete outcome data? |  |  |  |  |
|  |  | 2.4. Are outcome assessors blinded to the intervention provided? |  |  |  |  |
|  |  | 2.5 Did the participants adhere to the assigned intervention? |  |  |  |  |

| **Study** | **Category of study designs** | **Methodological quality criteria** | **Responses** | | | |
| --- | --- | --- | --- | --- | --- | --- |
|  |  |  | Yes | No | Can’t tell | Comments |
| (Litvin et al., 2023) | Screening questions (for all types) | S1. Are there clear research questions? |  |  |  |  |
|  |  | S2. Do the collected data allow to address the research questions? |  |  |  |  |
|  | **2. Quantitative randomized controlled trials** | 2.1. Is randomization appropriately performed? |  |  |  |  |
|  |  | 2.2. Are the groups comparable at baseline? |  |  |  |  |
|  |  | 2.3. Are there complete outcome data? |  |  |  |  |
|  |  | 2.4. Are outcome assessors blinded to the intervention provided? |  |  |  | The document does not specify whether outcome assessors were blinded to the intervention provided. |
|  |  | 2.5 Did the participants adhere to the assigned intervention? |  |  |  |  |

| **Study** | **Category of study designs** | **Methodological quality criteria** | **Responses** | | | |
| --- | --- | --- | --- | --- | --- | --- |
|  |  |  | Yes | No | Can’t tell | Comments |
| (Domar et al., 2023) | Screening questions (for all types) | S1. Are there clear research questions? |  |  |  |  |
|  |  | S2. Do the collected data allow to address the research questions? |  |  |  |  |
|  | **2. Quantitative randomized controlled trials** | 2.1. Is randomization appropriately performed? |  |  |  |  |
|  |  | 2.2. Are the groups comparable at baseline? |  |  |  |  |
|  |  | 2.3. Are there complete outcome data? |  |  |  |  |
|  |  | 2.4. Are outcome assessors blinded to the intervention provided? |  |  |  | The document does not explicitly state whether the outcome assessors were blinded to the intervention provided. |
|  |  | 2.5 Did the participants adhere to the assigned intervention? |  |  |  | It is not clear whether the participants adhered to the assigned intervention. |

| **Study** | **Category of study designs** | **Methodological quality criteria** | **Responses** | | | |
| --- | --- | --- | --- | --- | --- | --- |
|  |  |  | Yes | No | Can’t tell | Comments |
| (Lahtinen et al., 2023) | Screening questions (for all types) | S1. Are there clear research questions? |  |  |  |  |
|  |  | S2. Do the collected data allow to address the research questions? |  |  |  |  |
|  | **2. Quantitative randomized controlled trials** | 2.1. Is randomization appropriately performed? |  |  |  |  |
|  |  | 2.2. Are the groups comparable at baseline? |  |  |  | The document does not provide explicit information on the baseline comparability of the groups. |
|  |  | 2.3. Are there complete outcome data? |  |  |  |  |
|  |  | 2.4. Are outcome assessors blinded to the intervention provided? |  |  |  | The document does not specify whether outcome assessors were blinded to the intervention provided. |
|  |  | 2.5 Did the participants adhere to the assigned intervention? |  |  |  |  |

| **Study** | **Category of study designs** | **Methodological quality criteria** | **Responses** | | | |
| --- | --- | --- | --- | --- | --- | --- |
|  |  |  | Yes | No | Can’t tell | Comments |
| (Qin et al., 2022) | Screening questions (for all types) | S1. Are there clear research questions? |  |  |  |  |
|  |  | S2. Do the collected data allow to address the research questions? |  |  |  |  |
|  | **2. Quantitative randomized controlled trials** | 2.1. Is randomization appropriately performed? |  |  |  |  |
|  |  | 2.2. Are the groups comparable at baseline? |  |  |  |  |
|  |  | 2.3. Are there complete outcome data? |  |  |  |  |
|  |  | 2.4. Are outcome assessors blinded to the intervention provided? |  |  |  |  |
|  |  | 2.5 Did the participants adhere to the assigned intervention? |  |  |  |  |

| **Study** | **Category of study designs** | **Methodological quality criteria** | **Responses** | | | |
| --- | --- | --- | --- | --- | --- | --- |
|  |  |  | Yes | No | Can’t tell | Comments |
| (Liu et al., 2022) | Screening questions (for all types) | S1. Are there clear research questions? |  |  |  |  |
|  |  | S2. Do the collected data allow to address the research questions? |  |  |  |  |
|  | **2. Quantitative randomized controlled trials** | 2.1. Is randomization appropriately performed? |  |  |  |  |
|  |  | 2.2. Are the groups comparable at baseline? |  |  |  |  |
|  |  | 2.3. Are there complete outcome data? |  |  |  |  |
|  |  | 2.4. Are outcome assessors blinded to the intervention provided? |  |  |  | The document does not explicitly state whether the outcome assessors were blinded to the intervention provided. |
|  |  | 2.5 Did the participants adhere to the assigned intervention? |  |  |  |  |

| **Study** | **Category of study designs** | **Methodological quality criteria** | **Responses** | | | |
| --- | --- | --- | --- | --- | --- | --- |
|  |  |  | Yes | No | Can’t tell | Comments |
| (Pham et al., 2016) | Screening questions (for all types) | S1. Are there clear research questions? |  |  |  |  |
|  |  | S2. Do the collected data allow to address the research questions? |  |  |  |  |
|  | **2. Quantitative randomized controlled trials** | 2.1. Is randomization appropriately performed? |  |  |  |  |
|  |  | 2.2. Are the groups comparable at baseline? |  |  |  |  |
|  |  | 2.3. Are there complete outcome data? |  |  |  |  |
|  |  | 2.4. Are outcome assessors blinded to the intervention provided? |  |  |  |  |
|  |  | 2.5 Did the participants adhere to the assigned intervention? |  |  |  |  |

| **Study** | **Category of study designs** | **Methodological quality criteria** | **Responses** | | | |
| --- | --- | --- | --- | --- | --- | --- |
|  |  |  | Yes | No | Can’t tell | Comments |
| (Birney et al., 2016) | Screening questions (for all types) | S1. Are there clear research questions? |  |  |  |  |
|  |  | S2. Do the collected data allow to address the research questions? |  |  |  |  |
|  | **2. Quantitative randomized controlled trials** | 2.1. Is randomization appropriately performed? |  |  |  |  |
|  |  | 2.2. Are the groups comparable at baseline? |  |  |  |  |
|  |  | 2.3. Are there complete outcome data? |  |  |  |  |
|  |  | 2.4. Are outcome assessors blinded to the intervention provided? |  |  |  |  |
|  |  | 2.5 Did the participants adhere to the assigned intervention? |  |  |  |  |

| **Study** | **Category of study designs** | **Methodological quality criteria** | **Responses** | | | |
| --- | --- | --- | --- | --- | --- | --- |
|  |  |  | Yes | No | Can’t tell | Comments |
| (Boucher et al., 2023) | Screening questions (for all types) | S1. Are there clear research questions? |  |  |  |  |
|  |  | S2. Do the collected data allow to address the research questions? |  |  |  |  |
|  | **2. Quantitative randomized controlled trials** | 2.1. Is randomization appropriately performed? |  |  |  |  |
|  |  | 2.2. Are the groups comparable at baseline? |  |  |  |  |
|  |  | 2.3. Are there complete outcome data? |  |  |  |  |
|  |  | 2.4. Are outcome assessors blinded to the intervention provided? |  |  |  | The document does not specify whether outcome assessors were blinded to the intervention provided. |
|  |  | 2.5 Did the participants adhere to the assigned intervention? |  |  |  |  |

| **Study** | **Category of study designs** | **Methodological quality criteria** | **Responses** | | | |
| --- | --- | --- | --- | --- | --- | --- |
|  |  |  | Yes | No | Can’t tell | Comments |
| (Hicks et al., 2022) | Screening questions (for all types) | S1. Are there clear research questions? |  |  |  |  |
|  |  | S2. Do the collected data allow to address the research questions? |  |  |  |  |
|  | **2. Quantitative randomized controlled trials** | 2.1. Is randomization appropriately performed? |  |  |  |  |
|  |  | 2.2. Are the groups comparable at baseline? |  |  |  |  |
|  |  | 2.3. Are there complete outcome data? |  |  |  |  |
|  |  | 2.4. Are outcome assessors blinded to the intervention provided? |  |  |  |  |
|  |  | 2.5 Did the participants adhere to the assigned intervention? |  |  |  |  |

| **Study** | **Category of study designs** | **Methodological quality criteria** | **Responses** | | | |
| --- | --- | --- | --- | --- | --- | --- |
|  |  |  | Yes | No | Can’t tell | Comments |
| Takahashi et al., 2019 | Screening questions (for all types) | S1. Are there clear research questions? |  |  |  |  |
|  |  | S2. Do the collected data allow to address the research questions? |  |  |  |  |
|  | **3. Quantitative non- randomized** | 3.1. Are the participants representative of the target population? |  |  |  | not fully detailed or mentioned. |
|  |  | 3.2. Are measurements appropriate regarding both the outcome and intervention (or exposure)? |  |  |  |  |
|  |  | 3.3. Are there complete outcome data? |  |  |  |  |
|  |  | 3.4. Are the confounders accounted for in the design and analysis? |  |  |  |  |
|  |  | 3.5. During the study period, is the intervention administered (or exposure occurred) as intended? |  |  |  |  |

| **Study** | **Category of study designs** | **Methodological quality criteria** | **Responses** | | | |
| --- | --- | --- | --- | --- | --- | --- |
|  |  |  | Yes | No | Can’t tell | Comments |
| Kajitani et al., 2020 | Screening questions (for all types) | S1. Are there clear research questions? |  |  |  |  |
|  |  | S2. Do the collected data allow to address the research questions? |  |  |  |  |
|  | **3. Quantitative non- randomized** | 3.1. Are the participants representative of the target population? |  |  |  | the study does not provide enough information |
|  |  | 3.2. Are measurements appropriate regarding both the outcome and intervention (or exposure)? |  |  |  |  |
|  |  | 3.3. Are there complete outcome data? |  |  |  |  |
|  |  | 3.4. Are the confounders accounted for in the design and analysis? |  |  |  |  |
|  |  | 3.5. During the study period, is the intervention administered (or exposure occurred) as intended? |  |  |  |  |

| **Study** | **Category of study designs** | **Methodological quality criteria** | **Responses** | | | |
| --- | --- | --- | --- | --- | --- | --- |
|  |  |  | Yes | No | Can’t tell | Comments |
| Bakker & Rickard, 2018 | Screening questions (for all types) | S1. Are there clear research questions? |  |  |  |  |
|  |  | S2. Do the collected data allow to address the research questions? |  |  |  |  |
|  | **3. Quantitative non- randomized** | 3.1. Are the participants representative of the target population? |  |  |  | It does not give enough detail |
|  |  | 3.2. Are measurements appropriate regarding both the outcome and intervention (or exposure)? |  |  |  |  |
|  |  | 3.3. Are there complete outcome data? |  |  |  |  |
|  |  | 3.4. Are the confounders accounted for in the design and analysis? |  |  |  |  |
|  |  | 3.5. During the study period, is the intervention administered (or exposure occurred) as intended? |  |  |  |  |

| **Study** | **Category of study designs** | **Methodological quality criteria** | **Responses** | | | |
| --- | --- | --- | --- | --- | --- | --- |
|  |  |  | Yes | No | Can’t tell | Comments |
| Bakker & Rickard, 2019 | Screening questions (for all types) | S1. Are there clear research questions? |  |  |  |  |
|  |  | S2. Do the collected data allow to address the research questions? |  |  |  |  |
|  | **3. Quantitative non- randomized** | 3.1. Are the participants representative of the target population? |  |  |  |  |
|  |  | 3.2. Are measurements appropriate regarding both the outcome and intervention (or exposure)? |  |  |  |  |
|  |  | 3.3. Are there complete outcome data? |  |  |  |  |
|  |  | 3.4. Are the confounders accounted for in the design and analysis? |  |  |  |  |
|  |  | 3.5. During the study period, is the intervention administered (or exposure occurred) as intended? |  |  |  |  |

| **Study** | **Category of study designs** | **Methodological quality criteria** | **Responses** | | | |
| --- | --- | --- | --- | --- | --- | --- |
|  |  |  | Yes | No | Can’t tell | Comments |
| Cliffe & Stallard, 2023 | Screening questions (for all types) | S1. Are there clear research questions? |  |  |  |  |
|  |  | S2. Do the collected data allow to address the research questions? |  |  |  |  |
|  | **3. Quantitative non- randomized** | 3.1. Are the participants representative of the target population? |  |  |  |  |
|  |  | 3.2. Are measurements appropriate regarding both the outcome and intervention (or exposure)? |  |  |  |  |
|  |  | 3.3. Are there complete outcome data? |  |  |  |  |
|  |  | 3.4. Are the confounders accounted for in the design and analysis? |  |  |  |  |
|  |  | 3.5. During the study period, is the intervention administered (or exposure occurred) as intended? |  |  |  |  |

| **Study** | **Category of study designs** | **Methodological quality criteria** | **Responses** | | | |
| --- | --- | --- | --- | --- | --- | --- |
|  |  |  | Yes | No | Can’t tell | Comments |
| (Chang et al., 2023) | Screening questions (for all types) | S1. Are there clear research questions? |  |  |  |  |
|  |  | S2. Do the collected data allow to address the research questions? |  |  |  |  |
|  | **3. Quantitative non- randomized** | 3.1. Are the participants representative of the target population? |  |  |  |  |
|  |  | 3.2. Are measurements appropriate regarding both the outcome and intervention (or exposure)? |  |  |  |  |
|  |  | 3.3. Are there complete outcome data? |  |  |  |  |
|  |  | 3.4. Are the confounders accounted for in the design and analysis? |  |  |  |  |
|  |  | 3.5. During the study period, is the intervention administered (or exposure occurred) as intended? |  |  |  |  |

| **Study** | **Category of study designs** | **Methodological quality criteria** | **Responses** | | | |
| --- | --- | --- | --- | --- | --- | --- |
|  |  |  | Yes | No | Can’t tell | Comments |
| (Duraimani, 2019) | Screening questions (for all types) | S1. Are there clear research questions? |  |  |  |  |
|  |  | S2. Do the collected data allow to address the research questions? |  |  |  |  |
|  | **3. Quantitative non- randomized** | 3.1. Are the participants representative of the target population? |  |  |  |  |
|  |  | 3.2. Are measurements appropriate regarding both the outcome and intervention (or exposure)? |  |  |  |  |
|  |  | 3.3. Are there complete outcome data? |  |  |  |  |
|  |  | 3.4. Are the confounders accounted for in the design and analysis? |  |  |  |  |
|  |  | 3.5. During the study period, is the intervention administered (or exposure occurred) as intended? |  |  |  |  |

| **Study** | **Category of study designs** | **Methodological quality criteria** | **Responses** | | | |
| --- | --- | --- | --- | --- | --- | --- |
|  |  |  | Yes | No | Can’t tell | Comments |
| Carey et al., 2016 | Screening questions (for all types) | S1. Are there clear research questions? |  |  |  |  |
|  |  | S2. Do the collected data allow to address the research questions? |  |  |  |  |
|  | **5. Mixed methods** | 5.1. Is there an adequate rationale for using a mixed methods design to address the research question? |  |  |  |  |
|  |  | 5.2. Are the different components of the study effectively integrated to answer the research question? |  |  |  |  |
|  |  | 5.3. Are the outputs of the integration of qualitative and quantitative components adequately interpreted? |  |  |  |  |
|  |  | 5.4. Are divergences and inconsistencies between quantitative and qualitative results adequately addressed? |  |  |  | the text does not discuss if there were divergences or inconsistencies, nor how they were handled if present. |
|  |  | 5.5. Do the different components of the study adhere to the quality criteria of each tradition of the methods involved? |  |  |  |  |

| **Study** | **Category of study designs** | **Methodological quality criteria** | **Responses** | | | |
| --- | --- | --- | --- | --- | --- | --- |
|  |  |  | Yes | No | Can’t tell | Comments |
| (Zhou et al., 2023) | Screening questions (for all types) | S1. Are there clear research questions? |  |  |  |  |
|  |  | S2. Do the collected data allow you to address the research questions? |  |  |  |  |
|  | **5. Mixed methods** | 5.1. Is there an adequate rationale for using mixed methods design to address the research question? |  |  |  |  |
|  |  | 5.2. Are the different components of the study effectively integrated to answer the research question? |  |  |  |  |
|  |  | 5.3. Are the outputs of the integration of qualitative and quantitative components adequately interpreted? |  |  |  |  |
|  |  | 5.4. Are divergences and inconsistencies between quantitative and qualitative results adequately addressed? |  |  |  |  |
|  |  | 5.5. Do the different components of the study adhere to the quality criteria of each tradition of the methods involved? |  |  |  | The transcript does not provide detailed information on the specific quality criteria for each method used in the study. |

**References:**

BELL, I., ARNOLD, C., GILBERTSON, T., D’ALFONSO, S., CASTAGNINI, E., CHEN, N., NICHOLAS, J., O’SULLIVAN, S., VALENTINE, L. & ALVAREZ-JIMENEZ, M. 2023. A Personalized, Transdiagnostic Smartphone Intervention (Mello) Targeting Repetitive Negative Thinking in Young People With Depression and Anxiety: Pilot Randomized Controlled Trial. *Journal of Medical Internet Research,* 25**,** e47860.

BIRNEY, A. J., GUNN, R., RUSSELL, J. K. & ARY, D. V. 2016. MoodHacker mobile web app with email for adults to self-manage mild-to-moderate depression: randomized controlled trial. *JMIR mHealth and uHealth,* 4**,** e4231.

BODEN, L. M., RODRIGUEZ, C., KELLY IV, J. D., KHALSA, A. S. & CASPER, D. S. 2023. Mindfulness Applications: Can They Serve as a Stress, Anxiety, and Burnout Reduction Tool in Orthopaedic Surgery Training? A Randomized Control Trial. *JBJS Open Access,* 8**,** e22.

BOUCHER, V. G., HAIGHT, B. L., HIVES, B. A., ZUMBO, B. D., MERALI-DEWJI, A., HUTTON, S., LIU, Y., NGUYEN, S., BEAUCHAMP, M. R. & BLACK, A. T. 2023. Effects of 12 weeks of at-home, application-based exercise on health care workers’ depressive symptoms, burnout, and absenteeism: a randomized clinical trial. *JAMA psychiatry,* 80**,** 1101-1109.

CHANG, S., ALON, N. & TOROUS, J. 2023. An exploratory analysis of the effect size of the mobile mental health Application, mindLAMP. *Digital Health,* 9**,** 20552076231187244.

DEADY, M., COLLINS, D. A., LAVENDER, I., MACKINNON, A., GLOZIER, N., BRYANT, R., CHRISTENSEN, H. & HARVEY, S. B. 2023. Selective Prevention of Depression in Workers Using a Smartphone App: Randomized Controlled Trial. *Journal of Medical Internet Research,* 25**,** e45963.

DOMAR, A. D., JASULAITIS, L., MATEVOSSIAN, K., JASULAITIS, S., GRILL, E. A. & MEIKE, L. U. 2023. The Impact of the FertiStrong Mobile Application on Anxiety and Depression in Men: A Randomised Control Pilot Study. *Journal of human reproductive sciences,* 16**,** 195-203.

DURAIMANI, S. L. 2019. A cross-sectional and longitudinal study of the effects of a mindfulness meditation mobile application platform on reducing stress and anxiety. *International journal of yoga,* 12**,** 226-232.

EJIRI, H., UCHIDA, H., TSUCHIYA, K., FUJIWARA, K., KIKUCHI, S. & HIRAO, K. 2023. Immediate Effects of Mobile Phone App for Depressed Mood in Young Adults with Subthreshold Depression: A Pilot Randomized Controlled Trial. *Neuropsychiatric Disease and Treatment***,** 1695-1707.

HICKS, M., BRADEN, L., WALSH, E., GREENE, B. & GRAYSON, J. 2022. Mobile meditation for improving quality of life, anxiety and depression among surgical residents and faculty. *The Journal of Laryngology & Otology,* 136**,** 1034-1038.

KIRYKOWICZ, K., JAWORSKI, B., OWEN, J., KIRSCHBAUM, C., SEEDAT, S. & VAN DEN HEUVEL, L. L. 2023. Feasibility, acceptability and preliminary efficacy of a mental health self-management app in clinicians working during the COVID-19 pandemic: A pilot randomised controlled trial. *Psychiatry Research,* 329**,** 115493.

KUSUMADEWI, A. F., MARCHIRA, C. R., WIDYANDANA, D. & WIRASTO, R. T. 2023. Randomized Clinical Trial on the Comparison of Effect of Asynchronous Mobile Application and Guided Brief Cognitive Behavioral Therapy in Managing Anxiety among Medical Students. *Trends in Psychiatry and Psychotherapy***,** 0-0.

LAHTINEN, O., AALTONEN, J., KAAKINEN, J., FRANKLIN, L. & HYÖNÄ, J. 2023. The effects of app-based mindfulness practice on the well-being of university students and staff. *Current Psychology,* 42**,** 4412-4421.

LITVIN, S., SAUNDERS, R., JEFFERIES, P., SEELY, H., PÖSSEL, P. & LÜTTKE, S. 2023. The impact of a gamified Mobile mental health app (eQuoo) on resilience and mental health in a student population: large-scale randomized controlled trial. *JMIR Mental Health,* 10**,** e47285.

LIU, C., CHEN, H., ZHANG, A., GONG, X., WU, K., LIU, C.-Y. & CHIOU, W.-K. 2023. The effects of short video app-guided loving-kindness meditation on college students’ mindfulness, self-compassion, positive psychological capital, and suicide ideation. *Psicologia: Reflexão e Crítica,* 36**,** 32.

LIU, C., CHEN, H., ZHOU, F., LONG, Q., WU, K., LO, L.-M., HUNG, T.-H., LIU, C.-Y. & CHIOU, W.-K. 2022. Positive intervention effect of mobile health application based on mindfulness and social support theory on postpartum depression symptoms of puerperae. *BMC women's health,* 22**,** 413.

PHAM, Q., KHATIB, Y., STANSFELD, S., FOX, S. & GREEN, T. 2016. Feasibility and efficacy of an mHealth game for managing anxiety:“Flowy” randomized controlled pilot trial and design evaluation. *Games for health journal,* 5**,** 50-67.

QIN, X., LIU, C., ZHU, W., CHEN, Y. & WANG, Y. 2022. Preventing postpartum depression in the early postpartum period using an app-based cognitive behavioral therapy program: a pilot randomized controlled study. *International journal of environmental research and public health,* 19**,** 16824.

SCHWOB, J. T. & NEWMAN, M. G. 2023. Brief imaginal exposure exercises for social anxiety disorder: A randomized controlled trial of a self-help momentary intervention app. *Journal of Anxiety Disorders,* 98**,** 102749.

TAN, S., ISMAIL, M. A. B., DAUD, T. I. M., HOD, R. & AHMAD, N. 2023. A randomized controlled trial on the effect of smartphone-based mental health application among outpatients with depressive and anxiety symptoms: A pilot study in Malaysia. *Indian Journal of Psychiatry,* 65**,** 934-940.

ZHOU, X., EDIRIPPULIGE, S., JONES, A., BAI, X., SMITH, A. C. & BAMBLING, M. 2023. The feasibility, acceptability and efficacy of an app-based intervention (the Coping Camp) in reducing stress among Chinese school adolescents: A cluster randomised controlled trial. *Plos one,* 18**,** e0294119.
