# Supplementary material for: Effectiveness of evidence based mental health apps on user health outcome: A systematic literature review
Source: PLoS One. 2025 Mar 25;20(3):e0319983. doi: 10.1371/journal.pone.0319983 (PMC11936281; doi:10.1371/journal.pone.0319983)
Supplement: S4 File — (DOCX) [file pone.0319983.s004.docx]

|  | **DOI** | **Review** | **Title excluded** | **Abstract excluded** | **Full paper excluded** | **Final full paper** |
| --- | --- | --- | --- | --- | --- | --- |
| Pilot testing "Teach Ted": A digital application for children undergoing blood tests and their parents | 10.1016/j.pecinn.2023.100251 |  | Not at all relevant |  |  |  |
| Evaluation of phubbing online social support and trait anxiety in nurses | 10.1111/inr.12916 |  |  | No Mobile app |  |  |
| Enhancing the effectiveness of CBT for patients with unipolar depression by integrating digital interventions into treatment: A pilot randomized controlled trial | 10.1080/10503307.2023.2277866 |  |  | App usability |  |  |
| Examining the Impact of Assistive Technology on Psychological Health, Family Education, and Curriculum Research in Japan: Insights from Artificial Intelligence | 10.1007/s10803-023-06105-4 |  | Not at all relevant |  |  |  |
| Bouncing Back: Resilience as a Protective Factor for the Impact of Child Problem Behavior on Caregiver Depressive Cognitions Among Youth with Autism Spectrum Disorder | 10.1007/s10803-023-06094-4 |  | Not at all relevant |  |  |  |
| Affective arousal temporally precedes dissociation in patients with borderline personality disorder: A preliminary experience sampling study | 10.1037/tra0001516 |  | Not at all relevant |  |  |  |
| An Exploratory Study of Incels' Dating App Experiences, Mental Health, and Relational Well-Being | 10.1080/00224499.2023.2249775 |  | Not at all relevant |  |  |  |
| Perceptions about smartphone-based interventions to promote physical activity in inactive adults with knee pain - A qualitative study | 10.1080/17483107.2023.2272854 |  | Not at all relevant |  |  |  |
| Using a Randomized Clinical Trial to Test the Efficacy of a Culturally Responsive Mobile Health Application in African Americans | 10.1016/j.beth.2023.12.002 |  |  | App usability |  |  |
| Detecting adolescent depression through passive monitoring of linguistic markers in smartphone communication | 10.1111/jcpp.13931 |  | Not at all relevant |  |  |  |
| Editorial: Can Mobile Crisis Response Help Mitigate Racialized Disparities in Acute Child Mental Health Care? | 10.1016/j.jaac.2023.11.007 |  | Not at all relevant |  |  |  |
| Pilot testing the SUCCESS peer mentoring program for students with concussion: the role of personas in mobile technology development | 10.1080/17483107.2023.2239293 |  | Not at all relevant |  |  |  |
| Academic self-efficacy and cognitive strategy use in college students with and without depression or anxiety | 10.1080/07448481.2022.2076561 |  |  | No Mobile app |  |  |
| Evaluating the effects of gratitude interventions on college student well-being | 10.1080/07448481.2022.2076096 |  |  | No Mobile app |  |  |
| The efficacy, acceptability and safety of acceptance and commitment therapy for fibromyalgia - a systematic review and meta-analysis | 10.1177/20494637231221451 | Review paper |  |  |  |  |
| Fear of detachment from mobile phone: nomophobia and suicidality among Malaysian university students before and during the COVID-19 pandemic | 10.1080/13548506.2023.2274315 |  | Not at all relevant |  |  |  |
| App-based meditation habits maintain reductions in depression symptoms among autistic adults | 10.1177/13623613231200679 |  |  | Screening, no treatment |  |  |
| Photophobia Contributes to Migraine-Associated Disability and Reduced Work Productivity: Results From the American Registry for Migraine Research (ARMR) | 10.1097/WNO.0000000000001967 |  | Not at all relevant |  |  |  |
| Smartphone assessment uncovers real-time relationships between depressed mood and daily functional behaviors after stroke | 10.1177/1357633X221100061 |  | Not at all relevant |  |  |  |
| Evidence for Bidirectional, Cross-Lagged Associations Between Alliance and Psychological Distress in an Unguided Mobile-Health Intervention | 10.1177/21677026231184890 |  | Not at all relevant |  |  |  |
| Characterising use of recovery record among a large, transdiagnostic sample of adults with eating disorders across higher levels of care | 10.1002/erv.3053 |  | Not at all relevant |  |  |  |
| Imager-A mobile health mental imagery-based ecological momentary intervention targeting reward sensitivity: A randomized controlled trial | 10.1111/aphw.12505 |  | Not at all relevant |  |  |  |
| The impact of coping behaviors on perceived competence and social anxiety in the everyday social engagement of autistic adolescents | 10.1177/13623613231196773 |  |  | No Mobile app |  |  |
| Quality of Life and Real-time Patient Experience During Neoadjuvant Therapy: A Prospective Cohort Study | 10.1097/SLA.0000000000006090 |  | Not at all relevant |  |  |  |
| Testing the interpersonal theory of suicide in adolescents: A multi-wave longitudinal study | 10.1111/jcpp.13868 |  |  | No Mobile app |  |  |
| "My Brigham Baby" Application: A Pilot Study Using Technology to Enhance Parent's Experience in the Neonatal Intensive Care Unit | 10.1055/a-1990-2414 |  | Not at all relevant |  |  |  |
| Exploring the prevalence of nomophobia in a Canadian university: An environmental scan | 10.1080/07448481.2022.2070712 |  | Not at all relevant |  |  |  |
| A Randomized Control Trial of Meditation for Mothers Pumping Breastmilk for Preterm Infants | 10.1055/a-1787-7576 |  | Not at all relevant |  |  |  |
| Developing and Evaluating the DiabetesXcel Mobile Application for Adult Patients With Type 2 Diabetes | 10.2337/cd23-0034 |  | Not at all relevant |  |  |  |
| Breamy: An augmented reality mHealth prototype for surgical decision-making in breast cancer | 10.1049/htl2.12071 |  | Not at all relevant |  |  |  |
| Improving the Management of Maternal Mental Health with Digital Health Care | 10.1176/appi.prcp.20230035 |  |  | Screening, no treatment |  |  |
| Mental Health Apps for Children and Adolescents: A Clinician-Friendly Review | 10.1016/j.jaac.2023.07.1004 | Review paper |  |  |  |  |
| Live two-way video versus face-to-face treatment for depression, anxiety, and obsessive-compulsive disorder: A 24-week randomized controlled trial | 10.1111/pcn.13618 |  |  | Telehealth app |  |  |
| Mobile Crisis Services: A Clinician Survey of Current Suicide Prevention Practices and Barriers to Care Delivery | 10.1007/s10597-023-01208-9 |  | Not at all relevant |  |  |  |
| Self-Compassion, Health, and Empowerment: A Pilot Randomized Controlled Trial for Chinese Immigrant Women Experiencing Intimate Partner Violence | 10.1177/08862605231207624 |  | Not at all relevant |  |  |  |
| The emotional support plan: Feasibility trials of a brief, telehealth-based mobile intervention to support coping for autistic adults | 10.1177/13623613231186035 |  |  | No depression,anxiety,stress |  |  |
| Behavioral Activation, Depression, and Promotion of Health Behaviors: A Scoping Review | 10.1177/10901981221090157 | Review paper |  |  |  |  |
| Alleviation of behavioral deficits, amyloid-β deposition, and mitochondrial structure damage associated with mitophagy upregulation in AD animal models via AAV9-IGF-1 treatment | 10.1016/j.brainres.2023.148743 |  | Not at all relevant |  |  |  |
| Symptom Reduction and Engagement in a Cognitive-Behavioral Mobile Phone App: A Study of User Profiling to Determine Prognostic Indicators | 10.1016/j.beth.2023.05.014 |  |  | App usability |  |  |
| Smartphone-based augmented reality patient education in radiation oncology | 10.1016/j.tipsro.2023.100229 |  | Not at all relevant |  |  |  |
| Endometriosis and mental health disorders: identification and treatment as part of a multimodal approach | 10.1016/j.fertnstert.2023.12.033 |  |  | No Mobile app |  |  |
| Determinants and outcome correlates of engagement with a mobile mental health intervention for depression and anxiety in middle-aged and older adults | 10.1002/jclp.23636 |  |  | App usability |  |  |
| Nurse-guided Mobile Health Care Program to Reduce Emotional Distress Experienced by Parents of Infants Prenatally Diagnosed with Critical Congenital Heart Disease: A Pilot Study | 10.1016/j.ppedcard.2023.101687 |  |  | No depression,anxiety,stress |  |  |
| If it Ain't Broke, Don't Fix it: Positive Versus Negative Emotion Regulation in Daily Life and Depressive Symptoms | 10.1016/j.jad.2023.12.037 |  | Not at all relevant |  |  |  |
| Leveraging technology to increase the disseminability of evidence-based treatment of dental fear: An uncontrolled pilot study | 10.1111/jphd.12598 |  | Not at all relevant |  |  |  |
| Is internet-based psychological therapy effective for treating major depressive disorder? | 10.1080/14737175.2023.2295417 |  |  | No Mobile app |  |  |
| Neuroprotective effect of the traditional decoction Tian-Si-Yin against Alzheimer's disease via suppression of neuroinflammation | 10.1016/j.jep.2023.117569 |  | Not at all relevant |  |  |  |
| [VR-supported therapy for anxiety and posttraumatic stress disorder: current possibilities and limitations] | 10.1007/s00115-023-01570-9 |  | Not at all relevant |  |  |  |
| Brief observable anxiety sensitivity treatment: intervention development and a pilot randomized-controlled acceptability and feasibility trial to evaluate a brief intervention for anxiety sensitivity social concerns | 10.1080/16506073.2023.2288551 |  |  | Telehealth app |  |  |
| An investigation of the potential clinical utility of critical slowing down as an early warning sign for recurrence of depression | 10.1016/j.jbtep.2023.101922 |  | Not at all relevant |  |  |  |
| Design and implementation of a prototype radiotherapy menu in a patient portal | 10.1002/acm2.14201 |  | Not at all relevant |  |  |  |
| Barriers to cardiac rehabilitation and patient perceptions on the usage of technologies in cardiac rehabilitation: A cross-sectional study | 10.1111/jocn.16919 |  | Not at all relevant |  |  |  |
| Reliability (or lack thereof) of smartphone ecological momentary assessment of visual dot probe attention bias toward threat indices | 10.1016/j.jbtep.2023.101918 |  | Not at all relevant |  |  |  |
| 'Been there, experienced that': A qualitative study on the experiences and perceptions of online peer volunteers in supporting Singaporean mothers at risk of depression | 10.1111/jan.15867 |  | Not at all relevant |  |  |  |
| Disorganized attachment in emerging adulthood: Measurement comparisons and relations to childhood maltreatment and emotion dysregulation | 10.1111/famp.12866 |  | Not at all relevant |  |  |  |
| Effectiveness-implementation hybrid trial of Spanish language, digital cognitive-behavioral therapy (dCBT) intervention for depression and anxiety - protocol for the SUPERA (SUpport from PEeRs to expand Access) study | 10.1016/j.cct.2023.107422 |  | Not at all relevant |  |  |  |
| The efficacy of mindfulness apps on symptoms of depression and anxiety: An updated meta-analysis of randomized controlled trials | 10.1016/j.cpr.2023.102370 |  |  | No Mobile app |  |  |
| Orally administered selenium-containing α-D-1,6-glucan and α-D-1,6-glucan relief early cognitive deficit in APP/PS1 mice | 10.1016/j.ijbiomac.2023.128539 |  | Not at all relevant |  |  |  |
| Effectiveness of nurse-led electronic health interventions on illness management in patients with chronic heart failure: A systematic review and meta-analysis | 10.1016/j.ijnurstu.2023.104630 | Review paper |  |  |  |  |
| Nurse escorts' perceptions of nurse-led inter-hospital ambulance transfer in the Wheatbelt region of Western Australia: A descriptive survey study | 10.1111/ajr.13067 |  | Not at all relevant |  |  |  |
| Connection at your fingertips: A first look at the Agapé app's contributions to healthy relationships | 10.1037/fam0001166 |  | Not at all relevant |  |  |  |
| Frequency and predictors of virtual hope box use in individuals experiencing suicidal ideation: An ecological momentary assessment investigation | 10.1111/sltb.13018 |  | Not at all relevant |  |  |  |
| Protective effects of SSRI, Citalopram in mutant APP and mutant Tau expressed dorsal raphe neurons in Alzheimer's disease | 10.1016/j.bbadis.2023.166942 |  | Not at all relevant |  |  |  |
| ESSENCE: An Implementation Research Program to Scale Up Depression Care in Rural Communities | 10.1176/appi.ps.202100223 |  | Not at all relevant |  |  |  |
| Gastric Alimetry Expands Patient Phenotyping in Gastroduodenal Disorders Compared with Gastric Emptying Scintigraphy | 10.14309/ajg.0000000000002528 |  | Not at all relevant |  |  |  |
| Social Interactions in Everyday Life of Socially Anxious Adolescents: Effects on Mental State, Anxiety, and Depression | 10.1007/s10802-023-01121-5 |  |  | Screening, no treatment |  |  |
| Changes in affective control covary with changes in mental health difficulties following affective control training (AffeCT) in adolescents | 10.1017/S0033291723002167 |  |  | No Mobile app |  |  |
| Acceptability and Feasibility of Text Message Interface to Assess Parents' Real-time PICU Experiences | 10.1177/08850666231195754 |  | Not at all relevant |  |  |  |
| Association of Arizona's Implementation of a Behavioral Health Crisis Response System With Suicide Hospitalizations | 10.1176/appi.ps.20220628 |  | Not at all relevant |  |  |  |
| Micro-sequences of anger and shame and non-suicidal self-injury in youth: an ecological momentary assessment study | 10.1111/jcpp.13869 |  |  | No Mobile app |  |  |
| [An urban-rural comparison of family stress and early childhood intervention: Data linkage between two cross-sectional studies] | 10.1024/1012-5302/a000918 |  | Not at all relevant |  |  |  |
| Assessing Visual Avoidance of Faces During Real-Life Social Stress in Children with Social Anxiety Disorder: A Mobile Eye-Tracking Study | 10.1007/s10578-022-01383-y |  | Not at all relevant |  |  |  |
| Effectiveness of technology-based psychosocial interventions on diabetes distress and health-relevant outcomes among type 2 diabetes mellitus: A systematic review and meta-analysis | 10.1177/1357633X211058329 | Review paper |  |  |  |  |
| Pilot pragmatic randomized trial of mHealth mindfulness-based intervention for advanced cancer patients and their informal caregivers | 10.1002/pon.5557 |  |  | App usability |  |  |
| Feasibility and uptake of a digital mental health intervention for depression among Lebanese and Syrian displaced people in Lebanon: a qualitative study | 10.3389/fpubh.2023.1293187 |  |  | App usability |  |  |
| Development of a novel UHPLC-MS/MS method for the quantification of corynoxeine: Application to pharmacokinetics and tissue distribution studies in normal and chronic unpredictable mild stress-induced depression rats | 10.1016/j.jpba.2023.115850 |  | Not at all relevant |  |  |  |
| Qingxin Kaiqiao Fang decreases Tau hyperphosphorylation in Alzheimer's disease via the PI3K/Akt/GSK3β pathway in vitro and in vivo | 10.1016/j.jep.2023.117031 |  | Not at all relevant |  |  |  |
| Antidepressant sertraline increases thioflavin-S and Congo red deposition in APPswe/PSEN1dE9 transgenic mice | 10.3389/fphar.2023.1260838 |  | Not at all relevant |  |  |  |
| Current practices of psychoeducation interventions with persons with bipolar disorders: a literature review | 10.3389/fpsyt.2023.1320654 | Review paper |  |  |  |  |
| Availability of Internet-Based Cognitive-Behavioral Therapies for Depression: A Systematic Review | 10.1016/j.beth.2023.06.003 | Review paper |  |  |  |  |
| Smartphone-based safety plan for suicidal crisis: The SmartCrisis 2.0 pilot study | 10.1016/j.jpsychires.2023.11.039 |  |  | App usability |  |  |
| Increasing postpartum depression screening and resources in pediatric primary care: A quality improvement project to enhance provider confidence | 10.1016/j.pedn.2023.10.040 |  |  | No Mobile app |  |  |
| Associations with smartphone usage and life satisfaction among older adults: Mediating roles of depressive symptoms and cognitive function | 10.1016/j.gerinurse.2023.11.013 |  | Not at all relevant |  |  |  |
| Exposure traced in daily life: improvements in ecologically assessed social and physical activity following exposure-based psychotherapy for anxiety disorders | 10.1016/j.janxdis.2023.102792 |  |  | No Mobile app |  |  |
| Tanshinone IIA, the key compound in Salvia miltiorrhiza, improves cognitive impairment by upregulating Aβ-degrading enzymes in APP/PS1 mice | 10.1016/j.ijbiomac.2023.127923 |  | Not at all relevant |  |  |  |
| Adaptive Nutrition Intervention Stabilizes Serum Phosphorus Levels in Hemodialysis Patients: A Multicenter Decentralized Clinical Trial Using Real-World Data | 10.1053/j.jrn.2023.07.004 |  | Not at all relevant |  |  |  |
| The Patient-Determined Disease Steps scale is not interchangeable with the Expanded Disease Status Scale in mild to moderate multiple sclerosis | 10.1111/ene.16046 |  | Not at all relevant |  |  |  |
| Attributes of Provider Referrals for Digital Mental Health Applications in an Integrated Health System, 2019-2021 | 10.1176/appi.ps.20220401 | Review paper |  |  |  |  |
| Civilian Moral Injury and Amygdala Functional Connectivity During Attention to Threat | 10.1016/j.bpsc.2023.07.006 |  | Not at all relevant |  |  |  |
| Feasibility, Acceptability, and Health Outcomes Associated With Telehealth for Children in Families With Limited English Proficiency | 10.1016/j.acap.2023.06.025 |  | Not at all relevant |  |  |  |
| The effect of digital health interventions on postpartum depression or anxiety: a systematic review and meta-analysis of randomized controlled trials | 10.1016/j.ajog.2023.06.028 | Review paper |  |  |  |  |
| Discriminatory Experiences Among Black Youth: How Encounters and Expectations Explain Emotional Well-Being | 10.1007/s11121-023-01540-2 |  | Not at all relevant |  |  |  |
| A Mobile Health Behavior Change Intervention for Women With Coronary Heart Disease: A RANDOMIZED CONTROLLED PILOT STUDY | 10.1097/HCR.0000000000000804 |  | Not at all relevant |  |  |  |
| Factors influencing engagement in in-person and remotely delivered lifestyle interventions for young adults with serious mental illness: A qualitative study | 10.1111/eip.13432 |  |  | Telehealth app |  |  |
| The Feasibility and Effects of Smartphone-Based Application on Cardiac Rehabilitation for Patients After Percutaneous Coronary Intervention: A Randomized Controlled Trial | 10.1097/JCN.0000000000000993 |  | Not at all relevant |  |  |  |
| Dating Apps: A New Emerging Platform for Sexual Harassment? A Scoping Review | 10.1177/15248380231162969 | Review paper |  |  |  |  |
| Perceptions of Helpful and Unhelpful Responses to Disclosures of Suicidality in a Sample of Mobile App Users | 10.1080/13811118.2022.2152984 |  | Not at all relevant |  |  |  |
| Geri-Mobile Health: Feasibility of a VA Mental Health Mobile Apps Coaching Program for Older Veterans | 10.1080/07317115.2022.2115433 |  | Not at all relevant |  |  |  |
| Digital Phenotyping for Mood Disorders: Methodology-Oriented Pilot Feasibility Study | 10.2196/47006 |  | Not at all relevant |  |  |  |
| Effect of digital monitoring and counselling on self-management ability in patients with rheumatoid arthritis: a randomised controlled trial | 10.1093/rheumatology/kead709 |  | Not at all relevant |  |  |  |
| Lived experience codesign of self-harm interventions: a scoping review | 10.1136/bmjopen-2023-079090 | Review paper |  |  |  |  |
| Digital technology and mental health during the COVID-19 pandemic: a narrative review with a focus on depression, anxiety, stress, and trauma | 10.3389/fpsyt.2023.1227426 | Review paper |  |  |  |  |
| Study protocol for a feasibility study of microinterventions in smartphone-based assessments to reduce depressive rumination | 10.1136/bmjopen-2023-076031 |  |  | No Mobile app |  |  |
| Assessing the Impact of Evidence-Based Mental Health Guidance During the COVID-19 Pandemic: Systematic Review and Qualitative Evaluation | 10.2196/52901 | Review paper |  |  |  |  |
| Classifying and clustering mood disorder patients using smartphone data from a feasibility study | 10.1038/s41746-023-00977-7 |  |  | Screening, no treatment |  |  |
| Systematic review and meta-analysis of AI-based conversational agents for promoting mental health and well-being | 10.1038/s41746-023-00979-5 | Review paper |  |  |  |  |
| Evaluation of the Current State of Chatbots for Digital Health: Scoping Review | 10.2196/47217 | Review paper |  |  |  |  |
| The Contribution of the Internet to Promoting Mental Health for Older Adults: Cross-Sectional Survey in China | 10.2196/40172 |  | Not at all relevant |  |  |  |
| An umbrella review of effectiveness and efficacy trials for app-based health interventions | 10.1038/s41746-023-00981-x | Review paper |  |  |  |  |
| Experience of primary healthcare workers in using the mobile app-based WHO mhGAP intervention guide in detection and treatment of people with mental disorders: A qualitative study in Nepal | 10.1016/j.ssmmh.2023.100278 |  |  |  | No treatment |  |
| Expanding the UTAUT2 framework to determine the drivers of mobile shopping behaviour among older adults | 10.1371/journal.pone.0295581 |  | Not at all relevant |  |  |  |
| Phone-Based Text Therapy for Youth Mental Health: Rapid Review | 10.2196/47250 | Review paper |  |  |  |  |
| Blended-ALMAMAR app for inpatient mental health care for refugees: study protocol for a multicenter implementation study within the I-REACH consortium (Internet based REfugee mentAl healtH Care) | 10.1186/s12913-023-10403-z |  | Not at all relevant |  |  |  |
| Online Support Groups for Family Caregivers: Scoping Review | 10.2196/46858 | Review paper |  |  |  |  |
| A Personalized, Transdiagnostic Smartphone Intervention (Mello) Targeting Repetitive Negative Thinking in Young People With Depression and Anxiety: Pilot Randomized Controlled Trial | 10.2196/47860 |  |  |  |  | 1 |
| Safety Evaluation in Iterative Development of Wearable Patches for Aripiprazole Tablets With Sensor: Pooled Analysis of Clinical Trials | 10.2196/44768 |  | Not at all relevant |  |  |  |
| Using deep long-read RNAseq in Alzheimer's disease brain to assess medical relevance of RNA isoform diversity | 10.1101/2023.08.06.552162 |  | Not at all relevant |  |  |  |
| Secure Messaging Intervention in Patients Starting New Antidepressant to Promote Adherence: Pilot Randomized Controlled Trial | 10.2196/51277 |  | Not at all relevant |  |  |  |
| Social Media Use and Its Concurrent and Subsequent Relation to a Biological Marker of Inflammation: Short-Term Longitudinal Study | 10.2196/46309 |  | Not at all relevant |  |  |  |
| Altered synaptic plasticity at hippocampal CA1-CA3 synapses in Alzheimer's disease: integration of amyloid precursor protein intracellular domain and amyloid beta effects into computational models | 10.3389/fncom.2023.1305169 |  | Not at all relevant |  |  |  |
| Technology engagement is associated with higher perceived physical well-being in stroke patients prescribed smartwatches for atrial fibrillation detection | 10.3389/fdgth.2023.1243959 |  | Not at all relevant |  |  |  |
| The Impact of Generative Conversational Artificial Intelligence on the Lesbian, Gay, Bisexual, Transgender, and Queer Community: Scoping Review | 10.2196/52091 | Review paper |  |  |  |  |
| Nomophobia and Its Relationship with Social Anxiety and Procrastination in Nursing Students: An Observational Study | 10.3390/nursrep13040140 |  | Not at all relevant |  |  |  |
| Predictors of problematic smartphone use among young adult lesbian, gay and bisexual individuals during the COVID-19 pandemic: a four-year follow-up study | 10.1186/s12888-023-05326-2 |  | Not at all relevant |  |  |  |
| Using wearable activity trackers for research in the global south: Lessons learned from adolescent psychotherapy research in Kenya | 10.1017/gmh.2023.85 |  | Not at all relevant |  |  |  |
| Patient-driven decisions and perceptions of the 'safest possible choice': insights from patient-provider conversations about how some breast cancer patients choose contralateral prophylactic mastectomy | 10.1080/08870446.2023.2290170 |  | Not at all relevant |  |  |  |
| Effectiveness of a universal personalized intervention for the prevention of anxiety disorders: Protocol of a randomized controlled trial (the prevANS project) | 10.1016/j.invent.2023.100640 |  | Not at all relevant |  |  |  |
| Efficacy and acceptability of mobile application-delivered acceptance and commitment therapy for posttraumatic stress disorder in China: A randomized controlled trial | 10.1016/j.brat.2023.104440 |  |  |  | No app |  |
| Mobile health interventions in multiple sclerosis: A systematic review | 10.1177/13524585231201089 | Review paper |  |  |  |  |
| Specific associations of passively sensed smartphone data with future symptoms of avoidance, fear, and physiological distress in social anxiety | 10.1016/j.invent.2023.100683 |  |  | No depression,anxiety,stress |  |  |
| Effects of a Cognitive Behavioral Digital Therapeutic on Anxiety and Depression Symptoms in Patients With Cancer: A Randomized Controlled Trial | 10.1200/OP.23.00210 |  |  | Telehealth app |  |  |
| Black American women's attitudes toward seeking mental health services and use of mobile technology to support the management of anxiety | 10.1093/jamiaopen/ooad088 |  |  | Screening, no treatment |  |  |
| How should narcissism be treated best? | 10.1016/S2215-0366(23)00307-3 |  | Not at all relevant |  |  |  |
| Acceptability of a smartphone-based intervention targeting anxiety sensitivity among women receiving emergency care after sexual assault: A pilot uncontrolled trial | 10.1002/jts.22974 |  | Not at all relevant |  |  |  |
| Identification of demyelinating lesions and application of McDonald criteria when confronted with white matter lesions on brain MRI | 10.1016/j.neurol.2023.04.006 |  | Not at all relevant |  |  |  |
| Application of mobile-based web app to enhance simple suturing skills of nurse practitioners | 10.1016/j.nedt.2023.105959 |  | Not at all relevant |  |  |  |
| Evaluation of Montreal Cognitive Assessment (MoCA) Administered via Videoconference | 10.1016/j.jamda.2023.08.015 |  | Not at all relevant |  |  |  |
| Impairment of EQ-5D-5L Domains According to Allergic Rhinitis and Asthma Control: A MASK-air Real-World Study | 10.1016/j.jaip.2023.08.006 |  | Not at all relevant |  |  |  |
| Heterogeneity of Response to Methylphenidate in Apathetic Patients in the ADMET 2 Trial | 10.1016/j.jagp.2023.06.002 |  | Not at all relevant |  |  |  |
| The impact of climate change on country and community and the role of mental health professionals working with Aboriginal communities in recovery and promoting resilience | 10.1111/inm.13184 |  | Not at all relevant |  |  |  |
| Understanding nurse suicide using an ideation-to-action framework: An integrative review | 10.1111/jan.15681 | Review paper |  |  |  |  |
| HIV Treatment and Mental Health Outcomes Among Gay, Bisexual, and Other Men Who Have Sex With Men Living With HIV in a Pilot Multicomponent Intervention in Guatemala City | 10.1177/10901981231164598 |  | Not at all relevant |  |  |  |
| Digital health technologies and major depressive disorder | 10.1017/S1092852923002225 | Review paper |  |  |  |  |
| Internal capsule microstructure mediates the relationship between childhood maltreatment and PTSD following adulthood trauma exposure | 10.1038/s41380-023-02012-3 |  | Not at all relevant |  |  |  |
| Adapting a Counseling-Plus-mHealth Intervention for the Virtual Environment to Reduce Sexual and Reproductive Health Risk Among Young Women with Depression | 10.1007/s11121-023-01506-4 |  | Not at all relevant |  |  |  |
| Self-reported efficacy in patient-physician interaction in relation to anxiety, patient activation, and health-related quality of life among stroke survivors | 10.1080/07853890.2022.2159516 |  | Not at all relevant |  |  |  |
| Rapid, reliable mobile assessment of affect-related motor processing | 10.3758/s13428-022-02015-y |  | Not at all relevant |  |  |  |
| Social media-based interventions for patients with cancer: a meta-analysis and meta-regression of randomised controlled trials | 10.1007/s11764-022-01244-6 |  | Not at all relevant |  |  |  |
| Supporting women's health outcomes after breast cancer treatment comparing a text message intervention to usual care: the EMPOWER-SMS randomised clinical trial | 10.1007/s11764-022-01209-9 |  | Not at all relevant |  |  |  |
| Sadness-Based Approach-Avoidance Modification Training for Subjective Stress in Adults: Pilot Randomized Controlled Trial | 10.2196/50324 |  | Not at all relevant |  |  |  |
| User-Centered Design of a Gamified Mental Health App for Adolescents in Sub-Saharan Africa: Multicycle Usability Testing Study | 10.2196/51423 |  |  | App usability |  |  |
| Smartphone keyboard dynamics predict affect in suicidal ideation | 10.1101/2023.11.29.23299169 |  | Not at all relevant |  |  |  |
| The ChAMP App: A Scalable mHealth Technology for Detecting Digital Phenotypes of Early Childhood Mental Health | 10.1101/2023.01.19.23284753 |  | Not at all relevant |  |  |  |
| Testing a Home Solution for Preparing Young Children for an Awake MRI: A Promising Smartphone Application | 10.3390/children10121866 |  | Not at all relevant |  |  |  |
| Mediating Effect of Stress Recognition on the Effect of Generalized Anxiety Disorder on Smartphone Dependence | 10.3390/jcm12237359 |  |  | Mobile addiction |  |  |
| Factors affecting social phobia among Chinese college students in the context of COVID-19 pandemic: a cross-sectional study | 10.1038/s41598-023-48225-y |  | Not at all relevant |  |  |  |
| Automated Machine Learning Analysis of Patients With Chronic Skin Disease Using a Medical Smartphone App: Retrospective Study | 10.2196/50886 |  | Not at all relevant |  |  |  |
| Self-Reported Medication Use Across Racial and Rural or Urban Subgroups of People Who Are Pregnant in the United States: Decentralized App-Based Cohort Study | 10.2196/50867 |  | Not at all relevant |  |  |  |
| Longitudinal outcome monitoring in patients with chronic gastroduodenal symptoms investigated using the Gastric Alimetry system: study protocol | 10.1136/bmjopen-2023-074462 |  | Not at all relevant |  |  |  |
| Impact of mobile connectivity on students' wellbeing: Detecting learners' depression using machine learning algorithms | 10.1371/journal.pone.0294803 |  | Not at all relevant |  |  |  |
| The feasibility, acceptability and efficacy of an app-based intervention (the Coping Camp) in reducing stress among Chinese school adolescents: A cluster randomised controlled trial | 10.1371/journal.pone.0294119 |  |  |  |  | 1 |
| Nomophobia and Its Association with Depression, Anxiety and Stress (DASS Scale), among Young Adults in Greece | 10.3390/ejihpe13120191 |  |  | Mobile addiction |  |  |
| Efficacy of smartphone application-based multi-domain cognitive training in older adults without dementia | 10.3389/fnagi.2023.1250420 |  |  | No depression,anxiety,stress |  |  |
| Do community-based singing interventions have an impact on people living with dementia and their carers? A mixed-methods study protocol | 10.1136/bmjopen-2023-076168 |  | Not at all relevant |  |  |  |
| Technology use and attitudes towards digital mental health in people with severe mental health problems: a survey study in China | 10.3389/fpsyt.2023.1261795 |  |  | No Mobile app |  |  |
| Effect of a monitored home-based exercise program combined with a behavior change intervention and a smartphone app on walking distances and quality of life in adults with peripheral arterial disease: the WalkingPad randomized clinical trial | 10.3389/fcvm.2023.1272897 |  | Not at all relevant |  |  |  |
| Self-Guided Digital Intervention for Depression in Adolescents: Feasibility and Preliminary Efficacy Study | 10.2196/43260 |  |  |  | No app |  |
| The Impact of Digital Technology on Self-Management in Cancer: Systematic Review | 10.2196/45145 | Review paper |  |  |  |  |
| Musculoskeletal Rehabilitation: New Perspectives in Postoperative Care Following Total Knee Arthroplasty Using an External Motion Sensor and a Smartphone Application for Remote Monitoring | 10.3390/jcm12227163 |  | Not at all relevant |  |  |  |
| Association between microbiome and the development of adverse posttraumatic neuropsychiatric sequelae after traumatic stress exposure | 10.1038/s41398-023-02643-8 |  | Not at all relevant |  |  |  |
| mHealth Intervention to Promote Physical Activity Among Employees Using a Deep Learning Model for Passive Monitoring of Depression and Anxiety: Single-Arm Feasibility Trial | 10.2196/51334 |  | Not at all relevant |  |  |  |
| Randomized Clinical Trial on the Comparison of Effect of Asynchronous Mobile Application and Guided Brief Cognitive Behavioral Therapy in Managing Anxiety among Medical Students | 10.47626/2237-6089-2023-0713 |  |  |  |  | 1 |
| Home-Based Treatment for Chronic Pain Combining Neuromodulation, Computer-Assisted Training, and Telemonitoring in Patients With Breast Cancer: Protocol for a Rehabilitative Study | 10.2196/49508 |  | Not at all relevant |  |  |  |
| Assessing the Efficacy and Safety of a Digital Therapeutic for Symptoms of Depression in Adolescents: Protocol for a Randomized Controlled Trial | 10.2196/48740 |  |  | App usability |  |  |
| Examining the Feasibility of Implementing Digital Mental Health Innovations Into Hospitals to Support Youth in Suicide Crisis: Interview Study With Young People and Health Professionals | 10.2196/51398 |  | Not at all relevant |  |  |  |
| Screening for Psychological Distress in Healthcare Workers Using Machine Learning: A Proof of Concept | 10.1007/s10916-023-02011-5 |  |  | Screening, no treatment |  |  |
| Use of Assistive Technology for Persons with Psychosocial Disability: Systematic Review | 10.2196/49750 | Review paper |  |  |  |  |
| Multilingual markers of depression in remotely collected speech samples: A preliminary analysis | 10.1016/j.jad.2023.08.097 |  | Not at all relevant |  |  |  |
| Feasibility and Usability of the Job Adjustment Mobile App for Pregnant Women: Longitudinal Observational Study | 10.2196/48637 |  | Not at all relevant |  |  |  |
| Facilitated WhatsApp Support Groups for Youth Living With HIV in Nairobi, Kenya: Single-Arm Pilot Intervention Study | 10.2196/49174 |  | Not at all relevant |  |  |  |
| The Effects of Remote Cognitive Training Combined With a Mobile App Intervention on Psychosis: Double-Blind Randomized Controlled Trial | 10.2196/48634 |  |  | No depression,anxiety,stress |  |  |
| Repeated multi-domain cognitive training prevents cognitive decline, anxiety and amyloid pathology found in a mouse model of Alzheimer disease | 10.1038/s42003-023-05506-6 |  | Not at all relevant |  |  |  |
| Mobile Phone App-Based or Face-to-Face Pulmonary Rehabilitation in COVID-19 Survivors | 10.4103/ijnmr.ijnmr_337_22 |  | Not at all relevant |  |  |  |
| Predictors of mental health problems during the COVID-19 outbreak in Egypt in 2021 | 10.3389/fpubh.2023.1234201 |  | Not at all relevant |  |  |  |
| Association Between Concerns About COVID-19 Infection and Blood Donation Intention: Cross-Sectional Survey Study Through a Mobile Communication Platform | 10.2196/46588 |  | Not at all relevant |  |  |  |
| (No) Effects of a Self-Kindness Intervention on Self-Esteem and Visual Self-Perception: An Eye-Tracking Investigation on the Time-Course of Self-Face Viewing | 10.3390/ejihpe13110179 |  | Not at all relevant |  |  |  |
| An Internet- and Kinect-Based Multiple Sclerosis Fitness Intervention Training With Pilates Exercises: Development and Usability Study | 10.2196/41371 |  | Not at all relevant |  |  |  |
| Wearable Artificial Intelligence for Detecting Anxiety: Systematic Review and Meta-Analysis | 10.2196/48754 | Review paper |  |  |  |  |
| Elevated emotion network connectivity is associated with fluctuations in depression | 10.1073/pnas.2216499120 |  | Not at all relevant |  |  |  |
| Effects of Two Cannabidiol Oil Products on Self-Reported Stress Relief: A Quasi-Experimental Study | 10.1159/000531886 |  | Not at all relevant |  |  |  |
| Effectiveness of Functional or Aerobic Exercise Combined With Breathing Techniques in Telerehabilitation for Patients With Long COVID: A Randomized Controlled Trial | 10.1093/ptj/pzad118 |  | Not at all relevant |  |  |  |
| Using WhatsApp support groups to promote responsive caregiving, caregiver mental health and child development in the COVID-19 era: A randomised controlled trial of a fully digital parenting intervention | 10.1177/20552076231203893 |  |  | Telehealth app |  |  |
| Synchronous Web-Based Psychotherapy for Mental Disorders From a Health Quality Perspective: Scoping Review | 10.2196/40710 | Review paper |  |  |  |  |
| Adolescent Use of Dating Applications and the Associations with Online Victimization and Psychological Distress | 10.3390/bs13110903 |  | Not at all relevant |  |  |  |
| Dismantling and personalising task-sharing psychosocial interventions for common mental disorders: a study protocol for an individual participant data component network meta-analysis | 10.1136/bmjopen-2023-077037 |  | Not at all relevant |  |  |  |
| Preliminary Validation of The Eating Disorders Examination Questionnaire-Short Parent Version (EDE-QS-P) | 10.1080/10640266.2023.2218675 |  | Not at all relevant |  |  |  |
| At-home use of app-based mindfulness for children: A randomized active-controlled trial | 10.1007/s12671-023-02231-3 |  | Not at all relevant |  |  |  |
| Adolescent Social Communication Through Smartphones: Linguistic Features of Internalizing Symptoms and Daily Mood | 10.1177/21677026221125180 |  |  | Screening, no treatment |  |  |
| Relationship between Anxiety and Problematic Smartphone Use in First-Year Junior High School Students: Moderated Mediation Effects of Physical Activity and School Adjustment | 10.3390/bs13110901 |  |  | Mobile addiction |  |  |
| Untangling the association between COVID-19 health literacy, trust in the pandemic response, and mental distress, during the COVID-19 pandemic in Ireland: a repeated cross-sectional study | 10.1016/S0140-6736(23)02120-7 |  | Not at all relevant |  |  |  |
| App-Based Interventions for Moderate to Severe Depression: A Systematic Review and Meta-Analysis | 10.1001/jamanetworkopen.2023.44120 | Review paper |  |  |  |  |
| A smartphone- and wearable-based biomarker for the estimation of unipolar depression severity | 10.1038/s41598-023-46075-2 |  | Not at all relevant |  |  |  |
| Self-Care Mental Health App Intervention for Post-Intensive Care Syndrome-Family: A Randomized Pilot Study | 10.4037/ajcc2023800 |  |  | No Mobile app |  |  |
| User engagement in a randomised controlled trial for a digital health intervention for early psychosis (Actissist 2.0 trial) | 10.1016/j.psychres.2023.115536 |  |  | App usability |  |  |
| The associations of parental attitudes and peer bullying with alexithymia in adolescents: A structural equality model | 10.1016/j.pedn.2023.10.003 |  | Not at all relevant |  |  |  |
| Comprehensive analysis of long COVID in a Japanese nationwide prospective cohort study | 10.1016/j.resinv.2023.08.008 |  | Not at all relevant |  |  |  |
| Understanding the relationship between time spent outdoors, mental well-being and health-related behaviours in a Spanish sample: A real time smartphone-based study | 10.1016/j.psychres.2023.115494 |  |  | Screening, no treatment |  |  |
| Feasibility, acceptability and preliminary efficacy of a mental health self-management app in clinicians working during the COVID-19 pandemic: A pilot randomised controlled trial | 10.1016/j.psychres.2023.115493 |  |  |  |  | 1 |
| The cost-effectiveness of a real-time seizure detection application for people with epilepsy | 10.1016/j.yebeh.2023.109441 |  | Not at all relevant |  |  |  |
| Feasibility of Symptom Monitoring During the First Year of Endocrine Therapy for Early Breast Cancer Using Patient-Reported Outcomes Collected via Smartphone App | 10.1200/OP.23.00038 |  | Not at all relevant |  |  |  |
| Mechanisms underlying large-leaf yellow tea mediated inhibition of cognitive impairment in the 5xFAD model of Alzheimer's disease | 10.1016/j.phymed.2023.155030 |  | Not at all relevant |  |  |  |
| Effects of 12 Weeks of At-Home, Application-Based Exercise on Health Care Workers' Depressive Symptoms, Burnout, and Absenteeism: A Randomized Clinical Trial | 10.1001/jamapsychiatry.2023.2706 |  |  |  |  | 1 |
| Capturing mood dynamics through adolescent smartphone social communication | 10.1037/abn0000855 |  |  | Screening, no treatment |  |  |
| An Affordable Platform for Virtual Reality-Based Patient Education in Radiation Therapy | 10.1016/j.prro.2023.06.008 |  | Not at all relevant |  |  |  |
| Temporal Facelift: A New Method for Temporal and Mid-face Lifting | 10.1097/SCS.0000000000009525 |  | Not at all relevant |  |  |  |
| A noninferiority trial on information-based video versus self-selected video distraction technique for preoperative anxiety reduction in school children: Prepare trial | 10.1111/pan.14718 |  | Not at all relevant |  |  |  |
| Youth Mobile Response and Stabilization Services: Factors Associated with Multiple Episodes of Care | 10.1007/s10597-023-01153-7 |  | Not at all relevant |  |  |  |
| Suicide Safety Plan Self-knowledge in Serious Mental Illness: Psychiatric Symptom Correlates and Effects of Brief Intervention | 10.1007/s10597-023-01155-5 |  |  |  | No treatment |  |
| Telehealth in treating tinnitus: a systematic review and meta-analysis | 10.1017/S0022215123000373 | Review paper |  |  |  |  |
| Suicide Misconceptions and Attitudes Toward Suicide Prevention Measures in Taiwan | 10.1027/0227-5910/a000893 |  | Not at all relevant |  |  |  |
| Neighborhood disadvantage, household chaos, and personal stressors: exploring early-life contextual factors and current mental health symptoms in college students | 10.1080/07448481.2021.1970564 |  | Not at all relevant |  |  |  |
| Examining terror management theory in Ukraine: impact of air-raid alarms and explosions on mental health, somatic symptoms, and well-being | 10.3389/fpsyt.2023.1244335 |  | Not at all relevant |  |  |  |
| Improving Depression Severity Prediction from Passive Sensing: Symptom-Profiling Approach | 10.3390/s23218866 |  | Not at all relevant |  |  |  |
| Systematic review on the effectiveness of mobile health applications on mental health of breast cancer survivors | 10.1007/s11764-023-01470-6 | Review paper |  |  |  |  |
| The Efficacy of Little Lovely Dentist and Tell Show Do in Alleviating Dental Anxiety in Iraqi Children: A Randomized Clinical Trial | 10.4103/jispcd.JISPCD_112_23 |  | Not at all relevant |  |  |  |
| The effects of short video app-guided loving-kindness meditation on college students' mindfulness, self-compassion, positive psychological capital, and suicide ideation | 10.1186/s41155-023-00276-w |  |  |  |  | 1 |
| HIV-related stigma, depression and suicidal ideation among HIV-positive MSM in China: a moderated mediation model | 10.1186/s12889-023-17047-y |  | Not at all relevant |  |  |  |
| Evaluation of Menstrual Cycle Tracking Behaviors in the Ovulation and Menstruation Health Pilot Study: Cross-Sectional Study | 10.2196/42164 |  | Not at all relevant |  |  |  |
| Optimization of a Transdiagnostic Mobile Emotion Regulation Intervention for University Students: Protocol for a Microrandomized Trial | 10.2196/46603 |  | Not at all relevant |  |  |  |
| Reducing Dental Anxiety in Children Using a Mobile Health App: Usability and User Experience Study | 10.2196/30443 |  | Not at all relevant |  |  |  |
| Investigation of anti-depression effects and potential mechanisms of the ethyl acetate extract of Cynomorium songaricum Rupr. through the integration of in vivo experiments, LC-MS/MS chemical analysis, and a systems biology approach | 10.3389/fphar.2023.1239197 |  | Not at all relevant |  |  |  |
| Improving Mild to Moderate Depression With an App-Based Self-Guided Intervention: Protocol for a Randomized Controlled Trial | 10.2196/46651 |  |  |  | No treatment |  |
| Challenges With the Use of Digital Sham: Systematic Review and Recommendations | 10.2196/44764 | Review paper |  |  |  |  |
| Comparing the Acceptability and Quality of Intervention Modalities for Suicidality in the Emergency Department: Randomized Feasibility Trial | 10.2196/49783 |  | Not at all relevant |  |  |  |
| Real-Time Detection of Emotions Based on Facial Expression for Mental Health | 10.3233/SHTI230795 |  | Not at all relevant |  |  |  |
| Mind Note - Application for Mental Health Support | 10.3233/SHTI230771 |  |  | Screening, no treatment |  |  |
| Developing Advanced AI Ecosystems to Enhance Diagnosis and Care for Patients with Depression | 10.3233/SHTI230731 |  | Not at all relevant |  |  |  |
| Intermittent hypoxia therapy ameliorates beta-amyloid pathology via TFEB-mediated autophagy in murine Alzheimer's disease | 10.1186/s12974-023-02931-6 |  | Not at all relevant |  |  |  |
| Enhancing panic disorder treatment with mobile-aided case management: an exploratory study based on a 3-year cohort analysis | 10.3389/fpsyt.2023.1203194 |  | Not at all relevant |  |  |  |
| Compas-Y: A mixed methods pilot evaluation of a mobile self-compassion training for people with newly diagnosed cancer | 10.1177/20552076231205272 |  | Not at all relevant |  |  |  |
| Anxiety levels among school-going adolescents in peri-urban areas of Karachi, Pakistan | 10.1371/journal.pone.0289967 |  | Not at all relevant |  |  |  |
| Can mobile-health applications contribute to long-term increase in physical activity after medical rehabilitation?-A pilot-study | 10.1371/journal.pdig.0000359 |  | Not at all relevant |  |  |  |
| An Exposure-Based Video Game (Dr. Zoo) to Reduce Needle Phobia in Children Aged 3 to 6 Years: Development and Mixed Methods Pilot Study | 10.2196/42025 |  | Not at all relevant |  |  |  |
| Simultaneous separation and detection of nine kynurenine pathway metabolites by reversed-phase liquid chromatography-mass spectrometry: Quantitation of inflammation in human cerebrospinal fluid and plasma | 10.1016/j.aca.2023.341659 |  | Not at all relevant |  |  |  |
| Study protocol: combined N-of-1 trials to assess open-label placebo treatment for antidepressant discontinuation symptoms [FAB-study] | 10.1186/s12888-023-05184-y |  | Not at all relevant |  |  |  |
| User Engagement Clusters of an 8-Week Digital Mental Health Intervention Guided by a Relational Agent (Woebot): Exploratory Study | 10.2196/47198 |  |  | Screening, no treatment |  |  |
| Absolute and relative preferences for mobile phone internet content, mobile phone dependence, and depressive symptoms: a study of Chinese university students in the post-pandemic era | 10.3389/fpubh.2023.1247438 |  |  | Mobile addiction |  |  |
| Superior semicircular canal dehiscence and subsequent closure induces reversible impaired decision-making | 10.3389/fneur.2023.1259030 |  | Not at all relevant |  |  |  |
| Third-wave cognitive behavioral therapies for caregivers of cancer patients: a scoping review | 10.1186/s12906-023-04186-3 | Review paper |  |  |  |  |
| Patient Experience of Digitalized Follow-up of Antidepressant Treatment in Psychiatric Outpatient Care: Qualitative Analysis | 10.2196/48843 |  | Not at all relevant |  |  |  |
| A Digital Mental Health Support Program for Depression and Anxiety in Populations With Attention-Deficit/Hyperactivity Disorder: Feasibility and Usability Study | 10.2196/48362 |  |  | No depression,anxiety,stress |  |  |
| Short-term exposure sequences and anxiety symptoms: a time series clustering of smartphone-based mobility trajectories | 10.1186/s12942-023-00348-1 |  |  | Screening, no treatment |  |  |
| Digital Phenotyping for Monitoring and Disease Trajectory Prediction of Patients With Cancer: Protocol for a Prospective Observational Cohort Study | 10.2196/49096 |  | Not at all relevant |  |  |  |
| Dynamics of depressive states among university students in Japan during the COVID-19 pandemic: an interrupted time series analysis | 10.1186/s12991-023-00468-9 |  | Not at all relevant |  |  |  |
| Patient Anxiety and Communication Experience in the Emergency Department: A Mobile, Web-Based, Mixed-Methods Study on Patient Isolation During the COVID-19 Pandemic | 10.3346/jkms.2023.38.e303 |  | Not at all relevant |  |  |  |
| Evaluation on interactive waiting experience design of mobile internet products based on machine learning | 10.1038/s41598-023-43405-2 |  | Not at all relevant |  |  |  |
| Pro-dopaminergic pharmacological interventions for anhedonia in depression: protocol for a living systematic review of human and non-human studies | 10.12688/wellcomeopenres.19870.1 | Review paper |  |  |  |  |
| Impact of AfterAMI Mobile App on Quality of Life, Depression, Stress and Anxiety in Patients with Coronary Artery Disease: Open Label, Randomized Trial | 10.3390/life13102015 |  | Not at all relevant |  |  |  |
| Improvement of vertigo symptoms after 2 months of Vertigoheel treatment: a case series in patients with bilateral vestibulopathy and functional dizziness | 10.3389/fneur.2023.1264884 |  | Not at all relevant |  |  |  |
| Ecological Momentary Assessment of Midlife Adults' Daily Stress: Protocol for the Stress Reports in Variable Environments (STRIVE) App Study | 10.2196/51845 |  |  | No depression,anxiety,stress |  |  |
| Why Haven't You Texted Me Back? Adolescents' Digital Entrapment, Friendship Conflict, and Perceived General Health | 10.1080/15374416.2023.2261543 |  | Not at all relevant |  |  |  |
| Preferences for enhanced treatment options to address HIV care engagement among women living with HIV and perinatal depression in Malawi | 10.1186/s12889-023-16835-w |  | Not at all relevant |  |  |  |
| The Appa Health App for Youth Mental Health: Development and Usability Study | 10.2196/49998 |  |  | Telehealth app |  |  |
| The Degree of Anxiety and Depression in Patients With Cardiovascular Diseases as Assessed Using a Mobile App: Cross-Sectional Study | 10.2196/48750 |  |  | Screening, no treatment |  |  |
| The Use of Digital Telehealth for the Self-Management of Type 2 Diabetes Patients in Hinds County, Mississippi: A Pilot Study | 10.1177/23743735231188835 |  | Not at all relevant |  |  |  |
| Cultural Responsivity in Technology-Enabled Services: Integrating Culture Into Technology and Service Components | 10.2196/45409 |  | Not at all relevant |  |  |  |
| Testing the Effect of a Smartphone App on Hospital Admissions and Sedentary Behavior in Cardiac Rehabilitation Participants: ToDo-CR Randomized Controlled Trial | 10.2196/48229 |  | Not at all relevant |  |  |  |
| Individualized Virtual Reality for Increasing Self-Compassion: Evaluation Study | 10.2196/47617 |  | Not at all relevant |  |  |  |
| GeriKit: A novel app for comprehensive geriatric assessment | 10.1080/02701960.2022.2048298 |  | Not at all relevant |  |  |  |
| The impact of guided versus supportive coaching on mental health app engagement and clinical outcomes | 10.1177/14604582231215872 |  |  | Telehealth app |  |  |
| Tool to assess risk of bias in studies estimating the prevalence of mental health disorders (RoB-PrevMH) | 10.1136/bmjment-2023-300694 |  | Not at all relevant |  |  |  |
| Findings from the Tushirikiane mobile health (mHealth) HIV self-testing pragmatic trial with refugee adolescents and youth living in informal settlements in Kampala, Uganda | 10.1002/jia2.26185 |  | Not at all relevant |  |  |  |
| Neuronetwork Approach in the Early Diagnosis of Depression |  |  | Not at all relevant |  |  |  |
| Effects of an interactive coaching intervention on quality of life and psychological factors for colorectal cancer survivors: A single group pre and posttest design | 10.1016/j.ejon.2023.102413 |  | Not at all relevant |  |  |  |
| The association between persistent cognitive difficulties and depression and functional outcomes in people with major depressive disorder | 10.1017/S0033291722003671 |  |  | Screening, no treatment |  |  |
| Yourtime: The development and pilot of a perinatal mental wellbeing digital tool using a co-design approach | 10.1016/j.apnr.2023.151714 |  |  | No depression,anxiety,stress |  |  |
| Well-Being Implications of Digital Social Multitasking in Adolescent Friendship: A Latent Profile Analysis | 10.1089/cyber.2023.0049 |  | Not at all relevant |  |  |  |
| N-of-1 Trial in Epilepsy With Fixation-Off Sensitivity: Swimming in the Bathroom | 10.1212/CPJ.0000000000200193 |  | Not at all relevant |  |  |  |
| Reply to: Associations of use of smartphone and internet technologies with loneliness | 10.1111/jgs.18545 |  | Not at all relevant |  |  |  |
| Associations of use of smartphone and internet technologies with loneliness | 10.1111/jgs.18546 |  | Not at all relevant |  |  |  |
| Piloting a prenatal care smartphone application and care navigation intervention at a federally qualified health center | 10.1016/j.ajogmf.2023.101135 |  | Not at all relevant |  |  |  |
| The effectiveness of a thermography-driven preventive foot care protocol on the recurrence of diabetic foot ulcers in low-medical resource settings: An open-labeled randomized controlled trial | 10.1016/j.ijnurstu.2023.104571 |  | Not at all relevant |  |  |  |
| Experience of primary caregivers in utilising an mHealth application for remote dental screening in preschool children | 10.1071/AH23110 |  | Not at all relevant |  |  |  |
| The effectiveness of technology-based cognitive behavioral therapy on perinatal depression and anxiety: A systematic review and meta-analysis | 10.1111/wvn.12673 | Review paper |  |  |  |  |
| Early tactile stimulation influences the development of Alzheimer's disease in gestationally stressed APP (NL-G-F) adult offspring (NL-G-F/NL-G-F) mice | 10.1016/j.expneurol.2023.114498 |  | Not at all relevant |  |  |  |
| The Experience Sampling Method: A New Way of Assessing Variability of the Emotional Dimensions of Religiosity and Spirituality in a Dutch Psychiatric Population | 10.1007/s10943-023-01857-w |  | Not at all relevant |  |  |  |
| Virtually Delivered Psychosocial Intervention for Prenatally Diagnosed Congenital Heart Disease: Feasibility and Acceptability of HEARTPrep | 10.1007/s00246-023-03209-7 |  | Not at all relevant |  |  |  |
| Combining experience sampling with temporal network analysis to understand inertia of negative emotion in dysphoria | 10.1016/j.jad.2023.06.006 |  | Not at all relevant |  |  |  |
| Mobile Mindfulness for Psychological Distress and Burnout among Frontline COVID-19 Nurses: A Pilot Randomized Trial | 10.1513/AnnalsATS.202301-025OC |  | Not at all relevant |  |  |  |
| Imbalance of multiple neurotransmitter pathways leading to depression-like behavior and cognitive dysfunction in the triple transgenic mouse model of Alzheimer disease | 10.1007/s11011-023-01242-2 |  | Not at all relevant |  |  |  |
| Joyful by nature: approaches to investigate the evolution and function of joy in non-human animals | 10.1111/brv.12965 |  | Not at all relevant |  |  |  |
| Predicting real-world emotion and health from spontaneously assessed linguistic distancing using novel scalable technology | 10.1037/emo0001211 |  | Not at all relevant |  |  |  |
| Automated mobile virtual reality cognitive behavior therapy for aviophobia in a natural setting: a randomized controlled trial | 10.1017/S0033291722003531 |  | Not at all relevant |  |  |  |
| College Students' Experiences of Dating App Facilitated Sexual Violence and Associations with Mental Health Symptoms and Well-Being | 10.1080/00224499.2022.2130858 |  | Not at all relevant |  |  |  |
| Social Media Use Early in the Pandemic Predicted Later Social Well-Being and Mental Health in a National Online Sample of Adults in the United States | 10.1080/08964289.2022.2069667 |  |  | Mobile addiction |  |  |
| The effects of bee venom on behavior and the role of leptin in rats | 10.55782/ane-2023-2430 |  | Not at all relevant |  |  |  |
| Effectiveness of a Mobile App Intervention for Preparing Preschool Children and Parents for Day Surgery: Randomized Controlled Trial | 10.2196/46989 |  | Not at all relevant |  |  |  |
| Moderating Effect of eHealth Literacy on the Associations of Coronaphobia With Loneliness, Irritability, Depression, and Stigma in Chinese Young Adults: Bayesian Structural Equation Model Study | 10.2196/47556 |  | Not at all relevant |  |  |  |
| A Suicide Prevention Intervention for Emerging Adult Sexual and Gender Minority Groups: Protocol for a Pilot Hybrid Effectiveness Randomized Controlled Trial | 10.2196/48177 |  | Not at all relevant |  |  |  |
| A Systematic Review of the Long-Term Effects of Using Smartphone- and Tablet-Based Rehabilitation Technology for Balance and Gait Training and Exercise Programs | 10.3390/bioengineering10101142 | Review paper |  |  |  |  |
| Decreased step count prior to the first visit for MDD treatment: a retrospective, observational, longitudinal cohort study of continuously measured walking activity obtained from smartphones | 10.3389/fpubh.2023.1190464 |  | Not at all relevant |  |  |  |
| A practical development protocol for evidence-based digital integrative arts therapy content in public mental health services: digital transformation of mandala art therapy | 10.3389/fpubh.2023.1175093 |  |  | No Mobile app |  |  |
| Improving mental well-being in psychocardiology-a feasibility trial for a non-blended web application as a brief metacognitive-based intervention in cardiovascular disease patients | 10.3389/fpsyt.2023.1138475 |  | Not at all relevant |  |  |  |
| Integrating Neonatal Intensive Care Into a Family Birth Center: Describing the Integrated NICU (I-NIC) | 10.1097/JPN.0000000000000759 |  | Not at all relevant |  |  |  |
| The smartphone as a "significant other": interpersonal dependency and attachment in maladaptive smartphone and social networks use | 10.1186/s40359-023-01339-4 |  | Not at all relevant |  |  |  |
| Mediating and Moderating Effects of Internet Use on Urban-Rural Disparities in Health Among Older Adults: Nationally Representative Cross-Sectional Survey in China | 10.2196/45343 |  | Not at all relevant |  |  |  |
| Experiences of Using Digital Mindfulness-Based Interventions: Rapid Scoping Review and Thematic Synthesis | 10.2196/44220 | Review paper |  |  |  |  |
| Engagement, Satisfaction, and Mental Health Outcomes Across Different Residential Subgroup Users of a Digital Mental Health Relational Agent: Exploratory Single-Arm Study | 10.2196/46473 |  | Not at all relevant |  |  |  |
| Efficacy, Safety, and Evaluation Criteria of mHealth Interventions for Depression: Systematic Review | 10.2196/46877 | Review paper |  |  |  |  |
| Mindfulness-Based Mobile Health to Address Unhealthy Eating Among Middle-Aged Sexual Minority Women With Early Life Adversity: Mixed Methods Feasibility Trial | 10.2196/46310 |  | Not at all relevant |  |  |  |
| Self-help application for obsessive-compulsive disorder based on exposure and response prevention technique with prototype design and usability evaluation: A cross-sectional study | 10.1002/hsr2.1577 |  | Not at all relevant |  |  |  |
| Rolipram Ameliorates Memory Deficits and Depression-Like Behavior in APP/PS1/tau Triple Transgenic Mice: Involvement of Neuroinflammation and Apoptosis via cAMP Signaling | 10.1093/ijnp/pyad042 |  | Not at all relevant |  |  |  |
| The effect of mobile-based logotherapy on depression, suicidal ideation, and hopelessness in patients with major depressive disorder: a mixed-methods study | 10.1038/s41598-023-43051-8 |  |  | No Mobile app |  |  |
| Efficacy of a Text-Based Mental Health Coaching App in Improving the Symptoms of Stress, Anxiety, and Depression: Randomized Controlled Trial | 10.2196/46458 |  |  | Telehealth app |  |  |
| Specifying the Efficacy of Digital Therapeutic Tools for Depression and Anxiety: Retrospective, 2-Cohort, Real-World Analysis | 10.2196/47350 |  | Not at all relevant |  |  |  |
| Use of mobile technology to identify behavioral mechanisms linked to mental health outcomes in Kenya: protocol for development and validation of a predictive model | 10.1186/s13104-023-06498-6 |  | Not at all relevant |  |  |  |
| Developing Suicide Prevention Tools in the Context of Digital Peer Support: Qualitative Analysis of a Workshop With Multidisciplinary Stakeholders | 10.2196/47178 |  | Not at all relevant |  |  |  |
| Feeling anxious'- women's experiences of having a baby in Australia during the COVID-19 pandemic using the Voqual real time app | 10.1186/s12884-023-05993-9 |  | Not at all relevant |  |  |  |
| The Use of Mobile Assessments for Monitoring Mental Health in Youth: Umbrella Review | 10.2196/45540 | Review paper |  |  |  |  |
| Guided Internet-Based Cognitive Behavioral Therapy for Women With Bulimia Nervosa: Protocol for a Multicenter Randomized Controlled Trial | 10.2196/49828 |  | Not at all relevant |  |  |  |
| Nonpharmacological Behavior Guidance for the Pediatric Dental Patient |  |  | Not at all relevant |  |  |  |
| The healthy moms and babies app to prevent postpartum depression: analysis of user-profiles and dropout cases | 10.3389/fpubh.2023.1186963 |  |  | App usability |  |  |
| Timed intercourse for couples trying to conceive | 10.1002/14651858.CD011345.pub3 |  | Not at all relevant |  |  |  |
| Potential Mobile Health Applications for Improving the Mental Health of the Elderly: A Systematic Review | 10.2147/CIA.S410396 | Review paper |  |  |  |  |
| Internet-Based Cognitive Behavioral Therapy and Virtual Reality Exposure Therapy for Social Anxiety Disorder: Protocol for a Randomized Controlled Trial in Hong Kong | 10.2196/48437 |  |  | No Mobile app |  |  |
| The mechanism and effectiveness of mindfulness-based intervention for reducing the psychological distress of parents of children with autism spectrum disorder: A protocol of randomized control trial of ecological momentary intervention and assessment | 10.1371/journal.pone.0291168 |  |  | Telehealth app |  |  |
| Cardiff Online Cognitive Assessment in a National Sample: Cross-Sectional Web-Based Study | 10.2196/46675 |  | Not at all relevant |  |  |  |
| Community engagement programs on radiation and health: addressing public concerns | 10.17061/phrpp3332325 |  | Not at all relevant |  |  |  |
| CBT program to reduce recidivism risk for road crashes among adolescents and young adults: Results of a randomized controlled study and prospects | 10.1016/j.heliyon.2023.e20074 |  | Not at all relevant |  |  |  |
| StudiCare procrastination - Randomized controlled non-inferiority trial of a persuasive design-optimized internet- and mobile-based intervention with digital coach targeting procrastination in college students | 10.1186/s40359-023-01312-1 |  | Not at all relevant |  |  |  |
| A Digital System (YouXin) to Facilitate Self-Management by People With Psychosis in China: Protocol for a Nonrandomized Validity and Feasibility Study With a Mixed Methods Design | 10.2196/45170 |  |  | App usability |  |  |
| Chemotherapy-related symptoms and exercise adherence in older patients with myeloid neoplasms | 10.1007/s00520-023-08039-0 |  | Not at all relevant |  |  |  |
| Cardiac rehabilitation engagement and associated factors among heart failure patients: a cross-sectional study | 10.1186/s12872-023-03470-x |  | Not at all relevant |  |  |  |
| Efficacy of a Cognitive Behavioral Intervention for the Prevention of Depression in Nonprofessional Caregivers Administered through a Smartphone App: A Randomized Controlled Trial | 10.3390/jcm12185872 |  |  | Telehealth app |  |  |
| Exploring Sociodemographic Characteristics, Adverse Childhood Experience, and Mental Health History as Predictors of Anxiety and Depression among Adolescents and Young Adults: Findings from the MoreGoodDays Support Program in Alberta, Canada | 10.3390/bs13090749 |  | Not at all relevant |  |  |  |
| Geometric Wire-frame Fixation of Skin Grafting for Lip Reconstruction | 10.1097/GOX.0000000000005248 |  | Not at all relevant |  |  |  |
| Evaluating the feasibility and exploring the efficacy of an emotion-based approach-avoidance modification training (eAAMT) in the context of perceived stress in an adult sample - protocol of a parallel randomized controlled pilot study | 10.1186/s40814-023-01386-z |  | Not at all relevant |  |  |  |
| Randomized controlled trial of a smartphone-based cognitive behavioral therapy for chronic tinnitus | 10.1371/journal.pdig.0000337 |  | Not at all relevant |  |  |  |
| Beneficial effects of physical exercise and an orally active mGluR2/3 antagonist pro-drug on neurogenesis and behavior in an Alzheimer's amyloidosis model | 10.3389/frdem.2023.1198006 |  | Not at all relevant |  |  |  |
| The Use of Patient-Oriented Mobile Phone Apps in Oral Health: Scoping Review | 10.2196/46143 | Review paper |  |  |  |  |
| Changes in Intrapersonal Factors of Participants in the Pregnancy Remote Monitoring Study Who Are at Risk for Pregnancy-Induced Hypertension: Descriptive Quantitative Study | 10.2196/42686 |  | Not at all relevant |  |  |  |
| Mobile Health Intervention in Patients With Type 2 Diabetes: A Randomized Clinical Trial | 10.1001/jamanetworkopen.2023.33629 |  | Not at all relevant |  |  |  |
| Community Mobility and Depressive Symptoms During the COVID-19 Pandemic in the United States | 10.1001/jamanetworkopen.2023.34945 |  | Not at all relevant |  |  |  |
| Detecting Social Contexts from Mobile Sensing Indicators in Virtual Interactions with Socially Anxious Individuals | 10.1145/3610916 |  | Not at all relevant |  |  |  |
| A Randomized Clinical Trial of Technology-Enhanced Family-Focused Therapy for Youth in the Early Stages of Mood Disorders | 10.1016/j.jaacop.2023.04.002 |  |  |  | Telehealth |  |
| A study on prevalence and risk factors of depression among adolescent girls studying in government and private schools-A comparative study | 10.4103/jfmpc.jfmpc_402_23 |  | Not at all relevant |  |  |  |
| A randomized controlled trial on the effect of smartphone-based mental health application among outpatients with depressive and anxiety symptoms: A pilot study in Malaysia | 10.4103/indianjpsychiatry.indianjpsychiatry_240_23 |  |  |  |  | 1 |
| A Study of the Effectiveness of Mobile Health Application in A Self-management Intervention for Kidney Transplant Patients |  |  | Not at all relevant |  |  |  |
| The Effect of Running Water Sound Listened to Patients During Urodynamics on Anxiety and Urodynamic Parameters | 10.5213/inj.2346116.058 |  | Not at all relevant |  |  |  |
| Problematic Smartphone Use and Its Relationship With Anxiety and Suicidal Ideation Among South Korean Adolescents | 10.30773/pi.2023.0051 |  |  | Mobile addiction |  |  |
| Potential Role of Smartphone Technology in Advancing Work on Neurological Soft Signs with a Focus on Schizophrenia | 10.1097/HRP.0000000000000377 |  | Not at all relevant |  |  |  |
| Experiences of participating in an eHealth intervention for patients with abdominal aortic aneurysm: A qualitative study | 10.1016/j.jvn.2023.05.007 |  | Not at all relevant |  |  |  |
| Validation of Photoplethysmography Using a Mobile Phone Application for the Assessment of Heart Rate Variability in the Context of Heart Rate Variability-Biofeedback | 10.1097/PSY.0000000000001236 |  | Not at all relevant |  |  |  |
| Changes to healthcare utilisation and symptoms for common mental health problems over the first 21 months of the COVID-19 pandemic: parallel analyses of electronic health records and survey data in England | 10.1016/j.lanepe.2023.100697 |  | Not at all relevant |  |  |  |
| One-Week Suicide Risk Prediction Using Real-Time Smartphone Monitoring: Prospective Cohort Study | 10.2196/43719 |  | Not at all relevant |  |  |  |
| Utility of Smartphone-Based Digital Phenotyping Biomarkers in Assessing Treatment Response to Transcranial Magnetic Stimulation in Depression: Proof-of-Concept Study | 10.2196/40197 |  | Not at all relevant |  |  |  |
| Changes in stress, burnout, and resilience associated with an 8-week intervention with relational agent "Woebot" | 10.1016/j.invent.2023.100637 |  |  | Telehealth app |  |  |
| Automatic patient functionality assessment from multimodal data using deep learning techniques - Development and feasibility evaluation | 10.1016/j.invent.2023.100657 |  | Not at all relevant |  |  |  |
| Reactivity toward daily events: Intraindividual variability and change in recurrent depression - A measurement burst study | 10.1016/j.brat.2023.104383 |  | Not at all relevant |  |  |  |
| Feasibility and efficacy of a digital resilience training: A pilot study of the strengths-based training RESIST | 10.1016/j.invent.2023.100649 |  | Not at all relevant |  |  |  |
| A flexible micro-randomized trial design and sample size considerations | 10.1177/09622802231188513 |  | Not at all relevant |  |  |  |
| Smartphone Photoplethysmography Pulse Rate Covaries With Stress and Anxiety During a Digital Acute Social Stressor | 10.1097/PSY.0000000000001178 |  | Not at all relevant |  |  |  |
| Association between immigrant concentration and mental health service utilization in the United States over time: A geospatial big data analysis | 10.1016/j.healthplace.2023.103055 |  | Not at all relevant |  |  |  |
| Trait and state effects of different modes of thinking on salivary cortisol in daily life in patients with recurrent major depression and healthy individuals | 10.1016/j.psyneuen.2023.106307 |  | Not at all relevant |  |  |  |
| Investigation of the protective effect of long-term exercise on molecular pathways and behaviours in scopolamine induced alzheimer's disease-like condition | 10.1016/j.brainres.2023.148429 |  | Not at all relevant |  |  |  |
| Systematic review: Effect of mobile applications on college students with depressive symptoms | 10.1111/jan.15716 | Review paper |  |  |  |  |
| Intensive Longitudinal Assessment of Adolescents to Predict Suicidal Thoughts and Behaviors | 10.1016/j.jaac.2023.03.018 |  | Not at all relevant |  |  |  |
| Dietary Self-Management Using Mobile Health Technology for Adults With Type 2 Diabetes: A Scoping Review | 10.1177/19322968231174038 | Review paper |  |  |  |  |
| Emotional Dynamics in Fibromyalgia: Pain, Fatigue, and Stress Moderate Momentary Associations Between Positive and Negative Emotions | 10.1016/j.jpain.2023.04.007 |  |  | Screening, no treatment |  |  |
| Predictors of physical activity behavior change among patients with heart failure enrolled in home-based cardiac rehabilitation intervention | 10.1016/j.hrtlng.2023.04.003 |  | Not at all relevant |  |  |  |
| Meeting Youth Where They Live: How to Use a QR Code in Safety Planning | 10.1016/j.jaac.2023.01.025 |  | Not at all relevant |  |  |  |
| Adoption of Best Practices in Behavioral Health Crisis Care by Mental Health Treatment Facilities | 10.1176/appi.ps.20220427 |  |  | No Mobile app |  |  |
| The Relationship Between Depressive Symptoms, eHealth Literacy, and Asthma Outcomes in the Context of a Mobile Health Intervention | 10.1097/PSY.0000000000001170 |  | Not at all relevant |  |  |  |
| Clinically Significant Scores for Thwarted Belonging and Perceived Burden from the Interpersonal Needs Questionnaire (INQ-15) | 10.1027/0227-5910/a000898 |  | Not at all relevant |  |  |  |
| Mind the Mood: Momentary Depression and Anxiety Moderate the Correspondence Between Subjective and Objective Cognitive Functioning in Fibromyalgia | 10.1002/acr.25086 |  |  | Screening, no treatment |  |  |
| The Future Proofing Study: Design, methods and baseline characteristics of a prospective cohort study of the mental health of Australian adolescents | 10.1002/mpr.1954 |  | Not at all relevant |  |  |  |
| Prediction of impending mood episode recurrence using real-time digital phenotypes in major depression and bipolar disorders in South Korea: a prospective nationwide cohort study | 10.1017/S0033291722002847 |  | Not at all relevant |  |  |  |
| Introducing the Fear Learning and Anxiety Response (FLARe) app and web portal for the remote delivery of fear conditioning experiments | 10.3758/s13428-022-01952-y |  | Not at all relevant |  |  |  |
| Expenditure on Paid-for Gambling Advertising During the National COVID-19 'Lockdowns': An Observational Study of Media Monitoring Data from the United Kingdom | 10.1007/s10899-022-10153-3 |  | Not at all relevant |  |  |  |
| Culturally Adapting an Internet-Delivered Mindfulness Intervention for Indonesian University Students Experiencing Psychological Distress: Mixed Methods Study | 10.2196/47126 |  | Not at all relevant |  |  |  |
| Stress Management Apps: Systematic Search and Multidimensional Assessment of Quality and Characteristics | 10.2196/42415 | Review paper |  |  |  |  |
| Acceptability and Utility of a Smartphone App to Support Adolescent Mental Health (BeMe): Program Evaluation Study | 10.2196/47183 |  |  | Telehealth app |  |  |
| Feasibility of a smartphone app for prescribed exercise tutoring in patients with stable coronary heart disease | 10.1177/20552076231197424 |  | Not at all relevant |  |  |  |
| Technology-assisted cognitive-behavior therapy delivered by peers versus standard cognitive behavior therapy delivered by community health workers for perinatal depression: study protocol of a cluster randomized controlled non-inferiority trial | 10.1186/s13063-023-07581-w |  | Not at all relevant |  |  |  |
| Investigating two mobile just-in-time adaptive interventions to foster psychological resilience: research protocol of the DynaM-INT study | 10.1186/s40359-023-01249-5 |  | Not at all relevant |  |  |  |
| Digital Health Tools Can Support Patient Access to Culturally and Linguistically Competent Behavioral Health Treatment | 10.2196/51799 |  | Not at all relevant |  |  |  |
| Efficacy of a Brief Blended Cognitive Behavioral Therapy Program for the Treatment of Depression and Anxiety in University Students: Uncontrolled Intervention Study | 10.2196/44742 |  |  | Telehealth app |  |  |
| Novel Technologies in Breast Imaging: A Scoping Review | 10.7759/cureus.44061 | Review paper |  |  |  |  |
| Psychosocial interventions for the prevention of self-harm repetition: protocol for a systematic review and network meta-analysis | 10.1136/bmjopen-2023-072289 | Review paper |  |  |  |  |
| Selective Prevention of Depression in Workers Using a Smartphone App: Randomized Controlled Trial | 10.2196/45963 |  |  |  |  | 1 |
| Mobile Technologies for Supporting Mental Health in Youths: Scoping Review of Effectiveness, Limitations, and Inclusivity | 10.2196/46949 | Review paper |  |  |  |  |
| A Nurse-Led Care Delivery App and Telehealth System for Patients Requiring Wound Care: Mixed Methods Implementation and Evaluation Study | 10.2196/43258 |  | Not at all relevant |  |  |  |
| The Relationship of Depression Level and Physical Activity with Postural Control in Geriatric Individuals | 10.29399/npa.28217 |  | Not at all relevant |  |  |  |
| Emodiversity evaluation of remote workers through health monitoring based on intra-day emotion sampling | 10.3389/fpubh.2023.1196539 |  | Not at all relevant |  |  |  |
| Defining and Phenotyping Gastric Abnormalities in Long-Term Type 1 Diabetes Using a Novel Body Surface Gastric Mapping Device | 10.1016/j.gastha.2023.08.005 |  | Not at all relevant |  |  |  |
| Genetically predisposed and resilient animal models of depression reveal divergent responses to early-life adversity | 10.1017/neu.2023.37 |  | Not at all relevant |  |  |  |
| Telerehabilitation with ARC Intellicare to Cope with Motor and Respiratory Disabilities: Results about the Process, Usability, and Clinical Effect of the "Ricominciare" Pilot Study | 10.3390/s23167238 |  | Not at all relevant |  |  |  |
| A Novel Virtual-Based Comprehensive Clinical Approach to Headache Care | 10.3390/jcm12165349 |  | Not at all relevant |  |  |  |
| Suicidal behavior in persons attended in out-of-hospital emergency services in Spain | 10.3389/fpsyt.2023.1235583 |  | Not at all relevant |  |  |  |
| Effects of Community-Based Caring Contact in Reducing Thwarted Belongingness Among Postdischarge Young Adults With Self-Harm: Randomized Controlled Trial | 10.2196/43526 |  | Not at all relevant |  |  |  |
| The effects of ketamine on symptoms of depression and anxiety in real-world care settings: A retrospective controlled analysis | 10.1016/j.jad.2023.04.141 |  | Not at all relevant |  |  |  |
| Antenatal mobile-delivered mindfulness-based intervention to reduce perinatal depression risk and improve obstetric and neonatal outcomes: A randomized controlled trial | 10.1016/j.jad.2023.04.133 |  |  | No Mobile app |  |  |
| Efficacy and associated neurotransmitters of digital cognitive behavior therapy for atopic dermatitis: A comparative effectiveness research | 10.12932/AP-100223-1542 |  | Not at all relevant |  |  |  |
| Efficacy of an Internet-based intervention with self-applied exposure therapy in virtual reality for people with panic disorder: study protocol for a randomized controlled trial | 10.1186/s13063-023-07536-1 |  | Not at all relevant |  |  |  |
| Assessing Patient Adherence to and Engagement With Digital Interventions for Depression in Clinical Trials: Systematic Literature Review | 10.2196/43727 | Review paper |  |  |  |  |
| Evaluation of the mental health status of community frontline medical workers after the normalized management of COVID-19 in Sichuan, China | 10.3389/fpsyt.2023.1198822 |  | Not at all relevant |  |  |  |
| Maternal mental health after infant discharge: a quasi-experimental clinical trial of family integrated care versus family-centered care for preterm infants in U.S. NICUs | 10.1186/s12887-023-04211-x |  | Not at all relevant |  |  |  |
| Exploring facilitators and barriers of the sustainable acceptance of e-health system solutions in Ethiopia: A systematic review | 10.1371/journal.pone.0287991 | Review paper |  |  |  |  |
| A Fast and Minimal System to Identify Depression Using Smartphones: Explainable Machine Learning-Based Approach | 10.2196/28848 |  |  | Screening, no treatment |  |  |
| Tailoring a Text Messaging and Fotonovela Program to Increase Patient Engagement in Colorectal Cancer Screening in a Large Urban Community Clinic Population: Quality Improvement Project | 10.2196/43024 |  | Not at all relevant |  |  |  |
| The protocol of DECO-MOM: a clinical trial testing the effectiveness of a mobile application for an environmental health intervention among pregnant women | 10.1186/s12911-023-02258-5 |  | Not at all relevant |  |  |  |
| Impact of web application support versus standard management on adherence with adjuvant hormone therapy in patients treated for breast cancer: the WEBAPPAC study | 10.1186/s12885-023-11242-1 |  | Not at all relevant |  |  |  |
| Mental health of nursing professionals: Internet-based interventions | 10.47626/1679-4435-2022-801 |  | Not at all relevant |  |  |  |
| A protocol for randomized controlled trial on multidisciplinary interventions for mobility limitation in the older adults (M-MobiLE) | 10.1186/s12877-023-04117-4 |  | Not at all relevant |  |  |  |
| Usability and Satisfaction Testing of Game-Based Learning Avatar-Navigated Mobile (GLAm), an App for Cervical Cancer Screening: Mixed Methods Study | 10.2196/45541 |  | Not at all relevant |  |  |  |
| Opportunities for the Implementation of a Digital Mental Health Assessment Tool in the United Kingdom: Exploratory Survey Study | 10.2196/43271 |  | Not at all relevant |  |  |  |
| Assessing Mood With the Identifying Depression Early in Adolescence Chatbot (IDEABot): Development and Implementation Study | 10.2196/44388 |  |  | No Mobile app |  |  |
| Is asynchronous telerehabilitation equal to synchronous telerehabilitation in COVID-19 survivors with classes 4-6? | 10.1177/1357633X231189761 |  | Not at all relevant |  |  |  |
| A smartphone-based colorimetric assay using Au@Ag core-shell nanoparticles as the nanoprobes for visual tracing of fluvoxamine in biofluids as a common suicide drug | 10.1016/j.saa.2023.122665 |  | Not at all relevant |  |  |  |
| Patterns of smartphone usage associated with depressive symptoms in nursing students | 10.3389/fpsyt.2023.1136126 |  |  | Mobile addiction |  |  |
| Adherence and the Diabetic Foot: High Tech Meets High Touch? | 10.3390/s23156898 |  | Not at all relevant |  |  |  |
| The Influence of Greenspace Exposure on Affect in People With and Those Without Schizophrenia: Exploratory Study | 10.2196/44323 |  | Not at all relevant |  |  |  |
| Outcome's Classification in Mobile Applications Tailored to Parents of Premature Infants: A Systematic Review | 10.18502/ijph.v52i8.13402 | Review paper |  |  |  |  |
| Anxiety, patient activation, and quality of life among stroke survivors prescribed smartwatches for atrial fibrillation monitoring | 10.1016/j.cvdhj.2023.04.002 |  | Not at all relevant |  |  |  |
| Feasibility and impact of a mental health chatbot on postpartum mental health: a randomized controlled trial | 10.1016/j.xagr.2023.100165 |  |  | No Mobile app |  |  |
| Brief imaginal exposure exercises for social anxiety disorder: A randomized controlled trial of a self-help momentary intervention app | 10.1016/j.janxdis.2023.102749 |  |  |  |  | 1 |
| Measures of Connectivity and Dorsolateral Prefrontal Cortex Volumes and Depressive Symptoms Following Treatment With Selective Serotonin Reuptake Inhibitors in Adolescents | 10.1001/jamanetworkopen.2023.27331 |  | Not at all relevant |  |  |  |
| Micro urban spaces and mental well-being: Measuring the exposure to urban landscapes along daily mobility paths and their effects on momentary depressive symptomatology among older population | 10.1016/j.envint.2023.108095 |  | Not at all relevant |  |  |  |
| A Mobile Application Adjunct to Augment Cognitive-Behavioral Group Therapy for Adolescents with Social Anxiety: Feasibility and Acceptability Results from the Wiring Adolescents with Social Anxiety via Behavioral Interventions Pilot Trial | 10.1089/cap.2023.0001 |  |  | Telehealth app |  |  |
| Comparison of Ambulatory Quality Measures Between Shared Practice Panels and Independent Practice Panels | 10.1016/j.mayocpiqo.2023.05.005 |  | Not at all relevant |  |  |  |
| Spinal cord injury-activated C/EBPβ-AEP axis mediates cognitive impairment through APP C586/Tau N368 fragments spreading | 10.1016/j.pneurobio.2023.102467 |  | Not at all relevant |  |  |  |
| Investigation on risk factors of chronic diseases among community residents: A study based on health management systems supported by Mobile phones | 10.1002/nop2.1747 |  | Not at all relevant |  |  |  |
| Network analysis of ecological momentary assessment identifies frustration as a central node in irritability | 10.1111/jcpp.13794 |  | Not at all relevant |  |  |  |
| Real-time monitoring of increases in restlessness to assess idiographic risk of recurrence of depressive symptoms | 10.1017/S0033291722002069 |  | Not at all relevant |  |  |  |
| [Mobile child psychiatric team in French Guiana: Analyzing Amerindian teenager issues] | 10.1016/j.encep.2022.01.010 |  | Not at all relevant |  |  |  |
| Language skills and interpersonal trust in adolescents with and without mental illness | 10.1080/17549507.2022.2075466 |  | Not at all relevant |  |  |  |
| Dispositional and performance-specific music performance anxiety in young amateur musicians | 10.3389/fpsyg.2023.1208311 |  | Not at all relevant |  |  |  |
| Immediate Effects of Mobile Phone App for Depressed Mood in Young Adults with Subthreshold Depression: A Pilot Randomized Controlled Trial | 10.2147/NDT.S415937 |  |  |  |  | 1 |
| Smartphone-Based Video Antenatal Preterm Birth Education: The Preemie Prep for Parents Randomized Clinical Trial | 10.1001/jamapediatrics.2023.1586 |  | Not at all relevant |  |  |  |
| Effect of the INTER-ACT lifestyle intervention on maternal mental health during the first year after childbirth: A randomized controlled trial | 10.1371/journal.pone.0284770 |  | Not at all relevant |  |  |  |
| After 3 months of medication balloon therapy, a patient who had contrast-induced encephalopathy recovered: A case report | 10.1097/MD.0000000000034392 |  | Not at all relevant |  |  |  |
| Perspectives on emotional memory images and the persistence of pain | 10.3389/fpain.2023.1217721 |  | Not at all relevant |  |  |  |
| Nomophobia among university students in five Arab countries in the Middle East: prevalence and risk factors | 10.1186/s12888-023-05049-4 |  | Not at all relevant |  |  |  |
| Novel method for predicting nonvisible symptoms using machine learning in cancer palliative care | 10.1038/s41598-023-39119-0 |  | Not at all relevant |  |  |  |
| User-Centered Design of a Digitally Enabled Care Pathway in a Large Health System: Qualitative Interview Study | 10.2196/42768 |  | Not at all relevant |  |  |  |
| Effectiveness of Daily Mindfulness Meditation App Usage to Reduce Anxiety and Improve Well-Being During the COVID-19 Pandemic: A Randomized Controlled Trial | 10.7759/cureus.42432 |  |  |  |  |  |
| The effect of hybrid SCMC (BYOD) on foreign language anxiety and learning experience in comparison to pure SCMC and FTF communication | 10.3389/fpsyg.2023.1172442 |  | Not at all relevant |  |  |  |
| Mindfulness Applications: Can They Serve as a Stress, Anxiety, and Burnout Reduction Tool in Orthopaedic Surgery Training? A Randomized Control Trial | 10.2106/JBJS.OA.22.00114 |  |  |  |  | 1 |
| Efficacy of smartphone-based virtual reality relaxation in providing comfort to patients with cancer undergoing chemotherapy in oncology outpatient setting in Indonesia: protocol for a randomised controlled trial | 10.1136/bmjopen-2023-074506 |  | Not at all relevant |  |  |  |
| Evidence for a clathrin-independent endocytic pathway for APP internalization in the neuronal somatodendritic compartment | 10.1016/j.celrep.2023.112774 |  | Not at all relevant |  |  |  |
| Assessment of Different Distraction Behavioral Methods in Pediatric Dental Clinic: A Systematic Review | 10.7759/cureus.42366 | Review paper |  |  |  |  |
| Complex interactive multimodal intervention to improve personalized stress management among healthcare workers in China: A knowledge translation protocol | 10.1177/20552076231184052 |  | Not at all relevant |  |  |  |
| Using Intervention Mapping and Behavior Change Techniques to Develop a Digital Intervention for Self-Management in Stroke: Development Study | 10.2196/45099 |  | Not at all relevant |  |  |  |
| The Impact of a Gamified Mobile Mental Health App (eQuoo) on Resilience and Mental Health in a Student Population: Large-Scale Randomized Controlled Trial | 10.2196/47285 |  |  |  |  | 1 |
| A randomized 3-month, parallel-group, controlled trial of CALMA m-health app as an adjunct to therapy to reduce suicidal and non-suicidal self-injurious behaviors in adolescents: study protocol | 10.3389/fpsyt.2023.1087097 |  | Not at all relevant |  |  |  |
| Effects of Social Anxiety and Subjective Well-Being on Problematic Mobile Social Media Use in First-Year University Students: The Mediating Role of Self-Esteem | 10.1177/00332941231190326 |  | Not at all relevant |  |  |  |
| The Potential Usefulness of ChatGPT in Oral and Maxillofacial Radiology | 10.7759/cureus.42133 |  | Not at all relevant |  |  |  |
| Liver function test indices-based prediction model for post-stroke depression: a multicenter, retrospective study | 10.1186/s12911-023-02241-0 |  | Not at all relevant |  |  |  |
| Attitudes Toward Seeking Mental Health Services and Mobile Technology to Support the Management of Depression Among Black American Women: Cross-Sectional Survey Study | 10.2196/45766 |  |  | Screening, no treatment |  |  |
| Scope, Characteristics, Behavior Change Techniques, and Quality of Conversational Agents for Mental Health and Well-Being: Systematic Assessment of Apps | 10.2196/45984 | Review paper |  |  |  |  |
| The Geras virtual frailty rehabilitation program to build resilience in older adults with frailty during COVID-19: a randomized feasibility trial | 10.1186/s40814-023-01346-7 |  | Not at all relevant |  |  |  |
| The Effect of Loneliness on Nomophobia: A Moderated Mediation Model | 10.3390/bs13070595 |  | Not at all relevant |  |  |  |
| Association of Depressive Symptom Trajectory With Physical Activity Collected by mHealth Devices in the Electronic Framingham Heart Study: Cohort Study | 10.2196/44529 |  | Not at all relevant |  |  |  |
| Examining Predictors of Depression and Anxiety Symptom Change in Cognitive Behavioral Immersion: Observational Study | 10.2196/42377 |  | Not at all relevant |  |  |  |
| The psychological impact of COVID-19 pandemic and associated factors among college and university students in Ethiopia: a systematic review and meta-analysis, 2022 | 10.3389/fpubh.2023.1136031 | Review paper |  |  |  |  |
| Long-Term Efficacy and Tolerability of a Medium G' HA Filler with Tri-Hyal Technology on the Rejuvenation of the Mobile Facial Zone | 10.2147/CCID.S395353 |  | Not at all relevant |  |  |  |
| Smartphone use and well-being in Pakistan: Comparing the effect of self-reported and actual smartphone use | 10.1177/20552076231186075 |  | Not at all relevant |  |  |  |
| A self-guided Internet-based intervention for individuals with chronic pain and depressive symptoms: study protocol of a randomized controlled trial | 10.1186/s13063-023-07440-8 |  |  | No Mobile app |  |  |
| Correction: mHealth app using machine learning to increase physical activity in diabetes and depression: clinical trial protocol for the DIAMANTE Study | 10.1136/bmjopen-2019-034723corr1 |  | Not at all relevant |  |  |  |
| Testing Mechanisms of Change for Text Message-Delivered Cognitive Behavioral Therapy: Randomized Clinical Trial for Young Adult Depression | 10.2196/45186 |  | Not at all relevant |  |  |  |
| Effectiveness of an Integrated Mobile Application for Lifestyle Modifications in Overweight Women with Polycystic Ovarian Syndrome: A Randomized Controlled Trial | 10.3390/life13071533 |  | Not at all relevant |  |  |  |
| Sex-Specific Effects of Anxiety on Cognition and Activity-Dependent Neural Networks: Insights from (Female) Mice and (Wo)Men | 10.1101/2023.07.07.548180 |  | Not at all relevant |  |  |  |
| Rumination as a Transdiagnostic Phenomenon in the 21st Century: The Flow Model of Rumination | 10.3390/brainsci13071041 |  | Not at all relevant |  |  |  |
| An exploratory analysis of the effect size of the mobile mental health Application, mindLAMP | 10.1177/20552076231187244 |  |  |  |  | 1 |
| Benefits and Harms of Digital Health Interventions Promoting Physical Activity in People With Chronic Conditions: Systematic Review and Meta-Analysis | 10.2196/46439 | Review paper |  |  |  |  |
| The Use of Virtual Reality Interventions to Promote Positive Mental Health: Systematic Literature Review | 10.2196/44998 | Review paper |  |  |  |  |
| Molehill Mountain feasibility study: Protocol for a non-randomised pilot trial of a novel app-based anxiety intervention for autistic people | 10.1371/journal.pone.0286792 |  | Not at all relevant |  |  |  |
| Psychiatric Treatment Conducted via Telemedicine Versus In-Person Modality in Posttraumatic Stress Disorder, Mood Disorders, and Anxiety Disorders: Systematic Review and Meta-Analysis | 10.2196/44790 | Review paper |  |  |  |  |
| Dynamic Modelling of Mental Resilience in Young Adults: Protocol for a Longitudinal Observational Study (DynaM-OBS) | 10.2196/39817 |  | Not at all relevant |  |  |  |
| Young adults' adoption and use of mental health apps: efficient, effective, but no replacement for in-person care | 10.1080/07448481.2023.2227727 |  | Not at all relevant |  |  |  |
| Designing High-Fidelity Mobile Health for Depression in Indonesian Adolescents Using Design Science Research: Mixed Method Approaches | 10.2196/48913 |  | Not at all relevant |  |  |  |
| Digital therapeutic to improve cancer-related well-being: a pilot randomized controlled trial | 10.1136/ijgc-2023-004304 |  | Not at all relevant |  |  |  |
| Mobile Communication Log Time Series to Detect Depressive Symptoms | 10.1109/EMBC40787.2023.10341154 |  | Not at all relevant |  |  |  |
| Depressive Disorder Remote Detection through Touchscreen Typing Behaviour | 10.1109/EMBC40787.2023.10340393 |  | Not at all relevant |  |  |  |
| The Impact of the FertiStrong Mobile Application on Anxiety and Depression in Men: A Randomised Control Pilot Study | 10.4103/jhrs.jhrs_75_23 |  |  |  |  | 1 |
| What's the optimal duration of anticoagulation in patients with left ventricular thrombus? | 10.1080/14779072.2023.2270906 |  | Not at all relevant |  |  |  |
| Intolerance of uncertainty fuels preservice teachers' smartphone dependence through rumination and anxiety during the COVID-19 pandemic: A cross-sectional study | 10.1016/j.heliyon.2023.e17798 |  | Not at all relevant |  |  |  |
| Digital technologies to support adolescents with depression and anxiety: review | 10.1192/bja.2022.3 | Review paper |  |  |  |  |
| Survey of Postpartum People in the United States During the First Wave of the COVID-19 Pandemic to Explore Their Perspective on Support After Discharge | 10.1097/JPN.0000000000000747 |  | Not at all relevant |  |  |  |
| A case study of virtually delivered emotion-focused family therapy | 10.1111/jmft.12648 |  | Not at all relevant |  |  |  |
| Urate-lowering therapy adherence and the association with medication beliefs, self-efficacy, depression, anxiety, and COVID-19 pandemic-related concern in Chinese gout patients: a cross-sectional study | 10.1080/13548506.2023.2224039 |  | Not at all relevant |  |  |  |
| Reduced Compared With Traditional Schedules for Routine Antenatal Visits: A Systematic Review | 10.1097/AOG.0000000000005193 | Review paper |  |  |  |  |
| Analyzing text message linguistic features: Do people with depression communicate differently with their close and non-close contacts? | 10.1016/j.brat.2023.104342 |  | Not at all relevant |  |  |  |
| Dreissena polymorpha responses under thermal and hypoxic stress: New insights in the tolerance of this freshwater sentinel species | 10.1016/j.aquatox.2023.106586 |  | Not at all relevant |  |  |  |
| Evaluation of mobile applications focused on the care of patients with anxiety disorders: A systematic review in app stores in Brazil | 10.1016/j.ijmedinf.2023.105087 | Review paper |  |  |  |  |
| Characteristics associated with poor atrial fibrillation-related quality of life in adults with atrial fibrillation | 10.2459/JCM.0000000000001479 |  | Not at all relevant |  |  |  |
| Anticipated suicidal and death ideation in response to an imagined dementia diagnosis: A qualitative study | 10.1177/14713012231171490 |  | Not at all relevant |  |  |  |
| Less inhibited and more depressed? The puzzling association between mood, inhibitory control and depressive symptoms | 10.1016/j.comppsych.2023.152386 |  | Not at all relevant |  |  |  |
| Structured group education programme and accompanying mHealth intervention to promote physical activity in women with a history of gestational diabetes: A randomised controlled trial | 10.1111/dme.15118 |  | Not at all relevant |  |  |  |
| A Qualitative Investigation of Preparedness for the Launch of 988: Implications for the Continuum of Emergency Mental Health Care | 10.1007/s10488-023-01263-0 |  | Not at all relevant |  |  |  |
| Moderate dietary salt restriction improves blood pressure and mental well-being in patients with primary aldosteronism: The salt CONNtrol trial | 10.1111/joim.13618 |  | Not at all relevant |  |  |  |
| A Randomized Clinical Trial of Clinician-Supported PTSD Coach in VA Primary Care Patients | 10.1007/s11606-023-08130-6 |  | Not at all relevant |  |  |  |
| Clinical utility of a mHealth assisted intervention for activity modulation in chronic pain: The pilot implementation of pain ROADMAP | 10.1002/ejp.2104 |  | Not at all relevant |  |  |  |
| Effectiveness of a smartphone application on dental anxiety in adolescents: A randomized controlled trial | 10.1111/ipd.13064 |  | Not at all relevant |  |  |  |
| Structural inequities contribute to racial/ethnic differences in neurophysiological tone, but not threat reactivity, after trauma exposure | 10.1038/s41380-023-01971-x |  | Not at all relevant |  |  |  |
| Experiences of participating in group-based rehabilitation programmes: A qualitative study of community-dwelling adults with post-stroke aphasia | 10.1111/1460-6984.12845 |  | Not at all relevant |  |  |  |
| The Impact of Varied Music Applications on Pain Perception and Situational Pain Catastrophizing | 10.1016/j.jpain.2023.01.006 |  | Not at all relevant |  |  |  |
| A mobile-based aftercare intervention to increase self-esteem in inpatients diagnosed with depression: A randomized controlled trial | 10.1080/10503307.2022.2157226 |  | Not at all relevant |  |  |  |
| Technology-based communication among Hurricane Maria survivors in the United States: a trans-territorial lens | 10.1007/s00127-022-02404-w |  | Not at all relevant |  |  |  |
| Field Visit Contact Rate by Mobile Crisis Teams as a Crisis System Performance Metric | 10.1176/appi.ps.202100736 |  | Not at all relevant |  |  |  |
| Effect of mHealth interventions on psychological issues experienced by women undergoing chemotherapy for breast cancer: A systematic review and meta-analysis | 10.1111/jocn.16533 | Review paper |  |  |  |  |
| Exploring the Safety of a General Digital Mental Health Intervention to Effect Symptom Reduction among Individuals with and without Suicidal Ideation: A Secondary Analysis | 10.1080/13811118.2022.2096520 |  |  | App usability |  |  |
| Questionnaire survey about the effects of new lifestyles during the pandemic of COVID-19 on upper limb diseases | 10.1016/j.jos.2022.06.005 |  | Not at all relevant |  |  |  |
| The Relationship Between Internet Use and Mental Health Among Chinese Residents During the (COVID)-19 Pandemic: A Cross-Sectional Study | 10.5993/AJHB.47.3.10 |  | Not at all relevant |  |  |  |
| Synergistic Effect of Nano-Silica and Intumescent Flame Retardant on the Fire Reaction Properties of Polypropylene Composites | 10.3390/ma16134759 |  | Not at all relevant |  |  |  |
| A RE-AIM Analysis of a Mental Health App for Undergraduate and Medical Students during the COVID-19 Pandemic: A Retrospective Cross-Sectional Study | 10.3390/ijerph20136266 |  | Not at all relevant |  |  |  |
| Psychometric Evidence of the Arabic Version of Nomophobia Questionnaire Among Physical Education Students | 10.2147/PRBM.S416312 |  | Not at all relevant |  |  |  |
| Barriers to and Facilitators of Using Remote Measurement Technology in the Long-Term Monitoring of Individuals With ADHD: Interview Study | 10.2196/44126 |  | Not at all relevant |  |  |  |
| An Online Mindfulness Intervention for International Students: A Randomized Controlled Feasibility Trial | 10.32872/cpe.9341 |  | Not at all relevant |  |  |  |
| Western Balkan Network for Youth Support and Counselling Service in Digital Era | 10.3233/SHTI230568 |  | Not at all relevant |  |  |  |
| Why mobile social media-related fear of missing out promotes depressive symptoms? the roles of phubbing and social exclusion | 10.1186/s40359-023-01231-1 |  | Not at all relevant |  |  |  |
| The Effect of an Adapted Digital Mental Health Intervention for Sickle Cell Disease on Engagement: A Pilot Randomized Controlled Trial | 10.21203/rs.3.rs-3073103/v1 |  | Not at all relevant |  |  |  |
| Issues and opportunities of digital phenotyping: ecological momentary assessment and behavioral sensing in protecting the young from suicide | 10.3389/fpsyg.2023.1103703 |  | Not at all relevant |  |  |  |
| Moderators and mediators of change of an internet-based mindfulness intervention for college students: secondary analysis from a randomized controlled trial | 10.3389/fdgth.2023.1179216 |  | Not at all relevant |  |  |  |
| A Smartphone-Delivered Program (Anathema) to Promote the Sexual Health of Older Adults, Colorectal Cancer Survivors, and Stroke Survivors: Protocol for a Feasibility Pilot Randomized Controlled Trial | 10.2196/46734 |  | Not at all relevant |  |  |  |
| Understanding the Challenges of Intensive Care Staff in Communicating With Patients and Patients' Families During the COVID-19 Crisis: A Qualitative Exploration | 10.7759/cureus.40961 |  | Not at all relevant |  |  |  |
| Effectiveness of mHealth consultation services for preventing postpartum depressive symptoms: a randomized clinical trial | 10.1186/s12916-023-02918-3 |  | Not at all relevant |  |  |  |
| Real-Time Real-World Digital Monitoring of Adolescent Suicide Risk During the Six Months Following Emergency Department Discharge: Protocol for an Intensive Longitudinal Study | 10.2196/46464 |  | Not at all relevant |  |  |  |
| The Effectiveness of a Mobile Phone-Based Physical Activity Program for Treating Depression, Stress, Psychological Well-Being, and Quality of Life Among Adults: Quantitative Study | 10.2196/46286 |  |  | No Mobile app |  |  |
| Design and Implementation of a Digitally Enabled Care Pathway to Improve Management of Depression in a Large Health Care System: Protocol for the Implementation of a Patient Care Platform | 10.2196/43788 |  | Not at all relevant |  |  |  |
| Detecting Clinically Relevant Emotional Distress and Functional Impairment in Children and Adolescents: Protocol for an Automated Speech Analysis Algorithm Development Study | 10.2196/46970 |  | Not at all relevant |  |  |  |
| Real-World Evidence of Aripiprazole Tablets with Sensor: Treatment Patterns and Impacts on Psychiatric Healthcare Resource Utilization | 10.2147/CEOR.S402357 |  | Not at all relevant |  |  |  |
| Development of a digital biomarker and intervention for subclinical depression: study protocol for a longitudinal waitlist control study | 10.1186/s40359-023-01215-1 |  | Not at all relevant |  |  |  |
| Varicose Vein Education and Informed coNsent (VVEIN) study: a randomised controlled pilot feasibility study | 10.1186/s40814-023-01336-9 |  | Not at all relevant |  |  |  |
| Prevention and Treatment of Social Anxiety Disorder in Adolescents: Protocol for a Randomized Controlled Trial of the Online Guided Self-Help Intervention SOPHIE | 10.2196/44346 |  | Not at all relevant |  |  |  |
| Development of a smartphone virtual reality game to support the radiation therapy of children and adolescents in proton centers | 10.3389/fped.2023.1163022 |  | Not at all relevant |  |  |  |
| Blended Learning for the "Multi-Track" Undergraduate Students in Ghana in an Adverse Era | 10.1016/j.sciaf.2023.e01772 |  | Not at all relevant |  |  |  |
| A single-blind, parallel-group randomised trial of a Technology-assisted and remotely delivered Cognitive Behavioural Therapy intervention (Tech-CBT) versus usual care to reduce anxiety in people with mild cognitive impairment and dementia: study protocol for a randomised trial | 10.1186/s13063-023-07381-2 |  | Not at all relevant |  |  |  |
| Effectiveness of internet-based and mobile-based interventions for adults with overweight or obesity experiencing symptoms of depression: a systematic review protocol | 10.1136/bmjopen-2022-067930 | Review paper |  |  |  |  |
| Characteristics of Users and Nonusers of Symptom Checkers in Germany: Cross-Sectional Survey Study | 10.2196/46231 |  | Not at all relevant |  |  |  |
| Introduction to the Coproduction of Supervision Standards for Digital Peer Support: Qualitative Study | 10.2196/40607 |  | Not at all relevant |  |  |  |
| A Proof of Concept Study on Individual Trends in Suicidal Ideation: An Ecological Momentary Assessment Study of 5 Patients Over Three Months | 10.17505/jpor.2023.25265 |  | Not at all relevant |  |  |  |
| Models of Gender Dysphoria Using Social Media Data for Use in Technology-Delivered Interventions: Machine Learning and Natural Language Processing Validation Study | 10.2196/47256 |  | Not at all relevant |  |  |  |
| A Gamified Mobile App That Helps People Develop the Metacognitive Skills to Cope With Stressful Situations and Difficult Emotions: Formative Assessment of the InsightApp | 10.2196/44429 |  |  | No depression,anxiety,stress |  |  |
| Feasibility and Preliminary Efficacy of Digital Interventions for Depressive Symptoms in Working Adults: Multiarm Randomized Controlled Trial | 10.2196/41590 |  | Not at all relevant |  |  |  |
| Money can't buy happiness: A randomized controlled trial of a digital mental health app with versus without financial incentives | 10.1177/20552076231170693 |  | Not at all relevant |  |  |  |
| Use, satisfaction, and preference of online health services among older adults with multimorbidity in Hong Kong primary care during COVID-19 | 10.1186/s12877-023-04061-3 |  | Not at all relevant |  |  |  |
| The Detection of Acute Risk of Self-injury Project: Protocol for an Ecological Momentary Assessment Study Among Individuals Seeking Treatment | 10.2196/46244 |  | Not at all relevant |  |  |  |
| Patient preferences for key drivers and facilitators of adoption of mHealth technology to manage depression: A discrete choice experiment | 10.1016/j.jad.2023.03.030 |  | Not at all relevant |  |  |  |
| App-based interventions for the prevention of postpartum depression: a systematic review and meta-analysis | 10.1186/s12884-023-05749-5 | Review paper |  |  |  |  |
| Effectiveness of a gamified digital intervention based on lifestyle modification (iGAME) in secondary prevention: a protocol for a randomised controlled trial | 10.1136/bmjopen-2022-066669 |  | Not at all relevant |  |  |  |
| A closed-body preclinical model to investigate blast-induced spinal cord injury | 10.3389/fnmol.2023.1199732 |  | Not at all relevant |  |  |  |
| The intention to use mHealth applications among Dutch older adults prior and during the COVID pandemic | 10.3389/fpubh.2023.1130570 |  | Not at all relevant |  |  |  |
| Evaluating the utility of daily speech assessments for monitoring depression symptoms | 10.1177/20552076231180523 |  |  | Screening, no treatment |  |  |
| Mobile health in the specific management of first-episode psychosis: a systematic literature review | 10.3389/fpsyt.2023.1137644 | Review paper |  |  |  |  |
| The effects of everyday-life social interactions on anxiety-related autonomic responses differ between men and women | 10.1038/s41598-023-36118-z |  | Not at all relevant |  |  |  |
| Implementing a Digital Depression Prevention Program in Australian Secondary Schools: Cross-Sectional Qualitative Study | 10.2196/42349 |  | Not at all relevant |  |  |  |
| Effects of Prolonged Fasting during Inpatient Multimodal Treatment on Pain and Functional Parameters in Knee and Hip Osteoarthritis: A Prospective Exploratory Observational Study | 10.3390/nu15122695 |  | Not at all relevant |  |  |  |
| Acceptability of an mHealth App for Monitoring Perinatal and Postpartum Mental Health: Qualitative Study With Women and Providers | 10.2196/44500 |  | Not at all relevant |  |  |  |
| Feasibility and Acceptability of the Aboriginal and Islander Mental Health Initiative for Youth App: Nonrandomized Pilot With First Nations Young People | 10.2196/40111 |  | Not at all relevant |  |  |  |
| Promoting Engagement With Smartphone Apps for Suicidal Ideation in Young People: Development of an Adjunctive Strategy Using a Lived Experience Participatory Design Approach | 10.2196/45234 |  | Not at all relevant |  |  |  |
| Decision Aids for Patients With Head and Neck Cancer: Qualitative Elicitation of Design Recommendations From Patient End Users | 10.2196/43551 |  | Not at all relevant |  |  |  |
| Digital health technologies for peripartum depression management among low-socioeconomic populations: perspectives from patients, providers, and social media channels | 10.1186/s12884-023-05729-9 |  | Not at all relevant |  |  |  |
| Understanding the impact of an AI-enabled conversational agent mobile app on users' mental health and wellbeing with a self-reported maternal event: a mixed method real-world data mHealth study | 10.3389/fgwh.2023.1084302 |  | Not at all relevant |  |  |  |
| Exercise Alleviates Behavioral Disorders but Shapes Brain Metabolism of APP/PS1 Mice in a Region- and Exercise-Specific Manner | 10.1021/acs.jproteome.2c00691 |  | Not at all relevant |  |  |  |
| Reduced Environmental Stimulation Therapy (REST) in anxiety and depression: An experience sampling study | 10.1016/j.xjmad.2023.100003 |  | Not at all relevant |  |  |  |
| The use of advanced technology and statistical methods to predict and prevent suicide | 10.1038/s44159-023-00175-y |  | Not at all relevant |  |  |  |
| A Validation Study of Mental Health Monitoring Through a Mobile Application | 10.30773/pi.2023.0047 |  | Not at all relevant |  |  |  |
| New living evidence resource of human and non-human studies for early intervention and research prioritisation in anxiety, depression and psychosis | 10.1136/bmjment-2023-300759 |  | Not at all relevant |  |  |  |
| Evaluation of factors strongly associated with nomophobia using structural equation modelling: A cross-sectional study | 10.1016/j.apnu.2023.04.006 |  | Not at all relevant |  |  |  |
| The more you avoid, the worse you will get: a cross-lagged panel model of shyness, mobile phone dependence and depression | 10.1080/09638237.2023.2210665 |  | Not at all relevant |  |  |  |
| The future of HIV pre-exposure prophylaxis adherence: reducing barriers and increasing opportunities | 10.1016/S2352-3018(23)00079-6 |  | Not at all relevant |  |  |  |
| Candidate biomarkers in psychiatric disorders: state of the field | 10.1002/wps.21078 |  | Not at all relevant |  |  |  |
| Day-to-day individual alpha frequency variability measured by a mobile EEG device relates to anxiety | 10.1111/ejn.16002 |  | Not at all relevant |  |  |  |
| Smarter prehospital clinical trials through a smartphone app | 10.1016/j.resuscitation.2023.109813 |  | Not at all relevant |  |  |  |
| Internet-Delivered, Therapist-Assisted Treatment for Anxiety and Depression in Patients with Cardiovascular Disease: Evidence-Base and Challenges | 10.1007/s11886-023-01867-w |  | Not at all relevant |  |  |  |
| Biological effects of exposure to 2650 MHz electromagnetic radiation on the behavior, learning, and memory of mice | 10.1002/brb3.3004 |  | Not at all relevant |  |  |  |
| Revisiting the factor structure of the suicide-related coping scale: Results from two samples of Australian online help-seekers | 10.1016/j.psychres.2023.115195 |  | Not at all relevant |  |  |  |
| Efficacy of smartphone applications to help cancer patients quit smoking: Protocol of the Quit2Heal randomized controlled trial | 10.1016/j.cct.2023.107180 |  | Not at all relevant |  |  |  |
| Evidence-based consensus guidelines on patient selection and trial stimulation for spinal cord stimulation therapy for chronic non-cancer pain | 10.1136/rapm-2022-104097 |  | Not at all relevant |  |  |  |
| The WeChat platform-based health education improves health outcomes among patients with stable coronary artery disease | 10.1016/j.pec.2023.107704 |  | Not at all relevant |  |  |  |
| A blended face-to-face and smartphone intervention to improve suicide prevention literacy and help-seeking intentions among construction workers: a randomised controlled trial | 10.1007/s00127-023-02429-9 |  |  | Telehealth app |  |  |
| Upregulating positive emotion in generalized anxiety disorder: A randomized controlled trial of the SkillJoy ecological momentary intervention | 10.1037/ccp0000794 |  | Not at all relevant |  |  |  |
| The associations between HIV stigma and mental health symptoms, life satisfaction, and quality of life among Black sexual minority men with HIV | 10.1007/s11136-023-03342-z |  | Not at all relevant |  |  |  |
| Feasibility of Mobile App-based Coping Skills Training for Cardiorespiratory Failure Survivors: The Blueprint Pilot Randomized Controlled Trial | 10.1513/AnnalsATS.202210-890OC |  | Not at all relevant |  |  |  |
| The role of DNA methylation in progression of neurological disorders and neurodegenerative diseases as well as the prospect of using DNA methylation inhibitors as therapeutic agents for such disorders | 10.1016/j.ibneur.2022.12.002 |  | Not at all relevant |  |  |  |
| The Victoria Assistive Devices and Coach (VADAC) study | 10.17269/s41997-022-00717-6 |  | Not at all relevant |  |  |  |
| The Grow It! app-longitudinal changes in adolescent well-being during the COVID-19 pandemic: a proof-of-concept study | 10.1007/s00787-022-01982-z |  | Not at all relevant |  |  |  |
| Can assessment of disease burden and quality of life based on mobility level in patients with end-stage cancer provide an insight into unmet needs? An exploratory cross-sectional study | 10.1080/09593985.2022.2035029 |  | Not at all relevant |  |  |  |
| Development and Validation of a Smartphone-Based App for the Longitudinal Assessment of Anxiety in Daily Life | 10.1177/10731911211065166 |  | Not at all relevant |  |  |  |
| Neural networks for clustered and longitudinal data using mixed effects models | 10.1111/biom.13615 |  | Not at all relevant |  |  |  |
| Preschool children's screen time during the COVID-19 pandemic: associations with family characteristics and children's anxiety/withdrawal and approaches to learning | 10.1007/s12144-023-04783-y |  | Not at all relevant |  |  |  |
| SmartMoms - a web application to raise awareness and provide information on postpartum depression | 10.1186/s12884-023-05680-9 |  |  | App usability |  |  |
| Skill Enactment and Knowledge Acquisition in Digital Cognitive Behavioral Therapy for Depression and Anxiety: Systematic Review of Randomized Controlled Trials | 10.2196/44673 | Review paper |  |  |  |  |
| Study protocol for a randomized controlled trial investigating the effect of online interpretation bias intervention on stress reactivity in the children of parents with depression: the CoCo study | 10.1186/s12888-023-04803-y |  | Not at all relevant |  |  |  |
| Affectionate touch and diurnal oxytocin levels: An ecological momentary assessment study | 10.7554/eLife.81241 |  | Not at all relevant |  |  |  |
| Reliability and Validity of Noncognitive Ecological Momentary Assessment Survey Response Times as an Indicator of Cognitive Processing Speed in People's Natural Environment: Intensive Longitudinal Study | 10.2196/45203 |  | Not at all relevant |  |  |  |
| A Systematic Review of Location Data for Depression Prediction | 10.3390/ijerph20115984 | Review paper |  |  |  |  |
| The Use of Photoplethysmography in the Assessment of Mental Health: Scoping Review | 10.2196/40163 | Review paper |  |  |  |  |
| Early Life Obesity Increases Neuroinflammation, Amyloid Beta Deposition, and Cognitive Decline in a Mouse Model of Alzheimer's Disease | 10.3390/nu15112494 |  | Not at all relevant |  |  |  |
| Gastric Alimetry (®) improves patient phenotyping in gastroduodenal disorders compared to gastric emptying scintigraphy alone | 10.1101/2023.05.18.23290134 |  | Not at all relevant |  |  |  |
| Study protocol for a randomised controlled trial to determine the effectiveness of a mHealth application as a family supportive tool in paediatric otolaryngology perioperative process (TONAPP) | 10.1186/s13063-023-07376-z |  | Not at all relevant |  |  |  |
| Internet-Based Behavioral Activation for Depression: Systematic Review and Meta-Analysis | 10.2196/41643 | Review paper |  |  |  |  |
| Understanding feeling "high" and its role in medical cannabis patient outcomes | 10.3389/fphar.2023.1135453 |  | Not at all relevant |  |  |  |
| Efficacy of Intellect's self-guided anxiety and worry mobile health programme: A randomized controlled trial with an active control and a 2-week follow-up | 10.1371/journal.pdig.0000095 |  | Not at all relevant |  |  |  |
| Prediction of Diagnosis and Treatment Response in Adolescents With Depression by Using a Smartphone App and Deep Learning Approaches: Usability Study | 10.2196/45991 |  | Not at all relevant |  |  |  |
| A CBT-based mobile intervention as an adjunct treatment for adolescents with symptoms of depression: a virtual randomized controlled feasibility trial | 10.3389/fdgth.2023.1062471 |  |  | App usability |  |  |
| Optimization of smartphone psychotherapy for depression and anxiety among patients with cancer using the multiphase optimization strategy (MOST) framework and decentralized clinical trial system (SMartphone Intervention to LEssen depression/Anxiety and GAIN resilience: SMILE AGAIN project): a protocol for a randomized controlled trial | 10.1186/s13063-023-07307-y |  | Not at all relevant |  |  |  |
| Evaluating the Modified Patient Health Questionnaire-2 and Insomnia Severity Index-2 for Daily Digital Screening of Depression and Insomnia: Validation Study | 10.2196/45543 |  | Not at all relevant |  |  |  |
| Factors Associated With Online Patient-Provider Communications Among Cancer Survivors in the United States During COVID-19: Cross-sectional Study | 10.2196/44339 |  | Not at all relevant |  |  |  |
| Psychosocial and personality trait associates of phubbing and being phubbed in hispanic emerging adult college students | 10.1007/s12144-023-04767-y |  | Not at all relevant |  |  |  |
| Estimated prevalence and sociodemographic correlates of mental disorders in medical students of Hebei Province, China: A cross-sectional study | 10.5498/wjp.v13.i5.215 |  | Not at all relevant |  |  |  |
| Physical Versus Virtual Reality-Based Calm Rooms for Psychiatric Inpatients: Quasi-Randomized Trial | 10.2196/42365 |  | Not at all relevant |  |  |  |
| Clinical Practice Guideline: The Diagnosis and Treatment of Unipolar Depression—National Disease Management Guideline | 10.3238/arztebl.m2023.0074 |  | Not at all relevant |  |  |  |
| Longitudinal assessment and determinants of short-term and longer-term psychological distress in a sample of healthcare workers during the COVID-19 pandemic in Quebec, Canada | 10.3389/fpsyt.2023.1112184 |  | Not at all relevant |  |  |  |
| Protocol for the promoting resilience in stress management (PRISM) intervention: a multi-site randomized controlled trial for adolescents and young adults with advanced cancer | 10.1186/s12904-023-01179-4 |  | Not at all relevant |  |  |  |
| Nonpharmacological Behavior Guidance for Children During Preventive Dental Visits: A Systematic Review-Part 1 |  | Review paper |  |  |  |  |
| Decreased miR-451a in cerebrospinal fluid, a marker for both cognitive impairment and depressive symptoms in Alzheimer's disease | 10.7150/thno.81826 |  | Not at all relevant |  |  |  |
| Associations Between Smartphone Keystroke Metadata and Mental Health Symptoms in Adolescents: Findings From the Future Proofing Study | 10.2196/44986 |  | Not at all relevant |  |  |  |
| Mobile Acceptance and Commitment Therapy With Distressed First-Generation College Students: Microrandomized Trial | 10.2196/43065 |  | Not at all relevant |  |  |  |
| Effect of the COVID-19 Pandemic on Medical Students at the University of Jeddah, Saudi Arabia | 10.7759/cureus.38968 |  | Not at all relevant |  |  |  |
| The role of self-efficacy and parental communication in the association between cyber victimization and depression among adolescents and young adults: a structural equation model | 10.1186/s12888-023-04841-6 |  | Not at all relevant |  |  |  |
| Gamifying Cognitive Behavioral Therapy Techniques on Smartphones for Bangkok's Millennials With Depressive Symptoms: Interdisciplinary Game Development | 10.2196/41638 |  | Not at all relevant |  |  |  |
| A digital health intervention to support patients with chronic pain during prescription opioid tapering: a pilot randomised controlled trial | 10.1101/2023.05.10.23289771 |  | Not at all relevant |  |  |  |
| Patient-reported impact of myasthenia gravis in the real world: findings from a digital observational survey-based study (MyRealWorld MG) | 10.1136/bmjopen-2022-068104 |  | Not at all relevant |  |  |  |
| Development of "LvL UP 1.0": a smartphone-based, conversational agent-delivered holistic lifestyle intervention for the prevention of non-communicable diseases and common mental disorders | 10.3389/fdgth.2023.1039171 |  | Not at all relevant |  |  |  |
| Online Contingent Attention Training (OCAT): transfer effects to cognitive biases, rumination, and anxiety symptoms from two proof-of-principle studies | 10.1186/s41235-023-00480-3 |  | Not at all relevant |  |  |  |
| Optimal number of charging station and pricing strategy for the electric vehicle with component commonality considering consumer range anxiety | 10.1371/journal.pone.0283320 |  | Not at all relevant |  |  |  |
| Patient information, communication and competence empowerment in oncology: Results and learnings from the PIKKO study | 10.1007/s00520-023-07781-9 |  | Not at all relevant |  |  |  |
| Preliminary Evaluation of Translated and Culturally Adapted Internet-Delivered Cognitive Therapy for Social Anxiety Disorder: Multicenter, Single-Arm Trial in Japan | 10.2196/45136 |  | Not at all relevant |  |  |  |
| Influence of on emotions and behavior of adolescents with major depressive disorder | 10.1016/j.heliyon.2023.e15890 |  | Not at all relevant |  |  |  |
| Serious Games Based on Cognitive Bias Modification and Learned Helplessness Paradigms for the Treatment of Depression: Design and Acceptability Study | 10.2196/37105 |  | Not at all relevant |  |  |  |
| Mom's Good Mood: screening and management of perinatal depression within primary healthcare system in China-protocol for an effectiveness-implementation design study | 10.1136/bmjopen-2022-063593 |  | Not at all relevant |  |  |  |
| Adaptation of a mobile app for early anxiety and depression intervention in university students in Chile: participatory study | 10.1192/bji.2023.1 |  |  | Screening, no treatment |  |  |
| mHealth and eHealth Applications for a Medicalized Quarantine Hotel during the COVID-19 Pandemic | 10.1055/s-0043-1769912 |  | Not at all relevant |  |  |  |
| The Diabetic Foot-Pain-Depression Cycle | 10.7547/22-126 |  | Not at all relevant |  |  |  |
| Digital health and human rights of young adults in Ghana, Kenya and Vietnam: a qualitative participatory action research study | 10.1136/bmjgh-2022-011254 |  | Not at all relevant |  |  |  |
| Cardiovascular prevention in women: an update by the Italian Society of Cardiology working group on 'Prevention, hypertension and peripheral disease' | 10.2459/JCM.0000000000001423 |  | Not at all relevant |  |  |  |
| The effect of technology-based interventions on child and parent outcomes in pediatric oncology: A systemic review of experimental evidence | 10.1016/j.apjon.2023.100219 | Review paper |  |  |  |  |
| Social media and psychology of language learning: The role of telegram-based instruction on academic buoyancy, academic emotion regulation, foreign language anxiety, and English achievement | 10.1016/j.heliyon.2023.e15830 |  | Not at all relevant |  |  |  |
| Association of parent-child relationship quality and problematic mobile phone use with non-suicidal self-injury among adolescents | 10.1186/s12888-023-04786-w |  | Not at all relevant |  |  |  |
| The Synergistic Effect of Nurse Proactive Phone Calls With an mHealth App Program on Sustaining App Usage: 3-Arm Randomized Controlled Trial | 10.2196/43678 |  | Not at all relevant |  |  |  |
| The efficacy of self-guided internet and mobile-based interventions for preventing anxiety and depression - A systematic review and meta-analysis | 10.1016/j.brat.2023.104292 | Review paper |  |  |  |  |
| [Development of demetia therapeutics regurating synaptic plasticity] | 10.1254/fpj.22138 |  | Not at all relevant |  |  |  |
| Speech as a promising biosignal in precision psychiatry | 10.1016/j.neubiorev.2023.105121 |  | Not at all relevant |  |  |  |
| How to set up a mobile X-ray unit in the community - Implementation initiatives for patient-centred care | 10.1016/j.radi.2023.02.027 |  | Not at all relevant |  |  |  |
| A randomized controlled study: Evaluating the efficacy of a mobile application developed for mothers who have children with epilepsy in Turkiye | 10.1016/j.pedn.2023.02.010 |  | Not at all relevant |  |  |  |
| Association Between Frequent Use of Makeup and Presence of Depressive Symptoms-Population-Based Observational Study, Including 2400 Participants | 10.1007/s13555-023-00911-5 |  | Not at all relevant |  |  |  |
| A systematic review and meta-analysis of self-guided online acceptance and commitment therapy as a transdiagnostic self-help intervention | 10.1080/16506073.2023.2178498 | Review paper |  |  |  |  |
| A smartphone-based supportive counseling on health anxiety and acceptance of disability in Systemic Lupus Erythematosus patients: A randomized clinical trial | 10.1016/j.pec.2023.107676 |  | Not at all relevant |  |  |  |
| Feasibility, Acceptability, and Preliminary HIV Care and Psychological Health Effects of iTHRIVE 365 for Black Same Gender Loving Men | 10.1097/QAI.0000000000003167 |  | Not at all relevant |  |  |  |
| The selfBACK artificial intelligence-based smartphone app can improve low back pain outcome even in patients with high levels of depression or stress | 10.1002/ejp.2080 |  | Not at all relevant |  |  |  |
| Patient perceptions of a remote assessment pathway in otology: a qualitative descriptive analysis | 10.1007/s00405-022-07684-1 |  | Not at all relevant |  |  |  |
| Home to Stay: A Randomized Controlled Trial Evaluating the Effect of a Postdischarge Mobile App to Reduce 30-Day Readmission Following Elective Colorectal Surgery | 10.1097/SLA.0000000000005527 |  | Not at all relevant |  |  |  |
| Dental education profile in COVID-19 pandemic: A scoping review | 10.1111/eje.12798 | Review paper |  |  |  |  |
| Rising early warning signals in affect associated with future changes in depression: a dynamical systems approach | 10.1017/S0033291721005183 |  | Not at all relevant |  |  |  |
| Predicting short-term suicidal thoughts in adolescents using machine learning: developing decision tools to identify daily level risk after hospitalization | 10.1017/S0033291721005006 |  | Not at all relevant |  |  |  |
| The Effect of Loneliness on Death Anxiety in the Elderly During the COVID-19 Pandemic | 10.1177/00302228211010587 |  | Not at all relevant |  |  |  |
| Positive Prospective Mental Imagery Characteristics in Young Adults and Their Associations with Depressive Symptoms | 10.1007/s10608-023-10378-5 |  | Not at all relevant |  |  |  |
| Internet-based and mobile-based cognitive behavioral therapy for chronic diseases: a systematic review and meta-analysis | 10.1038/s41746-023-00809-8 | Review paper |  |  |  |  |
| Situating Meditation Apps Within the Ecosystem of Meditation Practice: Population-Based Survey Study | 10.2196/43565 |  | Not at all relevant |  |  |  |
| Conversational Agent Interventions for Mental Health Problems: Systematic Review and Meta-analysis of Randomized Controlled Trials | 10.2196/43862 | Review paper |  |  |  |  |
| Efficacy of an mHealth Behavior Change Intervention for Promoting Physical Activity in the Workplace: Randomized Controlled Trial | 10.2196/44108 |  | Not at all relevant |  |  |  |
| Mediating effect of mindfulness level on the relationship between marital quality and postpartum depression among primiparas | 10.12998/wjcc.v11.i12.2729 |  | Not at all relevant |  |  |  |
| The Impact of Japan's Soft Lockdown on Depressive Symptoms among Community-Dwelling Older Adults | 10.3390/healthcare11091239 |  | Not at all relevant |  |  |  |
| An Unguided, Computerized Cognitive Behavioral Therapy Intervention (TreadWill) in a Lower Middle-Income Country: Pragmatic Randomized Controlled Trial | 10.2196/41005 |  | Not at all relevant |  |  |  |
| The effect of the Ebe Evimde application on the self-efficacy and anxiety levels of mothers: Randomized controlled trial | 10.1177/20552076231169840 |  | Not at all relevant |  |  |  |
| Does an app designed to reduce repetitive negative thinking decrease depression and anxiety in young people? (RETHINK): a randomized controlled prevention trial | 10.1186/s13063-023-07295-z |  | Not at all relevant |  |  |  |
| Gender-based differences in interaction effects between childhood maltreatment and problematic mobile phone use on college students' depression and anxiety symptoms | 10.1186/s12888-023-04777-x |  | Not at all relevant |  |  |  |
| A smartphone-based serious game for depressive symptoms: Protocol for a pilot randomized controlled trial | 10.1016/j.invent.2023.100624 |  | Not at all relevant |  |  |  |
| Beyond Screen Time: The Different Longitudinal Relations between Adolescents' Smartphone Use Content and Their Mental Health | 10.3390/children10050770 |  | Not at all relevant |  |  |  |
| Exploring the potential of mobile health interventions to address behavioural risk factors for the prevention of non-communicable diseases in Asian populations: a qualitative study | 10.1186/s12889-023-15598-8 |  | Not at all relevant |  |  |  |
| Cardio4Health Study, a Cardiac Telerehabilitation Pilot Program Aimed at Patients After an Ischemic Event: Cross-sectional Study | 10.2196/44179 |  | Not at all relevant |  |  |  |
| Focusing on Digital Research Priorities for Advancing the Access and Quality of Mental Health | 10.2196/47898 |  | Not at all relevant |  |  |  |
| Effect of transcutaneous auricular vagal nerve stimulation on the fatigue syndrome in patients with gastrointestinal cancers - FATIVA: a randomized, placebo-controlled pilot study protocol | 10.1186/s40814-023-01289-z |  | Not at all relevant |  |  |  |
| Online group music therapy: proactive management of undergraduate students' stress and anxiety | 10.3389/fpsyt.2023.1183311 |  | Not at all relevant |  |  |  |
| Strength Back - A qualitative study on the co-creation of a positive psychology digital health intervention for spinal surgery patients | 10.3389/fpsyg.2023.1117357 |  | Not at all relevant |  |  |  |
| Improving adherence and health outcomes in testicular cancer survivors using a mobile health-based intervention: A mixed-methods pilot study | 10.1177/20552076231167002 |  | Not at all relevant |  |  |  |
| The associations between screen time and mental health in adolescents: a systematic review | 10.1186/s40359-023-01166-7 | Review paper |  |  |  |  |
| Effect of a novel telehealth device for dietary cognitive behavioral intervention in overweight or obesity care | 10.1038/s41598-023-33238-4 |  | Not at all relevant |  |  |  |
| Exploring the Relationship Between Privacy and Utility in Mobile Health: Algorithm Development and Validation via Simulations of Federated Learning, Differential Privacy, and External Attacks | 10.2196/43664 |  | Not at all relevant |  |  |  |
| Mobile Acceptance and Commitment Therapy in Bipolar Disorder: Microrandomized Trial | 10.2196/43164 |  | Not at all relevant |  |  |  |
| [Creation of Protective Equipment for Portable Radiography in Neonatal Intensive Care Unit] | 10.6009/jjrt.2023-1296 |  | Not at all relevant |  |  |  |
| Geolocation Patterns, Wi-Fi Connectivity Rates, and Psychiatric Symptoms Among Urban Homeless Youth: Mixed Methods Study Using Self-report and Smartphone Data | 10.2196/45309 |  | Not at all relevant |  |  |  |
| Network analysis of the relationships between problematic smartphone use and anxiety, and depression in a sample of Chinese college students | 10.3389/fpsyt.2023.1097301 |  | Not at all relevant |  |  |  |
| Determining the Acceptance of Digital Cardiac Rehabilitation and Its Influencing Factors among Patients Affected by Cardiac Diseases | 10.3390/jcdd10040174 |  | Not at all relevant |  |  |  |
| Digital Technology Use and Mental Health Consultations: Survey of the Views and Experiences of Clinicians and Young People | 10.2196/44064 |  | Not at all relevant |  |  |  |
| Translating Research Evidence Into Marketplace Application: Cohort Study of Internet-Based Intervention Platforms for Perinatal Depression | 10.2196/42777 |  | Not at all relevant |  |  |  |
| Digital Interventions for Treating Post-COVID or Long-COVID Symptoms: Scoping Review | 10.2196/45711 | Review paper |  |  |  |  |
| Children infected vs. uninfected with COVID-19: Differences in parent reports of the use of mobile phones to calm children, routines, parent-child relationship, and developmental outcomes | 10.3389/fpubh.2023.1114597 |  | Not at all relevant |  |  |  |
| Preventing Depression Relapse: A Qualitative Study on the Need for Additional Structured Support Following Mindfulness-Based Cognitive Therapy | 10.1177/27536130221144247 |  | Not at all relevant |  |  |  |
| Legalised active assistance in dying: palliative care stakeholders' national e-consultation | 10.1136/spcare-2022-004081 |  | Not at all relevant |  |  |  |
| Sensa Mobile App for Managing Stress, Anxiety, and Depression Symptoms: Pilot Cohort Study | 10.2196/40671 |  |  |  | No treatment |  |
| Descriptive study: the novel "full spectrum people-with-opioid-use-disorder care model" | 10.1186/s12954-023-00778-x |  | Not at all relevant |  |  |  |
| Time-Dependent Changes in Depressive Symptoms Among Control Participants in Digital-Based Psychological Intervention Studies: Meta-analysis of Randomized Controlled Trials | 10.2196/39029 |  | Not at all relevant |  |  |  |
| Barriers to and Facilitators of a Blended Cognitive Behavioral Therapy Program for Depression and Anxiety Based on Experiences of University Students: Qualitative Interview Study | 10.2196/45970 |  | Not at all relevant |  |  |  |
| Use of a Mobile Biofeedback App to Provide Health Coaching for Stress Self-management: Pilot Quasi-Experiment | 10.2196/41018 |  | Not at all relevant |  |  |  |
| Downregulation of Sirtuin 1 Does Not Account for the Impaired Long-Term Potentiation in the Prefrontal Cortex of Female APPswe/PS1dE9 Mice Modelling Alzheimer's Disease | 10.3390/ijms24086968 |  | Not at all relevant |  |  |  |
| Protocol for The Promoting Resilience in Stress Management (PRISM) Intervention: a multi-site randomized controlled trial for adolescents and young adults with advanced cancer | 10.21203/rs.3.rs-2748874/v1 |  | Not at all relevant |  |  |  |
| Late-life depression: Epidemiology, phenotype, pathogenesis and treatment before and during the COVID-19 pandemic | 10.3389/fpsyt.2023.1017203 |  | Not at all relevant |  |  |  |
| The contribution of the smartphone use to reducing depressive symptoms of Chinese older adults: The mediating effect of social participation | 10.3389/fnagi.2023.1132871 |  | Not at all relevant |  |  |  |
| Current situation and influencing factors for suicidal intent in patients with intentional acute pesticide poisoning | 10.3389/fpubh.2023.1168176 |  | Not at all relevant |  |  |  |
| Feasibility and Acceptability of a Mindfulness-Based Smartphone App among Pregnant Women with Obesity | 10.3390/ijerph20075421 |  | Not at all relevant |  |  |  |
| Tailoring a Digital Mental Health Program for Patients With Sickle Cell Disease: Qualitative Study | 10.2196/44216 |  | Not at all relevant |  |  |  |
| The Efficacy and Usability of an Unguided Web-Based Grief Intervention for Adults Who Lost a Loved One During the COVID-19 Pandemic: Randomized Controlled Trial | 10.2196/43839 |  | Not at all relevant |  |  |  |
| Barriers and facilitators for referring women with positive perinatal depression screening results in China: a qualitative study | 10.1186/s12884-023-05532-6 |  | Not at all relevant |  |  |  |
| A WeChat-based Intervention, Wellness Enhancement for Caregivers (WECARE), for Chinese American Dementia Caregivers: Pilot Assessment of Feasibility, Acceptability, and Preliminary Efficacy | 10.2196/42972 |  | Not at all relevant |  |  |  |
| A Probiotic Intervention With Bifidobacterium longum NCC3001 on Perinatal Mood Outcomes (PROMOTE Study): Protocol for a Decentralized Randomized Controlled Trial | 10.2196/41751 |  | Not at all relevant |  |  |  |
| Evaluation of the Effectiveness of Therapy for Anxiety in Williams Beuren Syndrome Using a Smartphone App: Protocol for a Single-Case Experiment | 10.2196/44393 |  | Not at all relevant |  |  |  |
| The Perceived Utility of Smartphone and Wearable Sensor Data in Digital Self-tracking Technologies for Mental Health | 10.1145/3544548.3581209 |  | Not at all relevant |  |  |  |
| Smartphone-derived Virtual Keyboard Dynamics Coupled with Accelerometer Data as a Window into Understanding Brain Health: Smartphone Keyboard and Accelerometer as Window into Brain Health | 10.1145/3544548.3580906 |  | Not at all relevant |  |  |  |
| Comparative evaluation of Tell-Show-Do technique and its modifications in managing anxious pediatric dental patients among 4-8 years of age | 10.4103/jisppd.jisppd_242_23 |  | Not at all relevant |  |  |  |
| Longitudinal follow-up of the randomized controlled trial of access to the trauma-focused self-management app PTSD Coach | 10.1016/j.invent.2023.100618 |  | Not at all relevant |  |  |  |
| App-based intervention for reducing depressive symptoms in postpartum women: Protocol for a feasibility randomized controlled trial | 10.1016/j.invent.2023.100616 |  |  | No Mobile app |  |  |
| Improving Suicide Prevention Through Evidence-Based Strategies: A Systematic Review | 10.1176/appi.focus.23021004 | Review paper |  |  |  |  |
| Clinical Considerations for Digital Resources in Care for Patients With Suicidal Ideation | 10.1176/appi.focus.20220073 |  | Not at all relevant |  |  |  |
| Development of a Nomogram to Estimate the 60-Day Probability of Death or Culling Due to Severe Clinical Mastitis in Dairy Cows at First Veterinary Clinical Evaluation | 10.3390/vetsci10040268 |  | Not at all relevant |  |  |  |
| [Crossed views on depressive symptomatology with suicidal expression in the elderly] | 10.1016/j.revinf.2023.03.007 |  | Not at all relevant |  |  |  |
| Validation of the Nine-Item Avoidant/Restrictive Food Intake Disorder Screen Among Transgender and Nonbinary Youth and Young Adults | 10.1089/trgh.2021.0021 |  | Not at all relevant |  |  |  |
| [Direct determination of five xanthic acids in water by ultra performance liquid chromatography-tandem mass spectrometry] | 10.3724/SP.J.1123.2022.09002 |  | Not at all relevant |  |  |  |
| App-Based Mindfulness Intervention to Improve Psychological Outcomes in Pretransplant Patients With Heart Failure | 10.4037/ccn2023411 |  | Not at all relevant |  |  |  |
| Case 312: Dercum Disease | 10.1148/radiol.213044 |  | Not at all relevant |  |  |  |
| Characterizing observed and effective behavioral engagement with smartphone cognitive behavioral therapy for body dysmorphic disorder: A methods roadmap and use case | 10.1016/j.invent.2023.100615 |  | Not at all relevant |  |  |  |
| The effect of self-compassion-based programs for infertility (SCPI) on anxiety and depression among women pursuing fertility treatment: a three-armed, randomised controlled trial | 10.1007/s10815-023-02771-6 |  | Not at all relevant |  |  |  |
| Depressive symptoms, perceived social support, and anticipated HIV stigma among HIV-negative/unknown men who have sex with men in China during the COVID-19 pandemic: A multicenter online cross-sectional study | 10.1002/brb3.2946 |  | Not at all relevant |  |  |  |
| The effectiveness of a mobile application-based programme for rehabilitation after total hip or knee arthroplasty: A randomised controlled trial | 10.1016/j.ijnurstu.2023.104455 |  | Not at all relevant |  |  |  |
| [The effects of the COVID-19 pandemic on BAPU students: What analyzes were made by clinicians? The specifics of student care needs in a crisis context] | 10.1016/j.amp.2023.02.001 |  | Not at all relevant |  |  |  |
| A relational agent for treating substance use in adults: Protocol for a randomized controlled trial with a psychoeducational comparator | 10.1016/j.cct.2023.107125 |  | Not at all relevant |  |  |  |
| Text mining methods for the characterisation of suicidal thoughts and behaviour | 10.1016/j.psychres.2023.115090 |  | Not at all relevant |  |  |  |
| Psychiatric emergencies among urban youth during COVID-19: Volume and acuity in a multi-channel program for the publicly insured | 10.1016/j.jpsychires.2023.01.047 |  | Not at all relevant |  |  |  |
| Design and methods of a randomized controlled trial evaluating the effects of the PE Coach mobile application on prolonged exposure among veterans with PTSD | 10.1016/j.cct.2023.107115 |  | Not at all relevant |  |  |  |
| Optimizing Clinical Throughput with a Smartphone App: Easing the Burden of Surgical Scheduling | 10.1097/PRS.0000000000010027 |  | Not at all relevant |  |  |  |
| Improving emotion regulation in breast cancer patients in the early survivorship period: Efficacy of a brief ecologically boosted group intervention | 10.1002/pon.6105 |  | Not at all relevant |  |  |  |
| Psychological mobile app for patients with acute myeloid leukemia: A pilot randomized clinical trial | 10.1002/cncr.34645 |  | Not at all relevant |  |  |  |
| 'Being a mother is not child's play': The capabilities of mothers in a low-resource setting in South Africa | 10.1111/hex.13689 |  | Not at all relevant |  |  |  |
| Impact of Mobile Technology-Enhanced Follow-Up Program for Mothers with New-Born Babies on Mothers' Anxiety, Self-Efficacy, and Infant Health | 10.1080/07370016.2022.2163851 |  | Not at all relevant |  |  |  |
| Impact of parent-targeted eHealth educational interventions on infant procedural pain management: a systematic review | 10.11124/JBIES-21-00435 | Review paper |  |  |  |  |
| Should Mindfulness for Health Care Workers Go Virtual? A Mindfulness-Based Intervention Using Virtual Reality and Heart Rate Variability in the Emergency Department | 10.1177/21650799221123258 |  | Not at all relevant |  |  |  |
| Trial of a patient-directed eHealth program to ameliorate perinatal depression: the MomMoodBooster2 practical effectiveness study | 10.1016/j.ajog.2022.09.027 |  | Not at all relevant |  |  |  |
| A Digital Cognitive-Behavioral Intervention for Depression and Anxiety Among Adolescents and Young Adults | 10.1176/appi.ps.202200045 |  |  | Telehealth app |  |  |
| Daily life positive affect regulation in early adolescence: Associations with symptoms of depression | 10.1037/emo0001129 |  | Not at all relevant |  |  |  |
| Can you feel the excitement? Physiological correlates of students' self-reported emotions | 10.1111/bjep.12534 |  | Not at all relevant |  |  |  |
| Mobile primary healthcare for post-COVID patients in rural areas: a proof-of-concept study | 10.1007/s15010-022-01881-0 |  | Not at all relevant |  |  |  |
| Pilot Randomized Control Trial of an App-Based CBT Program for Reducing Anxiety in Individuals with ASD without Intellectual Disability | 10.1007/s10803-022-05617-9 |  | Not at all relevant |  |  |  |
| The Acceptability of a Smartphone App (BlueIce) for University Students Who Self-harm | 10.1080/13811118.2021.2022552 |  |  | App usability |  |  |
| College extracurricular involvement as a suicide prevention and wellness promotion strategy: Exploring the roles of social support and meaning | 10.1080/07448481.2021.1904952 |  | Not at all relevant |  |  |  |
| Effectiveness of laptop-based versus smartphone-based videoconferencing interaction on loneliness, depression and social support in nursing home residents: A secondary data analysis | 10.1177/1357633X20972004 |  | Not at all relevant |  |  |  |
| Impact of COVID-19 on depression and anxiety among healthcare professionals in Abu Dhabi | 10.1371/journal.pone.0282198 |  | Not at all relevant |  |  |  |
| The Effectiveness of a Smartphone Intervention Targeting Suicidal Ideation in Young Adults: Randomized Controlled Trial Examining the Influence of Loneliness | 10.2196/44862 |  |  | Screening, no treatment |  |  |
| Transformation of chronic disease management: Before and after the COVID-19 outbreak | 10.3389/fpubh.2023.1074364 |  | Not at all relevant |  |  |  |
| Perceptions of a Digital Mental Health Platform Among Participants With Depressive Disorder, Anxiety Disorder, and Other Clinically Diagnosed Mental Disorders in Singapore: Usability and Acceptability Study | 10.2196/42167 |  | Not at all relevant |  |  |  |
| Mobile health to promote physical activity in people post stroke or transient ischemic attack - study protocol for a feasibility randomised controlled trial | 10.1186/s12883-023-03163-0 |  | Not at all relevant |  |  |  |
| Relationship between depressive symptoms, social isolation, visual complaints and hearing loss in middle-aged and older adults | 10.22365/jpsych.2022.086 |  | Not at all relevant |  |  |  |
| Types of Digital-Based Nursing Interventions for Reducing Stress and Depression Symptoms on Adolescents During COVID-19 Pandemic: A Scoping Review | 10.2147/JMDH.S406688 | Review paper |  |  |  |  |
| A machine learning approach to identifying suicide risk among text-based crisis counseling encounters | 10.3389/fpsyt.2023.1110527 |  | Not at all relevant |  |  |  |
| Understanding the Adoption and Use of Digital Mental Health Apps Among College Students: Secondary Analysis of a National Survey | 10.2196/43942 |  | Not at all relevant |  |  |  |
| Examining a Resilience Mental Health App in Adolescents: Acceptability and Feasibility Study | 10.2196/38042 |  | Not at all relevant |  |  |  |
| ARAM: A Technology Acceptance Model to Ascertain the Behavioural Intention to Use Augmented Reality | 10.3390/jimaging9030073 |  | Not at all relevant |  |  |  |
| A Digital Intervention to Improve Mental Health and Interpersonal Resilience in Young People Who Have Experienced Technology-Assisted Sexual Abuse: Protocol for a Nonrandomized Feasibility Clinical Trial and Nested Qualitative Study | 10.2196/40539 |  | Not at all relevant |  |  |  |
| Effectiveness of Digital Mental Health Tools to Reduce Depressive and Anxiety Symptoms in Low- and Middle-Income Countries: Systematic Review and Meta-analysis | 10.2196/43066 | Review paper |  |  |  |  |
| Evaluation and Management of Dyspnea in Hypermobile Ehlers-Danlos Syndrome and Generalized Hypermobility Spectrum Disorder: Protocol for a Pilot and Feasibility Randomized Controlled Trial | 10.2196/44832 |  | Not at all relevant |  |  |  |
| Exploring the Perceptions of mHealth Interventions for the Prevention of Common Mental Disorders in University Students in Singapore: Qualitative Study | 10.2196/44542 |  | Not at all relevant |  |  |  |
| Factors Influencing Depression in Adolescents Focusing on the Degree of Appearance Stress | 10.3390/nursrep13010047 |  | Not at all relevant |  |  |  |
| The Efficacy of Be a Mom, a Web-Based Intervention to Prevent Postpartum Depression: Examining Mechanisms of Change in a Randomized Controlled Trial | 10.2196/39253 |  |  | No Mobile app |  |  |
| CBT-informed psychological interventions for adult patients with anxiety and depression symptoms: A narrative review of digital treatment options | 10.1016/j.jad.2023.01.057 | Review paper |  |  |  |  |
| Testing the mood brightening hypothesis: Hedonic benefits of physical, outdoor, and social activities in people with anxiety, depression or both | 10.1016/j.jad.2023.01.017 |  | Not at all relevant |  |  |  |
| Detection of bipolar disorder in the prodromal phase: A systematic review of assessment instruments | 10.1016/j.jad.2023.01.012 | Review paper |  |  |  |  |
| Development and validation of a machine learning-based vocal predictive model for major depressive disorder | 10.1016/j.jad.2022.12.117 |  | Not at all relevant |  |  |  |
| Investigating the effectiveness of a smart mental health intervention (inMind) for stress reduction during pharmacological treatment for mild to moderate major depressive disorders: Study protocol for a randomized control trial | 10.3389/fpsyt.2023.1034246 |  | Not at all relevant |  |  |  |
| Eye movement desensitization and reprocessing (EMDR) therapy or supportive counseling prior to exposure therapy in patients with panic disorder: study protocol for a multicenter randomized controlled trial (IMPROVE) | 10.1186/s12888-022-04320-4 |  | Not at all relevant |  |  |  |
| Smartphone app-based intervention for reducing stress, depression, and anxiety in caregivers of people with dementia in Vietnam: Study protocol for a pilot randomized controlled trial | 10.1177/20552076231163786 |  |  | Telehealth app |  |  |
| Identifying vulnerable populations in the electronic Framingham Heart Study to improve digital device adherence | 10.1038/s41746-023-00789-9 |  | Not at all relevant |  |  |  |
| Acceptability and Feasibility of "Village," a Digital Communication App for Young People Experiencing Low Mood, Thoughts of Self-harm, and Suicidal Ideation to Obtain Support From Family and Friends: Mixed Methods Pilot Open Trial | 10.2196/41273 |  | Not at all relevant |  |  |  |
| Codesign of a digital health tool for suicide prevention: protocol for a scoping review | 10.1136/bmjopen-2022-070329 | Review paper |  |  |  |  |
| Psychometric properties of the Patient Health Questionnaire-4 among Hong Kong young adults in 2021: Associations with meaning in life and suicidal ideation | 10.3389/fpsyt.2023.1138755 |  | Not at all relevant |  |  |  |
| Green Heart Louisville: intra-urban, hyperlocal land-use regression modeling of nitrogen oxides and ozone | 10.1101/2023.03.03.23286765 |  | Not at all relevant |  |  |  |
| The Building Emotional Awareness and Mental health (BEAM) program developed with a community partner for mothers of infants: protocol for a feasibility randomized controlled trial | 10.1186/s40814-023-01260-y |  | Not at all relevant |  |  |  |
| Differences in Center for Epidemiologic Studies Depression Scale, Generalized Anxiety Disorder-7 and Kessler Screening Scale for Psychological Distress Scores between Smartphone Version versus Paper Version Administration: Evidence of Equivalence | 10.3390/ijerph20064773 |  | Not at all relevant |  |  |  |
| Long-term Pu-erh tea consumption improves blue light-induced depression-like behaviors | 10.1039/d2fo02780a |  | Not at all relevant |  |  |  |
| Trait Mindfulness and Problematic Smartphone Use in Chinese Early Adolescent: The Multiple Mediating Roles of Negative Affectivity and Fear of Missing Out | 10.3390/bs13030222 |  | Not at all relevant |  |  |  |
| A Relational Agent Intervention for Adolescents Seeking Mental Health Treatment: Protocol for a Randomized Controlled Trial | 10.2196/44940 |  | Not at all relevant |  |  |  |
| Real-time Symptom Assessment in Patients With Endometriosis: Psychometric Evaluation of an Electronic Patient-Reported Outcome Measure, Based on the Experience Sampling Method | 10.2196/29480 |  | Not at all relevant |  |  |  |
| Digital Phenotyping Models of Symptom Improvement in College Mental Health: Generalizability Across Two Cohorts | 10.1007/s41347-023-00310-9 |  | Not at all relevant |  |  |  |
| mGluR-dependent plasticity in rodent models of Alzheimer's disease | 10.3389/fnsyn.2023.1123294 |  | Not at all relevant |  |  |  |
| Multi-layered e-feedback anxiety: An action research study among Chinese learners using peer feedback activities in an academic writing course | 10.3389/fpsyg.2023.1062517 |  | Not at all relevant |  |  |  |
| Positive Psychological Intervention Effects on Depression: Positive Emotion Does Not Mediate Intervention Impact in a Sample with Elevated Depressive Symptoms | 10.1007/s42761-022-00140-7 |  | Not at all relevant |  |  |  |
| A chatbot-based intervention with ELME to improve stress and health-related parameters in a stressed sample: Study protocol of a randomised controlled trial | 10.3389/fdgth.2023.1046202 |  | Not at all relevant |  |  |  |
| Assessment of App Store Description and Privacy Policy to Explore Ethical and Safety Concerns Associated with the Use of Mental Health Apps for Depression | 10.1177/02537176221142046 |  | Not at all relevant |  |  |  |
| Digital technologies for mental health improvements in the COVID-19 pandemic: a scoping review | 10.1186/s12889-023-15302-w | Review paper |  |  |  |  |
| Association between seropositivity for toxocariasis and cognitive functioning in older adults: an analysis of cross-sectional data from the US National Health and Nutrition Examination Survey (NHANES), 2011-2014 | 10.1136/bmjopen-2022-068974 |  | Not at all relevant |  |  |  |
| Evaluation of a smartphone application for self-help for patients with social anxiety disorder: a randomized controlled study-SMASH | 10.1186/s13063-023-07168-5 |  | Not at all relevant |  |  |  |
| Use of Mobile Apps & Stepped-Care Model for Treating Depression in Primary Care | 10.1016/j.pop.2022.10.005 |  | Not at all relevant |  |  |  |
| Pilot feasibility trial of a brief mobile-augmented suicide prevention intervention for serious mental illness | 10.1037/prj0000547 |  | Not at all relevant |  |  |  |
| Lifetime history of an anxiety or depression disorder and adherence to medications used for the acute treatment of migraine | 10.1111/head.14477 |  | Not at all relevant |  |  |  |
| [End of life and cancer: Understanding the ethical issues of voluntary assisted dying] | 10.1016/j.bulcan.2023.01.005 |  | Not at all relevant |  |  |  |
| The Power and Promise of Postpartum Self Care: Evaluation of a Web-Based Tool for Underserved Women | 10.1007/s10995-023-03605-8 |  | Not at all relevant |  |  |  |
| Enhancing Internet-based psychotherapy for adults with emotional disorders using ecological momentary assessments and interventions: Study protocol of a feasibility trial with "My EMI, Emotional Well-being" app | 10.1016/j.invent.2023.100601 |  | Not at all relevant |  |  |  |
| A therapist-guided internet-based cognitive behavioral therapy program for Hong Kong university students with psychological distress: A study protocol for a randomized controlled trial | 10.1016/j.invent.2022.100600 |  | Not at all relevant |  |  |  |
| A pilot randomized controlled trial of a cognitive-behavioral therapy guided self-help mobile app for the post-acute treatment of anorexia nervosa: A registered report | 10.1002/eat.23891 |  | Not at all relevant |  |  |  |
| Relationship between daily rated depression symptom severity and the retrospective self-report on PHQ-9: A prospective ecological momentary assessment study on 80 psychiatric outpatients | 10.1016/j.jad.2022.12.127 |  | Not at all relevant |  |  |  |
| Rapid determination of polyethoxylated tallow amine surfactants in human plasma by LC-MSMS | 10.1016/j.talanta.2022.124115 |  | Not at all relevant |  |  |  |
| Update on treatments for anxiety-related disorders | 10.1097/YCO.0000000000000841 |  | Not at all relevant |  |  |  |
| A Pilot Study of a Three-Session Heart Rate Variability Biofeedback Intervention for Veterans with Posttraumatic Stress Disorder | 10.1007/s10484-022-09565-z |  | Not at all relevant |  |  |  |
| Tactile stimulation improves cognition, motor, and anxiety-like behaviors and attenuates the Alzheimer's disease pathology in adult APP(NL-G-F/NL-G-F) mice | 10.1002/syn.22257 |  | Not at all relevant |  |  |  |
| Age-specific and family-centered information modalities to prepare children at home for day-care surgery | 10.1016/j.jpedsurg.2022.08.023 |  | Not at all relevant |  |  |  |
| Effect of mHealth plus occupational therapy on cognitive function, mood and physical function in people after cancer: Secondary analysis of a randomized controlled trial | 10.1016/j.rehab.2022.101681 |  | Not at all relevant |  |  |  |
| Finding the "sweet spot" of smartphone use: Reduction or abstinence to increase well-being and healthy lifestyle?! An experimental intervention study | 10.1037/xap0000430 |  | Not at all relevant |  |  |  |
| Awake bruxism frequency and psychosocial factors in college preparatory students | 10.1080/08869634.2020.1829289 |  | Not at all relevant |  |  |  |
| Understanding the inclination of South Indian nursing graduates in using mobile learning applications | 10.4103/jehp.jehp_1082_22 |  | Not at all relevant |  |  |  |
| Digital Behavioral Activation Interventions During the Perinatal Period: Scoping Review | 10.2196/40937 | Review paper |  |  |  |  |
| Applying Human-Centered Design in Global Mental Health to Improve Reach Among Underserved Populations in the United States and India | 10.9745/GHSP-D-22-00312 |  | Not at all relevant |  |  |  |
| Development of an mHealth App to Support the Prevention of Sexually Transmitted Infections Among Black Men Who Have Sex With Men Engaged in Pre-exposure Prophylaxis Care in New Orleans, Louisiana: Qualitative User-Centered Design Study | 10.2196/43019 |  | Not at all relevant |  |  |  |
| Four 2×2 factorial trials of smartphone CBT to reduce subthreshold depression and to prevent new depressive episodes among adults in the community-RESiLIENT trial (Resilience Enhancement with Smartphone in LIving ENvironmenTs): a master protocol | 10.1136/bmjopen-2022-067850 |  | Not at all relevant |  |  |  |
| Effects of Fetal Images Produced in Virtual Reality on Maternal-Fetal Attachment: Randomized Controlled Trial | 10.2196/43634 |  | Not at all relevant |  |  |  |
| Can healthcare apps and smart speakers improve the health behavior and depression of older adults? A quasi-experimental study | 10.3389/fdgth.2023.1117280 |  | Not at all relevant |  |  |  |
| Effect of receiving mobile text messages on cortisol concentrations in students at the University of the Free State | 10.4102/hsag.v28i0.2064 |  | Not at all relevant |  |  |  |
| Using natural language from a smartphone pregnancy app to identify maternal depression | 10.21203/rs.3.rs-2583296/v1 |  | Not at all relevant |  |  |  |
| Cluster-based psychological phenotyping and differences in anxiety treatment outcomes | 10.1038/s41598-023-28660-7 |  | Not at all relevant |  |  |  |
| Using a Safety Planning Mobile App to Address Suicidality in Young People Attending Community Mental Health Services in Ireland: Protocol for a Pilot Randomized Controlled Trial | 10.2196/44205 |  | Not at all relevant |  |  |  |
| Building Emotional Awareness and Mental Health (BEAM): an open-pilot and feasibility study of a digital mental health and parenting intervention for mothers of infants | 10.1186/s40814-023-01245-x |  | Not at all relevant |  |  |  |
| Long-term participant retention and engagement patterns in an app and wearable-based multinational remote digital depression study | 10.1038/s41746-023-00749-3 |  | Not at all relevant |  |  |  |
| Testing the Impact of the #chatsafe Intervention on Young People's Ability to Communicate Safely About Suicide on Social Media: Protocol for a Randomized Controlled Trial | 10.2196/44300 |  | Not at all relevant |  |  |  |
| Health-related quality of life among Indian population: The EQ-5D population norms for India | 10.7189/jogh.13.04018 |  | Not at all relevant |  |  |  |
| Cognitive bias modification for interpretation (CBM-I) for post-traumatic stress disorder: study protocol of an app-based randomised controlled trial | 10.1136/bmjopen-2022-069228 |  | Not at all relevant |  |  |  |
| Suicidal ideation in the general population in China after the COVID-19 pandemic was initially controlled | 10.1016/j.jad.2022.12.036 |  | Not at all relevant |  |  |  |
| How to use participatory design to develop an eHealth intervention to reduce preprocedural stress and anxiety among children visiting the hospital: The Hospital Hero app multi-study and pilot report | 10.3389/fped.2023.1132639 |  | Not at all relevant |  |  |  |
| A Digital Single-Session Intervention Platform for Youth Mental Health: Cultural Adaptation, Evaluation, and Dissemination | 10.2196/43062 |  | Not at all relevant |  |  |  |
| Internet and depression in adolescents: Evidence from China | 10.3389/fpsyg.2023.1026920 |  | Not at all relevant |  |  |  |
| The Usability, Feasibility, Acceptability, and Efficacy of Digital Mental Health Services in the COVID-19 Pandemic: Scoping Review, Systematic Review, and Meta-analysis | 10.2196/43730 | Review paper |  |  |  |  |
| Depression-related phenotypes at early stages of Aβ and tau accumulation in inducible Alzheimer's disease mouse model: Task-oriented and concept-driven interpretations | 10.1016/j.bbr.2022.114187 |  | Not at all relevant |  |  |  |
| Selective orexin 1 receptor antagonist SB-334867 aggravated cognitive dysfunction in 3xTg-AD mice | 10.1016/j.bbr.2022.114171 |  | Not at all relevant |  |  |  |
| A Three-Armed Randomized Controlled Trial to Evaluate the Effectiveness, Acceptance, and Negative Effects of StudiCare Mindfulness, an Internet- and Mobile-Based Intervention for College Students with No and "On Demand" Guidance | 10.3390/ijerph20043208 |  | Not at all relevant |  |  |  |
| Design, development and randomised controlled trial protocol of a smartphone-delivered version of 'SOLAR' for emergency service workers to manage stress and trauma | 10.1136/bmjopen-2022-062710 |  | Not at all relevant |  |  |  |
| Effectiveness of Digital Guided Self-help Mindfulness Training During Pregnancy on Maternal Psychological Distress and Infant Neuropsychological Development: Randomized Controlled Trial | 10.2196/41298 |  | Not at all relevant |  |  |  |
| Smartphone Psychotherapy Reduces Fear of Cancer Recurrence Among Breast Cancer Survivors: A Fully Decentralized Randomized Controlled Clinical Trial (J-SUPPORT 1703 Study) | 10.1200/JCO.22.00699 |  | Not at all relevant |  |  |  |
| Virtual reality exposure therapy for reducing social anxiety in stuttering: A randomized controlled pilot trial | 10.3389/fdgth.2023.1061323 |  | Not at all relevant |  |  |  |
| Engaging in physical activity instead of (over)using the smartphone: An experimental investigation of lifestyle interventions to prevent problematic smartphone use and to promote mental health | 10.1007/s10389-023-01832-5 |  | Not at all relevant |  |  |  |
| Mindfulness-based stress reduction for community-dwelling older adults with subjective cognitive decline (SCD) and mild cognitive impairment (MCI) in primary care: a mixed-methods feasibility randomized control trial | 10.1186/s12875-023-02002-y |  | Not at all relevant |  |  |  |
| Association of the awareness of the epidemic, mental health status with mobile phone screen use time in Chinese college students during COVID-19 isolation and control | 10.1371/journal.pgph.0001259 |  | Not at all relevant |  |  |  |
| Nomophobia and Self-Esteem: A Cross Sectional Study in Greek University Students | 10.3390/ijerph20042929 |  | Not at all relevant |  |  |  |
| Systematic combinations of major cannabinoid and terpene contents in Cannabis flower and patient outcomes: a proof-of-concept assessment of the Vigil Index of Cannabis Chemovars | 10.1186/s42238-022-00170-9 |  | Not at all relevant |  |  |  |
| Mobile Mental Health in Women's Community-Based Organizations: Protocol for a Pilot Randomized Controlled Trial | 10.2196/42919 |  | Not at all relevant |  |  |  |
| Mimicking Pain-Perceptual Sensitization and Pattern Recognition Based on Capacitance- and Conductance-Regulated Neuroplasticity in Neural Network | 10.1021/acsami.2c20297 |  | Not at all relevant |  |  |  |
| Association between Problematic Use of Smartphones and Mental Health in the Middle East and North Africa (MENA) Region: A Systematic Review | 10.3390/ijerph20042891 | Review paper |  |  |  |  |
| Mental, neurological and substance use disorders among the Latino migrant population in the United States who visited the Health Windows and Mobile Health Units in 2021 | 10.3389/fpubh.2023.959535 |  | Not at all relevant |  |  |  |
| Osteocalcin ameliorates cognitive dysfunctions in a mouse model of Alzheimer's Disease by reducing amyloid β burden and upregulating glycolysis in neuroglia | 10.1038/s41420-023-01343-y |  | Not at all relevant |  |  |  |
| Modeling the impact of combined use of COVID Alert SA app and vaccination to curb COVID-19 infections in South Africa | 10.1371/journal.pone.0264863 |  | Not at all relevant |  |  |  |
| Effectiveness of Self-guided Tailored Implementation Strategies in Integrating and Embedding Internet-Based Cognitive Behavioral Therapy in Routine Mental Health Care: Results of a Multicenter Stepped-Wedge Cluster Randomized Trial | 10.2196/41532 |  | Not at all relevant |  |  |  |
| Factors Associated with Telehealth Utilization among Older African Americans in South Los Angeles during the COVID-19 Pandemic | 10.3390/ijerph20032675 |  | Not at all relevant |  |  |  |
| A pilot randomised controlled trial of the Peer Tree digital intervention targeting loneliness in young people: a study protocol | 10.1186/s13063-022-07029-7 |  | Not at all relevant |  |  |  |
| The Use of Sensors to Detect Anxiety for In-the-Moment Intervention: Scoping Review | 10.2196/42611 | Review paper |  |  |  |  |
| Increasing the value of digital phenotyping through reducing missingness: a retrospective review and analysis of prior studies | 10.1136/bmjment-2023-300718 | Review paper |  |  |  |  |
| [Effect of Digital Health Interventions on Psychotic Symptoms among Persons with Severe Mental Illness in Community: A Systematic Review and Meta-Analysis] | 10.4040/jkan.22121 | Review paper |  |  |  |  |
| A longitudinal assessment of posttraumatic stress symptoms and pain catastrophizing after injury | 10.1037/rep0000481 |  | Not at all relevant |  |  |  |
| Recurrence of depression can be foreseen by monitoring mental states with statistical process control | 10.1037/abn0000812 |  | Not at all relevant |  |  |  |
| Community Resilience After Hurricanes: Can Neuman's Systems Theory Guide Public Health Nursing? | 10.1891/RTNP-2022-0029 |  | Not at all relevant |  |  |  |
| Prediction of Suicide Attempts and Suicide-Related Events Among Adolescents Seen in Emergency Departments | 10.1001/jamanetworkopen.2022.55986 |  | Not at all relevant |  |  |  |
| Does Using a Mindfulness App Reduce Anxiety and Worry? A Randomized-Controlled Trial | 10.1891/JCPSY-D-20-00058 |  | Not at all relevant |  |  |  |
| A Novel Approach to Clustering Accelerometer Data for Application in Passive Predictions of Changes in Depression Severity | 10.3390/s23031585 |  | Not at all relevant |  |  |  |
| Mental health and psychosocial problems among laboratory technicians in response to the COVID-19 pandemic in Hebei, China | 10.1016/j.heliyon.2023.e13090 |  | Not at all relevant |  |  |  |
| Differential methylation of circRNA m6A in an APP/PS1 Alzheimer's disease mouse model | 10.3892/mmr.2023.12942 |  | Not at all relevant |  |  |  |
| A nurse-led mHealth intervention to alleviate depressive symptoms in older adults living alone in the community: A quasi-experimental study | 10.1016/j.ijnurstu.2022.104431 |  | Not at all relevant |  |  |  |
| Computational models of subjective feelings in psychiatry | 10.1016/j.neubiorev.2022.105008 |  | Not at all relevant |  |  |  |
| Effects of a Smartphone-Based Self-management Intervention for Individuals With Bipolar Disorder on Relapse, Symptom Burden, and Quality of Life: A Randomized Clinical Trial | 10.1001/jamapsychiatry.2022.4304 |  | Not at all relevant |  |  |  |
| Prognostic factors and effect modifiers for personalisation of internet-based cognitive behavioural therapy among university students with subthreshold depression: A secondary analysis of a factorial trial | 10.1016/j.jad.2022.11.024 |  | Not at all relevant |  |  |  |
| The Effects of APP-Based Intervention for Depression Among Community-Dwelling Individuals With Spinal Cord Injury: A Randomized Controlled Trial | 10.1016/j.apmr.2022.10.005 |  | Not at all relevant |  |  |  |
| Leveraging smartphones to observe couples remotely and illuminate how COVID-19 stress shaped marital communication | 10.1037/fam0001035 |  | Not at all relevant |  |  |  |
| Attachment, loneliness, and social connection as prospective predictors of suicidal ideation during the COVID-19 pandemic: A relational diathesis-stress experience sampling study | 10.1111/sltb.12922 |  | Not at all relevant |  |  |  |
| Do Americans Lacking a Local Orthopaedic Surgeon Have Adequate Broadband for Telehealth? | 10.1097/CORR.0000000000002374 |  | Not at all relevant |  |  |  |
| Creating Comprehensive Crisis Response Systems: An Opportunity to Build on The Promise of 988 | 10.1007/s10597-022-01017-6 |  | Not at all relevant |  |  |  |
| Evidence for distinct genetic and environmental influences on fear acquisition and extinction | 10.1017/S0033291721002580 |  | Not at all relevant |  |  |  |
| Examine the associations between smartphone hookup application uses and sexual health and relationship outcomes among college students | 10.1080/07448481.2021.1898406 |  | Not at all relevant |  |  |  |
| Patient-reported burden of myasthenia gravis: baseline results of the international prospective, observational, longitudinal real-world digital study MyRealWorld-MG | 10.1136/bmjopen-2022-066445 |  | Not at all relevant |  |  |  |
| Exploring Caregivers' Perspectives and Perceived Acceptability of a Mobile-Based Telemonitoring Program to Support Pregnant Women at High-Risk for Preeclampsia in Karachi, Pakistan: A Qualitative Descriptive Study | 10.3390/healthcare11030392 |  | Not at all relevant |  |  |  |
| An Effective Test (EOmciSS) for Screening Older Adults With Mild Cognitive Impairment in a Community Setting: Development and Validation Study | 10.2196/40858 |  | Not at all relevant |  |  |  |
| A pilot intervention of using a mobile health app (ONC Roadmap) to enhance health-related quality of life in family caregivers of pediatric patients with cancer | 10.21037/mhealth-22-24 |  | Not at all relevant |  |  |  |
| Effects of a Smartphone-Based, Multisession Interpretation-Bias Modification for Anxiety: Positive Intervention Effects and Low Attrition | 10.3390/ijerph20032270 |  |  | Screening, no treatment |  |  |
| ReachCare Mobile Apps for Patients Experiencing Suicidality in the Emergency Department: Development and Usability Testing Using Mixed Methods | 10.2196/41422 |  |  | App usability |  |  |
| P4 Medicine for Heterogeneity of Dry Eye: A Mobile Health-based Digital Cohort Study | 10.14789/jmj.JMJ22-0032-R |  | Not at all relevant |  |  |  |
| Examining young adults daily perspectives on usage of anxiety apps: A user study | 10.1371/journal.pdig.0000185 |  | Not at all relevant |  |  |  |
| Understanding the Subjective Experience of Long-term Remote Measurement Technology Use for Symptom Tracking in People With Depression: Multisite Longitudinal Qualitative Analysis | 10.2196/39479 |  | Not at all relevant |  |  |  |
| Understanding Digital Mental Health Needs and Usage With an Artificial Intelligence-Led Mental Health App (Wysa) During the COVID-19 Pandemic: Retrospective Analysis | 10.2196/41913 |  | Not at all relevant |  |  |  |
| Feasibility and acceptability of perioperative application of biofeedback-based virtual reality versus active control for pain and anxiety in children and adolescents undergoing surgery: protocol for a pilot randomised controlled trial | 10.1136/bmjopen-2022-071274 |  | Not at all relevant |  |  |  |
| Automatic Depression Detection Using Smartphone-Based Text-Dependent Speech Signals: Deep Convolutional Neural Network Approach | 10.2196/34474 |  | Not at all relevant |  |  |  |
| Effect of mobile application types on stroke rehabilitation: a systematic review | 10.1186/s12984-023-01124-9 | Review paper |  |  |  |  |
| The Feasibility of Implementing Remote Measurement Technologies in Psychological Treatment for Depression: Mixed Methods Study on Engagement | 10.2196/42866 |  | Not at all relevant |  |  |  |
| Comparing Smartphone Virtual Reality Exposure Preparation to Care as Usual in Children Aged 6 to 14 Years Undergoing Magnetic Resonance Imaging: Protocol for a Multicenter, Observer-Blinded, Randomized Controlled Trial | 10.2196/41080 |  | Not at all relevant |  |  |  |
| Mobile opportunity against stress: Open study protocol on the effectiveness of a mobile platform for stress self-management in the post-pandemic era | 10.3389/fpsyg.2022.917574 |  | Not at all relevant |  |  |  |
| Patients' Information Needs Related to a Monitoring Implant for Heart Failure: Co-designed Study Based on Affect Stories | 10.2196/38096 |  | Not at all relevant |  |  |  |
| Telehealth multicomponent exercise and health education in breast cancer patients undergoing primary treatment: rationale and methodological protocol for a randomized clinical trial (ABRACE: Telehealth) | 10.1186/s13063-022-07015-z |  | Not at all relevant |  |  |  |
| Within and between-day variation and associations of symptoms in Long Covid: Intensive longitudinal study | 10.1371/journal.pone.0280343 |  | Not at all relevant |  |  |  |
| Gait Characteristics Associated with Fear of Falling in Hospitalized People with Parkinson's Disease | 10.3390/s23031111 |  | Not at all relevant |  |  |  |
| 'Putting all my eggs into the app': Self, relational and systemic surveillance of mothers' use of digital technologies during the transition to parenting | 10.1177/20552076221150742 |  | Not at all relevant |  |  |  |
| Counseling for Health: How Psychological Distance Influences Continuance Intention towards Mobile Medical Consultation | 10.3390/ijerph20031718 |  | Not at all relevant |  |  |  |
| Gender differences in dry eye disease symptoms associated with psychological health indicators among adults using mobile mental health apps | 10.1371/journal.pone.0278921 |  | Not at all relevant |  |  |  |
| A Chilean survey of perinatal women and health care professionals' views towards perinatal apps | 10.21037/mhealth-22-37 |  | Not at all relevant |  |  |  |
| A web app-based music intervention reduces experimental thermal pain: A randomized trial on preferred versus least-liked music style | 10.3389/fpain.2022.1055259 |  | Not at all relevant |  |  |  |
| Use of Mobile Technology to Identify Behavioral Mechanisms Linked to Mental Health Outcomes in Kenya: Protocol for Development and Validation of a Predictive Model | 10.21203/rs.3.rs-2458763/v1 |  | Not at all relevant |  |  |  |
| Analysis of Mobile App-Based Mental Health Solutions for College Students: A Rapid Review | 10.3390/healthcare11020272 | Review paper |  |  |  |  |
| The Use of Technology to Provide Mental Health Services to Youth Experiencing Homelessness: Scoping Review | 10.2196/41939 | Review paper |  |  |  |  |
| Evaluating the Effectiveness of the Supportive Parenting App on Parental Outcomes: Randomized Controlled Trial | 10.2196/41859 |  | Not at all relevant |  |  |  |
| A randomized controlled trial of a 14-day mindfulness ecological momentary intervention (MEMI) for generalized anxiety disorder | 10.1192/j.eurpsy.2023.2 |  | Not at all relevant |  |  |  |
| Improvements in Glycemic Control and Depressive Symptoms Among Adults With Type 2 Diabetes: Retrospective Study | 10.2196/41880 |  | Not at all relevant |  |  |  |
| Use of WhatsApp by older adults screened for depression in socioeconomically deprived areas of Guarulhos, São Paulo State, Brazil: challenges and possibilities for telehealth | 10.1590/0102-311XEN093422 |  | Not at all relevant |  |  |  |
| A Systematic Review of Mobile Apps as an Adjunct to Psychological Interventions for Emotion Dysregulation | 10.3390/ijerph20021431 | Review paper |  |  |  |  |
| Training Staff Across the Veterans Affairs Health Care System to Use Mobile Mental Health Apps: A National Quality Improvement Project | 10.2196/41773 |  | Not at all relevant |  |  |  |
| A Real-Time Mobile Intervention to Reduce Sedentary Behavior Before and After Cancer Surgery: Pilot Randomized Controlled Trial | 10.2196/41425 |  | Not at all relevant |  |  |  |
| Randomized Controlled Trial of a Mindfulness Mobile Application for Ruminative Adolescents | 10.1080/15374416.2022.2158840 |  | Not at all relevant |  |  |  |
| Ratings and experiences in using a mobile application to increase physical activity among university students: implications for future design | 10.1007/s10209-022-00962-z |  | Not at all relevant |  |  |  |
| The PTSD Family Coach App in Veteran Family Members: Pilot Randomized Controlled Trial | 10.2196/42053 |  | Not at all relevant |  |  |  |
| Assessment and Disruption of Ruminative Episodes to Enhance Mobile Cognitive Behavioral Therapy Just-in-Time Adaptive Interventions in Clinical Depression: Pilot Randomized Controlled Trial | 10.2196/37270 |  | Not at all relevant |  |  |  |
| How does centralized isolation treatment strategy affect the medical staff's mental health during the COVID-19 pandemic? | 10.3389/fpubh.2022.1055564 |  | Not at all relevant |  |  |  |
| Virtual Reality Exercise Program Effects on Body Mass Index, Depression, Exercise Fun and Exercise Immersion in Overweight Middle-Aged Women: A Randomized Controlled Trial | 10.3390/ijerph20020900 |  | Not at all relevant |  |  |  |
| Association of Neural Connectome With Early Experiences of Abuse in Adults | 10.1001/jamanetworkopen.2022.53082 |  | Not at all relevant |  |  |  |
| The Role of Prenatal Psychosocial Stress in the Associations of a Proinflammatory Diet in Pregnancy With Child Adiposity and Growth Trajectories | 10.1001/jamanetworkopen.2022.51367 |  | Not at all relevant |  |  |  |
| Effects of Mobile Mindfulness Meditation on the Mental Health of University Students: Systematic Review and Meta-analysis | 10.2196/39128 | Review paper |  |  |  |  |
| Technology in psychology: a bibliometric analysis of technology-based interventions in clinical and health psychology | 10.1080/17538157.2022.2054343 |  | Not at all relevant |  |  |  |
| Skin Conductance Reactivity as a Predictor of Stroke-induced Posttraumatic Stress Disorder Symptoms: A Dimensional Approach | 10.1155/2023/6671337 |  | Not at all relevant |  |  |  |
| Self-esteem only goes so far: the moderating effect of social media screen time on self-esteem and depressive symptoms | 10.1080/0144929x.2022.2139759 |  | Not at all relevant |  |  |  |
| Investigation of adolescents and their mothers in terms of nomophobia | 10.24953/turkjped.2023.350 |  | Not at all relevant |  |  |  |
| False Atrial Fibrillation Alerts from Smartwatches are Associated with Decreased Perceived Physical Well-being and Confidence in Chronic Symptoms Management | 10.26502/fccm.92920314 |  | Not at all relevant |  |  |  |
| Effects of Single-Session Meditation on Aortic Pulsatility and Anxiety in Mildly to Moderately Anxious Adults |  |  | Not at all relevant |  |  |  |
| Prehospital Treatment of Benzodiazepine-Resistant Pediatric Status Epilepticus with Parenteral Ketamine: A Case Series | 10.1080/10903127.2023.2221967 |  | Not at all relevant |  |  |  |
| The impact of mindfulness-based stress reduction on psychological health among patients with chronic diseases during COVID-19 outbreak lockdown | 10.1186/s43088-023-00389-2 |  | Not at all relevant |  |  |  |
| APP/PS1 Gene-Environmental Cadmium Interaction Aggravates the Progression of Alzheimer's Disease in Mice via the Blood-Brain Barrier, Amyloid-β, and Inflammation | 10.3233/JAD-221205 |  | Not at all relevant |  |  |  |
| The Impact of Virtual Reality (VR) on Psychological and Physiological Variables in Children Receiving Chemotherapy: A Pilot Cross-Over Study | 10.1177/15347354231168984 |  | Not at all relevant |  |  |  |
| Efficacy of a Mobile-Based Multidomain Intervention to Improve Cognitive Function and Health-Related Outcomes Among Older Korean Adults with Subjective Cognitive Decline | 10.3233/JAD-221299 |  | Not at all relevant |  |  |  |
| Sequence Modeling of Passive Sensing Data for Treatment Response Prediction in Major Depressive Disorder | 10.1109/TNSRE.2023.3260301 |  | Not at all relevant |  |  |  |
| The effect of a game-based mobile app 'MyHeartMate' to promote lifestyle change in coronary disease patients: a randomized controlled trial | 10.1093/ehjdh/ztac069 |  | Not at all relevant |  |  |  |
| Wearable devices for anxiety & depression: A scoping review | 10.1016/j.cmpbup.2023.100095 | Review paper |  |  |  |  |
| Mental Health Problems During COVID-19 and Attitudes Toward Digital Therapeutics | 10.30773/pi.2022.0150 |  | Not at all relevant |  |  |  |
| Systematic Review of Online Interventions to Reduce Perinatal Mood and Anxiety Disorders in Underserved Populations | 10.1097/JPN.0000000000000658 | Review paper |  |  |  |  |
| Feasibility of Problem-Solving Training During Inpatient Rehabilitation in Patients With Stroke | 10.5014/ajot.2023.050083 |  | Not at all relevant |  |  |  |
| Developing a Multimodal Monitoring System for Geriatric Depression: A Feasibility Study | 10.1097/CIN.0000000000000925 |  | Not at all relevant |  |  |  |
| Intolerance of aloneness as a prospective predictor of suicidal ideation during COVID-19 | 10.1016/j.jadr.2023.100469 |  | Not at all relevant |  |  |  |
| Effects of a community gardening intervention on diet, physical activity, and anthropometry outcomes in the USA (CAPS): an observer-blind, randomised controlled trial | 10.1016/S2542-5196(22)00303-5 |  | Not at all relevant |  |  |  |
| Comparing online cognitive load on mobile versus PC-based devices | 10.1007/s00779-022-01707-8 |  | Not at all relevant |  |  |  |
| Effects of a mobile health intervention on activities of stress self-management for workers | 10.3233/WOR-211406 |  | Not at all relevant |  |  |  |
| Information and Communication Technology Use for Alleviation of Disability Onset in Socially Isolated Older Adults: A Longitudinal Cohort Study | 10.1159/000528134 |  | Not at all relevant |  |  |  |
| Emotion detection for supporting depression screening | 10.1007/s11042-022-14290-0 |  | Not at all relevant |  |  |  |
| Real-time digital monitoring of a suicide attempt by a hospital patient | 10.1016/j.genhosppsych.2022.12.005 |  | Not at all relevant |  |  |  |
| Reducing contrast avoidance in GAD by savoring positive emotions: Outcome and mediation in a randomized controlled trial | 10.1016/j.janxdis.2022.102659 |  | Not at all relevant |  |  |  |
| Smartphone app engagement and clinical outcomes in a hybrid clinic | 10.1016/j.psychres.2022.115015 |  | Not at all relevant |  |  |  |
| Improving screening for major depressive disorder | 10.1097/JXX.0000000000000817 |  |  | No Mobile app |  |  |
| Hybrid-delivered cognitive behavioral symptom management and activity coaching intervention for patients undergoing hematopoietic stem cell transplant: Findings from intervention development and a pilot randomized trial | 10.1080/07347332.2022.2152519 |  | Not at all relevant |  |  |  |
| Needs and Demands for e-Mental Health Interventions in Individuals with Overweight and Obesity: User-Centred Design Approach | 10.1159/000527914 |  | Not at all relevant |  |  |  |
| The Impact of Smartphone Use on Course Comprehension and Psychological Well-Being in the College Classroom | 10.1007/s10755-022-09638-1 |  | Not at all relevant |  |  |  |
| Intranasal interferon-beta alleviates anxiety and depressive-like behaviors by modulating microglia polarization in an Alzheimer's disease model | 10.1016/j.neulet.2022.136968 |  | Not at all relevant |  |  |  |
| Descriptive Exploratory Study to Understand Postpartum Anxiety Using Multiple Measures | 10.1016/j.jogn.2022.09.003 |  | Not at all relevant |  |  |  |
| Redefining Disability: Patient-Reported Outcome Measures After Minor Stroke and Transient Ischemic Attack | 10.1161/STROKEAHA.122.040409 |  | Not at all relevant |  |  |  |
| Telemedicine for neurological diseases: A systematic review and meta-analysis | 10.1111/ene.15599 | Review paper |  |  |  |  |
| Barriers and facilitators to technology-enhanced measurement based care for depression among Canadian clinicians and patients: Results of an online survey | 10.1016/j.jad.2022.09.055 |  | Not at all relevant |  |  |  |
| Prevalence and Correlates of Embitterment in a National Representative Sample | 10.1159/000526457 |  | Not at all relevant |  |  |  |
| Care4AD: A Technology-Driven Platform for Care Coordination and Management: Acceptability Study in Dementia | 10.1159/000526219 |  | Not at all relevant |  |  |  |
| Augmented reality with algorithm animation and their effect on students' emotions | 10.1007/s11042-022-13679-1 |  | Not at all relevant |  |  |  |
| The Effects of Mobile Technology on Learning Performance and Motivation in Mathematics Education | 10.1007/s10639-022-11166-6 |  | Not at all relevant |  |  |  |
| Examining the temporal dynamics of anxiety and depressive symptoms during a therapist-supported, smartphone-based intervention for depression: Longitudinal observational study | 10.1002/jclp.23401 |  | Not at all relevant |  |  |  |
| Responding to Acute Mental Health Crises in Black Youth: Is It Safe to Call 911? | 10.1007/s10597-022-00980-4 |  | Not at all relevant |  |  |  |
| A novel multi-modal depression detection approach based on mobile crowd sensing and task-based mechanisms | 10.1007/s11042-022-12315-2 |  | Not at all relevant |  |  |  |
| A pilot study for a smartphone app for the prevention of depression in non-professional caregivers | 10.1080/13607863.2022.2056878 |  | Not at all relevant |  |  |  |
| Assessing anxiety-linked impairment in attentional control without eye-tracking: The masked-target antisaccade task | 10.3758/s13428-022-01800-z |  | Not at all relevant |  |  |  |
| Speech as a Biomarker for Depression | 10.2174/1871527320666211213125847 |  | Not at all relevant |  |  |  |
| Efficient on-site confirmatory testing for atrial fibrillation with derived 12-lead ECG in a wireless body area network | 10.1007/s12652-021-03543-9 |  | Not at all relevant |  |  |  |
| Mindfulness-based emotional regulation for patients with implantable cardioverter-defibrillators: A randomized pilot study of efficacy, applicability, and safety | 10.5603/CJ.a2021.0094 |  | Not at all relevant |  |  |  |
| Implementing a Suicide Audit in Montreal: Taking Suicide Review Further to Make Concrete Recommendations for Suicide Prevention | 10.1080/13811118.2021.1965058 | Review paper |  |  |  |  |
| The effects of app-based mindfulness practice on the well-being of university students and staff | 10.1007/s12144-021-01762-z |  |  |  |  | 1 |
| An online observational study assessing clinical characteristics and impacts of the COVID-19 pandemic on mental health: a perspective study from Bangladesh | 10.1007/s10389-020-01445-2 |  | Not at all relevant |  |  |  |
| An Augmented Reality (AR) App Enhances the Pulmonary Function and Potency/Feasibility of Perioperative Rehabilitation in Patients Undergoing Orthopedic Surgery | 10.3390/ijerph20010648 |  | Not at all relevant |  |  |  |
| Telemedicine as a Tool to Improve Medicine Adherence in Patients with Affective Disorders - A Systematic Literature Review | 10.2147/PPA.S388106 | Review paper |  |  |  |  |
| Digital delivery of behavioural activation therapy to overcome depression and facilitate social and economic transitions of adolescents in South Africa (the DoBAt study): protocol for a pilot randomised controlled trial | 10.1136/bmjopen-2022-065977 |  | Not at all relevant |  |  |  |
| Patient and Therapist Expectations for a Blended Cognitive Behavioral Therapy Program for Depression: Qualitative Exploratory Study | 10.2196/36806 |  | Not at all relevant |  |  |  |
| Intergenerational Social Mobility and Health in Later Life: Diagonal Reference Models Applied to the Lothian Birth Cohort 1936 | 10.1093/geronb/gbac107 |  | Not at all relevant |  |  |  |
| Evaluation of accuracy of photogrammetry with 3D scanning and conventional impression method for craniomaxillofacial defects using a software analysis | 10.1186/s13063-022-07005-1 |  | Not at all relevant |  |  |  |
| Pilot randomised controlled trial of a remotely delivered online intervention for adolescent mental health problems in India: lessons learned about low acceptability and feasibility during the COVID-19 pandemic | 10.1192/bjo.2022.624 |  | Not at all relevant |  |  |  |
| Factors associated with long-term use of digital devices in the electronic Framingham Heart Study | 10.1038/s41746-022-00735-1 |  | Not at all relevant |  |  |  |
| Recommendations for design of a mobile application to support management of anxiety and depression among Black American women | 10.3389/fdgth.2022.1028408 |  | Not at all relevant |  |  |  |
| Web-Based Short Video Intervention and Short Message Comparison of Repeat Blood Donation Behavior Based on an Extended Theory of Planned Behavior: Prospective Randomized Controlled Trial Study | 10.2196/37467 |  | Not at all relevant |  |  |  |
| Acceptance of a flipped classroom to improve university students' learning: An empirical study on the TAM model and the unified theory of acceptance and use of technology (UTAUT) | 10.1016/j.heliyon.2022.e12529 |  | Not at all relevant |  |  |  |
| Digital Medicine System in Veterans With Severe Mental Illness: Feasibility and Acceptability Study | 10.2196/34893 |  | Not at all relevant |  |  |  |
| Effect of Mobile Phone App-Based Interventions on Quality of Life and Psychological Symptoms Among Adult Cancer Survivors: Systematic Review and Meta-analysis of Randomized Controlled Trials | 10.2196/39799 | Review paper |  |  |  |  |
| Naturalizing digital and quality of life in chronic diseases: Systematic review to research perspective into technological advancing and personalized medicine | 10.1177/20552076221144857 | Review paper |  |  |  |  |
| Depression, anxiety and related factors among Syrian breast cancer patients: a cross-sectional study | 10.1186/s12888-022-04469-y |  | Not at all relevant |  |  |  |
| Applying Gamification Principles and Therapeutic Movement Sequences to Design an Interactive Physical Activity Game: Development Study | 10.2196/38133 |  | Not at all relevant |  |  |  |
| Preventing Postpartum Depression in the Early Postpartum Period Using an App-Based Cognitive Behavioral Therapy Program: A Pilot Randomized Controlled Study | 10.3390/ijerph192416824 |  |  |  |  | 1 |
| Personalised app-based relapse prevention of depressive and anxiety disorders in remitted adolescents and young adults: a protocol of the StayFine RCT | 10.1136/bmjopen-2021-058560 |  | Not at all relevant |  |  |  |
| Pre-partum HRV as a predictor of postpartum depression: The potential use of a smartphone application for physiological recordings | 10.1016/j.jad.2022.09.056 |  | Not at all relevant |  |  |  |
| Evaluation of chatbot-delivered interventions for self-management of depression: Content analysis | 10.1016/j.jad.2022.09.028 |  | Not at all relevant |  |  |  |
| E-mental health implementation in inpatient care: Exploring its potential and future challenges | 10.3389/fdgth.2022.1027864 |  | Not at all relevant |  |  |  |
| Efficacy and Feasibility of the Minimal Therapist-Guided Four-Week Online Audio-Based Mindfulness Program 'Mindful Senses' for Burnout and Stress Reduction in Medical Personnel: A Randomized Controlled Trial | 10.3390/healthcare10122532 |  | Not at all relevant |  |  |  |
| Six-Month Clinical and Ecological Momentary Assessment Follow-Up of Patients at High Risk of Suicide: A Survival Analysis | 10.4088/JCP.22m14411 |  | Not at all relevant |  |  |  |
| Efficacy of the Mental Health App Intellect to Reduce Stress: Randomized Controlled Trial With a 1-Month Follow-up | 10.2196/40723 |  | Not at all relevant |  |  |  |
| The Use of Passive Smartphone Data to Monitor Anxiety and Depression Among College Students in Real-World Settings: Protocol for a Systematic Review | 10.2196/38785 | Review paper |  |  |  |  |
| Development of an mHealth App-Based Intervention for Depressive Rumination (RuminAid): Mixed Methods Focus Group Evaluation | 10.2196/40045 |  | Not at all relevant |  |  |  |
| Use of an Interactive Obesity Treatment Approach in Individuals With Severe Mental Illness: Feasibility, Acceptability, and Proposed Engagement Criteria | 10.2196/38496 |  | Not at all relevant |  |  |  |
| The Effectiveness of Internet-Guided Self-help Interventions to Promote Physical Activity Among Individuals With Depression: Systematic Review | 10.2196/38049 | Review paper |  |  |  |  |
| The Mediating Role of Selfitis in the Associations between Self-Esteem, Problematic Social Media Use, Problematic Smartphone Use, Body-Self Appearance, and Psychological Distress among Young Ghanaian Adults | 10.3390/healthcare10122500 |  | Not at all relevant |  |  |  |
| Determining the readiness of patients with renal failure to use health information technology | 10.1186/s12911-022-02073-4 |  | Not at all relevant |  |  |  |
| Differentiated mental health patterns in pregnancy during COVID-19 first two waves in Sweden: a mixed methods study using digital phenotyping | 10.1038/s41598-022-25107-3 |  | Not at all relevant |  |  |  |
| Mobile Health-Supported Virtual Reality and Group Problem Management Plus: Protocol for a Cluster Randomized Trial Among Urban Refugee and Displaced Youth in Kampala, Uganda (Tushirikiane4MH, Supporting Each Other for Mental Health) | 10.2196/42342 |  | Not at all relevant |  |  |  |
| Effects and safety of auricular acupressure on depression and anxiety in isolated COVID-19 patients: A single-blind randomized controlled trial | 10.3389/fpsyt.2022.1041829 |  | Not at all relevant |  |  |  |
| Associations between smartphone keystroke dynamics and cognition in MS | 10.1177/20552076221143234 |  | Not at all relevant |  |  |  |
| Experiences of Patients and Therapists Testing a Virtual Reality Exposure App for Symptoms of Claustrophobia: Mixed Methods Study | 10.2196/40056 |  | Not at all relevant |  |  |  |
| A Smartphone-Based Intervention for Anxiety and Depression in Racially and Ethnically Diverse Adults (EASE): Protocol for a Randomized Controlled Trial | 10.2196/40713 |  |  | Telehealth app |  |  |
| The effects of daily autobiographical memory training on memory bias, mood and stress resilience in dysphoric individuals | 10.1038/s41598-022-25379-9 |  | Not at all relevant |  |  |  |
| Validity and reliability of the Chinese version of the Young Positive Schema Questionnaire | 10.3389/fpsyg.2022.1048954 |  | Not at all relevant |  |  |  |
| Associations between anxiety, depression, and risk of suicidal behaviors in Chinese medical college students | 10.3389/fpsyt.2022.1012298 |  | Not at all relevant |  |  |  |
| College students' anxiety after returning to school during the COVID-19 epidemic: What should we care | 10.1097/MD.0000000000032068 |  | Not at all relevant |  |  |  |
| Public Trust in Artificial Intelligence Applications in Mental Health Care: Topic Modeling Analysis | 10.2196/38799 |  | Not at all relevant |  |  |  |
| Case 312 | 10.1148/radiol.213042 |  | Not at all relevant |  |  |  |
| Virtual reality mobility for burn patients (VR-MOBILE): A within-subject-controlled trial protocol | 10.1002/pne2.12086 |  | Not at all relevant |  |  |  |
| Psychophysiological effects of rhythmic music combined with aerobic exercise in college students with minimal depressive symptoms | 10.1016/j.smhs.2022.03.004 |  | Not at all relevant |  |  |  |
| The impact of media on children during the COVID-19 pandemic: A narrative review | 10.1016/j.heliyon.2022.e12489 | Review paper |  |  |  |  |
| Efficacy and mechanisms of mobile application-delivered Acceptance and Commitment Therapy for posttraumatic stress disorder in China: Study protocol for a randomized controlled trial | 10.1016/j.invent.2022.100585 |  | Not at all relevant |  |  |  |
| Relationships between body image and mental health in white, cisgender college students | 10.1007/s40519-022-01495-3 |  | Not at all relevant |  |  |  |
| Impact of smartphone overuse on 1-year severe depressive symptoms and momentary negative affect: Longitudinal and experience sampling findings from a representative epidemiological youth sample in Hong Kong | 10.1016/j.psychres.2022.114939 |  |  | Mobile addiction |  |  |
| The association between autistic traits and excessive smartphone use in Chinese college students: The chain mediating roles of social interaction anxiety and loneliness | 10.1016/j.ridd.2022.104369 |  | Not at all relevant |  |  |  |
| Women's Experiences of Symptoms of Suspected or Confirmed COVID-19 Illness During the Pandemic | 10.1016/j.nwh.2022.09.005 |  | Not at all relevant |  |  |  |
| Excessive audio-visual stimulation leads to impaired social behaviour with an effect on amygdala: Early life excessive exposure to digital devices in male rats | 10.1111/ejn.15837 |  | Not at all relevant |  |  |  |
| Efficacy and safety of a mobile app intervention in patients with inflammatory arthritis: a prospective pilot study | 10.1007/s00296-022-05175-4 |  | Not at all relevant |  |  |  |
| The Assessment of Driving Fitness Using an On-Road Evaluation in Patients With Cirrhosis | 10.14309/ajg.0000000000001927 |  | Not at all relevant |  |  |  |
| Using smartphone-based ecological momentary assessment and personalized feedback for patients with chronic cancer-related fatigue: A proof-of-concept study | 10.1016/j.invent.2022.100568 |  | Not at all relevant |  |  |  |
| Mindfulness based intervention through mobile app for colorectal cancer people awaiting surgery: A randomized clinical trial | 10.1016/j.cireng.2022.08.008 |  | Not at all relevant |  |  |  |
| Home to Stay: A randomized controlled trial protocol to assess use of a mobile app to reduce readmissions following colorectal surgery | 10.1111/codi.16312 |  | Not at all relevant |  |  |  |
| Triiodothyronine Treatment reverses Depression-Like Behavior in a triple-transgenic animal model of Alzheimer's Disease | 10.1007/s11011-022-01055-9 |  | Not at all relevant |  |  |  |
| A creative and practical approach to postpartum discharge education: Pecha Kucha training via smart phone | 10.1080/07399332.2022.2043860 |  | Not at all relevant |  |  |  |
| Components of smartphone cognitive-behavioural therapy for subthreshold depression among 1093 university students: a factorial trial | 10.1136/ebmental-2022-300455 |  | Not at all relevant |  |  |  |
| "We are looking at the future right now": community acceptability of a home-based viral load test device in the context of HIV cure-related research with analytical treatment interruptions in the United States |  |  | Not at all relevant |  |  |  |
| Patient and therapist experiences of using a smartphone application monitoring anxiety symptoms | 10.1080/17482631.2022.2044981 |  |  | Screening, no treatment |  |  |
| Maternal depression: Technology enabled self screening in real time | 10.1080/07399332.2022.2037603 |  | Not at all relevant |  |  |  |
| Psychosocial and Culturally-Specific Factors Related to Intimate Partner Violence Victimization among a Sample of Latino Sexual Minority Cis Men in the U.S | 10.1177/08862605211072167 |  | Not at all relevant |  |  |  |
| Preconception menstrual cycle disorder and antenatal depression: a cross-sectional study with prerecorded information | 10.1080/0167482X.2021.2010699 |  | Not at all relevant |  |  |  |
| Roll Back Stroke: The Way Forward for Physicians and Patients |  |  | Not at all relevant |  |  |  |
| Protocol for a randomised controlled trial evaluating the effectiveness of a CBT-based smartphone application for improving mental health outcomes in adolescents: the MobiliseMe study | 10.1186/s12888-022-04383-3 |  |  | Telehealth app |  |  |
| The Passive Monitoring of Depression and Anxiety Among Workers Using Digital Biomarkers Based on Their Physical Activity and Working Conditions: 2-Week Longitudinal Study | 10.2196/40339 |  |  | Screening, no treatment |  |  |
| Digital Phenotyping Data to Predict Symptom Improvement and App Personalization: Protocol for a Prospective Study | 10.2196/37954 |  | Not at all relevant |  |  |  |
| Passive Sensing in the Prediction of Suicidal Thoughts and Behaviors: Protocol for a Systematic Review | 10.2196/42146 | Review paper |  |  |  |  |
| A Digital Health Intervention for Stress and Anxiety Relief in Perioperative Care: Protocol for a Feasibility Randomized Controlled Trial | 10.2196/38536 |  | Not at all relevant |  |  |  |
| Effectiveness of Mindfulness and Positive Strengthening mHealth Interventions for the Promotion of Subjective Emotional Wellbeing and Management of Self-Efficacy for Chronic Cardiac Diseases | 10.3390/jpm12121953 |  | Not at all relevant |  |  |  |
| Does prediction error during exposure relate to clinical outcomes in cognitive behavior therapy for social anxiety disorder? A study protocol | 10.3389/fpsyt.2022.1000686 |  | Not at all relevant |  |  |  |
| Can a mobile app technology reduce emergency department visits and readmissions after lung resection? A prospective cohort study | 10.1503/cjs.000122 |  | Not at all relevant |  |  |  |
| Positive Affective Recovery in Daily Life as a Momentary Mechanism Across Subclinical and Clinical Stages of Mental Disorder: Experience Sampling Study | 10.2196/37394 |  | Not at all relevant |  |  |  |
| Effects of a Smartphone-Based Out-of-Hospital Screening App for Neonatal Hyperbilirubinemia on Neonatal Readmission Rates and Maternal Anxiety: Randomized Controlled Trial | 10.2196/37843 |  | Not at all relevant |  |  |  |
| Health Tracking via Mobile Apps for Depression Self-management: Qualitative Content Analysis of User Reviews | 10.2196/40133 | Review paper |  |  |  |  |
| Factors Influencing Use of Fitness Apps by Adults under Influence of COVID-19 | 10.3390/ijerph192315460 |  | Not at all relevant |  |  |  |
| Chatbot-Delivered Cognitive Behavioral Therapy in Adolescents With Depression and Anxiety During the COVID-19 Pandemic: Feasibility and Acceptability Study | 10.2196/40242 |  | Not at all relevant |  |  |  |
| A Conversational, Virtual, Avatar-Led Cognitive Behavioral Therapy App Intervention for Improving the Quality of Life and Mental Health of People With Epilepsy: Protocol for a Randomized Controlled Trial | 10.2196/40261 |  | Not at all relevant |  |  |  |
| Mental Health Chatbot for Young Adults With Depressive Symptoms During the COVID-19 Pandemic: Single-Blind, Three-Arm Randomized Controlled Trial | 10.2196/40719 |  | Not at all relevant |  |  |  |
| Heart and Breathing Rate Variations as Biomarkers for Anxiety Detection | 10.3390/bioengineering9110711 |  | Not at all relevant |  |  |  |
| Personalised depression forecasting using mobile sensor data and ecological momentary assessment | 10.3389/fdgth.2022.964582 |  | Not at all relevant |  |  |  |
| Impact of glycemic variability on cognitive impairment, disordered eating behaviors and self-management skills in patients with type 1 diabetes: study protocol for a cross-sectional online study, the Sugar Swing study | 10.1186/s12902-022-01191-4 |  | Not at all relevant |  |  |  |
| Adjusting intervention strategies for mental health of COVID-19 patients: A network analysis based on a survey in Omicron-infected patients | 10.3389/fpubh.2022.1038296 |  | Not at all relevant |  |  |  |
| Effects of a Digital Musculoskeletal Acute Care Program on Chronic Pain Prevention: An Observational Study with Nonparticipant Comparison Group | 10.2147/JPR.S385134 |  | Not at all relevant |  |  |  |
| Development and evaluation of a psychological adjustment communication system for adolescents with polycystic ovary syndrome at a high risk of depression: A mixed-method study protocol | 10.3389/fpsyt.2022.937280 |  | Not at all relevant |  |  |  |
| Students' Emotional Well-being and Academic Functioning Before, During, and After Lockdown in Germany: Cohort Study | 10.2196/34388 |  | Not at all relevant |  |  |  |
| All-in-one calcium nanoflowers for dual outputs biosensor: A simultaneous strategy for depression drug evaluation and non-invasive stress assessment | 10.1016/j.bios.2022.114655 |  | Not at all relevant |  |  |  |
| Characteristics and trends in acceptance and commitment therapy research: A bibliometric analysis | 10.3389/fpsyg.2022.980848 |  | Not at all relevant |  |  |  |
| Telemedicine Interventions as an Attempt to Improve the Mental Health of Populations during the COVID-19 Pandemic-A Narrative Review | 10.3390/ijerph192214945 | Review paper |  |  |  |  |
| Developing an app-based self-management program for people living with HIV: a randomized controlled pilot study during the COVID-19 pandemic | 10.1038/s41598-022-19238-w |  | Not at all relevant |  |  |  |
| Effect of mobile-based self-management application on stroke outcomes: a study protocol for triple blinded randomized controlled trial | 10.1186/s12911-022-02033-y |  | Not at all relevant |  |  |  |
| The Effects of Tinnitus in Probabilistic Learning Tasks: Protocol for an Ecological Momentary Assessment Study | 10.2196/36583 |  | Not at all relevant |  |  |  |
| Clinical EFT as an evidence-based practice for the treatment of psychological and physiological conditions: A systematic review | 10.3389/fpsyg.2022.951451 | Review paper |  |  |  |  |
| An approach to psychosocial health among middle-aged and older people by remote sharing of photos and videos from family members not living together: A feasibility study | 10.3389/fpubh.2022.962977 |  | Not at all relevant |  |  |  |
| Muscular Swedish mutant APP-to-Brain axis in the development of Alzheimer's disease | 10.1038/s41419-022-05378-4 |  | Not at all relevant |  |  |  |
| Stressors and Destressors in Working From Home Based on Context and Physiology From Self-Reports and Smartwatch Measurements: International Observational Study Trial | 10.2196/38562 |  | Not at all relevant |  |  |  |
| Personalized Prediction of Response to Smartphone-Delivered Meditation Training: Randomized Controlled Trial | 10.2196/41566 |  | Not at all relevant |  |  |  |
| Interventions to support the mental health and well-being of front-line healthcare workers in hospitals during pandemics: an evidence review and synthesis | 10.1136/bmjopen-2022-061317 | Review paper |  |  |  |  |
| Effectiveness of Mental Health Apps for Distress During COVID-19 in US Unemployed and Essential Workers: Remote Pragmatic Randomized Clinical Trial | 10.2196/41689 |  | Not at all relevant |  |  |  |
| Applying Deep Learning on a Few EEG Electrodes during Resting State Reveals Depressive States. A Data Driven Study | 10.3390/brainsci12111506 |  | Not at all relevant |  |  |  |
| Parental Confidence in Relation to Antipyretic Use, Warning Signs, Symptoms and Well-Being in Fever Management-Results from an App-Based Registry | 10.3390/ijerph192114502 |  | Not at all relevant |  |  |  |
| Feasibility of a smartphone app to monitor patient reported outcomes in multiple sclerosis: The haMSter interventional trial | 10.1177/20552076221135387 |  | Not at all relevant |  |  |  |
| Mobile phone-based interventions for mental health show promise of effectiveness, but what does the evidence tell us about what needs to come next? | 10.1371/journal.pdig.0000126 |  | Not at all relevant |  |  |  |
| A systematic review and meta-analysis of digital application use in clinical research in pain medicine | 10.3389/fdgth.2022.850601 | Review paper |  |  |  |  |
| More Than a Health Fair: Preventive Health Care During COVID-19 Vaccine Events | 10.12788/fp.0328 |  | Not at all relevant |  |  |  |
| A Pilot Feasibility Open Trial of an Interpretation Bias Intervention for Parents of Anxious Children | 10.1016/j.cbpra.2021.09.005 |  | Not at all relevant |  |  |  |
| Striatal ZBTB16 Is Associated With Cognitive Deficits in Alzheimer Disease Mice | 10.5213/inj.2244254.127 |  | Not at all relevant |  |  |  |
| A global mental health opportunity: How can cultural concepts of distress broaden the construct of immobility? | 10.1016/j.gloenvcha.2022.102594 |  | Not at all relevant |  |  |  |
| A Randomized Controlled Trial of a Smartphone-Based Well-Being Training in Public School System Employees During the COVID-19 Pandemic | 10.1037/edu0000739 |  | Not at all relevant |  |  |  |
| Online Dating and Mental Health among Young Sexual Minority Black Men: Is Ethnic Identity Protective in the Face of Sexual Racism? | 10.3390/ijerph192114263 |  | Not at all relevant |  |  |  |
| Telemedicine for outpatient treatment of depressive disorders | 10.12788/acp.0091 |  | Not at all relevant |  |  |  |
| The preventive effect of internet-based cognitive behavioral therapy for prevention of depression during pregnancy and in the postpartum period (iPDP): a large scale randomized controlled trial | 10.1111/pcn.13458 |  | Not at all relevant |  |  |  |
| An Initial Test of the Efficacy of a Digital Health Intervention for Bariatric Surgery Candidates | 10.1007/s11695-022-06258-8 |  | Not at all relevant |  |  |  |
| SGO and the elephant that is still in the room: Wellness, burnout and gynecologic oncology | 10.1016/j.ygyno.2022.08.018 |  | Not at all relevant |  |  |  |
| Scoping review of interventions to support families with preterm infants post-NICU discharge | 10.1016/j.pedn.2022.08.014 | Review paper |  |  |  |  |
| Effectiveness of mobile application-based perinatal interventions in improving parenting outcomes: A systematic review | 10.1016/j.midw.2022.103457 | Review paper |  |  |  |  |
| [Feasibility study: The medical imaging as a tool for therapeutic education in radiotherapy] | 10.1016/j.canrad.2022.04.004 |  | Not at all relevant |  |  |  |
| Parental Phubbing, Self-Esteem, and Suicidal Ideation among Chinese Adolescents: A Longitudinal Mediational Analysis | 10.1007/s10964-022-01655-9 |  | Not at all relevant |  |  |  |
| Smartphone Usage Patterns and Their Physical, Psychological, and Cyber-Behavioral Predictors Among Adolescents in South Korea | 10.1111/josh.13198 |  | Not at all relevant |  |  |  |
| Web-based support services to help prevent suicide in young people and students: A mixed-methods, user-informed review of characteristics and effective elements | 10.1111/hsc.13819 | Review paper |  |  |  |  |
| Diet or medication in primary care patients with IBS: the DOMINO study - a randomised trial supported by the Belgian Health Care Knowledge Centre (KCE Trials Programme) and the Rome Foundation Research Institute | 10.1136/gutjnl-2021-325821 |  | Not at all relevant |  |  |  |
| A follow-up of pain reported by children undergoing outpatient surgery using a smartphone application: AlgoDARPEF multicenter descriptive prospective study | 10.1097/j.pain.0000000000002620 |  | Not at all relevant |  |  |  |
| Small Moments, Big Impact: Pilot Trial of a Relational Health App for Primary Care | 10.1016/j.acap.2022.02.010 |  | Not at all relevant |  |  |  |
| Parent and child mental health trajectories April 2020 to May 2021: Strict lockdown versus no lockdown in Australia | 10.1177/00048674211065365 |  | Not at all relevant |  |  |  |
| Mobile meditation for improving quality of life, anxiety and depression among surgical residents and faculty | 10.1017/S0022215121003091 |  |  |  |  | 1 |
| Effect of a Smart Pill Bottle Reminder Intervention on Medication Adherence, Self-efficacy, and Depression in Breast Cancer Survivors | 10.1097/NCC.0000000000001030 |  | Not at all relevant |  |  |  |
| Physical outcomes of patients infected with HIV requiring intensive care unit admission for mechanical ventilation at one South African hospital: a pilot study | 10.1080/09593985.2021.1941456 |  | Not at all relevant |  |  |  |
| Media and technology usage and attitudes in emergency department patients | 10.3389/fdgth.2022.894683 |  | Not at all relevant |  |  |  |
| Interventions Including Smart Technology Compared With Face-to-face Physical Activity Interventions in Older Adults: Systematic Review and Meta-analysis | 10.2196/36134 | Review paper |  |  |  |  |
| The interplay of daily affect and impulsivity measured by mobile surveys in bipolar disorder | 10.1186/s40345-022-00270-8 |  | Not at all relevant |  |  |  |
| Racial and Ethnic Differences in Outcomes of a 12-Week Digital Rehabilitation Program for Musculoskeletal Pain: Prospective Longitudinal Cohort Study | 10.2196/41306 |  | Not at all relevant |  |  |  |
| Can ResilienceNHope, an evidence-based text and email messaging innovative suite of programs help to close the psychological treatment and mental health literacy gaps in college students? | 10.3389/fpubh.2022.890131 |  | Not at all relevant |  |  |  |
| Development of an Auxiliary Platform (Mentali) for the Primary Screening of Anxiety and Depression in Young Adults | 10.3390/ijerph192114033 |  | Not at all relevant |  |  |  |
| Introducing mobile apps to promote the well-being of German and Italian university students. A cross-national application of the Technology Acceptance Model | 10.1007/s12144-022-03856-8 |  | Not at all relevant |  |  |  |
| Smartphone-based ecological momentary assessment reveals mental health benefits of birdlife | 10.1038/s41598-022-20207-6 |  | Not at all relevant |  |  |  |
| An App-Based Digit Symbol Substitution Test for Assessment of Cognitive Deficits in Adults With Major Depressive Disorder: Evaluation Study | 10.2196/33871 |  | Not at all relevant |  |  |  |
| Mindfulness Training for Depressed Older Adults Using Smartphone Technology: Protocol for a Fully Remote Precision Clinical Trial | 10.2196/39233 |  | Not at all relevant |  |  |  |
| Mobile App to Enhance Patient Activation and Patient-Provider Communication in Major Depressive Disorder Management: Collaborative, Randomized Controlled Pilot Study | 10.2196/34923 |  | Not at all relevant |  |  |  |
| User Behavior of a Publicly Available, Free-to-Use, Self-guided mHealth App for Depression: Observational Study in a Global Sample | 10.2196/35538 |  |  |  | No treatment |  |
| Virtual Reality Applications in Medicine During the COVID-19 Pandemic: Systematic Review | 10.2196/35000 | Review paper |  |  |  |  |
| Smartphone-assisted guided self-help cognitive behavioral therapy for young people with distressing voices (SmartVoices): study protocol for a randomized controlled trial | 10.1186/s13063-022-06846-0 |  | Not at all relevant |  |  |  |
| Brief internet-delivered cognitive-behavioural intervention for children and adolescents with symptoms of anxiety and depression during the COVID-19 pandemic: a randomised controlled trial protocol | 10.1186/s13063-022-06836-2 |  |  | No Mobile app |  |  |
| Gamification improves antidepressant effects of cognitive control training-A pilot trial | 10.3389/fdgth.2022.994484 |  | Not at all relevant |  |  |  |
| Beyond Blue and Green Spaces: Identifying and Characterizing Restorative Environments on Sichuan Technology and Business University Campus | 10.3390/ijerph192013500 |  | Not at all relevant |  |  |  |
| Study protocol of EMPOWER: A cluster randomized trial of a multimodal eHealth intervention for promoting mental health in the workplace following a stepped wedge trial design | 10.1177/20552076221131145 |  | Not at all relevant |  |  |  |
| A randomized controlled trial of an mHealth application with nursing interaction to promote quality of life among community-dwelling older adults | 10.3389/fpsyt.2022.978416 |  | Not at all relevant |  |  |  |
| Urban-Rural Distinction or Economic Segmentation: A Study on Fear and Inferiority in Poor Children's Peer Relationships | 10.3390/healthcare10102057 |  | Not at all relevant |  |  |  |
| Clinical course and serum amyloid β levels in elderly patients with major depressive disorder | 10.1016/j.jad.2022.07.073 |  | Not at all relevant |  |  |  |
| Temporal and contemporaneous network structures of affect and physical activity in emotional disorders | 10.1016/j.jad.2022.07.061 |  | Not at all relevant |  |  |  |
| Moringa Oleifera Alleviates Aβ Burden and Improves Synaptic Plasticity and Cognitive Impairments in APP/PS1 Mice | 10.3390/nu14204284 |  | Not at all relevant |  |  |  |
| Digital Mental Health Interventions for Depression: Scoping Review of User Engagement | 10.2196/39204 | Review paper |  |  |  |  |
| The cumulative effect of chronic stress and depressive symptoms affects heart rate in a working population | 10.3389/fpsyt.2022.1022298 |  | Not at all relevant |  |  |  |
| Neurological Outpatients Prefer EEG Home-Monitoring over Inpatient Monitoring-An Analysis Based on the UTAUT Model | 10.3390/ijerph192013202 |  | Not at all relevant |  |  |  |
| Staying connected: smartphone acceptance and use level differences of older adults in China | 10.1007/s10209-022-00933-4 |  | Not at all relevant |  |  |  |
| An Evaluation of a Mobile App for Chronic Low Back Pain Management: Prospective Pilot Study | 10.2196/40869 |  | Not at all relevant |  |  |  |
| Mental Health Mobile Apps in the French App Store: Assessment Study of Functionality and Quality | 10.2196/41282 | Review paper |  |  |  |  |
| A Digital Intervention Using Daily Financial Incentives to Increase Medication Adherence in Severe Mental Illness: Single-Arm Longitudinal Pilot Study | 10.2196/37184 |  | Not at all relevant |  |  |  |
| Study on a Bayes evaluation of the working ability of petroleum workers in the Karamay region, Xinjiang, China | 10.3389/fpsyg.2022.1011137 |  | Not at all relevant |  |  |  |
| Positive intervention effect of mobile health application based on mindfulness and social support theory on postpartum depression symptoms of puerperae | 10.1186/s12905-022-01996-4 |  |  |  |  | 1 |
| A Digital Mental Health Intervention (Inuka) for Common Mental Health Disorders in Zimbabwean Adults in Response to the COVID-19 Pandemic: Feasibility and Acceptability Pilot Study | 10.2196/37968 |  | Not at all relevant |  |  |  |
| Home-based transcranial direct current stimulation in dual active treatments for symptoms of depression and anxiety: A case series | 10.3389/fpsyt.2022.947435 |  | Not at all relevant |  |  |  |
| University freshmen's excessive smartphone use and psychological safety during the COVID-19 pandemic | 10.3389/fpsyt.2022.993555 |  | Not at all relevant |  |  |  |
| The BEACON study: an update to the protocol for a cohort study as part of an evaluation of the effectiveness of smartphone-assisted problem-solving therapy in men who present with intentional self-harm to emergency departments in Ontario | 10.1186/s13063-022-06788-7 |  | Not at all relevant |  |  |  |
| Impact of adherence to a lifestyle-integrated programme on physical function and behavioural complexity in young older adults at risk of functional decline: a multicentre RCT secondary analysis | 10.1136/bmjopen-2021-054229 |  | Not at all relevant |  |  |  |
| Guided Internet-Delivered Treatment for Depression: Scoping Review | 10.2196/37342 | Review paper |  |  |  |  |
| Associations Between Depression Symptom Severity and Daily-Life Gait Characteristics Derived From Long-Term Acceleration Signals in Real-World Settings: Retrospective Analysis | 10.2196/40667 |  | Not at all relevant |  |  |  |
| Assessment of Oncology Advanced Practice Professional Willingness to Participate in Medical Aid in Dying | 10.1001/jamanetworkopen.2022.39068 |  | Not at all relevant |  |  |  |
| Unveiling mechanisms of change in digital interventions for depression: Study protocol for a systematic review and individual participant data meta-analysis | 10.3389/fpsyt.2022.899115 | Review paper |  |  |  |  |
| Fibroadenoma: a guide for junior clinicians | 10.12968/hmed.2022.0070 |  | Not at all relevant |  |  |  |
| Empowering health workers and leveraging digital technology to improve access to mental health and epilepsy care: A longitudinal quasi-experimental study in Hlaing Thar Yar Township | 10.1016/j.lansea.2022.100052 |  | Not at all relevant |  |  |  |
| Screening for mental illness using GMHAT App of patients with Type 2 diabetes mellitus at a teaching institute hospital in India - A cross sectional study | 10.4103/jfmpc.jfmpc_277_22 |  | Not at all relevant |  |  |  |
| Telepsychology for Individuals With Spinal Cord Injury: Protocol for a Randomized Control Study of Video-Based Cognitive Behavioral Therapy | 10.46292/sci22-00010 |  | Not at all relevant |  |  |  |
| Going Remote: A Revised Study Protocol for a Pilot Randomized Controlled Trial for Biofeedback Treatment of Anxiety Associated With Chronic Spinal Cord Injury | 10.46292/sci22-00012 |  | Not at all relevant |  |  |  |
| Controllability of Structural Brain Networks and the Waxing and Waning of Negative Affect in Daily Life | 10.1016/j.bpsgos.2021.11.008 |  | Not at all relevant |  |  |  |
| The Problem Management Plus psychosocial intervention for distressed and functionally impaired asylum seekers and refugees: the PROSPER feasibility RCT | 10.3310/NZXA0081 |  | Not at all relevant |  |  |  |
| Preliminary validation of the Turkish version of the pain catastrophizing scale for children and parents (PCS-C and PCS-P) in primary childhood headache | 10.14744/agri.2021.92195 |  | Not at all relevant |  |  |  |
| Locomotor Hyperactivity in the Early-Stage Alzheimer's Disease-like Pathology of APP/PS1 Mice: Associated with Impaired Polarization of Astrocyte Aquaporin 4 | 10.14336/AD.2022.0219 |  | Not at all relevant |  |  |  |
| The MK2 cascade mediates transient alteration in mGluR-LTD and spatial learning in a murine model of Alzheimer's disease | 10.1111/acel.13717 |  | Not at all relevant |  |  |  |
| Problematic smartphone use and functional somatic symptoms among adolescents: Mediating roles of depressive symptoms and peer relationships by gender | 10.1016/j.apnu.2022.04.003 |  | Not at all relevant |  |  |  |
| Severity of loneliness, depression and perceived social support in adults in the empty nest stage of the family life cycle and the influence of using digital technology | 10.1016/j.ajp.2022.103245 |  | Not at all relevant |  |  |  |
| The impact of COVID-19 pandemic on mental health in gay, bisexual, and other men who have sex with men in China: Difference by HIV status | 10.1016/j.jpsychires.2022.07.028 |  | Not at all relevant |  |  |  |
| A randomized controlled trial on virtual reality distraction during venous cannulation in young children | 10.1111/aas.14120 |  | Not at all relevant |  |  |  |
| The feasibility and acceptability of using smartphones to assess suicide risk among Spanish-speaking adult outpatients | 10.1111/sltb.12889 |  | Not at all relevant |  |  |  |
| Perceptions of Divine Forgiveness, Religious Comfort, and Depression in Psychiatric Inpatients: A Mixed Methods Study | 10.1007/s10943-022-01511-x |  |  | Screening, no treatment |  |  |
| The feasibility, acceptability, and preliminary efficacy of an mHealth mindfulness therapy for caregivers of adults with cognitive impairment | 10.1080/13607863.2021.1963949 |  | Not at all relevant |  |  |  |
| Adult quality of life patterns and trajectories during the COVID-19 pandemic in Germany | 10.1007/s12144-022-03628-4 |  | Not at all relevant |  |  |  |
| Dramatic impacts on brain pathology, anxiety, and cognitive function in the knock-in APP(NL-G-F) mouse model of Alzheimer disease following long-term voluntary exercise | 10.1186/s13195-022-01085-6 |  | Not at all relevant |  |  |  |
| Systematic assessment of the quality and integrity of popular mental health smartphone apps using the American Psychiatric Association's app evaluation model | 10.3389/fdgth.2022.1003181 |  | Not at all relevant |  |  |  |
| Effect of a Digital Literacy Program on Older Adults' Digital Social Behavior: A Quasi-Experimental Study | 10.3390/ijerph191912404 |  | Not at all relevant |  |  |  |
| Acceptability and Feasibility of the Telehealth Bariatric Behavioral Intervention to Increase Physical Activity: Protocol for a Single-Case Experimental Study | 10.2196/39633 |  | Not at all relevant |  |  |  |
| A Social Media-Based Intervention for Chinese American Caregivers of Persons With Dementia: Protocol Development | 10.2196/40171 |  | Not at all relevant |  |  |  |
| Can a new role, the (Trainee) Associate Psychological Practitioner (T/APP), add value in General Practice? Results from the pilot year evaluation | 10.1017/S1463423622000482 |  | Not at all relevant |  |  |  |
| Synthesis of the Evidence on What Works for Whom in Telemental Health: Rapid Realist Review | 10.2196/38239 | Review paper |  |  |  |  |
| Impact of COVID-19 pandemic on learning status of student in Nepal | 10.4103/jehp.jehp_354_22 |  | Not at all relevant |  |  |  |
| Development and Evaluation: A Behavioral Activation Mobile Application for Self-Management of Stress for College Students | 10.3390/healthcare10101880 |  | Not at all relevant |  |  |  |
| The feasibility of using smartphone apps as treatment components for depressed suicidal outpatients | 10.3389/fpsyt.2022.971046 |  | Not at all relevant |  |  |  |
| Development and Evaluation of an Artificial Intelligence-Based Cognitive Exercise Game: A Pilot Study | 10.1155/2022/4403976 |  | Not at all relevant |  |  |  |
| Trends in Effectiveness of Organizational eHealth Interventions in Addressing Employee Mental Health: Systematic Review and Meta-analysis | 10.2196/37776 | Review paper |  |  |  |  |
| The Effectiveness of Mobile Apps for Monitoring and Management of Suicide Crisis: A Systematic Review of the Literature | 10.3390/jcm11195616 | Review paper |  |  |  |  |
| Assessing the Impact of Conversational Artificial Intelligence in the Treatment of Stress and Anxiety in Aging Adults: Randomized Controlled Trial | 10.2196/38067 |  | Not at all relevant |  |  |  |
| Social Frailty among Community-Dwelling Older Adults during the COVID-19 Pandemic in Korea: A Cross-Sectional Study | 10.3390/ijerph191911963 |  | Not at all relevant |  |  |  |
| Perinatal depression and its impact on infant outcomes and maternal-nurse SMS communication in a cohort of Kenyan women | 10.1186/s12884-022-05039-6 |  | Not at all relevant |  |  |  |
| The Transcranial Light Therapy Improves Synaptic Plasticity in the Alzheimer's Disease Mouse Model | 10.3390/brainsci12101272 |  | Not at all relevant |  |  |  |
| Ethnomedicinal Plants with Protective Effects against Beta-Amyloid Peptide (Aβ)1-42 Indicate Therapeutic Potential in a New In Vivo Model of Alzheimer's Disease | 10.3390/antiox11101865 |  | Not at all relevant |  |  |  |
| Gastric dysfunction in patients with chronic nausea and vomiting syndromes defined by a noninvasive gastric mapping device | 10.1126/scitranslmed.abq3544 |  | Not at all relevant |  |  |  |
| Preliminary Investigation of Shift, a Novel Smartphone App to Support Junior Doctors' Mental Health and Well-being: Examination of Symptom Progression, Usability, and Acceptability After 1 Month of Use | 10.2196/38497 |  | Not at all relevant |  |  |  |
| The impact of psychological distress on problematic smartphone use among college students: The mediating role of metacognitions about smartphone use | 10.3389/fpsyg.2022.932838 |  | Not at all relevant |  |  |  |
| Using Mobile Video-Teleconferencing to Deliver Secondary Stroke Prevention Interventions: A Pilot Study | 10.1089/tmr.2022.0026 |  | Not at all relevant |  |  |  |
| The relationship between COVID-19-related restrictions and fear of missing out, problematic smartphone use, and mental health in college students: The moderated moderation effect of resilience and social support | 10.3389/fpubh.2022.986498 |  | Not at all relevant |  |  |  |
| Smartphone-based Ecological Momentary Intervention for secondary prevention of suicidal thoughts and behaviour: protocol for the SmartCrisis V.2.0 randomised clinical trial | 10.1136/bmjopen-2021-051807 |  | Not at all relevant |  |  |  |
| Efficacy and Conflicts of Interest in Randomized Controlled Trials Evaluating Headspace and Calm Apps: Systematic Review | 10.2196/40924 | Review paper |  |  |  |  |
| Impact of Routines and Rituals on Burden of Treatment, Patient Training, Cognitive Load, and Anxiety in Self-Injected Biologic Therapy | 10.2147/PPA.S375037 |  | Not at all relevant |  |  |  |
| The effect of social media on the development of students' affective variables | 10.3389/fpsyg.2022.1010766 |  | Not at all relevant |  |  |  |
| Study Protocol of an App-Based Prevention Program for Perinatal Depression | 10.3390/ijerph191811634 |  | Not at all relevant |  |  |  |
| ACTonDiabetes: study protocol of a pragmatic randomised controlled trial for the evaluation of an acceptance and commitment-based internet-based and mobile-based intervention for adults living with type 1 or type 2 diabetes | 10.1136/bmjopen-2021-059336 |  | Not at all relevant |  |  |  |
| Participants' and Nurses' Experiences With a Digital Intervention for Patients With Depressive Symptoms and Comorbid Hypertension or Diabetes in Peru: Qualitative Post-Randomized Controlled Trial Study | 10.2196/35486 |  | Not at all relevant |  |  |  |
| Suction feeding of West African lungfish (Protopterus annectens): An XROMM analysis of jaw mechanics, cranial kinesis, and hyoid mobility | 10.1242/bio.059447 |  | Not at all relevant |  |  |  |
| Development and Pilot-Testing of a Patient Decision-Making Aid for Nutrition in Age-Related Macular Degeneration | 10.2147/PPA.S377748 |  | Not at all relevant |  |  |  |
| The Use of Smart Devices for Mental Health Diagnosis and Care | 10.3390/jcm11185359 |  | Not at all relevant |  |  |  |
| Association Between Personality Traits/Dimensions and Fear of No Mobile Phone Connectivity (nomophobia): Results of a Lebanese National Study | 10.4088/PCC.21m03036 |  | Not at all relevant |  |  |  |
| Effects of WeChat use on the subjective health of older adults | 10.3389/fpsyg.2022.919889 |  | Not at all relevant |  |  |  |
| Web-Based Mindfulness-Based Interventions for Well-being: Randomized Comparative Effectiveness Trial | 10.2196/35620 |  |  | No Mobile app |  |  |
| Usefulness of a Mobile Application (Mentali) for Anxiety and Depression Screening in Medical Students and Description of the Associated Triggering Factors | 10.3390/brainsci12091223 |  |  | Screening, no treatment |  |  |
| Two-Dimensional Convolutional Neural Network for Depression Episodes Detection in Real Time Using Motor Activity Time Series of Depresjon Dataset | 10.3390/bioengineering9090458 |  | Not at all relevant |  |  |  |
| Brain-In-Hand technology for adults with acquired brain injury: A convergence of mixed methods findings | 10.1177/20556683221117759 |  | Not at all relevant |  |  |  |
| The Achieving Self-directed Integrated Cancer Aftercare Intervention for Detection of Recurrent and Second Primary Melanoma in Survivors of Melanoma: Pilot Randomized Controlled Trial | 10.2196/37539 |  | Not at all relevant |  |  |  |
| An Intervention Offering Self-management Support Through mHealth and Health Coaching to Patients With Prostate Cancer: Interpretive Description of Patients' Experiences and Perspectives | 10.2196/34471 |  | Not at all relevant |  |  |  |
| Depression and anxiety of medical students at Kunming Medical University during COVID-19: A cross-sectional survey | 10.3389/fpubh.2022.957597 |  | Not at all relevant |  |  |  |
| Analyzing the changing relationship between personal consumption and suicide mortality during COVID-19 pandemic in Japan, using governmental and personal consumption transaction databases | 10.3389/fpubh.2022.982341 |  | Not at all relevant |  |  |  |
| Affective Recommender System for Pet Social Network | 10.3390/s22186759 |  | Not at all relevant |  |  |  |
| Effectiveness and Minimum Effective Dose of App-Based Mobile Health Interventions for Anxiety and Depression Symptom Reduction: Systematic Review and Meta-Analysis | 10.2196/39454 | Review paper |  |  |  |  |
| Reductions in anxiety, depression and insomnia in health care workers using a non-pharmaceutical intervention | 10.3389/fpsyt.2022.983165 |  | Not at all relevant |  |  |  |
| Effects of Serious Games on Depression in Older Adults: Systematic Review and Meta-analysis of Randomized Controlled Trials | 10.2196/37753 | Review paper |  |  |  |  |
| Efficiency of ER:YAG laser therapy in combination with behaviour management technique in reducing anxiety among paediatric dental patients - a study protocol for a randomised clinical trial | 10.1136/bmjopen-2021-054523 |  | Not at all relevant |  |  |  |
| Interventions and methods to prepare, educate or familiarise children and young people for radiological procedures: a scoping review | 10.1186/s13244-022-01278-5 | Review paper |  |  |  |  |
| Building Emotional Awareness and Mental Health (BEAM): study protocol for a phase III randomized controlled trial of the BEAM app-based program for mothers of children 18-36 months | 10.1186/s13063-022-06512-5 |  | Not at all relevant |  |  |  |
| Binary-Synaptic Plasticity in Ambipolar Ni-Silicide Schottky Barrier Poly-Si Thin Film Transistors Using Chitosan Electric Double Layer | 10.3390/nano12173063 |  | Not at all relevant |  |  |  |
| Lessons learned from recruiting into a longitudinal remote measurement study in major depressive disorder | 10.1038/s41746-022-00680-z |  | Not at all relevant |  |  |  |
| The Effects of a Digital Well-being Intervention on Older Adults: Retrospective Analysis of Real-world User Data | 10.2196/39851 |  | Not at all relevant |  |  |  |
| The association between premenstrual syndrome before pregnancy and antenatal depression: A cross-sectional study with prerecorded information | 10.1002/pcn5.27 |  | Not at all relevant |  |  |  |
| Yoga as an Escape from Depreciating Mental Health due to COVID 19: A Qualitative study analyzing the factors associated with mental status based on the experiences of geriatric population's participation in an Online program during COVID 19 lockdown in India | 10.4103/ijoy.ijoy_121_22 |  | Not at all relevant |  |  |  |
| Does Objectively Measured Social-Media or Smartphone Use Predict Depression, Anxiety, or Social Isolation Among Young Adults? | 10.1177/21677026221078309 |  | Not at all relevant |  |  |  |
| Which adolescents are well-suited to app-based mindfulness training? A randomized clinical trial and data-driven approach for personalized recommendations | 10.1037/ccp0000763 |  | Not at all relevant |  |  |  |
| The Efficacy of Mobile Phone-Based Interventions for the Treatment of Depression: A Systematic Meta-Review of Meta-Analyses of Randomized Controlled Trials |  | Review paper |  |  |  |  |
| Positive and negative psychosocial factors related to healthy and unhealthy weight control among nursing students | 10.1016/j.profnurs.2022.07.017 |  | Not at all relevant |  |  |  |
| The effects of a mobile phone-based psychological intervention program on stress, anxiety and self-efficacy among undergraduate nursing students during clinical practice: A randomized controlled trial | 10.1016/j.profnurs.2022.07.016 |  |  |  | Telehealth |  |
| Understanding users' perspectives on mobile apps for anxiety management | 10.3389/fdgth.2022.854263 |  | Not at all relevant |  |  |  |
| The Fudan Tinnitus Relieving System (FTRS): The initial results of a smartphone application for tinnitus management and treatment | 10.1016/j.invent.2022.100564 |  | Not at all relevant |  |  |  |
| Digital prevention of depression for farmers? A qualitative study on participants' experiences regarding determinants of acceptance and satisfaction with a tailored guided internet intervention program | 10.1016/j.invent.2022.100566 |  | Not at all relevant |  |  |  |
| Patient-Reported Symptom Burden of Charcot-Marie-Tooth Disease Type 1A: Findings From an Observational Digital Lifestyle Study | 10.1097/CND.0000000000000426 |  | Not at all relevant |  |  |  |
| A Pilot Implementation-Effectiveness Trial of a Single-Session Telehealth Workshop and Smartphone-Based Cognitive Behavioral Intervention for Managing Emotions Among College Students | 10.1016/j.beth.2022.04.008 |  | Not at all relevant |  |  |  |
| Rapid Deployment of a Mobile Medical Clinic During the COVID-19 Pandemic: Assessment of Dyadic Maternal-Child Care | 10.1007/s10995-022-03483-6 |  | Not at all relevant |  |  |  |
| Technology-Based Approaches for Supporting Perinatal Mental Health | 10.1007/s11920-022-01349-w |  | Not at all relevant |  |  |  |
| An Internet-delivered Cognitive-Behavioral Therapy (iCBT) for Prolonged Grief Disorder (PGD) in adults: A multiple-baseline single-case experimental design study | 10.1016/j.invent.2022.100558 |  | Not at all relevant |  |  |  |
| Smartphone-based interventions in bipolar disorder: Systematic review and meta-analyses of efficacy. A position paper from the International Society for Bipolar Disorders (ISBD) Big Data Task Force | 10.1111/bdi.13243 | Review paper |  |  |  |  |
| Temporal profiles of suicidal thoughts in daily life: Results from two mobile-based monitoring studies with high-risk adolescents | 10.1016/j.jpsychires.2022.06.050 |  | Not at all relevant |  |  |  |
| Predictors of mortality in emergency centre patients with acute pesticide poisoning in Uganda | 10.1016/j.afjem.2022.05.005 |  | Not at all relevant |  |  |  |
| Trauma-Informed Pedagogy to Foster Resilience in Graduate Nursing Education | 10.1097/01.NEP.0000000000001006 |  | Not at all relevant |  |  |  |
| [Experience of medical oncologists when telling a patient to be referred to a palliative care unit] | 10.1016/j.bulcan.2022.02.012 |  | Not at all relevant |  |  |  |
| The use of mobile technology to support work integrated learning in undergraduate nursing programs: An integrative review | 10.1016/j.nedt.2022.105451 | Review paper |  |  |  |  |
| Heart Rate Variability biofeedback therapy for children and adolescents with chronic pain: A pilot study | 10.1016/j.pedn.2022.06.008 |  | Not at all relevant |  |  |  |
| Early indicators of vulnerability to depression: The role of rumination and heart rate variability | 10.1016/j.jad.2022.06.049 |  | Not at all relevant |  |  |  |
| Persistent Dissociation and Its Neural Correlates in Predicting Outcomes After Trauma Exposure | 10.1176/appi.ajp.21090911 |  | Not at all relevant |  |  |  |
| Tele-medicine and improvement of mental health problems in COVID-19 pandemic: A systematic review | 10.1002/mpr.1924 | Review paper |  |  |  |  |
| Establishment to measure oxycodone in plasma with liquid chromatography-tandem mass spectrometry | 10.1002/npr2.12268 |  | Not at all relevant |  |  |  |
| Feasibility, Acceptability, and Potential Utility of Peer-supported Ecological Momentary Assessment Among People with Serious Mental Illness: a Pilot Study | 10.1007/s11126-022-09986-3 |  | Not at all relevant |  |  |  |
| Impact of COVID-19 on pregnancy worry in the United States | 10.1111/birt.12608 |  | Not at all relevant |  |  |  |
| A Group Intervention for Motivational Deficits: Preliminary Investigation of a Blended Care Approach Using Ambulatory Assessment | 10.1177/01454455211047605 |  | Not at all relevant |  |  |  |
| Describing, predicting and explaining adherence to total skin self-examination (TSSE) in people with melanoma: a 12-month longitudinal study | 10.1136/bmjopen-2021-056755 |  | Not at all relevant |  |  |  |
| Digital Interventions to Enhance Readiness for Psychological Therapy: Scoping Review | 10.2196/37851 | Review paper |  |  |  |  |
| Predictors of Dropout in a Digital Intervention for the Prevention and Treatment of Depression in Patients With Chronic Back Pain: Secondary Analysis of Two Randomized Controlled Trials | 10.2196/38261 |  | Not at all relevant |  |  |  |
| A Machine Learning Approach for Continuous Mining of Nonidentifiable Smartphone Data to Create a Novel Digital Biomarker Detecting Generalized Anxiety Disorder: Prospective Cohort Study | 10.2196/38943 |  | Not at all relevant |  |  |  |
| Digital Content-Free Speech Analysis Tool to Measure Affective Distress in Mental Health: Evaluation Study | 10.2196/37061 |  | Not at all relevant |  |  |  |
| Proactive Electronic Visits for Smoking Cessation and Chronic Obstructive Pulmonary Disease Screening in Primary Care: Randomized Controlled Trial of Feasibility, Acceptability, and Efficacy | 10.2196/38663 |  | Not at all relevant |  |  |  |
| Academic Anxiety, Self-Regulated Learning Ability, and Self-Esteem in Chinese Candidates for College Entrance Examination During the COVID-19 Outbreak: A Survey Study | 10.2147/PRBM.S360127 |  | Not at all relevant |  |  |  |
| Digital and Interactive Health Interventions Minimize the Physical and Psychological Impact of Breast Cancer, Increasing Women's Quality of Life: A Systematic Review and Meta-Analysis | 10.3390/cancers14174133 | Review paper |  |  |  |  |
| Heart Rate Variability Biofeedback to Treat Anxiety in Young People With Autism Spectrum Disorder: Findings From a Home-Based Pilot Study | 10.2196/37994 |  | Not at all relevant |  |  |  |
| The Parental and Children Report of the Prevalence of Depressive Symptoms in Children and Adolescents Amid the COVID-19 Pandemic: A Cross-Sectional Study From Oman | 10.3389/ijph.2022.1604474 |  | Not at all relevant |  |  |  |
| Consumer acceptance of using a digital technology to manage postpartum depression | 10.3389/fgwh.2022.844172 |  | Not at all relevant |  |  |  |
| Depressive rumination and heart rate variability: A pilot study on the effect of biofeedback on rumination and its physiological concomitants | 10.3389/fpsyt.2022.961294 |  | Not at all relevant |  |  |  |
| Health Care Workers' Need for Headspace: Findings From a Multisite Definitive Randomized Controlled Trial of an Unguided Digital Mindfulness-Based Self-help App to Reduce Healthcare Worker Stress | 10.2196/31744 |  | Not at all relevant |  |  |  |
| Has Smartphone Use Influenced Loneliness during the COVID-19 Pandemic in Japan? | 10.3390/ijerph191710540 |  | Not at all relevant |  |  |  |
| Development and evaluation of the HRSD-D, an image-based digital measure of the Hamilton rating scale for depression | 10.1038/s41598-022-18434-y |  | Not at all relevant |  |  |  |
| Depression in young people | 10.1016/S0140-6736(22)01012-1 |  | Not at all relevant |  |  |  |
| Digital Interventions for Emotion Regulation in Children and Early Adolescents: Systematic Review and Meta-analysis | 10.2196/31456 | Review paper |  |  |  |  |
| A Mobile Application for Symptom Management in Patients With Breast Cancer | 10.1188/22.ONF.409-420 |  | Not at all relevant |  |  |  |
| Remote Follow-up of Self-isolating Patients With COVID-19 Using a Patient Portal: Protocol for a Mixed Methods Pilot Study (Opal-COVID Study) | 10.2196/35760 |  | Not at all relevant |  |  |  |
| Feasibility and acceptability of experience sampling among LGBTQ+ young people with self-harmful thoughts and behaviours | 10.3389/fpsyt.2022.916164 |  | Not at all relevant |  |  |  |
| Clinical and cost-effectiveness of paramedic administered fascia iliaca compartment block for emergency hip fracture (RAPID 2)-protocol for an individually randomised parallel-group trial | 10.1186/s13063-022-06522-3 |  | Not at all relevant |  |  |  |
| Appropriate Use and Operationalization of Adherence to Digital Cognitive Behavioral Therapy for Depression and Anxiety in Youth: Systematic Review | 10.2196/37640 | Review paper |  |  |  |  |
| Effectiveness of a complex, pre-conception intervention to reduce the risk of diabetes by reducing adiposity in young adults in Malaysia: The Jom Mama project - A randomised controlled trial | 10.7189/jogh.12.04053 |  | Not at all relevant |  |  |  |
| Artificial intelligence and machine learning in mobile apps for mental health: A scoping review | 10.1371/journal.pdig.0000079 | Review paper |  |  |  |  |
| A Psychometric Pilot Study Examining the Functions of Suicidal Communications Using IRT and Factor Analysis | 10.3390/ijerph191610081 |  | Not at all relevant |  |  |  |
| Clinical Targets and Attitudes Toward Implementing Digital Health Tools for Remote Measurement in Treatment for Depression: Focus Groups With Patients and Clinicians | 10.2196/38934 |  | Not at all relevant |  |  |  |
| Analyzing the Impact of Mobile App Engagement on Mental Health Outcomes: Secondary Analysis of the Unwinding Anxiety Program | 10.2196/33696 |  | Not at all relevant |  |  |  |
| Cognitive training using a mobile app as a coping tool against COVID-19 distress: A crossover randomized controlled trial | 10.1016/j.jad.2022.05.118 |  | Not at all relevant |  |  |  |
| App-enhanced transdiagnostic CBT for adolescents with mood or psychotic spectrum disorders | 10.1016/j.jad.2022.05.094 |  | Not at all relevant |  |  |  |
| Daily space-time activities, multiple environmental exposures, and anxiety symptoms: A cross-sectional mobile phone-based sensing study | 10.1016/j.scitotenv.2022.155276 |  | Not at all relevant |  |  |  |
| Fostering EFL learners' motivation, anxiety, and self-efficacy through computer-assisted language learning- and mobile-assisted language learning-based instructions | 10.3389/fpsyg.2022.899557 |  | Not at all relevant |  |  |  |
| Efficacy of Smartphone Apps in Patients With Depressive Disorders: A Systematic Review | 10.3389/fpsyt.2022.871966 | Review paper |  |  |  |  |
| Adversity coping capability and its associations with mental health and family wellbeing amid the COVID-19 pandemic in Hong Kong | 10.1186/s12888-022-04198-2 |  | Not at all relevant |  |  |  |
| Acceptability and satisfaction with emma, a smartphone application dedicated to suicide ecological assessment and prevention | 10.3389/fpsyt.2022.952865 |  |  |  | Telehealth |  |
| A deep tensor-based approach for automatic depression recognition from speech utterances | 10.1371/journal.pone.0272659 |  | Not at all relevant |  |  |  |
| Efficacy of attention bias modification via smartphones in a large population sample | 10.1098/rsos.211629 |  | Not at all relevant |  |  |  |
| The mental health burden of racial and ethnic minorities during the COVID-19 pandemic | 10.1371/journal.pone.0271661 |  | Not at all relevant |  |  |  |
| Mitigating Feelings of Loneliness and Depression by Means of Web-Based or Print-Based Physical Activity Interventions: Pooled Analysis of 2 Community-Based Intervention Trials | 10.2196/36515 |  | Not at all relevant |  |  |  |
| The Effect of Mental Health App Customization on Depressive Symptoms in College Students: Randomized Controlled Trial | 10.2196/39516 |  | Not at all relevant |  |  |  |
| Adding an App-Based Intervention to the Cognitive Behavioral Analysis System of Psychotherapy in Routine Outpatient Psychotherapy Treatment: Proof-of-Concept Study | 10.2196/35482 |  | Not at all relevant |  |  |  |
| A Web-Based Application for Personalized Ecological Momentary Assessment in Psychiatric Care: User-Centered Development of the PETRA Application | 10.2196/36430 |  | Not at all relevant |  |  |  |
| Stigma-directed services (Stig2Health) to improve 'linkage to care' for people living with HIV in rural Tanzania: study protocol for a nested pre-post implementation study within the Kilombero and Ulanga Antiretroviral Cohort | 10.12688/aasopenres.13353.2 |  | Not at all relevant |  |  |  |
| Development and validation of UHPLC-MS/MS method for simultaneous quantification of escitalopram and its major metabolites in human plasma and its application in depressed patients | 10.1016/j.jpba.2022.114810 |  | Not at all relevant |  |  |  |
| Safety of low-intensity repetitive transcranial magneTic brAin stimUlation foR people living with mUltiple Sclerosis (TAURUS): study protocol for a randomised controlled trial | 10.1186/s13063-022-06526-z |  | Not at all relevant |  |  |  |
| Parental Efficacy in Managing Smartphone Use of Adolescents with Attention-Deficit/Hyperactivity Disorder: Parental and Adolescent Related Factors | 10.3390/ijerph19159505 |  | Not at all relevant |  |  |  |
| Relationship between Psychological Stress Determined by Voice Analysis and Periodontal Status: A Cohort Study | 10.3390/ijerph19159489 |  | Not at all relevant |  |  |  |
| Prevalence and predictors of nomophobia among the general population in two middle eastern countries | 10.1186/s12888-022-04168-8 |  | Not at all relevant |  |  |  |
| A Biofeedback-Based Mobile App With Serious Games for Young Adults With Anxiety in the United Arab Emirates: Development and Usability Study | 10.2196/36936 |  | Not at all relevant |  |  |  |
| Fächerübergreifende psychokardiologische Rehabilitation: vom Modellprojekt zur Blaupause des dualen Rehabilitationskonzeptes – von Erfahrungswerten zu wissenschaftlichen Daten | 10.1055/a-1866-6781 |  | Not at all relevant |  |  |  |
| The Effects of Smartphone Use on Life Satisfaction in Older Adults: The Mediating Role of Depressive Symptoms | 10.1097/01.NCN.0000872004.67999.f5 |  | Not at all relevant |  |  |  |
| The Effects of Smartphone Use on Life Satisfaction in Older Adults: The Mediating Role of Depressive Symptoms | 10.1097/CIN.0000000000000867 |  | Not at all relevant |  |  |  |
| Transdiagnostic Psychopathology in a Help-Seeking Population of an Early Recognition Center for Mental Disorders: Protocol for an Experience Sampling Study | 10.2196/35206 |  | Not at all relevant |  |  |  |
| Service Demand for and Awareness of a Primary Healthcare Pilot Project for People With Disabilities | 10.3346/jkms.2022.37.e241 |  | Not at all relevant |  |  |  |
| A new model in medicine education: smart model education set | 10.1007/s00276-022-02989-6 |  | Not at all relevant |  |  |  |
| Assessment of blinding in randomized controlled trials of antidepressants for depressive disorders 2000-2020: A systematic review and meta-analysis | 10.1016/j.eclinm.2022.101505 | Review paper |  |  |  |  |
| Effects of Tai Chi App and Facebook health education programs on breast cancer survivors' stress and quality of life in the Era of pandemic | 10.1016/j.ctcp.2022.101621 |  | Not at all relevant |  |  |  |
| Effectiveness and cost-effectiveness for the treatment of depressive symptoms in refugees and asylum seekers: A multi-centred randomized controlled trial | 10.1016/j.lanepe.2022.100413 |  | Not at all relevant |  |  |  |
| Predictors of engagement with remote sensing technologies for symptom measurement in Major Depressive Disorder | 10.1016/j.jad.2022.05.005 |  | Not at all relevant |  |  |  |
| Smartphone-based Ecological Momentary Assessment to study "scanxiety" among Adolescent and Young Adult survivors of childhood cancer: A feasibility study | 10.1002/pon.5935 |  | Not at all relevant |  |  |  |
| Do you see what I mean?: Using mobile eye tracking to capture parent-child dynamics in the context of anxiety risk | 10.1017/S0954579420001601 |  | Not at all relevant |  |  |  |
| An intervention to reduce stigma and improve management of depression, risk of suicide/self-harm and other significant emotional or medically unexplained complaints among adolescents living in urban slums: protocol for the ARTEMIS project | 10.1186/s13063-022-06539-8 |  | Not at all relevant |  |  |  |
| Fostering Nursing Staff Competence in Personal Protective Equipment Education during COVID-19: A Mobile-Video Online Learning Approach | 10.3390/ijerph19159238 |  | Not at all relevant |  |  |  |
| Effects of a Gamified, Behavior Change Technique-Based Mobile App on Increasing Physical Activity and Reducing Anxiety in Adults With Autism Spectrum Disorder: Feasibility Randomized Controlled Trial | 10.2196/35701 |  | Not at all relevant |  |  |  |
| App-based intervention among adolescents with persistent pain: a pilot feasibility randomized controlled trial | 10.1186/s40814-022-01113-0 |  | Not at all relevant |  |  |  |
| Assessing an Internet-Delivered, Emotion-Focused Intervention Compared With a Healthy Lifestyle Active Control Intervention in Improving Mental Health in Cancer Survivors: Protocol for a Randomized Controlled Trial | 10.2196/36658 |  | Not at all relevant |  |  |  |
| Characterizing veteran and PTSD service dog teams: Exploring potential mechanisms of symptom change and canine predictors of efficacy | 10.1371/journal.pone.0269186 |  | Not at all relevant |  |  |  |
| Digital phenotype of mood disorders: A conceptual and critical review | 10.3389/fpsyt.2022.895860 | Review paper |  |  |  |  |
| Mobile Health Applications for Depression in China: A Systematic Review | 10.7759/cureus.27299 | Review paper |  |  |  |  |
| A mobile health + health coaching application for the management of chronic non-cancer pain in older adults: Results from a pilot randomized controlled study | 10.3389/fpain.2022.921428 |  | Not at all relevant |  |  |  |
| Machine Learning for Anxiety Detection Using Biosignals: A Review | 10.3390/diagnostics12081794 | Review paper |  |  |  |  |
| Feasibility, engagement, and preliminary clinical outcomes of a digital biodata-driven intervention for anxiety and depression | 10.3389/fdgth.2022.868970 |  | Not at all relevant |  |  |  |
| Appsolutely secure? Psychometric properties of the German version of an app information privacy concerns measure during COVID-19 | 10.3389/fpsyg.2022.899092 |  | Not at all relevant |  |  |  |
| Mental Health Risk Factors and Coping Strategies among Students in Asia Pacific during COVID-19 Pandemic-A Scoping Review | 10.3390/ijerph19158894 | Review paper |  |  |  |  |
| Evaluating the Efficacy of a Guided and Unguided Internet-Based Self-help Intervention for Chronic Loneliness: Protocol for a 3-Arm Randomized Controlled Trial | 10.2196/36358 |  | Not at all relevant |  |  |  |
| Association Between Care Modality and Use With Treatment Response Among Members Accessing Virtual Mental Health Services: Real-world Observational Study | 10.2196/36956 |  | Not at all relevant |  |  |  |
| Ad hoc Setup of an Online Mental Health Self-Help Program During the COVID-19 Pandemic: Description of the Development and Implementation Processes and Analysis of Its Users' and Usage Profiles | 10.3389/fpsyg.2022.853371 |  | Not at all relevant |  |  |  |
| Using a Brief Mental Imagery Competing Task to Reduce the Number of Intrusive Memories: Exploratory Case Series With Trauma-Exposed Women | 10.2196/37382 |  | Not at all relevant |  |  |  |
| Public Attitudes Regarding Trade-offs Between the Functional Aspects of a Contact-Confirming App for COVID-19 Infection Control and the Benefits to Individuals and Public Health: Cross-sectional Survey | 10.2196/37720 |  | Not at all relevant |  |  |  |
| Prescribing Time in Nature for Human Health and Well-Being: Study Protocol for Tailored Park Prescriptions | 10.3389/fdgth.2022.932533 |  | Not at all relevant |  |  |  |
| Experience of using a smartphone WeChat applet for dental anxiety assessment and preoperative evaluation: A nationwide multicenter study | 10.3389/fpubh.2022.900899 |  | Not at all relevant |  |  |  |
| Grouping of mood symptoms by time series dynamics | 10.1016/j.jad.2022.04.117 |  | Not at all relevant |  |  |  |
| A Golden Thread approach to transforming Maternal and Child Health in Singapore | 10.1186/s12884-022-04893-8 |  | Not at all relevant |  |  |  |
| Digital Health in Children's Oral and Dental Health: An Overview and a Bibliometric Analysis | 10.3390/children9071039 |  | Not at all relevant |  |  |  |
| Mild-to-moderate COVID-19 impact on the cardiorespiratory fitness in young and middle-aged populations | 10.1590/1414-431X2022e12118 |  | Not at all relevant |  |  |  |
| Automated app-based augmented reality cognitive behavioral therapy for spider phobia: Study protocol for a randomized controlled trial | 10.1371/journal.pone.0271175 |  | Not at all relevant |  |  |  |
| The Effect of Video-Based Preanaesthetic Preparation Versus Conventional Approach on Parental Anxiety in Paediatric Dental Procedures: A Prospective Cohort Study | 10.7759/cureus.26768 |  | Not at all relevant |  |  |  |
| Proximal Risk for Suicide: Protocol for an Ecological Momentary Assessment Study | 10.2196/37583 |  | Not at all relevant |  |  |  |
| Improving Well-being With a Mobile Artificial Intelligence-Powered Acceptance Commitment Therapy Tool: Pragmatic Retrospective Study | 10.2196/36018 |  | Not at all relevant |  |  |  |
| Health indicators and poor health dynamics during COVID-19 pandemic | 10.1007/s12144-022-03425-z |  | Not at all relevant |  |  |  |
| Mobile rehabilitation support versus usual care in patients after total hip or knee arthroplasty: study protocol for a randomised controlled trial | 10.1186/s13063-022-06269-x |  | Not at all relevant |  |  |  |
| Web-Based Psychological Interventions for People Living With and Beyond Cancer: Meta-Review of What Works and What Does Not for Maximizing Recruitment, Engagement, and Efficacy | 10.2196/36255 | Review paper |  |  |  |  |
| Preliminary Real-World Evidence Supporting the Efficacy of a Remote Neurofeedback System in Improving Mental Health: Retrospective Single-Group Pretest-Posttest Study | 10.2196/35636 |  | Not at all relevant |  |  |  |
| Acoustic and Linguistic Features of Impromptu Speech and Their Association With Anxiety: Validation Study | 10.2196/36828 |  | Not at all relevant |  |  |  |
| Suicidal Behaviour, including Ideation and Self-Harm, in Young Migrants: A Systematic Review | 10.3390/ijerph19148329 | Review paper |  |  |  |  |
| Spanish Adaptation of Meaning-Centered Psychotherapy for Participants With Cancer: Study Protocol of a Randomized Control Trial | 10.3389/fpsyt.2022.892573 |  | Not at all relevant |  |  |  |
| Have the COVID-19 pandemic and lockdown affected children's mental health in the long term? A repeated cross-sectional study | 10.1136/bmjopen-2021-058609 |  | Not at all relevant |  |  |  |
| A German Smartphone-Based Self-management Tool for Psoriasis: Community-Driven Development and Evaluation of Quality-of-Life Effects | 10.2196/32593 |  | Not at all relevant |  |  |  |
| Idiopathic Atrophoderma of Pasini and Pierini: A Case Report and Literature Review | 10.7759/cureus.26571 | Review paper |  |  |  |  |
| Relationship between Nomophobia, Various Emotional Difficulties, and Distress Factors among Students | 10.3390/ejihpe12070053 |  | Not at all relevant |  |  |  |
| Improvements in Depression Outcomes Following a Digital Cognitive Behavioral Therapy Intervention in a Polychronic Population: Retrospective Study | 10.2196/38005 |  | Not at all relevant |  |  |  |
| A Remote Assessment of Anxiety on Young People: Towards Their Views and Their Different Pet Interaction | 10.3390/healthcare10071242 |  | Not at all relevant |  |  |  |
| What drives older adults' use of mobile registration apps in Taiwan? An investigation using the extended UTAUT model | 10.1080/17538157.2021.1990299 |  | Not at all relevant |  |  |  |
| Depression Diagnosis and Forecast based on Mobile Phone Sensor Data | 10.1109/EMBC48229.2022.9871255 |  | Not at all relevant |  |  |  |
| Journaling Data for Daily PHQ-2 Depression Prediction and Forecasting | 10.1109/EMBC48229.2022.9871015 |  | Not at all relevant |  |  |  |
| Design and Methods of a Prospective Smartphone App-Based Study for Digital Phenotyping of Mood and Anxiety Symptoms Mixed With Centralized and Decentralized Research Form: The Search Your Mind (S.Y.M., ) Project | 10.30773/pi.2022.0102 |  | Not at all relevant |  |  |  |
| An mHealth Platform for Augmenting Behavioral Health in Primary Care: Longitudinal Feasibility Study | 10.2196/36021 |  | Not at all relevant |  |  |  |
| Using Technology to Promote Therapist Use of Exposure Therapy for Childhood Anxiety Disorders: A Randomized Pilot Study | 10.1016/j.beth.2022.01.010 |  | Not at all relevant |  |  |  |
| Longitudinal Cross-Lagged Analysis Between Mobile Phone Dependence, Friendships, and Depressive Symptoms Among Korean Adolescents | 10.1089/cyber.2022.0015 |  | Not at all relevant |  |  |  |
| Heal-me PiONEer (personalized online nutrition and exercise): An RCT assessing 2 levels of app-based programming in individuals with chronic disease | 10.1016/j.cct.2022.106791 |  | Not at all relevant |  |  |  |
| Rates of self-reported postpartum depressive symptoms in the United States before and after the start of the COVID-19 pandemic | 10.1016/j.jpsychires.2022.04.011 |  | Not at all relevant |  |  |  |
| A Mindfulness Application for Reducing Prenatal Stress | 10.1111/jmwh.13359 |  | Not at all relevant |  |  |  |
| Internet- and mobile-based intervention for depression in adults with chronic back pain: A health economic evaluation | 10.1016/j.jad.2022.04.004 |  | Not at all relevant |  |  |  |
| A Pilot Feasibility Study of Digital Health Coaching for Men With Prostate Cancer | 10.1200/OP.21.00712 |  | Not at all relevant |  |  |  |
| Effectiveness of mobile applications for patients with severe mental illness: A meta-analysis of randomized controlled trials | 10.1111/jjns.12476 |  | Not at all relevant |  |  |  |
| Enhancing Problem-Solving Therapy With Smartphone Technology: A Pilot Randomized Controlled Trial | 10.1176/appi.ps.201900254 |  | Not at all relevant |  |  |  |
| The role of mood state and emotion regulation in the discrepancy between gastrointestinal symptom burden recorded prospectively and via recall questionnaire | 10.1111/nmo.14304 |  | Not at all relevant |  |  |  |
| Are Mental Health Apps Adequately Equipped to Handle Users in Crisis? | 10.1027/0227-5910/a000785 |  | Not at all relevant |  |  |  |
| Longitudinal Impact of the myPlan App on Health and Safety Among College Women Experiencing Partner Violence | 10.1177/0886260521991880 |  | Not at all relevant |  |  |  |
| Levels of depression, anxiety, and psychological distress among Ugandan adults during the first wave of the COVID-19 pandemic: cross-sectional evidence from a mobile phone-based population survey | 10.1017/gmh.2022.28 |  | Not at all relevant |  |  |  |
| Prevalence of the risk of depression and worry in pregnant women in the context of the COVID-19 pandemic in Antioquia, Colombia, 2020-2021 | 10.18597/rcog.3821 |  | Not at all relevant |  |  |  |
| Technology-assisted peer therapy: a new way of delivering evidence-based psychological interventions | 10.1186/s12913-022-08233-6 |  | Not at all relevant |  |  |  |
| Patient's knowledge of daily activities, need for information and quality of life after cardiac electronic device implantation | 10.24425/fmc.2022.141695 |  | Not at all relevant |  |  |  |
| Using mHealth Technologies to Promote Public Health and Well-Being in Urban Areas with Blue-Green Solutions | 10.3233/SHTI220791 |  | Not at all relevant |  |  |  |
| The relationship between screen time exposure and the presence of anxiety-related disorders among adolescents during the COVID-19 pandemic: A cross-sectional study | 10.33546/bnj.2058 |  | Not at all relevant |  |  |  |
| Long-term Effects of a Social Media-Based Intervention (Run4Love) on Depressive Symptoms of People Living With HIV: 3-Year Follow-up of a Randomized Controlled Trial | 10.2196/36809 |  | Not at all relevant |  |  |  |
| Examining Social Media Experiences and Attitudes Toward Technology-Based Interventions for Reducing Social Isolation Among LGBTQ Youth Living in Rural United States: An Online Qualitative Study | 10.3389/fdgth.2022.900695 |  | Not at all relevant |  |  |  |
| Depressive Symptoms Feature-Based Machine Learning Approach to Predicting Depression Using Smartphone | 10.3390/healthcare10071189 |  | Not at all relevant |  |  |  |
| A Machine Learning Approach for Predicting Non-Suicidal Self-Injury in Young Adults | 10.3390/s22134790 |  | Not at all relevant |  |  |  |
| The effectiveness of e-healthcare interventions for mental health of nurses: A PRISMA-compliant systematic review of randomized controlled trials | 10.1097/MD.0000000000029125 | Review paper |  |  |  |  |
| Predicting Depression in Adolescents Using Mobile and Wearable Sensors: Multimodal Machine Learning-Based Exploratory Study | 10.2196/35807 |  | Not at all relevant |  |  |  |
| Efficacy, efficiency and safety of a cardiac telerehabilitation programme using wearable sensors in patients with coronary heart disease: the TELEWEAR-CR study protocol | 10.1136/bmjopen-2021-059945 |  | Not at all relevant |  |  |  |
| Alleviating Pregastroscopy Anxiety Using Mobile Social Media Application | 10.3389/fmed.2022.855892 |  | Not at all relevant |  |  |  |
| Correction: One-year follow-up of functional impairment in inpatients with mood and anxiety disorders - Potentials of the Mini-ICF-APP | 10.1186/s12888-022-04051-6 |  | Not at all relevant |  |  |  |
| Predictors of Disengagement and Symptom Improvement Among Adults With Depression Enrolled in Talkspace, a Technology-Mediated Psychotherapy Platform: Naturalistic Observational Study | 10.2196/36521 |  | Not at all relevant |  |  |  |
| Perceptions of Smartphone App Use among Mothers Raising Young Children | 10.3390/ijerph19137585 |  | Not at all relevant |  |  |  |
| Distinguishing the Effect of Time Spent at Home during COVID-19 Pandemic on the Mental Health of Urban and Suburban College Students Using Cell Phone Geolocation | 10.3390/ijerph19127513 |  | Not at all relevant |  |  |  |
| Protocol for process evaluation of SMART Mental Health cluster randomised control trial: an intervention for management of common mental disorders in India | 10.1136/bmjopen-2021-058669 |  | Not at all relevant |  |  |  |
| Advancing Posttraumatic Stress Disorder Diagnosis and the Treatment of Trauma in Humanitarian Emergencies via Mobile Health: Protocol for a Proof-of-Concept Nonrandomized Controlled Trial | 10.2196/38223 |  | Not at all relevant |  |  |  |
| Exposure Detection Applications Acceptance: The Case of COVID-19 | 10.3390/ijerph19127307 |  | Not at all relevant |  |  |  |
| Effect of smartphone app-based health care intervention for health management of high-risk mothers: a study protocol for a randomized controlled trial | 10.1186/s13063-022-06425-3 |  | Not at all relevant |  |  |  |
| Exploring association of mobile phone access with positive health outcomes and behaviors amongst post-partum mothers in rural Malawi | 10.1186/s12884-022-04782-0 |  | Not at all relevant |  |  |  |
| Feasibility and Acceptability of Internet-Based Interpersonal Psychotherapy for Stress, Anxiety, and Depression in Prenatal Women: Thematic Analysis | 10.2196/23879 |  | Not at all relevant |  |  |  |
| [FiD: a smartphone application for anxiety assessment: two weeks study] | 10.22365/jpsych.2021.037 |  | Not at all relevant |  |  |  |
| A novel solid phase extraction sample preparation method for sensitively determining doxepin and N-nordoxepin in human plasma and its application in a bioequivalence study in healthy Chinese volunteers | 10.1039/d2ay00129b |  | Not at all relevant |  |  |  |
| Neuropathological and behavioral features of an APP/PS1/MAPT (6xTg) transgenic model of Alzheimer's disease | 10.1186/s13041-022-00933-8 |  | Not at all relevant |  |  |  |
| Sociodemographic Characteristics Associated With an eHealth System Designed to Reduce Depressive Symptoms Among Patients With Breast or Prostate Cancer: Prospective Study | 10.2196/33734 |  | Not at all relevant |  |  |  |
| Feasibility, Acceptability, and Preliminary Efficacy of an App-Based Meditation Intervention to Decrease Firefighter Psychological Distress and Burnout: A One-Group Pilot Study | 10.2196/34951 |  | Not at all relevant |  |  |  |
| Mental Well-Being and Sexual Intimacy among Men and Gender Diverse People Who Have Sex with Men during the First UK COVID-19 Lockdown: A Mixed-Methods Study | 10.3390/ijerph19126985 |  | Not at all relevant |  |  |  |
| Analysis on the Cognitive Impact of Social Mobile Games on Left-Behind Children in the Era of Big Data | 10.3389/fpubh.2022.915801 |  | Not at all relevant |  |  |  |
| Mobile Health Applications for Postpartum Depression Management: A Theory-Informed Analysis of Change-Use-Engagement (CUE) Criteria in the Digital Environment | 10.3233/SHTI220198 |  | Not at all relevant |  |  |  |
| Protocol for Rhapsody: a longitudinal observational study examining the feasibility of speech phenotyping for remote assessment of neurodegenerative and psychiatric disorders | 10.1136/bmjopen-2022-061193 |  | Not at all relevant |  |  |  |
| Similarity matrix-based anomaly detection for clinical intervention | 10.1038/s41598-022-12792-3 |  | Not at all relevant |  |  |  |
| Understanding the Relationship Between Mood Symptoms and Mobile App Engagement Among Patients With Breast Cancer Using Machine Learning: Case Study | 10.2196/30712 |  | Not at all relevant |  |  |  |
| Outcome Measure Harmonization and Data Infrastructure for Patient-Centered Outcomes Research in Depression: Final Report | 10.23970/AHRQEPCWHITEPAPERDEPRESSIONFINAL |  | Not at all relevant |  |  |  |
| Effectiveness of Telehealth for Women’s Preventive Services | 10.23970/AHRQEPCCER256 |  | Not at all relevant |  |  |  |
| Usability of a smartwatch for atrial fibrillation detection in older adults after stroke | 10.1016/j.cvdhj.2022.03.003 |  | Not at all relevant |  |  |  |
| The NEVERMIND e-health system in the treatment of depressive symptoms among patients with severe somatic conditions: A multicentre, pragmatic randomised controlled trial | 10.1016/j.eclinm.2022.101423 |  | Not at all relevant |  |  |  |
| A digital therapeutic for management of psychosocial aspects of psoriasis: A pre-post proof of concept study | 10.1002/ski2.103 |  | Not at all relevant |  |  |  |
| Predictors of adverse pregnancy outcomes among Kenyan women with HIV on antiretroviral treatment in pregnancy | 10.1097/QAD.0000000000003215 |  | Not at all relevant |  |  |  |
| Differences in mobility patterns according to machine learning models in patients with bipolar disorder and patients with unipolar disorder | 10.1016/j.jad.2022.03.054 |  | Not at all relevant |  |  |  |
| Understanding the association between reappraisal use and depressive symptoms during adolescence: the moderating influence of regulatory success | 10.1080/02699931.2022.2043245 |  | Not at all relevant |  |  |  |
| The Effect of M-Health-Based Core Stability Exercise Combined with Self-Compassion Training for Patients with Nonspecific Chronic Low Back Pain: A Randomized Controlled Pilot Study | 10.1007/s40122-022-00358-0 |  | Not at all relevant |  |  |  |
| The psychological reassurance effect of mobile tracing apps in Covid-19 Era | 10.1016/j.chb.2022.107210 |  | Not at all relevant |  |  |  |
| SlowMo therapy, a new digital blended therapy for fear of harm from others: An account of therapy personalisation within a targeted intervention | 10.1111/papt.12377 |  | Not at all relevant |  |  |  |
| Mobile application for couple relationships: Results of a pilot effectiveness study | 10.1111/famp.12733 |  | Not at all relevant |  |  |  |
| Psychological helpline in response to the COVID-19 pandemic in the Dominican Republic | 10.1002/capr.12482 |  | Not at all relevant |  |  |  |
| Interpretation Bias and Anticipated Distress in the Face of Ambiguity: Predictors of Change in Cognitive Behavioral Therapy for Youth Anxiety | 10.1007/s10578-021-01147-0 |  | Not at all relevant |  |  |  |
| A Study on the Effect of the Pre-Go-Live Training in Anxiety and Depression of Medical Staff Based on the Data of Wuhan Fangcang Shelter Hospital During COVID-19 in the Era of Big Data | 10.3389/fpubh.2022.909241 |  | Not at all relevant |  |  |  |
| The effect of a therapeutic smartphone application on suicidal ideation in young adults: Findings from a randomized controlled trial in Australia | 10.1371/journal.pmed.1003978 |  | Not at all relevant |  |  |  |
| Pilot Testing in the Wild: Feasibility, Acceptability, Usage Patterns, and Efficacy of an Integrated Web and Smartphone Platform for Bipolar II Disorder | 10.2196/32740 |  | Not at all relevant |  |  |  |
| Maternal Prenatal Inflammation Increases Brain Damage Susceptibility of Lipopolysaccharide in Adult Rat Offspring via COX-2/PGD-2/DPs Pathway Activation | 10.3390/ijms23116142 |  | Not at all relevant |  |  |  |
| Three-arm randomised controlled trial of an m-health app and digital engagement strategy for improving treatment adherence and reducing suicidal ideation in young people: study protocol | 10.1136/bmjopen-2021-058584 |  | Not at all relevant |  |  |  |
| An Integrated mHealth App for Smoking Cessation in Black Smokers With Anxiety: Protocol for a Randomized Controlled Trial | 10.2196/38905 |  | Not at all relevant |  |  |  |
| The Relationship between Duration of Smartphone Uses and Anxiety in University Students during the COVID-19 Outbreak | 10.3390/ijerph19116620 |  | Not at all relevant |  |  |  |
| What criteria are young people using to select mobile mental health applications? A nominal group study | 10.1177/20552076221102775 |  | Not at all relevant |  |  |  |
| Diurnal dynamics of stress and mood during COVID-19 lockdown: a large multinational ecological momentary assessment study | 10.1098/rspb.2021.2480 |  | Not at all relevant |  |  |  |
| Effectiveness of home-based cardiac telerehabilitation as an alternative to Phase 2 cardiac rehabilitation of coronary heart disease: a systematic review and meta-analysis | 10.1093/eurjpc/zwab106 | Review paper |  |  |  |  |
| A Mobile App for Stress Management in Middle-Aged Men and Women (Calm): Feasibility Randomized Controlled Trial | 10.2196/30294 |  | Not at all relevant |  |  |  |
| Digital screening for postnatal depression: mixed methods proof-of-concept study | 10.1186/s12884-022-04756-2 |  | Not at all relevant |  |  |  |
| Key Drivers and Facilitators of the Choice to Use mHealth Technology in People With Neurological Conditions: Observational Study | 10.2196/29509 |  | Not at all relevant |  |  |  |
| Adolescent Health Promotion Interventions Using Well-Care Visits and a Smartphone Cognitive Behavioral Therapy App: Randomized Controlled Trial | 10.2196/34154 |  | Not at all relevant |  |  |  |
| Adherence and Engagement With a Cognitive Behavioral Therapy-Based Conversational Agent (Wysa for Chronic Pain) Among Adults With Chronic Pain: Survival Analysis | 10.2196/37302 |  | Not at all relevant |  |  |  |
| "Who needs an app? Fertility patients' use of a novel mobile health app" | 10.1177/20552076221102248 |  | Not at all relevant |  |  |  |
| Effects of Smartphone Overdependence and the Quality of Friendship on Depression among High School Students | 10.1155/2022/3932326 |  | Not at all relevant |  |  |  |
| Correction: A Mobile-Based Intervention to Increase Self-esteem in Students with Depressive Symptoms: Randomized Controlled Trial | 10.2196/39448 |  | Not at all relevant |  |  |  |
| A Microanalysis of Mood and Self-Reported Functionality in Stroke Patients Using Ecological Momentary Assessment | 10.3389/fneur.2022.854777 |  | Not at all relevant |  |  |  |
| Efficacy of a Combined Acceptance and Commitment Intervention to Improve Psychological Flexibility and Associated Symptoms in Cancer Patients: Study Protocol for a Randomized Controlled Trial | 10.3389/fpsyg.2022.871929 |  | Not at all relevant |  |  |  |
| Pet Presence Can Reduce Anxiety in the Elderly: The Italian Experience during COVID-19 Lockdown Assessed by an Electronic Survey | 10.3390/ijerph19106135 |  | Not at all relevant |  |  |  |
| The Effectiveness of the Digital Environment and Perfectionism on Anxiety and Depression in the Light of the COVID-19 Pandemic in Northern Iraq | 10.3389/fpsyg.2022.804071 |  | Not at all relevant |  |  |  |
| Effect of "Mehrpishegan" web-based support group on depression, anxiety, and stress among elderly informal caregivers: a protocol for a randomized-controlled trial | 10.1186/s13063-022-06351-4 |  | Not at all relevant |  |  |  |
| Are All Urban Parks Robust to the COVID-19 Pandemic? Focusing on Type, Functionality, and Accessibility | 10.3390/ijerph19106062 |  | Not at all relevant |  |  |  |
| Early Detection of Neurodevelopmental Disorders of Toddlers and Postnatal Depression by Mobile Health App: Observational Cross-sectional Study | 10.2196/38181 |  | Not at all relevant |  |  |  |
| Evaluation of the Effect of Patient Education and Strengthening Exercise Therapy Using a Mobile Messaging App on Work Productivity in Japanese Patients With Chronic Low Back Pain: Open-Label, Randomized, Parallel-Group Trial | 10.2196/35867 |  | Not at all relevant |  |  |  |
| SERO - A New Mobile App for Suicide Prevention | 10.3233/SHTI220310 |  |  |  | Telehealth |  |
| A Machine Learning Approach for Detecting Digital Behavioral Patterns of Depression Using Nonintrusive Smartphone Data (Complementary Path to Patient Health Questionnaire-9 Assessment): Prospective Observational Study | 10.2196/37736 |  | Not at all relevant |  |  |  |
| One-year follow-up of functional impairment in inpatients with mood and anxiety disorders - Potentials of the Mini-ICF-APP | 10.1186/s12888-022-03977-1 |  | Not at all relevant |  |  |  |
| MindKind: A mixed-methods protocol for the feasibility of global digital mental health studies in young people | 10.12688/wellcomeopenres.17167.2 |  | Not at all relevant |  |  |  |
| Platelet TAU is Associated with Changes in Depression and Alzheimer's Disease | 10.31083/j.fbl2705153 |  | Not at all relevant |  |  |  |
| The Effects of a Web-Based Tool for Parents of Children With Juvenile Idiopathic Arthritis: Randomized Controlled Trial | 10.2196/29787 |  | Not at all relevant |  |  |  |
| Digital Health Interventions for Delivery of Mental Health Care: Systematic and Comprehensive Meta-Review | 10.2196/35159 | Review paper |  |  |  |  |
| Exploring Use Patterns and Racial and Ethnic Differences in Real Time Affective States During Social Media Use Among a Clinical Sample of Adolescents With Depression: Prospective Cohort Study | 10.2196/30900 |  | Not at all relevant |  |  |  |
| Transtheoretical model-based mobile health application for PCOS | 10.1186/s12978-022-01422-w |  | Not at all relevant |  |  |  |
| Curiosity for information predicts wellbeing mediated by loneliness during COVID-19 pandemic | 10.1038/s41598-022-11924-z |  | Not at all relevant |  |  |  |
| A rapid and sensitive LC-MS/MS method for determination of the active component K6 in serum of patients with depression | 10.1016/j.jpba.2022.114691 |  | Not at all relevant |  |  |  |
| Characterization of labyrinth emitter-clogging substances in biogas slurry drip irrigation systems | 10.1016/j.scitotenv.2022.153315 |  | Not at all relevant |  |  |  |
| The Use of an Electronic Painting Platform by Family Caregivers of Persons with Dementia: A Feasibility and Acceptability Study | 10.3390/healthcare10050870 |  | Not at all relevant |  |  |  |
| Online Health Information Seeking for Self and Child: An Experimental Study of Parental Symptom Search | 10.2196/29618 |  | Not at all relevant |  |  |  |
| Making remote measurement technology work in multiple sclerosis, epilepsy and depression: survey of healthcare professionals | 10.1186/s12911-022-01856-z |  | Not at all relevant |  |  |  |
| Users' satisfaction levels about mHealth applications in post-Covid-19 times in Saudi Arabia | 10.1371/journal.pone.0267002 |  | Not at all relevant |  |  |  |
| Smartphone Sensor Data for Identifying and Monitoring Symptoms of Mood Disorders: A Longitudinal Observational Study | 10.2196/35549 |  | Not at all relevant |  |  |  |
| Mhealth interventions to improve health and quality of life related outcomes for informal dementia caregivers: A scoping review | 10.1080/10400435.2020.1829174 | Review paper |  |  |  |  |
| Effect of a Virtual Reality Contact-Based Educational Intervention on the Public Stigma of Depression: Randomized Controlled Pilot Study | 10.2196/28072 |  | Not at all relevant |  |  |  |
| Effects of psychiatric symptoms, age, and gender on fear of missing out (FoMO) and problematic smartphone use: A path analysis with clinical-based adolescent sample | 10.4103/indianjpsychiatry.indianjpsychiatry_34_21 |  | Not at all relevant |  |  |  |
| Implementation of a Mobile Technology-Supported Diaphragmatic Breathing Intervention in Military mTBI With PTSD | 10.1097/HTR.0000000000000774 |  | Not at all relevant |  |  |  |
| Factors Affecting Women's Participation in Breast Cancer Screening in Turkey | 10.31557/APJCP.2022.23.5.1627 |  | Not at all relevant |  |  |  |
| You are not alone: Smartphone use, friendship satisfaction, and anxiety during the COVID-19 crisis | 10.1177/20501579211051820 |  | Not at all relevant |  |  |  |
| Usability and Acceptability of the QuestLeukemia Mobile Application: A Pilot Study for An Educational and Psychological Intervention for Children with Chronic Illnesses | 10.1177/27527530221068422 |  | Not at all relevant |  |  |  |
| Children's shyness and frontal electroencephalogram delta-beta correlation in the pediatric surgical setting | 10.1002/dev.22275 |  | Not at all relevant |  |  |  |
| Automaticity and depression: Daily mood-reactive rumination in people with and without depression history | 10.1037/abn0000752 |  | Not at all relevant |  |  |  |
| Food insecurity, depressive symptoms, and the salience of gendered family roles during the COVID-19 pandemic in South Africa | 10.1016/j.socscimed.2022.114830 |  | Not at all relevant |  |  |  |
| An integrated early care pathway for autism | 10.1016/S2352-4642(22)00037-2 |  | Not at all relevant |  |  |  |
| Real-world feasibility and acceptability of real-time suicide risk monitoring via smartphones: A 6-month follow-up cohort | 10.1016/j.jpsychires.2022.02.026 |  | Not at all relevant |  |  |  |
| Geolocation features differentiate healthy from remitted depressed adults | 10.1037/abn0000742 |  | Not at all relevant |  |  |  |
| Icarisid II rescues cognitive dysfunction via activation of Wnt/β-catenin signaling pathway promoting hippocampal neurogenesis in APP/PS1 transgenic mice | 10.1002/ptr.7430 |  | Not at all relevant |  |  |  |
| Evaluation of the effectiveness of a mobile application in the management of dental anxiety: A randomised controlled trial | 10.1111/joor.13311 |  | Not at all relevant |  |  |  |
| Anxiety, home blood pressure monitoring, and cardiovascular events among older hypertension patients during the COVID-19 pandemic | 10.1038/s41440-022-00852-0 |  | Not at all relevant |  |  |  |
| Chronic exposure to environmentally relevant concentrations of guanylurea induces neurotoxicity of Danio rerio adults | 10.1016/j.scitotenv.2022.153095 |  | Not at all relevant |  |  |  |
| Protocol for a pilot randomized controlled trial of a mobile health exercise intervention for older patients with myeloid neoplasms (GO-EXCAP 2) | 10.1016/j.jgo.2021.12.011 |  | Not at all relevant |  |  |  |
| An Emotion Regulation Tablet App for Middle-Aged and Older Adults at High Suicide Risk: Feasibility, Acceptability, and Two Case Studies | 10.1016/j.jagp.2021.08.015 |  | Not at all relevant |  |  |  |
| COVID-19 Pandemic and Exercise (COPE) trial: a multigroup pragmatic randomised controlled trial examining effects of app-based at-home exercise programs on depressive symptoms | 10.1136/bjsports-2021-104379 |  | Not at all relevant |  |  |  |
| Thwarted Belongingness Mediates Interpersonal Stress and Suicidal Thoughts: An Intensive Longitudinal Study with High-risk Adolescents | 10.1080/15374416.2021.1969654 |  | Not at all relevant |  |  |  |
| Exploring differential item functioning on eating disorder measures by food security status | 10.1007/s40519-021-01289-z |  | Not at all relevant |  |  |  |
| Application of computerized cognitive test battery in major depressive disorder: a narrative literature review | 10.1080/08039488.2021.1965654 | Review paper |  |  |  |  |
| Cognitive bias modification for threat interpretations: using passive Mobile Sensing to detect intervention effects in daily life | 10.1080/10615806.2021.1959916 |  | Not at all relevant |  |  |  |
| Cognitive processes in autism: Repetitive thinking in autistic versus non-autistic adults | 10.1177/13623613211034380 |  | Not at all relevant |  |  |  |
| Toward Technology-Based Education and English as a Foreign Language Motivation: A Review of Literature | 10.3389/fpsyg.2022.870540 | Review paper |  |  |  |  |
| Acceptability and feasibility of a pilot randomized controlled trial of Narrative e-Writing Intervention (NeW-I) for parent-caregivers of children with chronic life-threatening illnesses in Singapore | 10.1186/s12904-022-00945-0 |  | Not at all relevant |  |  |  |
| Occupational sitting time, its determinants and intervention strategies in Malaysian office workers: a mixed-methods study | 10.1093/heapro/daab149 |  | Not at all relevant |  |  |  |
| mHealth Solutions for Mental Health Screening and Diagnosis: A Review of App User Perspectives Using Sentiment and Thematic Analysis | 10.3389/fpsyt.2022.857304 | Review paper |  |  |  |  |
| Predicting perinatal health outcomes using smartphone-based digital phenotyping and machine learning in a prospective Swedish cohort (Mom2B): study protocol | 10.1136/bmjopen-2021-059033 |  | Not at all relevant |  |  |  |
| The Effect of Transcranial Alternating Current Stimulation With Cognitive Training on Executive Brain Function in Individuals With Dementia: Protocol for a Crossover Randomized Controlled Trial | 10.2196/37282 |  | Not at all relevant |  |  |  |
| Live Video Mind-Body Program for Patients With Knee Osteoarthritis, Comorbid Depression, and Obesity: Development and Feasibility Pilot Study | 10.2196/34654 |  | Not at all relevant |  |  |  |
| Impact of a Digital Lifestyle Intervention on Diabetes Self-Management: A Pilot Study | 10.3390/nu14091810 |  | Not at all relevant |  |  |  |
| Phubbing among Lebanese young adults: Scale validation and association with mental health (depression, anxiety, and stress) | 10.1007/s12144-022-03104-z |  | Not at all relevant |  |  |  |
| My Grief App for Prolonged Grief in Bereaved Parents: A Pilot Study | 10.3389/fpsyt.2022.872314 |  | Not at all relevant |  |  |  |
| Psychological Stress Identification and Evaluation Method Based on Mobile Human-Computer Interaction Equipment | 10.1155/2022/6039789 |  | Not at all relevant |  |  |  |
| Understanding Patient Experiences, Opinions, and Actions Taken After Viewing Their Own Radiology Images Online: Web-Based Survey | 10.2196/29496 |  | Not at all relevant |  |  |  |
| Evaluation and Correlation Analysis of Mental and Psychological Factors and Premature Ejaculation in Patients with Benign Prostatic Hyperplasia in Mobile Medical System | 10.1155/2022/8260640 |  | Not at all relevant |  |  |  |
| Effects of mobile-based mindfulness meditation for mental health of nurses: a protocol for systematic review and meta-analysis | 10.1136/bmjopen-2021-058686 | Review paper |  |  |  |  |
| Unguided Computer-Assisted Self-Help Interventions Without Human Contact in Patients With Obsessive-Compulsive Disorder: Systematic Review and Meta-analysis | 10.2196/35940 | Review paper |  |  |  |  |
| Digital Health Technologies for Long-term Self-management of Osteoporosis: Systematic Review and Meta-analysis | 10.2196/32557 | Review paper |  |  |  |  |
| Residual Effect of Texting to Promote Medication Adherence for Villagers with Schizophrenia in China: 18-Month Follow-up Survey After the Randomized Controlled Trial Discontinuation | 10.2196/33628 |  | Not at all relevant |  |  |  |
| Antecedents for Older Adults' Intention to Use Smart Health Wearable Devices-Technology Anxiety as a Moderator | 10.3390/bs12040114 |  | Not at all relevant |  |  |  |
| Efficacy and acceptability of next step treatment strategies in adults with treatment-resistant major depressive disorder: protocol for systematic review and network meta-analysis | 10.1136/bmjopen-2021-056777 | Review paper |  |  |  |  |
| Feasibility, Acceptability, and Preliminary Outcomes of a Cognitive Behavioral Therapy-Based Mobile Mental Well-being Program (Noom Mood): Single-Arm Prospective Cohort Study | 10.2196/36794 |  |  |  | Telehealth |  |
| COVID-19 and the Infodemic: An Overview of the Role and Impact of Social Media, the Evolution of Medical Knowledge, and Emerging Problems | 10.3390/healthcare10040732 |  | Not at all relevant |  |  |  |
| App-Based Mindfulness Meditation for People of Color Who Experience Race-Related Stress: Protocol for a Randomized Controlled Trial | 10.2196/35196 |  | Not at all relevant |  |  |  |
| Online Support and Intervention for Child Anxiety (OSI): Development and Usability Testing | 10.2196/29846 |  | Not at all relevant |  |  |  |
| Loneliness and Social Isolation Detection Using Passive Sensing Techniques: Scoping Review | 10.2196/34638 | Review paper |  |  |  |  |
| Assessment of a Mobile Health iPhone App for Semiautomated Self-management of Chronic Recurrent Medical Conditions Using an N-of-1 Trial Framework: Feasibility Pilot Study | 10.2196/34827 |  | Not at all relevant |  |  |  |
| Evaluating the Therapeutic Alliance With a Free-Text CBT Conversational Agent (Wysa): A Mixed-Methods Study | 10.3389/fdgth.2022.847991 |  | Not at all relevant |  |  |  |
| Phase 3b Multicenter, Prospective, Open-label Trial to Evaluate the Effects of a Digital Medicine System on Inpatient Psychiatric Hospitalization Rates for Adults With Schizophrenia | 10.4088/JCP.21m14132 |  | Not at all relevant |  |  |  |
| Anticipating manic and depressive transitions in patients with bipolar disorder using early warning signals | 10.1186/s40345-022-00258-4 |  | Not at all relevant |  |  |  |
| Feasibility of a Machine Learning-Based Smartphone Application in Detecting Depression and Anxiety in a Generally Senior Population | 10.3389/fpsyg.2022.811517 |  | Not at all relevant |  |  |  |
| Does Mobile Internet Use Affect the Depression of Young Chinese Adults? An Instrumental Variable Quantile Analysis | 10.3390/ijerph19084473 |  | Not at all relevant |  |  |  |
| Boamente: A Natural Language Processing-Based Digital Phenotyping Tool for Smart Monitoring of Suicidal Ideation | 10.3390/healthcare10040698 |  | Not at all relevant |  |  |  |
| Human Support in App-Based Cognitive Behavioral Therapies for Emotional Disorders: Scoping Review | 10.2196/33307 | Review paper |  |  |  |  |
| Empirical Investigation for Predicting Depression from Different Machine Learning Based Voice Recognition Techniques | 10.1155/2022/6395860 |  | Not at all relevant |  |  |  |
| Baicalin Attenuates Continuous Activation of β-Catenin Induced by Lipopolysaccharide (LPS) and Depression Complicated by Infertility in Male Rats | 10.1155/2022/2112359 |  | Not at all relevant |  |  |  |
| User experience and acceptance of patients and healthy adults testing a personalized self-management app for depression: A non-randomized mixed-methods feasibility study | 10.1177/20552076221091353 |  | Not at all relevant |  |  |  |
| Linking Individual-Level Facebook Posts With Psychological and Health Data in an Epidemiological Cohort: Feasibility Study | 10.2196/32423 |  | Not at all relevant |  |  |  |
| Impact of Depression on Anxiety, Well-being, and Suicidality in Mexican Adolescent and Young Adult Students From Mexico City: A Mental Health Screening Using Smartphones | 10.4088/JCP.20m13806 |  | Not at all relevant |  |  |  |
| Association Between Step Count Measured With a Smartphone App (Pain-Note) and Pain Level in Patients With Chronic Pain: Observational Study | 10.2196/23657 |  | Not at all relevant |  |  |  |
| Education Racial and Gender Disparities in COVID-19 Worry, Stress, and Food Insecurities across Undergraduate Biology Students at a Southeastern University | 10.1128/jmbe.00224-21 |  | Not at all relevant |  |  |  |
| Multiomic profiling of the acute stress response in the mouse hippocampus | 10.1038/s41467-022-29367-5 |  | Not at all relevant |  |  |  |
| Addressing the range anxiety of battery electric vehicles with charging en route | 10.1038/s41598-022-08942-2 |  | Not at all relevant |  |  |  |
| Brief Digital Interventions to Support the Psychological Well-being of NHS Staff During the COVID-19 Pandemic: 3-Arm Pilot Randomized Controlled Trial | 10.2196/34002 |  | Not at all relevant |  |  |  |
| COVID Student Study: A Year in the Life of College Students during the COVID-19 Pandemic Through the Lens of Mobile Phone Sensing | 10.1145/3491102.3502043 |  | Not at all relevant |  |  |  |
| SIBAT-A Computerized Assessment Tool for Suicide Ideation and Behavior: Development and Psychometric Properties |  |  | Not at all relevant |  |  |  |
| Wellness of hospitalists and hospital medicine advanced practice providers during the COVID-19 pandemic, 2020-2021 | 10.1002/jhm.12812 |  | Not at all relevant |  |  |  |
| Prevalence of phubbing by student pharmacists at two colleges of pharmacy | 10.1016/j.cptl.2022.03.008 |  | Not at all relevant |  |  |  |
| [Internet- and Mobile-based interventions aimed at suicide risks: a review of the literature.] | 10.1701/3792.37766 | Review paper |  |  |  |  |
| Two defence systems eliminate plasmids from seventh pandemic Vibrio cholerae | 10.1038/s41586-022-04546-y |  | Not at all relevant |  |  |  |
| Lessons learned from designing an asynchronous remote community approach for behavioral activation intervention for teens | 10.1016/j.brat.2022.104065 |  | Not at all relevant |  |  |  |
| Qualitative analysis of participant experiences during an ecological momentary assessment study of nonsuicidal self-injury among veterans | 10.1016/j.psychres.2022.114437 |  | Not at all relevant |  |  |  |
| The relationship between text message sentiment and self-reported depression | 10.1016/j.jad.2021.12.048 |  | Not at all relevant |  |  |  |
| Affective states and nonsuicidal self-injury (NSSI): Results from an ecological momentary assessment study of veterans with NSSI disorder | 10.1111/sltb.12818 |  | Not at all relevant |  |  |  |
| Digital stress management in cancer: Testing StressProffen in a 12-month randomized controlled trial | 10.1002/cncr.34046 |  | Not at all relevant |  |  |  |
| Deficiency of MTH1 and/or OGG1 increases the accumulation of 8-oxoguanine in the brain of the App(NL-G-F/NL-G-F) knock-in mouse model of Alzheimer's disease, accompanied by accelerated microgliosis and reduced anxiety-like behavior | 10.1016/j.neures.2021.11.009 |  | Not at all relevant |  |  |  |
| The efficacy of web or mobile-based interventions to alleviate emotional symptoms in people with advanced cancer: a systematic review and meta-analysis | 10.1007/s00520-021-06496-z | Review paper |  |  |  |  |
| Pretransplant Patient Education in Solid-organ Transplant: A Narrative Review | 10.1097/TP.0000000000003893 | Review paper |  |  |  |  |
| The impact of social unrest and pandemic on mental health of young people in Hong Kong: The transdiagnostic role of event-based rumination | 10.1177/00048674211025710 |  | Not at all relevant |  |  |  |
| Assessing College Students' Perceptions of and Intentions to Use a Mobile App for Mental Health | 10.1089/tmj.2021.0106 |  | Not at all relevant |  |  |  |
| Adherence to hydroxyurea, health-related quality of life domains and attitudes towards a smartphone app among Irish adolescents and young adults with sickle cell disease | 10.1007/s11845-021-02588-1 |  | Not at all relevant |  |  |  |
| CALMA, a Mobile Health Application, as an Accessory to Therapy for Reduction of Suicidal and Non-Suicidal Self-Injured Behaviors: A Pilot Cluster Randomized Controlled Trial | 10.1080/13811118.2020.1834476 |  | Not at all relevant |  |  |  |
| Utilizing ACT daily as a self-guided app for clients waiting for services at a college counseling center: A pilot study | 10.1080/07448481.2020.1763366 |  | Not at all relevant |  |  |  |
| Trajectory Analysis of Suicidal Ideation in Spanish College Students Using Ecological Momentary Assessment | 10.3389/fpsyt.2022.853464 |  | Not at all relevant |  |  |  |
| Assessment of Mental Workload by Visual Motor Activity among Control Group and Patient Suffering from Depressive Disorder | 10.1155/2022/8555489 |  | Not at all relevant |  |  |  |
| Effectiveness of digital mental health interventions for university students: an umbrella review | 10.7717/peerj.13111 | Review paper |  |  |  |  |
| Delivery of a Mental Health Intervention for Chronic Pain Through an Artificial Intelligence-Enabled App (Wysa): Protocol for a Prospective Pilot Study | 10.2196/36910 |  | Not at all relevant |  |  |  |
| A Web-Based App for Emotional Management During the COVID-19 Pandemic: Platform Development and Retrospective Analysis of its Use Throughout Two Waves of the Outbreak in Spain | 10.2196/27402 |  | Not at all relevant |  |  |  |
| Efficacy, Benefits, and Harms of a Self-management App in a Swedish Trauma-Exposed Community Sample (PTSD Coach): Randomized Controlled Trial | 10.2196/31419 |  | Not at all relevant |  |  |  |
| Toward a Digital Health Intervention for Vestibular Rehabilitation: Usability and Subjective Outcomes of a Novel Platform | 10.3389/fneur.2022.836796 |  | Not at all relevant |  |  |  |
| A Smartphone-Based Intervention as an Adjunct to Standard-of-Care Treatment for Schizophrenia: Randomized Controlled Trial | 10.2196/29154 |  | Not at all relevant |  |  |  |
| Sarcopenia and its association with objectively measured life-space mobility and moderate-to-vigorous physical activity in the oldest-old amid the COVID-19 pandemic when a physical distancing policy is in force | 10.1186/s12877-022-02861-7 |  | Not at all relevant |  |  |  |
| Feasibility and Acceptability of Ecological Momentary Assessment With Young Adults Who Are Currently or Were Formerly Homeless: Mixed Methods Study | 10.2196/33387 |  | Not at all relevant |  |  |  |
| Attempted suicide of two confirmed SARS-CoV-2 infected patients in an isolation facility and recommendations to prevent COVID-19 suicides: a case report | 10.11604/pamj.2022.41.245.29660 |  | Not at all relevant |  |  |  |
| Role of Telemedicine in Inflammatory Bowel Disease: Systematic Review and Meta-analysis of Randomized Controlled Trials | 10.2196/28978 | Review paper |  |  |  |  |
| Review of Mobile Apps for Women With Anxiety in Pregnancy: Maternity Care Professionals' Guide to Locating and Assessing Anxiety Apps | 10.2196/31831 | Review paper |  |  |  |  |
| Process and Outcome Evaluations of Smartphone Apps for Bipolar Disorder: Scoping Review | 10.2196/29114 | Review paper |  |  |  |  |
| Optimizing Existing Mental Health Screening Methods in a Dementia Screening and Risk Factor App: Observational Machine Learning Study | 10.2196/31209 |  | Not at all relevant |  |  |  |
| Smartphone applications for informal caregivers of chronically ill patients: a scoping review | 10.1038/s41746-022-00567-z | Review paper |  |  |  |  |
| Digital phenotyping in depression diagnostics: Integrating psychiatric and engineering perspectives | 10.5498/wjp.v12.i3.393 |  | Not at all relevant |  |  |  |
| Design and Preliminary Realization of a Screening and Early Warning Health Management System for Populations at High Risk for Depression | 10.3390/ijerph19063599 |  | Not at all relevant |  |  |  |
| A Data-Driven Clustering Method for Discovering Profiles in the Dynamics of Major Depressive Disorder Using a Smartphone-Based Ecological Momentary Assessment of Mood | 10.3389/fpsyt.2022.755809 |  | Not at all relevant |  |  |  |
| Predicting Acceptance of e-Mental Health Interventions in Patients With Obesity by Using an Extended Unified Theory of Acceptance Model: Cross-sectional Study | 10.2196/31229 |  | Not at all relevant |  |  |  |
| Usage Intensity of a Relapse Prevention Program and Its Relation to Symptom Severity in Remitted Patients With Anxiety and Depression: Pre-Post Study | 10.2196/25441 |  | Not at all relevant |  |  |  |
| Remote data collection speech analysis and prediction of the identification of Alzheimer's disease biomarkers in people at risk for Alzheimer's disease dementia: the Speech on the Phone Assessment (SPeAk) prospective observational study protocol | 10.1136/bmjopen-2021-052250 |  | Not at all relevant |  |  |  |
| An Educational and Exercise Mobile Phone-Based Intervention to Elicit Electrophysiological Changes and to Improve Psychological Functioning in Adults With Nonspecific Chronic Low Back Pain (BackFit App): Nonrandomized Clinical Trial | 10.2196/29171 |  | Not at all relevant |  |  |  |
| Effects of a Person-Centered eHealth Intervention for Patients on Sick Leave Due to Common Mental Disorders (PROMISE Study): Open Randomized Controlled Trial | 10.2196/30966 |  | Not at all relevant |  |  |  |
| Risk factors associated with postpartum depressive symptoms: A multinational study | 10.1016/j.jad.2021.12.121 |  | Not at all relevant |  |  |  |
| Development of the Validated Stability-Indicating Method for the Determination of Vortioxetine in Bulk and Pharmaceutical Formulation by HPLC-DAD, Stress Degradation Kinetics Studies and Detection of Degradation Products by LC-ESI-QTOF-MS | 10.3390/molecules27061883 |  | Not at all relevant |  |  |  |
| Use of Mobile and Wearable Artificial Intelligence in Child and Adolescent Psychiatry: Scoping Review | 10.2196/33560 | Review paper |  |  |  |  |
| Clinical outcomes one year after a digital musculoskeletal (MSK) program: an observational, longitudinal study with nonparticipant comparison group | 10.1186/s12891-022-05188-x |  | Not at all relevant |  |  |  |
| Longitudinal Relationships Between Depressive Symptom Severity and Phone-Measured Mobility: Dynamic Structural Equation Modeling Study | 10.2196/34898 |  | Not at all relevant |  |  |  |
| Implementing Machine Learning Models for Suicide Risk Prediction in Clinical Practice: Focus Group Study With Hospital Providers | 10.2196/30946 |  | Not at all relevant |  |  |  |
| Toward a Better Understanding of Quality Social Connections. Comment on "Quality Social Connection as an Active Ingredient in Digital Interventions for Young People With Depression and Anxiety: Systematic Scoping Review and Meta-analysis" | 10.2196/36739 | Review paper |  |  |  |  |
| Authors' Reply to: Toward a Better Understanding of Quality Social Connections. Comment on "Quality Social Connection as an Active Ingredient in Digital Interventions for Young People With Depression and Anxiety: Systematic Scoping Review and Meta-analysis" | 10.2196/37440 | Review paper |  |  |  |  |
| Thematic Analysis on User Reviews for Depression and Anxiety Chatbot Apps: Machine Learning Approach | 10.2196/27654 | Review paper |  |  |  |  |
| CoGNIT Automated Tablet Computer Cognitive Testing in Patients With Mild Cognitive Impairment: Feasibility Study | 10.2196/23589 |  | Not at all relevant |  |  |  |
| Update on hormone therapy for the management of postmenopausal women | 10.5582/bst.2021.01418 |  | Not at all relevant |  |  |  |
| Higher Dietary Inflammatory Index Scores Are Associated With Stress and Anxiety in Dormitory-Residing Female University Students in the United Arab Emirates | 10.3389/fnut.2022.814409 |  | Not at all relevant |  |  |  |
| Suicide rates and suicidal behaviour in displaced people: A systematic review | 10.1371/journal.pone.0263797 | Review paper |  |  |  |  |
| Education on Depression in Mental Health Apps: Systematic Assessment of Characteristics and Adherence to Evidence-Based Guidelines | 10.2196/28942 | Review paper |  |  |  |  |
| Informing the Future of Integrated Digital and Clinical Mental Health Care: Synthesis of the Outcomes From Project Synergy | 10.2196/33060 |  | Not at all relevant |  |  |  |
| The Development of an mHealth Tool for Children With Long-term Illness to Enable Person-Centered Communication: User-Centered Design Approach | 10.2196/30364 |  | Not at all relevant |  |  |  |
| [The clinical application of mobile internet remote guidance platform for vestibular rehabilitation] | 10.3760/cma.j.cn115330-20210528-00302 |  | Not at all relevant |  |  |  |
| Time for united action on depression: a Lancet-World Psychiatric Association Commission | 10.1016/S0140-6736(21)02141-3 |  | Not at all relevant |  |  |  |
| A Famous Chinese Medicine Formula: Yinhuo Decoction Antagonizes the Damage of Corticosterone to PC12 Cells and Improves Depression by Regulating the SIRT1/PGC-1α Pathway | 10.1155/2022/3714857 |  | Not at all relevant |  |  |  |
| Future anxiety and coping methods of nursing students during COVID-19 pandemic: A cross-sectional study | 10.1097/MD.0000000000028989 |  | Not at all relevant |  |  |  |
| Does m-health-based exercise (guidance plus education) improve efficacy in patients with chronic low-back pain? A preliminary report on the intervention's significance | 10.1186/s13063-022-06116-z |  | Not at all relevant |  |  |  |
| A Smartphone Serious Game for Adolescents (Grow It! App): Development, Feasibility, and Acceptance Study | 10.2196/29832 |  | Not at all relevant |  |  |  |
| Feasibility and Preliminary Efficacy of Web-Based and Mobile Interventions for Common Mental Health Problems in Working Adults: Multi-Arm Randomized Pilot Trial | 10.2196/34032 |  | Not at all relevant |  |  |  |
| State of anxiety may be associated with exocrine pancreatic insufficiency in functional dyspepsia patients with pancreatic enzyme abnormalities | 10.3164/jcbn.21-67 |  | Not at all relevant |  |  |  |
| Relation of Anxiety, Depression, and Behavioral Problems With Time Allocated to Television, Computer, and Smartphone in Children Receiving Renal Replacement Therapy | 10.6002/ect.MESOT2021.P42 |  | Not at all relevant |  |  |  |
| Erratum to "Supportive effect of body contact care with ylang ylang aromatherapy and mobile intervention team for suicide prevention: A pilot study" | 10.1177/03000605221092811 |  | Not at all relevant |  |  |  |
| Effects of an integrated mindfulness intervention for veterans with diabetes distress: a randomized controlled trial | 10.1136/bmjdrc-2021-002631 |  | Not at all relevant |  |  |  |
| Adverse experiences of social adaptation in children with leukaemia: a qualitative study from China | 10.1136/bmjopen-2021-051953 |  | Not at all relevant |  |  |  |
| An Ecological Momentary Intervention Study of Emotional Responses to Smartphone-Prompted CBT Skills Practice and the Relationship to Clinical Outcomes | 10.1016/j.beth.2021.09.001 |  | Not at all relevant |  |  |  |
| Mental health monitoring apps for depression and anxiety in children and young people: A scoping review and critical ecological analysis | 10.1016/j.socscimed.2022.114802 | Review paper |  |  |  |  |
| In a life full of risks, COVID-19 makes little difference. Responses to COVID-19 among mobile migrants in gold mining areas in Suriname and French Guiana | 10.1016/j.socscimed.2022.114747 |  | Not at all relevant |  |  |  |
| Family interventions for relapse prevention in schizophrenia: a systematic review and network meta-analysis | 10.1016/S2215-0366(21)00437-5 | Review paper |  |  |  |  |
| Integration of technology to clinical teaching:The impact of mobile and web-based software automation designed for midwifery students on motivation, time management and anxiety levels | 10.1016/j.midw.2021.103248 |  | Not at all relevant |  |  |  |
| Using AI chatbots to provide self-help depression interventions for university students: A randomized trial of effectiveness | 10.1016/j.invent.2022.100495 |  | Not at all relevant |  |  |  |
| Uptake and effectiveness of a self-guided mobile app platform for college student mental health | 10.1016/j.invent.2021.100493 |  | Not at all relevant |  |  |  |
| Food insecurity is associated with eating disorders independent of depression and anxiety: Findings from the 2020-2021 Healthy Minds Study | 10.1002/eat.23668 |  | Not at all relevant |  |  |  |
| Enhanced PAtient Clinical Streamlining (EPACS): Quality Initiative to Improve Healthcare for New Surgical Outpatient Visits | 10.1245/s10434-021-11126-3 |  | Not at all relevant |  |  |  |
| Thymoma type B2 progression, due to fear of contamination, in association with hydrocephalus: A case report of avoidant behavior during COVID-19 pandemic | 10.1016/j.radcr.2021.12.028 |  | Not at all relevant |  |  |  |
| Current directions in digital interventions for mood and anxiety disorders | 10.1097/YCO.0000000000000772 |  | Not at all relevant |  |  |  |
| Therapeutic alliance in family therapy and clinical outcomes among adolescents at risk for mood disorders | 10.1016/j.jad.2021.12.088 |  | Not at all relevant |  |  |  |
| Which Variant of Anxiety Is Associated with Smartphone Expertise in Community Dwelling Older Adults? | 10.1080/07317115.2021.2013382 |  | Not at all relevant |  |  |  |
| Discriminating between patients with unipolar disorder, bipolar disorder, and healthy control individuals based on voice features collected from naturalistic smartphone calls | 10.1111/acps.13391 |  | Not at all relevant |  |  |  |
| A gamified mobile health intervention for children in day surgery care: Protocol for a randomized controlled trial | 10.1002/nop2.1143 |  | Not at all relevant |  |  |  |
| Personal Characteristics Associated with Ecological Momentary Assessment Compliance in Adult Cochlear Implant Candidates and Users | 10.1055/a-1674-0060 |  | Not at all relevant |  |  |  |
| Factors Related to Smartphone Overdependence in Mothers of Preschoolers: A Systematic Review and Meta-Analysis | 10.3928/02793695-20210915-01 | Review paper |  |  |  |  |
| Effectiveness of mobile health-based self-management interventions in breast cancer patients: a meta-analysis | 10.1007/s00520-021-06568-0 |  | Not at all relevant |  |  |  |
| A mindfulness-based mobile health (mHealth) intervention among psychologically distressed university students in quarantine during the COVID-19 pandemic: A randomized controlled trial | 10.1037/cou0000568 |  |  |  |  |  |
| Lockdown impact on lifestyle and its association with oral parafunctional habits and bruxism in a Spanish adolescent population | 10.1111/ipd.12843 |  | Not at all relevant |  |  |  |
| A comparative study of two Mobile Mental Health Units in different catchment rural areas in Greece | 10.1177/0020764020985896 |  | Not at all relevant |  |  |  |
| When expanding training from working memory to emotional working memory: not only improving explicit emotion regulation but also implicit negative control for anxious individuals | 10.1017/S0033291720002275 |  | Not at all relevant |  |  |  |
| Blended Therapies and Mobile Phones for Improving the Health of Female Victims of Gender Violence | 10.3390/healthcare10030445 |  | Not at all relevant |  |  |  |
| Associations of Sustainable Development Goals Accelerators With Adolescents' Well-Being According to Head-of-Household's Disability Status-A Cross-Sectional Study From Zambia | 10.3389/ijph.2022.1604341 |  | Not at all relevant |  |  |  |
| Text Messaging Intervention for Mental Wellness in American Indian and Alaska Native Teens and Young Adults (BRAVE Study): Analysis of User Engagement Patterns | 10.2196/32138 |  | Not at all relevant |  |  |  |
| Telework and Mental Health during COVID-19 | 10.3390/ijerph19052602 |  | Not at all relevant |  |  |  |
| #Brachytherapy: Physicians As Influencers on Instagram | 10.7759/cureus.22524 |  | Not at all relevant |  |  |  |
| Lessons Learned from a Distributed RF-EMF Sensor Network | 10.3390/s22051715 |  | Not at all relevant |  |  |  |
| eLoriCorps Immersive Body Rating Scale and eLoriCorps Mobile Versions: Validation to Assess Body Image Disturbances from Allocentric and Egocentric Perspectives in a Nonclinical Sample of Adolescents | 10.3390/jcm11051156 |  | Not at all relevant |  |  |  |
| The Impact of COVID-19 Confinement on Cognition and Mental Health and Technology Use Among Socially Vulnerable Older People: Retrospective Cohort Study | 10.2196/30598 |  | Not at all relevant |  |  |  |
| Remote Assessment of Disease and Relapse in Major Depressive Disorder (RADAR-MDD): recruitment, retention, and data availability in a longitudinal remote measurement study | 10.1186/s12888-022-03753-1 |  | Not at all relevant |  |  |  |
| eHealth Interventions for Treatment and Prevention of Depression, Anxiety, and Insomnia During Pregnancy: Systematic Review and Meta-analysis | 10.2196/31116 | Review paper |  |  |  |  |
| The Investigation of Adoption of Voice-User Interface (VUI) in Smart Home Systems among Chinese Older Adults | 10.3390/s22041614 |  | Not at all relevant |  |  |  |
| Positive Coping as a Mediator of Mobile Health Intervention Effects on Quality of Life Among People Living With HIV: Secondary Analysis of the Randomized Controlled Trial Run4Love | 10.2196/25948 |  | Not at all relevant |  |  |  |
| Negative Parenting, Adolescents' Emotion Regulation, Self-Efficacy in Emotion Regulation, and Psychological Adjustment | 10.3390/ijerph19042251 |  | Not at all relevant |  |  |  |
| Digital Interventions to Reduce Distress Among Health Care Providers at the Frontline: Protocol for a Feasibility Trial | 10.2196/32240 |  | Not at all relevant |  |  |  |
| Mobile Applications in Mood Disorders and Mental Health: Systematic Search in Apple App Store and Google Play Store and Review of the Literature | 10.3390/ijerph19042186 | Review paper |  |  |  |  |
| Effects of mHealth on the psychosocial health of pregnant women and mothers: a systematic review | 10.1136/bmjopen-2021-056807 | Review paper |  |  |  |  |
| Remotely Delivered Interventions to Support Women With Symptoms of Anxiety in Pregnancy: Mixed Methods Systematic Review and Meta-analysis | 10.2196/28093 | Review paper |  |  |  |  |
| The Efficacy of a Smartphone-Based App on Stress Reduction: Randomized Controlled Trial | 10.2196/28703 |  | Not at all relevant |  |  |  |
| Development and Evaluation of the Usefulness, Usability, and Feasibility of iNNOV Breast Cancer: Mixed Methods Study | 10.2196/33550 |  | Not at all relevant |  |  |  |
| Cannabis use and suicidal ideation among youth: Can we democratize school policies using digital citizen science? | 10.1371/journal.pone.0263533 |  | Not at all relevant |  |  |  |
| The Effectiveness of Serious Games in Alleviating Anxiety: Systematic Review and Meta-analysis | 10.2196/29137 | Review paper |  |  |  |  |
| In Search of State and Trait Emotion Markers in Mobile-Sensed Language: Field Study | 10.2196/31724 |  | Not at all relevant |  |  |  |
| Generational Perspectives on Technology's Role in Mental Health Care: A Survey of Adults With Lived Mental Health Experience | 10.3389/fdgth.2022.840169 |  | Not at all relevant |  |  |  |
| Web-Based Cognitive Testing in Psychiatric Research: Validation and Usability Study | 10.2196/28233 |  | Not at all relevant |  |  |  |
| The Effectiveness of Virtual Reality Exposure-Based Cognitive Behavioral Therapy for Severe Anxiety Disorders, Obsessive-Compulsive Disorder, and Posttraumatic Stress Disorder: Meta-analysis | 10.2196/26736 |  | Not at all relevant |  |  |  |
| Antecedents and outcomes of work-family conflict: A mega-meta path analysis | 10.1371/journal.pone.0263631 |  | Not at all relevant |  |  |  |
| Multimorbidity and co-occurring musculoskeletal pain do not modify the effect of the SELFBACK app on low back pain-related disability | 10.1186/s12916-022-02237-z |  | Not at all relevant |  |  |  |
| Smart clothes-assisted home-nursing care program for family caregivers of older persons with dementia and hip fracture: a mixed-methods study | 10.1186/s12877-022-02789-y |  | Not at all relevant |  |  |  |
| Characteristics of Mobile Health Platforms for Depression and Anxiety: Content Analysis Through a Systematic Review of the Literature and Systematic Search of Two App Stores | 10.2196/27388 | Review paper |  |  |  |  |
| The Quality of Health Apps and Their Potential to Promote Behavior Change in Patients With a Chronic Condition or Multimorbidity: Systematic Search in App Store and Google Play | 10.2196/33168 |  | Not at all relevant |  |  |  |
| Use of technology for self-care in surgical wound infection surveillance: integrative review | 10.1590/0034-7167-2021-0208 | Review paper |  |  |  |  |
| Effectiveness of eHealth and mHealth Interventions Supporting Children and Young People Living With Juvenile Idiopathic Arthritis: Systematic Review and Meta-analysis | 10.2196/30457 | Review paper |  |  |  |  |
| Effectiveness of the iParent app for postnatal depression: a randomised controlled trial (abridged secondary publication) |  |  | Not at all relevant |  |  |  |
| Estimating longitudinal depressive symptoms from smartphone data in a transdiagnostic cohort | 10.1002/brb3.2077 |  | Not at all relevant |  |  |  |
| Digital biomarkers of anxiety disorder symptom changes: Personalized deep learning models using smartphone sensors accurately predict anxiety symptoms from ecological momentary assessments | 10.1016/j.brat.2021.104013 |  | Not at all relevant |  |  |  |
| Prevalence of Nomophobia and an Analysis of Its Contributing Factors in the Undergraduate Students of Pakistan | 10.1089/cyber.2021.0148 |  | Not at all relevant |  |  |  |
| Sharing positive events: Ecological momentary assessment of emotion regulation via social capitalization in schizotypy | 10.1016/j.psychres.2021.114377 |  | Not at all relevant |  |  |  |
| Suction feeding biomechanics of Polypterus bichir: investigating linkage mechanisms and the contributions of cranial kinesis to oral cavity volume change | 10.1242/jeb.243283 |  | Not at all relevant |  |  |  |
| Electrolyte-Gated Vertical Synapse Array based on Van Der Waals Heterostructure for Parallel Computing | 10.1002/advs.202103808 |  | Not at all relevant |  |  |  |
| Feasibility of a theory-informed mobile app for changing physical activity in youth with multiple sclerosis | 10.1016/j.msard.2021.103467 |  | Not at all relevant |  |  |  |
| Effect of digital cognitive behavioral therapy on psychological symptoms among perinatal women in high income-countries: A systematic review and meta-regression | 10.1016/j.jpsychires.2021.11.012 | Review paper |  |  |  |  |
| Longitudinal association between smartphone ownership and depression among schoolchildren under COVID-19 pandemic | 10.1007/s00127-021-02196-5 |  | Not at all relevant |  |  |  |
| Optimizing Pediatric Induction Experiences Using Human-centered Design | 10.1016/j.jopan.2021.03.001 |  | Not at all relevant |  |  |  |
| Posttraumatic growth in Chinese nurses and general public during the COVID-19 outbreak | 10.1080/13548506.2021.1897148 |  | Not at all relevant |  |  |  |
| Prehabilitation with wearables versus standard of care before major abdominal cancer surgery: a randomised controlled pilot study (trial registration: NCT04047524) | 10.1007/s00464-021-08365-6 |  | Not at all relevant |  |  |  |
| Factors associated with mental health outcomes among health care workers in the Fangcang shelter hospital in China | 10.1177/0020764020975805 |  | Not at all relevant |  |  |  |
| Preventing depression using a smartphone app: a randomized controlled trial | 10.1017/S0033291720002081 |  | Not at all relevant |  |  |  |
| The effectiveness of mHealth interventions on postpartum depression: A systematic review and meta-analysis | 10.1177/1357633X20917816 | Review paper |  |  |  |  |
| Diagnostic Performance of an App-Based Symptom Checker in Mental Disorders: Comparative Study in Psychotherapy Outpatients | 10.2196/32832 |  | Not at all relevant |  |  |  |
| Feasibility and potential efficacy of a guided internet- and mobile-based CBT for adolescents and young adults with chronic medical conditions and comorbid depression or anxiety symptoms (youthCOACH(CD)): a randomized controlled pilot trial | 10.1186/s12887-022-03134-3 |  | Not at all relevant |  |  |  |
| Problematic Internet Use Before and During the COVID-19 Pandemic in Youth in Outpatient Mental Health Treatment: App-Based Ecological Momentary Assessment Study | 10.2196/33114 |  | Not at all relevant |  |  |  |
| The Association Between Home Stay and Symptom Severity in Major Depressive Disorder: Preliminary Findings From a Multicenter Observational Study Using Geolocation Data From Smartphones | 10.2196/28095 |  | Not at all relevant |  |  |  |
| Diagnosis of Depressive Disorder Model on Facial Expression Based on Fast R-CNN | 10.3390/diagnostics12020317 |  | Not at all relevant |  |  |  |
| Social+Me: a persuasive application to increase communication between students and their support networks in Southern Chile | 10.7717/peerj-cs.848 |  | Not at all relevant |  |  |  |
| Identifying and Monitoring the Daily Routine of Seniors Living at Home | 10.3390/s22030992 |  | Not at all relevant |  |  |  |
| Heart Rate Information-Based Machine Learning Prediction of Emotions Among Pregnant Women | 10.3389/fpsyt.2021.799029 |  | Not at all relevant |  |  |  |
| Agomelatine Prevents Amyloid Plaque Deposition, Tau Phosphorylation, and Neuroinflammation in APP/PS1 Mice | 10.3389/fnagi.2021.766410 |  | Not at all relevant |  |  |  |
| Impact of Smartphone App-Based Psychological Interventions for Reducing Depressive Symptoms in People With Depression: Systematic Literature Review and Meta-analysis of Randomized Controlled Trials | 10.2196/29621 | Review paper |  |  |  |  |
| The Influence of Personality and Demographic Characteristics on Aggressive Driving Behaviors in Eastern Chinese Drivers | 10.2147/PRBM.S323431 |  | Not at all relevant |  |  |  |
| Challenges of Telemonitoring Programs for Complex Chronic Conditions: Randomized Controlled Trial With an Embedded Qualitative Study | 10.2196/31754 |  | Not at all relevant |  |  |  |
| Psychopathological Symptoms and Personality Traits as Predictors of Problematic Smartphone Use in Different Age Groups | 10.3390/bs12020020 |  | Not at all relevant |  |  |  |
| Effects of Mindfulness Exercise Guided by a Smartphone App on Negative Emotions and Stress in Non-Clinical Populations: A Systematic Review and Meta-Analysis | 10.3389/fpubh.2021.773296 | Review paper |  |  |  |  |
| Analysis of E-mental health research: mapping the relationship between information technology and mental healthcare | 10.1186/s12888-022-03713-9 |  | Not at all relevant |  |  |  |
| Patterns of social relationships among community-dwelling older adults in Japan: latent class analysis | 10.1186/s12877-022-02748-7 |  | Not at all relevant |  |  |  |
| Effectiveness, User Engagement and Experience, and Safety of a Mobile App (Lumi Nova) Delivering Exposure-Based Cognitive Behavioral Therapy Strategies to Manage Anxiety in Children via Immersive Gaming Technology: Preliminary Evaluation Study | 10.2196/29008 |  | Not at all relevant |  |  |  |
| A Virtual Coach (Motibot) for Supporting Healthy Coping Strategies Among Adults With Diabetes: Proof-of-Concept Study | 10.2196/32211 |  | Not at all relevant |  |  |  |
| An Acceptance and Commitment Therapy Prototype Mobile Program for Individuals With a Visible Difference: Mixed Methods Feasibility Study | 10.2196/33449 |  | Not at all relevant |  |  |  |
| Mobile Phone-Based Intervention Among Adolescents Living With Perinatally Acquired HIV Transitioning from Pediatric to Adult Care: Protocol for the Interactive Transition Support for Adolescents Living With HIV using Social Media (InTSHA) Study | 10.2196/35455 |  | Not at all relevant |  |  |  |
| Mobility enhancement among older adults 75 + in rural areas: Study protocol of the MOBILE randomized controlled trial | 10.1186/s12877-021-02739-0 |  | Not at all relevant |  |  |  |
| [Smartphone apps for self-diagnosis of skin cancer] |  |  | Not at all relevant |  |  |  |
| A Digital Intervention for Respiratory Tract Infections (Internet Dr): Process Evaluation to Understand How to Support Self-care for Minor Ailments | 10.2196/24239 |  | Not at all relevant |  |  |  |
| Efficacy, Effectiveness, and Quality of Resilience-Building Mobile Health Apps for Military, Veteran, and Public Safety Personnel Populations: Scoping Literature Review and App Evaluation | 10.2196/26453 | Review paper |  |  |  |  |
| Trends and Factors Associated With Risk Perception, Anxiety, and Behavior From the Early Outbreak Period to the Controlled Period of COVID-19 Epidemic: Four Cross-Sectional Online Surveys in China in 2020 | 10.3389/fpubh.2021.768867 |  | Not at all relevant |  |  |  |
| Acceptability and efficacy of the Zemedy app versus a relaxation training and meditation app for IBS: protocol for a randomised controlled trial | 10.1136/bmjopen-2021-055014 |  | Not at all relevant |  |  |  |
| mHealth Solutions for Perinatal Mental Health: Scoping Review and Appraisal Following the mHealth Index and Navigation Database Framework | 10.2196/30724 | Review paper |  |  |  |  |
| Attention control moderates the relationship between social media use and psychological distress | 10.1016/j.jad.2021.10.071 |  | Not at all relevant |  |  |  |
| Prefrontal transcranial magnetic stimulation for depression in US military veterans - A naturalistic cohort study in the veterans health administration | 10.1016/j.jad.2021.10.025 |  | Not at all relevant |  |  |  |
| Development of a Web-App for the Ecological Momentary Assessment of Dietary Habits among College Students: The HEALTHY-UNICT Project | 10.3390/nu14020330 |  | Not at all relevant |  |  |  |
| A Counseling Mobile App to Reduce the Psychosocial Impact of Human Papillomavirus Testing: Formative Research Using a User-Centered Design Approach in a Low-Middle-Income Setting in Argentina | 10.2196/32610 |  | Not at all relevant |  |  |  |
| Toward Designs of Workplace Stress Management Mobile Apps for Frontline Health Workers During the COVID-19 Pandemic and Beyond: Mixed Methods Qualitative Study | 10.2196/30640 |  | Not at all relevant |  |  |  |
| The impact of depression, anxiety and comorbidity on occupational outcomes | 10.1093/occmed/kqab142 |  | Not at all relevant |  |  |  |
| Information-Seeking Strategies of People with Multiple Sclerosis in Spain: The INFOSEEK-MS Study | 10.2147/PPA.S344690 |  | Not at all relevant |  |  |  |
| Effectiveness of Live Health Professional-Led Group eHealth Interventions for Adult Mental Health: Systematic Review of Randomized Controlled Trials | 10.2196/27939 | Review paper |  |  |  |  |
| Extracellular Vesicle Delivery of Neferine for the Attenuation of Neurodegenerative Disease Proteins and Motor Deficit in an Alzheimer's Disease Mouse Model | 10.3390/ph15010083 |  | Not at all relevant |  |  |  |
| Developing a Web-Based App to Assess Mental Health Difficulties in Secondary School Pupils: Qualitative User-Centered Design Study | 10.2196/30565 |  | Not at all relevant |  |  |  |
| Modelling the geographical spread of HIV among MSM in Guangdong, China: a metapopulation model considering the impact of pre-exposure prophylaxis | 10.1098/rsta.2021.0126 |  | Not at all relevant |  |  |  |
| Implementation of a Mobile DBT App and Its Impact on Suicidality in Transitional Age Youth with Borderline Personality Disorder: A Qualitative Study | 10.3390/ijerph19020701 |  | Not at all relevant |  |  |  |
| A Feasibility Study of the WHO Digital Mental Health Intervention Step-by-Step to Address Depression Among Chinese Young Adults | 10.3389/fpsyt.2021.812667 |  | Not at all relevant |  |  |  |
| Evaluation of animal model congruence to human depression based on large-scale gene expression patterns of the CNS | 10.1038/s41598-021-04020-1 |  | Not at all relevant |  |  |  |
| Exploring Barriers to and Enablers of the Adoption of Information and Communication Technology for the Care of Older Adults With Chronic Diseases: Scoping Review | 10.2196/25251 | Review paper |  |  |  |  |
| Improved Prediction Model of Protein Lysine Crotonylation Sites Using Bidirectional Recurrent Neural Networks | 10.1021/acs.jproteome.1c00848 |  | Not at all relevant |  |  |  |
| Effectiveness of Digital Counseling Environments on Anxiety, Depression, and Adherence to Treatment Among Patients Who Are Chronically Ill: Systematic Review | 10.2196/30077 | Review paper |  |  |  |  |
| The microRNA-455 Null Mouse Has Memory Deficit and Increased Anxiety, Targeting Key Genes Involved in Alzheimer's Disease | 10.3390/ijms23010554 |  | Not at all relevant |  |  |  |
| Dose-Response Effects of Patient Engagement on Health Outcomes in an mHealth Intervention: Secondary Analysis of a Randomized Controlled Trial | 10.2196/25586 |  | Not at all relevant |  |  |  |
| Implications and Preventions of Cyberbullying and Social Exclusion in Social Media: Systematic Review | 10.2196/30286 | Review paper |  |  |  |  |
| Informing about the invisible: communicating en route air pollution and noise exposure to cyclists and pedestrians using focus groups | 10.1186/s12544-022-00571-0 |  | Not at all relevant |  |  |  |
| Feasibility and acceptability of the mobile application for the prevention of suicide (MAPS) | 10.1080/08995605.2021.1962187 |  | Not at all relevant |  |  |  |
| Sexual Experiences and Attachment Styles in Online and Offline Dating Contexts | 10.1080/19317611.2022.2110349 |  | Not at all relevant |  |  |  |
| Identification of maternal depression risk from natural language collected in a mobile health app | 10.1016/j.procs.2022.09.092 |  | Not at all relevant |  |  |  |
| Prevalence of depression, anxiety, stress and its relationship with knowledge about COVID-19 in medical and laboratory medicine students of Umm-Al-Qura University: a cross-sectional survey | 10.1186/s41983-022-00590-7 |  | Not at all relevant |  |  |  |
| Effects of Metformin on Modulating the Expression of Brain-related Genes of APP/PS1 Transgenic Mice based on Single Cell Sequencing | 10.2174/1567205020666221201143323 |  | Not at all relevant |  |  |  |
| Appreciating the Good Things in Life During the Covid-19 Pandemic: A Randomized Controlled Trial and Evaluation of a Gratitude App | 10.1007/s10902-022-00586-3 |  | Not at all relevant |  |  |  |
| The Impact of a Mindfulness App on Postnatal Distress | 10.1007/s12671-022-01992-7 |  | Not at all relevant |  |  |  |
| Use of Mobile Apps and Online Programs of Mindfulness and Self-Compassion Training in Workers: A Scoping Review | 10.1007/s41347-022-00267-1 | Review paper |  |  |  |  |
| Rediscovering Allergic Rhinitis: The Use of a Novel mHealth Solution to Describe and Monitor Health-Related Quality of Life in Elderly Patients | 10.1159/000525595 |  | Not at all relevant |  |  |  |
| The Association of Embracing with Daily Mood and General Life Satisfaction: An Ecological Momentary Assessment Study | 10.1007/s10919-022-00411-8 |  | Not at all relevant |  |  |  |
| Effect of life course factors on dental fear among adult dental patients attending out-reach clinics in a rural area of Southern India | 10.4103/ijdr.ijdr_113_21 |  | Not at all relevant |  |  |  |
| Evaluation of different pre-treatment behaviour modification techniques in 4-7-year olds: A randomised controlled trial | 10.4103/ijdr.ijdr_373_21 |  | Not at all relevant |  |  |  |
| The use of digital solutions in alleviating the burden of IAPT's waiting times | 10.3233/JRS-227033 |  | Not at all relevant |  |  |  |
| Just-in-Time Adaptive Interventions for Suicide Prevention: Promise, Challenges, and Future Directions | 10.1080/00332747.2022.2092828 |  | Not at all relevant |  |  |  |
| Transcranial Electrical Stimulation for Psychiatric Disorders in Adults: A Primer | 10.1176/appi.focus.20210020 |  | Not at all relevant |  |  |  |
| Effects of App-Based Mobile Interventions for Dementia Family Caregivers: A Systematic Review and Meta-Analysis | 10.1159/000524780 | Review paper |  |  |  |  |
| Efficacy of App-Based Cognitive Behavioral Therapy for Body Dysmorphic Disorder with Coach Support: Initial Randomized Controlled Clinical Trial | 10.1159/000524628 |  | Not at all relevant |  |  |  |
| Current Approaches in Telehealth and Telerehabilitation for Spinal Cord Injury (TeleSCI) | 10.1007/s40141-022-00348-5 |  | Not at all relevant |  |  |  |
| Skinny Genes' Six-week, Online, Clinical Emotional Freedom Techniques Program: Durable Weight Loss and Improved Psychological Symptoms |  |  | Not at all relevant |  |  |  |
| A survey of women's experiences of using period tracker applications: Attitudes, ovulation prediction and how the accuracy of the app in predicting period start dates affects their feelings and behaviours | 10.1177/17455057221095246 |  | Not at all relevant |  |  |  |
| Using Telehealth to Provide Behavioral Healthcare to Young Adults and College Students |  |  | Not at all relevant |  |  |  |
| Co-Treatment with the Herbal Medicine SIP3 and Donepezil Improves Memory and Depression in the Mouse Model of Alzheimer's Disease | 10.2174/1567205019666220413082130 |  | Not at all relevant |  |  |  |
| Predicting subjective well-being in a high-risk sample of Russian mental health app users | 10.1140/epjds/s13688-022-00333-x |  | Not at all relevant |  |  |  |
| Financial Stress Among Latino Adults in California During COVID-19 | 10.1007/s41996-021-00087-0 |  | Not at all relevant |  |  |  |
| Nomophobia and relationships with latent classes of solitude | 10.1521/bumc.2022.86.1.1 |  | Not at all relevant |  |  |  |
| Telehealth Education via WeChat Improves the Quality of Life of Parents of Children with Type-1 Diabetes Mellitus | 10.1055/s-0042-1743239 |  | Not at all relevant |  |  |  |
| C5: A Step Towards Smart World with Enhanced Holistic Wellbeing | 10.1007/s11277-021-09314-1 |  | Not at all relevant |  |  |  |
| Mathematical modeling and impact analysis of the use of COVID Alert SA app | 10.3934/publichealth.2022009 |  | Not at all relevant |  |  |  |
| Effect of 2400 MHz mobile phone radiation exposure on the behavior and hippocampus morphology in Swiss mouse model | 10.1016/j.sjbs.2021.08.063 |  | Not at all relevant |  |  |  |
| Assessing costs of developing a digital program for training community health workers to deliver treatment for depression: A case study in rural India | 10.1016/j.psychres.2021.114299 |  | Not at all relevant |  |  |  |
| Neighborhood disadvantage moderates the effect of a mobile health intervention on adolescent depression | 10.1016/j.healthplace.2021.102728 |  | Not at all relevant |  |  |  |
| Mitochondrial Membrane Potential Influences Amyloid-β Protein Precursor Localization and Amyloid-β Secretion | 10.3233/JAD-215280 |  | Not at all relevant |  |  |  |
| Adiponectin Ameliorates Cognitive Behaviors and in vivo Synaptic Plasticity Impairments in 3xTg-AD Mice | 10.3233/JAD-215063 |  | Not at all relevant |  |  |  |
| A network analysis on self-harming and problematic smartphone use - The role of self-control, internalizing and externalizing problems in a sample of self-harming adolescents | 10.1016/j.comppsych.2021.152285 |  | Not at all relevant |  |  |  |
| mHealth and technology innovations for anxiety and OC spectrum disorders | 10.1111/bjc.12341 |  | Not at all relevant |  |  |  |
| The feasibility of home monitoring of young people with cystic fibrosis: Results from CLIMB-CF | 10.1016/j.jcf.2021.09.018 |  | Not at all relevant |  |  |  |
| Evaluating the effectiveness and quality of mobile applications for perinatal depression and anxiety: A systematic review and meta-analysis | 10.1016/j.jad.2021.09.106 | Review paper |  |  |  |  |
| Smartphone non-users experience disproportionately higher psychological distress than their counterparts: Mediations via psychosocial resources in a large sample of college students in China | 10.1016/j.jad.2021.09.058 |  | Not at all relevant |  |  |  |
| Antenatal depressive symptoms in Kenyan women living with HIV: contributions of recent HIV diagnosis, stigma, and partner violence | 10.1080/09540121.2021.1981216 |  | Not at all relevant |  |  |  |
| A Prospective Study of the Effect of Tinnitus Sound Matching Degree on the Efficacy of Customized Sound Therapy in Patients with Chronic Tinnitus | 10.1159/000517631 |  | Not at all relevant |  |  |  |
| Use of a mobile app to capture supplemental health information during pregnancy: Implications for clinical research | 10.1002/pds.5320 |  | Not at all relevant |  |  |  |
| Effects of a Video-Based mHealth Program for Homebound Older Adults: Study Protocol for a Pilot Randomized Controlled Trial | 10.1159/000516967 |  | Not at all relevant |  |  |  |
| Transactional links between children daily emotions and internalizing symptoms: a six-wave ecological momentary assessment study | 10.1111/jcpp.13432 |  | Not at all relevant |  |  |  |
| Response to: Mindfulness-based mobile app reduces anxiety and increases self-compassion in healthcare students: A randomised controlled trial | 10.1080/0142159X.2021.1918657 |  | Not at all relevant |  |  |  |
| Emotion network density is a potential clinical marker for anxiety and depression: Comparison of ecological momentary assessment and daily diary | 10.1111/bjc.12295 |  | Not at all relevant |  |  |  |
| Sailing in a sea of perplexity: Family caregivers' experience of patients with delirium | 10.1111/nicc.12620 |  | Not at all relevant |  |  |  |
| Allopurinol for fibromyalgia pain in adults: A randomized controlled trial | 10.1111/papr.13019 |  | Not at all relevant |  |  |  |
| Does interpersonal sensitivity and paranoid ideation predict nomophobia: an analysis with a young adult sample | 10.1007/s12144-021-01501-4 |  | Not at all relevant |  |  |  |
| Building an emotion regulation recommender algorithm for socially anxious individuals using contextual bandits | 10.1111/bjc.12282 |  | Not at all relevant |  |  |  |
| The Effect of Mobile Learning on Student Success and Anxiety in Teaching Genital System Anatomy | 10.1002/ase.2059 |  | Not at all relevant |  |  |  |
| Examining the interpersonal theory of suicide in acutely suicidal psychiatric inpatients | 10.1080/07481187.2020.1852337 |  | Not at all relevant |  |  |  |
| Phenomenon of depression and anxiety related to precautions for prevention among population during the outbreak of COVID-19 in Kurdistan Region of Iraq: based on questionnaire survey | 10.1007/s10389-020-01325-9 |  | Not at all relevant |  |  |  |
| Instability of Suicidal Ideation in Patients Hospitalized for Depression: An Exploratory Study Using Smartphone Ecological Momentary Assessment | 10.1080/13811118.2020.1783410 |  | Not at all relevant |  |  |  |
| Pilot evaluation of the stop, breathe & think mindfulness app for student clients on a college counseling center waitlist | 10.1080/07448481.2020.1728281 |  | Not at all relevant |  |  |  |
| Implementation, efficacy and cost effectiveness of the unified protocol in a blended format for the transdiagnostic treatment of emotional disorders: a study protocol for a multicentre, randomised, superiority controlled trial in the Spanish National Health System | 10.1136/bmjopen-2021-054286 |  | Not at all relevant |  |  |  |
| Exploring interpersonal theory of suicide typologies in patients with cancer: A latent profile analysis | 10.1080/07481187.2021.2021567 |  | Not at all relevant |  |  |  |
| A Mobile App With Multimodality Prehabilitation Programs for Patients Awaiting Elective Surgery: Development and Usability Study | 10.2196/32575 |  | Not at all relevant |  |  |  |
| Development of a Supportive Parenting App to Improve Parent and Infant Outcomes in the Perinatal Period: Development Study | 10.2196/27033 |  | Not at all relevant |  |  |  |
| Centering Lived Experience in Developing Digital Interventions for Suicide and Self-injurious Behaviors: User-Centered Design Approach | 10.2196/31367 |  | Not at all relevant |  |  |  |
| [Information tools commonly used during a natural disaster among information and communication technology device users] | 10.11236/jph.21-023 |  | Not at all relevant |  |  |  |
| Re-engineering a mobile-CRISPR/Cas9 system for antimicrobial resistance gene curing and immunization in Escherichia coli | 10.1093/jac/dkab368 |  | Not at all relevant |  |  |  |
| Development and Evaluation of the Canteen Connect Online Health Community: Using a Participatory Design Approach in Meeting the Needs of Young People Impacted by Cancer | 10.3390/cancers14010050 |  | Not at all relevant |  |  |  |
| Evaluation of a two-way SMS messaging strategy to reduce neonatal mortality: rationale, design and methods of the Mobile WACh NEO randomised controlled trial in Kenya | 10.1136/bmjopen-2021-056062 |  | Not at all relevant |  |  |  |
| Exploring the Effects of In-App Components on Engagement With a Symptom-Tracking Platform Among Participants With Major Depressive Disorder (RADAR-Engage): Protocol for a 2-Armed Randomized Controlled Trial | 10.2196/32653 |  | Not at all relevant |  |  |  |
| Mental Health Implications of the COVID-19 Pandemic Among Children and Adolescents: What Do We Know so Far? | 10.2147/PHMT.S315887 |  | Not at all relevant |  |  |  |
| Effectiveness of online mindfulness interventions on medical students' mental health: a systematic review | 10.1186/s12889-021-12341-z | Review paper |  |  |  |  |
| Quality Social Connection as an Active Ingredient in Digital Interventions for Young People With Depression and Anxiety: Systematic Scoping Review and Meta-analysis | 10.2196/26584 | Review paper |  |  |  |  |
| Smartphone Interventions Effect in Pediatric Subjects on the Day of Surgery: A Meta-Analysis | 10.3389/fsurg.2021.759958 |  | Not at all relevant |  |  |  |
| Awareness, Prevention, Detection, and Therapy Applications for Depression and Anxiety in Serious Games for Children and Adolescents: Systematic Review | 10.2196/30482 | Review paper |  |  |  |  |
| User Reviews of Depression App Features: Sentiment Analysis | 10.2196/17062 | Review paper |  |  |  |  |
| Smartphone Psychological Therapy During COVID-19: A Study on the Effectiveness of Five Popular Mental Health Apps for Anxiety and Depression | 10.3389/fpsyg.2021.775775 |  | Not at all relevant |  |  |  |
| Developing an mHealth Application to Coordinate Nurse-Provided Respite Care Services for Families Coping With Palliative-Stage Cancer: Protocol for a User-Centered Design Study | 10.2196/34652 |  | Not at all relevant |  |  |  |
| The Life Goals Self-Management Mobile App for Bipolar Disorder: Consumer Feasibility, Usability, and Acceptability Study | 10.2196/32450 |  | Not at all relevant |  |  |  |
| Correlates of interpersonal emotion regulation problems in Loss of Control eating (LOC) in youth: study protocol of the combined online and App based questionnaire, laboratory and randomized controlled online intervention i-BEAT trial | 10.1186/s40359-021-00690-8 |  | Not at all relevant |  |  |  |
| Psychosocial Factors Associated With Increased Adolescent Non-suicidal Self-Injury During the COVID-19 Pandemic | 10.3389/fpsyt.2021.743526 |  | Not at all relevant |  |  |  |
| Prenatal anxiety and the associated factors among Chinese pregnant women during the COVID-19 pandemic--a smartphone questionnaire survey study | 10.1186/s12888-021-03624-1 |  | Not at all relevant |  |  |  |
| Long-Term Effectiveness of a Decision Support App (Pink Journey) for Women Considering Breast Reconstruction Surgery: Pilot Randomized Controlled Trial | 10.2196/31092 |  | Not at all relevant |  |  |  |
| An eHealth Intervention for Promoting COVID-19 Knowledge and Protective Behaviors and Reducing Pandemic Distress Among Sexual and Gender Minorities: Protocol for a Randomized Controlled Trial (#SafeHandsSafeHearts) | 10.2196/34381 |  | Not at all relevant |  |  |  |
| Changes in Quality of Life and Loneliness Among Middle-Aged and Older Adults Participating in Therapist-Guided Digital Mental Health Intervention | 10.3389/fpubh.2021.746904 |  | Not at all relevant |  |  |  |
| Effectiveness and Feasibility of Internet-Based Interventions for Grief After Bereavement: Systematic Review and Meta-analysis | 10.2196/29661 | Review paper |  |  |  |  |
| Internet and Face-to-face Cognitive Behavioral Therapy for Postnatal Depression Compared With Treatment as Usual: Randomized Controlled Trial of MumMoodBooster | 10.2196/17185 |  | Not at all relevant |  |  |  |
| Mobile app for prolonged grief among bereaved parents: study protocol for a randomised controlled trial | 10.1136/bmjopen-2021-052763 |  | Not at all relevant |  |  |  |
| A Coached Digital Cognitive Behavioral Intervention Reduces Anxiety and Depression in Adults With Functional Gastrointestinal Disorders | 10.14309/ctg.0000000000000436 |  | Not at all relevant |  |  |  |
| The Association of Maternal Emotional Status With Child Over-Use of Electronic Devices During the COVID-19 Pandemic | 10.3389/fped.2021.760996 |  | Not at all relevant |  |  |  |
| Digital Interventions for Generalized Anxiety Disorder (GAD): Systematic Review and Network Meta-Analysis | 10.3389/fpsyt.2021.726222 | Review paper |  |  |  |  |
| Examining the Theoretical Framework of Behavioral Activation for Major Depressive Disorder: Smartphone-Based Ecological Momentary Assessment Study | 10.2196/32007 |  | Not at all relevant |  |  |  |
| Age- and Diagnosis-Based Trends for Unplanned Pediatric Rehospitalizations in the United States | 10.7759/cureus.20181 |  | Not at all relevant |  |  |  |
| Effects of a Novel, Transdiagnostic, Hybrid Ecological Momentary Intervention for Improving Resilience in Youth (EMIcompass): Protocol for an Exploratory Randomized Controlled Trial | 10.2196/27462 |  | Not at all relevant |  |  |  |
| The effect of a smartphone-based perioperative nursing intervention: prayer, education, exercise therapy, hypnosis, and music toward pain, anxiety, and early mobilization on cardiac surgery | 10.4081/jphr.2021.2742 |  | Not at all relevant |  |  |  |
| Clinical Efficacy and Psychological Mechanisms of an App-Based Digital Therapeutic for Generalized Anxiety Disorder: Randomized Controlled Trial | 10.2196/26987 |  | Not at all relevant |  |  |  |
| An Ecological Momentary Intervention for people with social anxiety: A descriptive case study | 10.1080/17538157.2021.1896525 |  | Not at all relevant |  |  |  |
| Mobile Eye Tracking Captures Changes in Attention Over Time During a Naturalistic Threat Paradigm in Behaviorally Inhibited Children | 10.1007/s42761-021-00077-3 |  | Not at all relevant |  |  |  |
| COVID-Related Stress and Work Intentions in a Sample of US Health Care Workers | 10.1016/j.mayocpiqo.2021.08.007 |  | Not at all relevant |  |  |  |
| A self-guided Internet-delivered intervention for adults with ADHD: a protocol for a randomized controlled trial | 10.1016/j.invent.2021.100485 |  | Not at all relevant |  |  |  |
| A Conversational Artificial Intelligence Agent for a Mental Health Care App: Evaluation Study of Its Participatory Design | 10.2196/30053 |  | Not at all relevant |  |  |  |
| Voice analyses using smartphone-based data in patients with bipolar disorder, unaffected relatives and healthy control individuals, and during different affective states | 10.1186/s40345-021-00243-3 |  | Not at all relevant |  |  |  |
| Accessibility of mental health support in China and preferences on web-based services for mood disorders: A qualitative study | 10.1016/j.invent.2021.100475 |  | Not at all relevant |  |  |  |
| [Quality of care in family practice and quality of life from the point of view of older patients with gon- and coxosteoarthritis - results from the MobilE-TRA cohort study] | 10.1007/s15006-021-0455-x |  | Not at all relevant |  |  |  |
| Beach tourists behavior and beach management strategy under the ongoing prevention and control of the COVID-19 pandemic: A case study of Qingdao, China | 10.1016/j.ocecoaman.2021.105974 |  | Not at all relevant |  |  |  |
| Practices for monitoring and responding to incoming data on self-injurious thoughts and behaviors in intensive longitudinal studies: A systematic review | 10.1016/j.cpr.2021.102098 | Review paper |  |  |  |  |
| Effectiveness of web-based and mobile-based psychological interventions to prevent perinatal depression: Study protocol for a systematic review and meta-analysis of randomized controlled trials | 10.1016/j.invent.2021.100471 | Review paper |  |  |  |  |
| Are online mental health interventions for youth effective? A systematic review | 10.1177/1357633X211047285 | Review paper |  |  |  |  |
| Effects of cranial electrotherapy stimulation with novel in-ear electrodes on anxiety and resting-state brain activity: A randomized double-blind placebo-controlled trial | 10.1016/j.jad.2021.08.141 |  | Not at all relevant |  |  |  |
| Quality evaluation of stress, anxiety and depression apps for COVID-19 | 10.1016/j.jadr.2021.100255 |  | Not at all relevant |  |  |  |
| Online mental health interventions designed for students in higher education: A user-centered perspective | 10.1016/j.invent.2021.100468 |  | Not at all relevant |  |  |  |
| Untangling the associations between generalized anxiety and body dissatisfaction: The mediating effects of social physique anxiety among collegiate men and women | 10.1016/j.bodyim.2021.10.002 |  | Not at all relevant |  |  |  |
| Efficacy of an internet-based psychological intervention for problem gambling and gambling disorder: Study protocol for a randomized controlled trial | 10.1016/j.invent.2021.100466 |  | Not at all relevant |  |  |  |
| Internet- and mobile-based interventions for the treatment of specific phobia: A systematic review and preliminary meta-analysis | 10.1016/j.invent.2021.100462 | Review paper |  |  |  |  |
| Mental contamination, disgust, and other negative emotions among survivors of sexual trauma: Results from a daily monitoring study | 10.1016/j.janxdis.2021.102477 |  | Not at all relevant |  |  |  |
| Sensitivity to change and minimal clinically important difference of Edinburgh postnatal depression scale | 10.1016/j.ajp.2021.102873 |  | Not at all relevant |  |  |  |
| Engagement with mobile health interventions for depression: A systematic review | 10.1016/j.invent.2021.100454 | Review paper |  |  |  |  |
| Therapeutic processes in digital interventions for anxiety: A systematic review and meta-analytic structural equation modeling of randomized controlled trials | 10.1016/j.cpr.2021.102084 | Review paper |  |  |  |  |
| Acceptance towards digital health interventions - Model validation and further development of the Unified Theory of Acceptance and Use of Technology | 10.1016/j.invent.2021.100459 |  | Not at all relevant |  |  |  |
| The effect of depression and anxiety symptom severity on clinical outcomes and app use in digital mental health treatments: Meta-regression of three trials | 10.1016/j.brat.2021.103972 |  | Not at all relevant |  |  |  |
| Anxiety and depression symptoms after COVID-19 infection: results from the COVID Symptom Study app | 10.1136/jnnp-2021-327565 |  | Not at all relevant |  |  |  |
| You're Worried, We're Listening: Online Testing of the Effectiveness of Education Materials to Improve Consumer Knowledge and Confidence in Reporting Patient Deterioration | 10.1097/PTS.0000000000000906 |  | Not at all relevant |  |  |  |
| [Which Digital Services do Psychosomatic Rehabilitation Patients Use During the Corona Pandemic and do Interrelations with Anxiety and Depressive Symptoms Exist?] | 10.1055/a-1503-5548 |  | Not at all relevant |  |  |  |
| Using an app to count calories: Motives, perceptions, and connections to thinness- and muscularity-oriented disordered eating | 10.1016/j.eatbeh.2021.101568 |  | Not at all relevant |  |  |  |
| The relationship between phubbing and the depression of primary and secondary school teachers: A moderated mediation model of rumination and job burnout | 10.1016/j.jad.2021.08.070 |  | Not at all relevant |  |  |  |
| Are eHealth interventions for adults who are scheduled for or have undergone bariatric surgery as effective as usual care? A systematic review | 10.1016/j.soard.2021.07.020 | Review paper |  |  |  |  |
| Discovering different profiles in the dynamics of depression based on real-time monitoring of mood: a first exploration | 10.1016/j.invent.2021.100437 |  | Not at all relevant |  |  |  |
| Perceptions towards an interaction partner predict social anxiety: an ecological momentary assessment study | 10.1080/02699931.2021.1969339 |  | Not at all relevant |  |  |  |
| Introducing Dietary Self-Monitoring to Undergraduate Women via a Calorie Counting App Has No Effect on Mental Health or Health Behaviors: Results From a Randomized Controlled Trial | 10.1016/j.jand.2021.06.311 |  | Not at all relevant |  |  |  |
| A tale of two surges: messaging app and public COVID-19 data summarize one anesthesiology practice's pandemic year in review | 10.1007/s12630-021-02088-x | Review paper |  |  |  |  |
| Managing depression in India: Opportunities for a targeted smartphone app | 10.1177/00207640211032253 |  | Not at all relevant |  |  |  |
| Pilot Study on Reducing Symptoms of Anxiety with a Heart Rate Variability Biofeedback Wearable and Remote Stress Management Coach | 10.1007/s10484-021-09519-x |  | Not at all relevant |  |  |  |
| Worldwide increases in adolescent loneliness | 10.1016/j.adolescence.2021.06.006 |  | Not at all relevant |  |  |  |
| Gratitude and patience moderate meaning struggles and suicidal risk in a cross-sectional study of inpatients at a Christian psychiatric hospital | 10.1111/jopy.12644 |  | Not at all relevant |  |  |  |
| Response to: 'Mindfulness-based mobile app reduces anxiety and increases self-compassion in healthcare students: A randomised controlled trial' | 10.1080/0142159X.2021.1914324 |  | Not at all relevant |  |  |  |
| Application of Mobile Call-Based Integrative Body-Mind-Spirit (IBMS) Intervention to Deal With Psychological Issues of COVID-19 Patients: A Case Study in India | 10.1177/0898010121993001 |  | Not at all relevant |  |  |  |
| Using Intensive Longitudinal Data to Identify Early Predictors of Suicide-Related Outcomes in High-Risk Adolescents: Practical and Conceptual Considerations | 10.1177/1073191120939168 |  | Not at all relevant |  |  |  |
| Using Ecological Momentary Assessment to Study the Development of COVID-19 Worries in Sweden: Longitudinal Study | 10.2196/26743 |  | Not at all relevant |  |  |  |
| Effectiveness of a Mobile Device-Based Resilience Training Program in Reducing Depressive Symptoms and Enhancing Resilience and Quality of Life in Parents of Children With Cancer: Randomized Controlled Trial | 10.2196/27639 |  | Not at all relevant |  |  |  |
| Examining the Effectiveness of Gamification in Mental Health Apps for Depression: Systematic Review and Meta-analysis | 10.2196/32199 | Review paper |  |  |  |  |
| Bridging juvenile justice and behavioral health systems: development of a clinical pathways approach to connect youth at risk for suicidal behavior to care | 10.1186/s40352-021-00164-4 |  | Not at all relevant |  |  |  |
| Evaluation of Android and Apple Store Depression Applications Based on Mobile Application Rating Scale | 10.3390/ijerph182312505 |  | Not at all relevant |  |  |  |
| Healthy Moms and Babies Preventive Psychological Intervention Application: A Study Protocol | 10.3390/ijerph182312485 |  | Not at all relevant |  |  |  |
| Smart Homes as Enablers for Depression Pre-Diagnosis Using PHQ-9 on HMI through Fuzzy Logic Decision System | 10.3390/s21237864 |  | Not at all relevant |  |  |  |
| An App-Based Mindfulness-Based Self-compassion Program to Support Caregivers of People With Dementia: Participatory Feasibility Study | 10.2196/28652 |  | Not at all relevant |  |  |  |
| Osteoblastic Swedish mutant APP expedites brain deficits by inducing endoplasmic reticulum stress-driven senescence | 10.1038/s42003-021-02843-2 |  | Not at all relevant |  |  |  |
| Longitudinal Smartphone-Based Post-hospitalisation Symptom Monitoring in SARS-CoV-2 Associated Respiratory Failure: A Multi-Centre Observational Study | 10.3389/fresc.2021.777396 |  | Not at all relevant |  |  |  |
| Machine Learning Methods for Predicting Postpartum Depression: Scoping Review | 10.2196/29838 | Review paper |  |  |  |  |
| The effectiveness of psychological support interventions for those exposed to mass infectious disease outbreaks: a systematic review | 10.1186/s12888-021-03602-7 | Review paper |  |  |  |  |
| Prokaryotic reverse transcriptases: from retroelements to specialized defense systems | 10.1093/femsre/fuab025 |  | Not at all relevant |  |  |  |
| Internet-Based Psychotherapy Intervention for Depression Among Older Adults Receiving Home Care: Qualitative Study of Participants' Experiences | 10.2196/27630 |  | Not at all relevant |  |  |  |
| Effectiveness of Self-Guided Virtual Reality-Based Cognitive Behavioral Therapy for Panic Disorder: Randomized Controlled Trial | 10.2196/30590 |  | Not at all relevant |  |  |  |
| Kukaa Salama (Staying Safe): study protocol for a pre/post-trial of an interactive mHealth intervention for increasing COVID-19 prevention practices with urban refugee youth in Kampala, Uganda | 10.1136/bmjopen-2021-055530 |  | Not at all relevant |  |  |  |
| Screening for Depression in Mobile Devices Using Patient Health Questionnaire-9 (PHQ-9) Data: A Diagnostic Meta-Analysis via Machine Learning Methods | 10.2147/NDT.S339412 |  | Not at all relevant |  |  |  |
| Smartphone-Delivered Ecological Momentary Interventions Based on Ecological Momentary Assessments to Promote Health Behaviors: Systematic Review and Adapted Checklist for Reporting Ecological Momentary Assessment and Intervention Studies | 10.2196/22890 | Review paper |  |  |  |  |
| The Effects of a Digital Mental Health Intervention in Adults With Cardiovascular Disease Risk Factors: Analysis of Real-World User Data | 10.2196/32351 |  | Not at all relevant |  |  |  |
| Adults who microdose psychedelics report health related motivations and lower levels of anxiety and depression compared to non-microdosers | 10.1038/s41598-021-01811-4 |  | Not at all relevant |  |  |  |
| Contextual Predictors of Engagement in a Tailored mHealth Intervention for Adolescent and Young Adult Cancer Survivors | 10.1093/abm/kaab008 |  | Not at all relevant |  |  |  |
| Evaluating a Strengths-Based mHealth Tool (MyStrengths): Explorative Feasibility Trial | 10.2196/30572 |  | Not at all relevant |  |  |  |
| Technology-Assisted Collaborative Care Program for People with Diabetes and/or High Blood Pressure Attending Primary Health Care: A Feasibility Study | 10.3390/ijerph182212000 |  | Not at all relevant |  |  |  |
| A Smartphone App for Supporting the Self-management of Daytime Urinary Incontinence in Adolescents: Development and Formative Evaluation Study of URApp | 10.2196/26212 |  | Not at all relevant |  |  |  |
| Repetitive Low-Level Blast Exposure Improves Behavioral Deficits and Chronically Lowers Aβ42 in an Alzheimer Disease Transgenic Mouse Model | 10.1089/neu.2021.0184 |  | Not at all relevant |  |  |  |
| A Smartphone Intervention for People With Serious Mental Illness: Fully Remote Randomized Controlled Trial of CORE | 10.2196/29201 |  | Not at all relevant |  |  |  |
| Case Report: A gambling-related suicide in rural Malawi | 10.12688/wellcomeopenres.17333.1 |  | Not at all relevant |  |  |  |
| Mobile Diary App Versus Paper-Based Diary Cards for Patients With Borderline Personality Disorder: Economic Evaluation | 10.2196/28874 |  | Not at all relevant |  |  |  |
| Do Anxiety and Depression Predict Persistent Physical Symptoms After a Severe COVID-19 Episode? A Prospective Study | 10.3389/fpsyt.2021.757685 |  | Not at all relevant |  |  |  |
| Development of a Severity Score and Comparison With Validated Measures for Depression and Anxiety: Validation Study | 10.2196/30313 |  | Not at all relevant |  |  |  |
| Mediating Effects of Stigma and Depressive Symptoms in a Social Media-Based Intervention to Improve Long-term Quality of Life Among People Living With HIV: Secondary Analysis of a Randomized Controlled Trial | 10.2196/27897 |  | Not at all relevant |  |  |  |
| Exploring the Barriers to and Motivators for Using Digital Mental Health Interventions Among Construction Personnel in Nigeria: Qualitative Study | 10.2196/18969 |  | Not at all relevant |  |  |  |
| The impact of participant mental health on attendance and engagement in a trial of behavioural weight management programmes: secondary analysis of the WRAP randomised controlled trial | 10.1186/s12966-021-01216-6 |  | Not at all relevant |  |  |  |
| Application of the Unified Protocol for a Japanese Patient with Post-Traumatic Stress Disorder and Multiple Comorbidities: A Single-Case Study | 10.3390/ijerph182111644 |  | Not at all relevant |  |  |  |
| Acute objective and subjective intoxication effects of legal-market high potency THC-dominant versus CBD-dominant cannabis concentrates | 10.1038/s41598-021-01128-2 |  | Not at all relevant |  |  |  |
| GPS Mobile Health Intervention Among People Experiencing Homelessness: Pre-Post Study | 10.2196/25553 |  | Not at all relevant |  |  |  |
| Risk Factors, Trends, and Preventive Measures for 30-Day Unplanned Diabetic Ketoacidosis Readmissions in the Pediatric Population | 10.7759/cureus.19205 |  | Not at all relevant |  |  |  |
| The psychological impact of COVID-19 infection on athletes: example of professional male football players | 10.1080/24733938.2021.1933156 |  | Not at all relevant |  |  |  |
| Leveraging Longitudinal Lifelog Data Using Survival Models for Predicting Risk of Relapse among Patients with Depression in Remission | 10.1109/EMBC46164.2021.9629798 |  | Not at all relevant |  |  |  |
| Classification of Depression and Other Psychiatric Conditions Using Speech Features Extracted from a Thai Psychiatric and Verbal Screening Test | 10.1109/EMBC46164.2021.9629571 |  | Not at all relevant |  |  |  |
| Clustering and Feature Analysis of Smartphone Data for Depression Monitoring | 10.1109/EMBC46164.2021.9629737 |  | Not at all relevant |  |  |  |
| COVID-19: Affect recognition through voice analysis during the winter lockdown in Scotland | 10.1109/EMBC46164.2021.9630833 |  | Not at all relevant |  |  |  |
| An Evaluation of 5-Year Web Analytics for HeadsUpGuys: A Men's Depression E-Mental Health Resource | 10.1177/15579883211063322 |  | Not at all relevant |  |  |  |
| Loneliness unlocked: Associations with smartphone use and personality | 10.1016/j.actpsy.2021.103454 |  | Not at all relevant |  |  |  |
| In Search of Digital Dopamine: How Apps Can Motivate Depressed Patients, a Review and Conceptual Analysis | 10.3390/brainsci11111454 | Review paper |  |  |  |  |
| Information processing by community health nurses using mobile health (mHealth) tools for early identification of suicide and depression risks in Fiji Islands | 10.1136/bmjhci-2021-100342 |  | Not at all relevant |  |  |  |
| Induction of acute stress through an internet-delivered Trier Social Stress Test as assessed by photoplethysmography on a smartphone | 10.1080/10253890.2021.1995714 |  | Not at all relevant |  |  |  |
| Machine learning-augmented objective functional testing in the degenerative spine: quantifying impairment using patient-specific five-repetition sit-to-stand assessment | 10.3171/2021.8.FOCUS21386 |  | Not at all relevant |  |  |  |
| Delivering Cognitive Behavioral Therapy for Post-Intensive Care Syndrome-Family via a Mobile Health App | 10.4037/ajcc2021962 |  |  |  |  |  |
| Acceptability and Preliminary Effects of a Mindfulness Mobile Application for Ruminative Adolescents | 10.1016/j.beth.2021.03.004 |  | Not at all relevant |  |  |  |
| Naturalistic smartphone keyboard typing reflects processing speed and executive function | 10.1002/brb3.2363 |  | Not at all relevant |  |  |  |
| Coenzyme Q10 a mitochondrial restorer for various brain disorders | 10.1007/s00210-021-02161-8 |  | Not at all relevant |  |  |  |
| Acceptability, Engagement, and Exploratory Outcomes of an Emotional Well-being App: Mixed Methods Preliminary Evaluation and Descriptive Analysis | 10.2196/31064 |  | Not at all relevant |  |  |  |
| A Text Messaging Intervention (StayWell at Home) to Counteract Depression and Anxiety During COVID-19 Social Distancing: Pre-Post Study | 10.2196/25298 |  | Not at all relevant |  |  |  |
| A SMART approach to optimizing delivery of an mHealth intervention among cancer survivors with posttraumatic stress symptoms | 10.1016/j.cct.2021.106569 |  | Not at all relevant |  |  |  |
| How Has COVID-19-Related Income Loss and Household Stress Affected Adolescent Mental Health in Kenya? | 10.1016/j.jadohealth.2021.07.023 |  | Not at all relevant |  |  |  |
| Ecological momentary assessment of affect in context after traumatic brain injury | 10.1037/rep0000403 |  | Not at all relevant |  |  |  |
| A prospective examination of sex differences in posttraumatic autonomic functioning | 10.1016/j.ynstr.2021.100384 |  | Not at all relevant |  |  |  |
| High-fat diet alters stress behavior, inflammatory parameters and gut microbiota in Tg APP mice in a sex-specific manner | 10.1016/j.nbd.2021.105495 |  | Not at all relevant |  |  |  |
| Can platelet activation result in increased plasma Aβ levels and contribute to the pathogenesis of Alzheimer's disease? | 10.1016/j.arr.2021.101420 |  | Not at all relevant |  |  |  |
| Design and Redesign of the IACTA App, an Interactive Communication Tool Intended to Facilitate Young Children's Participation in Healthcare Situations | 10.1016/j.pedn.2021.07.015 |  | Not at all relevant |  |  |  |
| Traditional vs Extended Hybrid Cardiac Rehabilitation Based on the Continuous Care Model for Patients Who Have Undergone Coronary Artery Bypass Surgery in a Middle-Income Country: A Randomized Controlled Trial | 10.1016/j.apmr.2021.04.026 |  | Not at all relevant |  |  |  |
| In-person victimization, cyber victimization, and polyvictimization in relation to internalizing symptoms and self-esteem in adolescents with attention-deficit/hyperactivity disorder | 10.1111/cch.12888 |  | Not at all relevant |  |  |  |
| A Preliminary Test of an mHealth Facilitated Health Coaching Intervention to Improve Medication Adherence among Persons Living with HIV | 10.1007/s10461-021-03342-5 |  | Not at all relevant |  |  |  |
| Prevalences of comorbid anxiety disorder and daily smartphone-based self-reported anxiety in patients with newly diagnosed bipolar disorder | 10.1136/ebmental-2021-300259 |  | Not at all relevant |  |  |  |
| Gender differences between adolescents with autism in emergency psychiatry | 10.1177/13623613211019855 |  | Not at all relevant |  |  |  |
| Multiple environmental exposures along daily mobility paths and depressive symptoms: A smartphone-based tracking study | 10.1016/j.envint.2021.106635 |  | Not at all relevant |  |  |  |
| Reviewing the availability, efficacy and clinical utility of Telepsychology in dialectical behavior therapy (Tele-DBT) | 10.1186/s40479-021-00165-7 | Review paper |  |  |  |  |
| Mobile interventions targeting common mental disorders among pregnant and postpartum women: An equity-focused systematic review | 10.1371/journal.pone.0259474 | Review paper |  |  |  |  |
| Supportive Care Interventions for People With Cancer Assisted by Digital Technology: Systematic Review | 10.2196/24722 | Review paper |  |  |  |  |
| Using virtual patient to assess primary health workers' competence to detect postpartum depression in Hunan, China | 10.11817/j.issn.1672-7347.2021.210139 |  | Not at all relevant |  |  |  |
| A Counseling Application as an Alternative Tool in Increasing Coping Self-Efficacy Among University Students With Academic Distress During Coronavirus Disease 2019 Pandemic in Indonesia: A Study Protocol for a Randomized Controlled Non-Inferiority Trial | 10.3389/fpsyg.2021.712806 |  | Not at all relevant |  |  |  |
| Use of Passive Sensing in Psychotherapy Studies in Late Life: A Pilot Example, Opportunities and Challenges | 10.3389/fpsyt.2021.732773 |  | Not at all relevant |  |  |  |
| Association of Autism Spectrum Disorder and Attention Deficit Hyperactivity Disorder Traits with Depression and Empathy Among Medical Students | 10.2147/AMEP.S334155 |  | Not at all relevant |  |  |  |
| Toward an Extended Definition of Major Depressive Disorder Symptomatology: Digital Assessment and Cross-validation Study | 10.2196/27908 |  | Not at all relevant |  |  |  |
| Adults with higher social anxiety show avoidant gaze behaviour in a real-world social setting: A mobile eye tracking study | 10.1371/journal.pone.0259007 |  | Not at all relevant |  |  |  |
| Evaluating the Clinical Feasibility of an Artificial Intelligence-Powered, Web-Based Clinical Decision Support System for the Treatment of Depression in Adults: Longitudinal Feasibility Study | 10.2196/31862 |  | Not at all relevant |  |  |  |
| Impact of an eHealth Smartphone App on the Mental Health of Patients With Psoriasis: Prospective Randomized Controlled Intervention Study | 10.2196/28149 |  | Not at all relevant |  |  |  |
| Development and Pilot Testing of a Smartphone-Based Self-Care Program for Patients with Chronic Hepatitis B | 10.3390/ijerph182111139 |  | Not at all relevant |  |  |  |
| Internet Search Activity of Young People With Mood Disorders Who Are Hospitalized for Suicidal Thoughts and Behaviors: Qualitative Study of Google Search Activity | 10.2196/28262 |  | Not at all relevant |  |  |  |
| Reducing worry and rumination in young adults via a mobile phone app: study protocol of the ECoWeB (Emotional Competence for Well-Being in Young Adults) randomised controlled trial focused on repetitive negative thinking | 10.1186/s12888-021-03536-0 |  | Not at all relevant |  |  |  |
| Academic Burnout and Problematic Smartphone Use During the COVID-19 Pandemic: The Effects of Anxiety and Resilience | 10.3389/fpsyt.2021.725740 |  | Not at all relevant |  |  |  |
| A Mobile App to Identify Lifestyle Indicators Related to Undergraduate Mental Health (Smart Healthy Campus): Observational App-Based Ecological Momentary Assessment | 10.2196/29160 |  | Not at all relevant |  |  |  |
| Validity and Reliability of the Self-administered Psycho-TherApy-SystemS (SELFPASS) Item Pool for the Daily Mood Tracking of Depressive Symptoms: Cross-sectional Web-Based Survey | 10.2196/29615 |  | Not at all relevant |  |  |  |
| The Mediation Effect of Phobic Anxiety on the Treatment Outcome of Activity and Participation across Age: Comparison between Online and Face-to-Face Rehabilitation Aftercare of an RCT | 10.3390/ijerph182010919 |  | Not at all relevant |  |  |  |
| Loss of APP in mice increases thigmotaxis and is associated with elevated brain expression of IL-13 and IP-10/CXCL10 | 10.1016/j.physbeh.2021.113533 |  | Not at all relevant |  |  |  |
| Mental Illness and Amyloid: A Scoping Review of Scientific Evidence over the Last 10 Years (2011 to 2021) | 10.3390/brainsci11101352 | Review paper |  |  |  |  |
| Acceptability, Engagement, and Effects of a Mobile Digital Intervention to Support Mental Health for Young Adults Transitioning to College: Pilot Randomized Controlled Trial | 10.2196/32271 |  | Not at all relevant |  |  |  |
| The impact of data from remote measurement technology on the clinical practice of healthcare professionals in depression, epilepsy and multiple sclerosis: survey | 10.1186/s12911-021-01640-5 |  | Not at all relevant |  |  |  |
| Text Messaging Versus Email Messaging to Support Patients With Major Depressive Disorder: Protocol for a Randomized Hybrid Type II Effectiveness-Implementation Trial | 10.2196/29495 |  | Not at all relevant |  |  |  |
| A Mobile Health Intervention for Patients With Depressive Symptoms: Protocol for an Economic Evaluation Alongside Two Randomized Trials in Brazil and Peru | 10.2196/26164 |  | Not at all relevant |  |  |  |
| The Use of Task Shifting to Improve Treatment Engagement in an Internet-Based Mindfulness Intervention Among Chinese University Students: Randomized Controlled Trial | 10.2196/25772 |  | Not at all relevant |  |  |  |
| Obstacle Avoidance of Multi-Sensor Intelligent Robot Based on Road Sign Detection | 10.3390/s21206777 |  | Not at all relevant |  |  |  |
| Clinician-Created Educational Video Resources for Shared Decision-making in the Outpatient Management of Chronic Disease: Development and Evaluation Study | 10.2196/26732 |  | Not at all relevant |  |  |  |
| Reaching people soon after a traumatic event: an exploratory observational feasibility study of recruitment in the emergency department to deliver a brief behavioral intervention via smartphone to prevent intrusive memories of trauma | 10.1186/s40814-021-00916-x |  | Not at all relevant |  |  |  |
| The Functionality of Mobile Apps for Anxiety: Systematic Search and Analysis of Engagement and Tailoring Features | 10.2196/26712 | Review paper |  |  |  |  |
| Worry and Positive Episodes in the Daily Lives of Individuals With Generalized Anxiety Disorder: An Ecological Momentary Assessment Study | 10.3389/fpsyg.2021.722881 |  | Not at all relevant |  |  |  |
| Use of a Guided Imagery Mobile App (See Me Serene) to Reduce COVID-19-Related Stress: Pilot Feasibility Study | 10.2196/32353 |  | Not at all relevant |  |  |  |
| Cognitive-behavioral therapy for management of mental health and stress-related disorders: Recent advances in techniques and technologies | 10.1186/s13030-021-00219-w |  | Not at all relevant |  |  |  |
| App-based Mindfulness Training for Adolescent Rumination: Predictors of Immediate and Cumulative Benefit | 10.1007/s12671-021-01719-0 |  | Not at all relevant |  |  |  |
| Heart rate trajectories in patients recovering from acute myocardial infarction: A longitudinal analysis of Apple Watch heart rate recordings | 10.1016/j.cvdhj.2021.05.003 |  | Not at all relevant |  |  |  |
| Community-based participatory research with police: Development of a tech-enhanced structured suicide risk assessment and communication smartphone application | 10.1037/lhb0000470 |  | Not at all relevant |  |  |  |
| Promoting Physical Activity in Rural Settings: Effectiveness and Potential Strategies | 10.1249/tjx.0000000000000180 |  | Not at all relevant |  |  |  |
| Assessment of Hypertension Control Among Adults Participating in a Mobile Technology Blood Pressure Self-management Program | 10.1001/jamanetworkopen.2021.27008 |  | Not at all relevant |  |  |  |
| The development of the postpartum mobile support application and the effect of the application on mothers' anxiety and depression symptoms | 10.1016/j.apnu.2021.06.009 |  | Not at all relevant |  |  |  |
| Relationships between daily mood states and real-time cognitive performance in individuals with bipolar disorder and healthy comparators: A remote ambulatory assessment study | 10.1080/13803395.2021.1975656 |  | Not at all relevant |  |  |  |
| Measuring interoception: The phase adjustment task | 10.1016/j.biopsycho.2021.108171 |  | Not at all relevant |  |  |  |
| Use of smartphones, mobile apps and wearables for health promotion by people with anxiety or depression: An analysis of a nationally representative survey data | 10.1016/j.psychres.2021.114120 |  | Not at all relevant |  |  |  |
| Patient Satisfaction Through an Immersive Experience Using a Mobile Phone-Based Head-Mounted Display During Arthroscopic Knee Surgery Under Spinal Anesthesia: A Randomized Clinical Trial | 10.1213/ANE.0000000000005666 |  | Not at all relevant |  |  |  |
| Factors associated with orthodontic pain | 10.1111/joor.13227 |  | Not at all relevant |  |  |  |
| The effectiveness of a nurse-led home-based heart failure self-management programme (the HOM-HEMP) for patients with chronic heart failure: A three-arm stratified randomized controlled trial | 10.1016/j.ijnurstu.2021.104026 |  | Not at all relevant |  |  |  |
| Persistence of symptoms after improvement of acute COVID19 infection, a longitudinal study | 10.1002/jmv.27156 |  | Not at all relevant |  |  |  |
| VidaTalk™ patient communication application "opened up" communication between nonvocal ICU patients and their family | 10.1016/j.iccn.2021.103075 |  | Not at all relevant |  |  |  |
| Promoting Culturally Tailored mHealth: A Scoping Review of Mobile Health Interventions in Latinx Communities | 10.1007/s10903-021-01209-4 | Review paper |  |  |  |  |
| The impact of caregiver stigma on real-life social experience of Taiwanese adolescents with autism spectrum disorder | 10.1177/13623613211004329 |  | Not at all relevant |  |  |  |
| Vascular Dysfunction after Modeled Traumatic Brain Injury Is Preserved with Administration of Umbilical Cord Derived Mesenchymal Stromal Cells and Is Associated with Modulation of the Angiogenic Response | 10.1089/neu.2021.0158 |  | Not at all relevant |  |  |  |
| Hospitalist perspectives on barriers to recommend and potential benefit of the COVID-19 vaccine | 10.1080/21548331.2021.1914465 |  | Not at all relevant |  |  |  |
| Resourcefulness revisited: Further psychometric evaluation of resourcefulness scale | 10.1002/smi.3024 |  | Not at all relevant |  |  |  |
| Toward a Typology of Transnational Communication among Venezuelan Immigrant Youth: Implications for Behavioral Health | 10.1007/s10903-020-01099-y |  | Not at all relevant |  |  |  |
| The assessment of capacity limitations in psychiatric work disability evaluations by the social functioning scale Mini-ICF-APP | 10.1186/s12888-021-03467-w |  | Not at all relevant |  |  |  |
| Prevention of Suicidal Relapses in Adolescents With a Smartphone Application: Bayesian Network Analysis of a Preclinical Trial Using In Silico Patient Simulations | 10.2196/24560 |  | Not at all relevant |  |  |  |
| Moral injury in civilians: associations with trauma exposure, PTSD, and suicide behavior | 10.1080/20008198.2021.1965464 |  | Not at all relevant |  |  |  |
| Health Care Provider Perspectives on the Use of a Digital Behavioral Health App to Support Patients: Qualitative Study | 10.2196/28538 |  | Not at all relevant |  |  |  |
| Just-in-Time Adaptive Mechanisms of Popular Mobile Apps for Individuals With Depression: Systematic App Search and Literature Review | 10.2196/29412 | Review paper |  |  |  |  |
| Psychosocial and Behavioral Factors in Awake Bruxism-Self-Report versus Ecological Momentary Assessment | 10.3390/jcm10194447 |  | Not at all relevant |  |  |  |
| The relation between oxytocin receptor gene polymorphisms, adult attachment and Instagram sociability: An exploratory analysis | 10.1016/j.heliyon.2021.e07894 |  | Not at all relevant |  |  |  |
| Determinants of the Use of Health and Fitness Mobile Apps by Patients With Asthma: Secondary Analysis of Observational Studies | 10.2196/25472 |  | Not at all relevant |  |  |  |
| Facilitator Contact, Discussion Boards, and Virtual Badges as Adherence Enhancements to a Web-Based, Self-guided, Positive Psychological Intervention for Depression: Randomized Controlled Trial | 10.2196/25922 |  | Not at all relevant |  |  |  |
| Comparing a Virtual Reality-Based Simulation App (VR-MRI) With a Standard Preparatory Manual and Child Life Program for Improving Success and Reducing Anxiety During Pediatric Medical Imaging: Randomized Clinical Trial | 10.2196/22942 |  | Not at all relevant |  |  |  |
| Psychoeducational Social Anxiety Mobile Apps: Systematic Search in App Stores, Content Analysis, and Evaluation | 10.2196/26603 |  | Not at all relevant |  |  |  |
| Evaluation of a Commercial Mobile Health App for Depression and Anxiety (AbleTo Digital+): Retrospective Cohort Study | 10.2196/27570 |  | Not at all relevant |  |  |  |
| Pain Distraction During Awake Major Colorectal Surgery: Supporting Patients Beyond the COVID-19 Era. Preliminary Findings | 10.3389/fsurg.2021.754059 |  | Not at all relevant |  |  |  |
| Effectiveness of a customised mobile phone text messaging intervention supported by data from activity monitors for improving lifestyle factors related to the risk of type 2 diabetes among women after gestational diabetes: protocol for a multicentre randomised controlled trial (SMART MUMS with smart phones 2) | 10.1136/bmjopen-2021-054756 |  | Not at all relevant |  |  |  |
| Cross-Disorder Genomics Data Analysis Elucidates a Shared Genetic Basis Between Major Depression and Osteoarthritis Pain | 10.3389/fgene.2021.687687 |  | Not at all relevant |  |  |  |
| Alpha activity neuromodulation induced by individual alpha-based neurofeedback learning in ecological context: a double-blind randomized study | 10.1038/s41598-021-96893-5 |  | Not at all relevant |  |  |  |
| A Cyberbullying Media-Based Prevention Intervention for Adolescents on Instagram: Pilot Randomized Controlled Trial | 10.2196/26029 |  | Not at all relevant |  |  |  |
| Social Media Use, Physical Activity, and Internalizing Symptoms in Adolescence: Cross-sectional Analysis | 10.2196/26134 |  | Not at all relevant |  |  |  |
| Shift in Social Media App Usage During COVID-19 Lockdown and Clinical Anxiety Symptoms: Machine Learning-Based Ecological Momentary Assessment Study | 10.2196/30833 |  | Not at all relevant |  |  |  |
| A Social Media Group Cognitive Behavioral Therapy Intervention to Prevent Depression in Perinatal Youth: Stakeholder Interviews and Intervention Design | 10.2196/26188 |  | Not at all relevant |  |  |  |
| A qualitative exploration of the feasibility of incorporating depression apps into integrated primary care clinics | 10.1093/tbm/ibab075 |  | Not at all relevant |  |  |  |
| A Suite of Mobile Conversational Agents for Daily Stress Management (Popbots): Mixed Methods Exploratory Study | 10.2196/25294 |  | Not at all relevant |  |  |  |
| Mobile-based interventions for common mental disorders in youth: a systematic evaluation of pediatric health apps | 10.1186/s13034-021-00401-6 |  | Not at all relevant |  |  |  |
| Factors Associated With Longitudinal Psychological and Physiological Stress in Health Care Workers During the COVID-19 Pandemic: Observational Study Using Apple Watch Data | 10.2196/31295 |  | Not at all relevant |  |  |  |
| Effect of a personalised mHealth home-based training application on physical activity levels during and after centre-based cardiac rehabilitation: rationale and design of the Cardiac RehApp randomised control trial | 10.1136/bmjsem-2021-001159 |  | Not at all relevant |  |  |  |
| Facilitating improvements in young people's social relationships to prevent or treat depression: there's an app for that | 10.1038/s41398-021-01597-z |  | Not at all relevant |  |  |  |
| The PostStroke-Manager - combining mobile, digital and sensor-based technology with personal assistance: protocol of the feasibility study | 10.1186/s42466-021-00137-w |  | Not at all relevant |  |  |  |
| Impact of COVID-19 pandemic on migraine management in the United States: insights from migraine tracking app users | 10.1186/s12883-021-02378-3 |  | Not at all relevant |  |  |  |
| Association Between Psychological Distress, Cognitive Complaints, and Neuropsychological Status After a Severe COVID-19 Episode: A Cross-Sectional Study | 10.3389/fpsyt.2021.725861 |  | Not at all relevant |  |  |  |
| Evaluation of Changes in Depression, Anxiety, and Social Anxiety Using Smartphone Sensor Features: Longitudinal Cohort Study | 10.2196/22844 |  | Not at all relevant |  |  |  |
| Predicting Emotional Valence of People Living with the Human Immunodeficiency Virus Using Daily Voice Clips: A Preliminary Study | 10.3390/healthcare9091148 |  | Not at all relevant |  |  |  |
| Physical Exercise and Psychological Distress: The Mediating Roles of Problematic Mobile Phone Use and Learning Burnout among Adolescents | 10.3390/ijerph18179261 |  | Not at all relevant |  |  |  |
| Ecological Momentary Assessment of Bipolar Disorder Symptoms and Partner Affect: Longitudinal Pilot Study | 10.2196/30472 |  | Not at all relevant |  |  |  |
| Implementing mHealth-Enabled Integrated Care for Complex Chronic Patients With Osteoarthritis Undergoing Primary Hip or Knee Arthroplasty: Prospective, Two-Arm, Parallel Trial | 10.2196/28320 |  | Not at all relevant |  |  |  |
| Enhancing smart healthcare in dentistry: an approach to managing patients' stress | 10.1080/17538157.2021.1893322 |  | Not at all relevant |  |  |  |
| Technological prescription: evaluation of the effectiveness of mobile applications to improve depression and anxiety. Systematic review | 10.1080/17538157.2021.1887196 | Review paper |  |  |  |  |
| (Mis)information and anxiety: Evidence from a randomized Covid-19 information campaign | 10.1016/j.jdeveco.2021.102699 |  | Not at all relevant |  |  |  |
| Can Approaching Anxiety Like a Habit Lead to Novel Treatments? | 10.1177/15598276211008144 |  | Not at all relevant |  |  |  |
| Smartphone Screen Time Among University Students in Lebanon and Its Association With Insomnia, Bedtime Procrastination, and Body Mass Index During the COVID-19 Pandemic: A Cross-Sectional Study | 10.30773/pi.2021.0120 |  | Not at all relevant |  |  |  |
| Reminder through mobile messaging application improves outpatient attendance and medication adherence among patients with depression: An open-label randomised controlled trial |  |  | Not at all relevant |  |  |  |
| Factors Associated With a Negative Emotional Response to News Media and Nationwide Emergency Text Alerts During the COVID-19 Outbreak in Korea | 10.30773/pi.2021.0087 |  | Not at all relevant |  |  |  |
| Effect of a Telecare Case Management Program for Older Adults Who Are Homebound During the COVID-19 Pandemic: A Pilot Randomized Clinical Trial | 10.1001/jamanetworkopen.2021.23453 |  | Not at all relevant |  |  |  |
| Applying digital technology to promote active and healthy confinement lifestyle during pandemics in the elderly | 10.5114/biolsport.2021.100149 |  | Not at all relevant |  |  |  |
| Findings From a Provider-Led, Mindfulness-Based, Internet-Streamed Yoga Video Addressing the Psychological Outcomes of Breast Cancer Survivors | 10.1097/HNP.0000000000000465 |  | Not at all relevant |  |  |  |
| Terms and conditions apply: Critical issues for readability and jargon in mental health depression apps | 10.1016/j.invent.2021.100433 |  | Not at all relevant |  |  |  |
| Is clinician-supported use of a mindfulness smartphone app a feasible treatment for depression? A mixed-methods feasibility study | 10.1016/j.invent.2021.100413 |  | Not at all relevant |  |  |  |
| Significant reduction in depressive symptoms among patients with moderately-severe to severe depressive symptoms after participation in a therapist-supported, evidence-based mobile health program delivered via a smartphone app | 10.1016/j.invent.2021.100408 |  | Not at all relevant |  |  |  |
| Targeting subjective engagement in experimental therapeutics for digital mental health interventions | 10.1016/j.invent.2021.100403 |  | Not at all relevant |  |  |  |
| Engagement with smartphone-delivered behavioural activation interventions: a study of the MoodMission smartphone application | 10.1017/S1352465820000922 |  | Not at all relevant |  |  |  |
| A mobile mindfulness intervention for emergency department staff to improve stress and wellbeing: A qualitative study | 10.1016/j.ienj.2021.101039 |  | Not at all relevant |  |  |  |
| Efficacy of a Web App-Based Music Intervention During Cataract Surgery: A Randomized Clinical Trial | 10.1001/jamaophthalmol.2021.2767 |  | Not at all relevant |  |  |  |
| Suicide in the COVID-19 pandemic: What we learnt and great expectations | 10.1016/j.euroneuro.2021.06.005 |  | Not at all relevant |  |  |  |
| The effects of psychotherapies for depression on response, remission, reliable change, and deterioration: A meta-analysis | 10.1111/acps.13335 |  | Not at all relevant |  |  |  |
| Mobile Health Application for Patients Undergoing Breast Cancer Surgery: Feasibility Study | 10.1200/OP.20.01026 |  | Not at all relevant |  |  |  |
| Crowdsourcing to design a marketing package to promote a WHO digital mental health intervention among Chinese young adults | 10.1016/j.invent.2021.100397 |  | Not at all relevant |  |  |  |
| Quantifying Patient Engagement in Total Joint Arthroplasty Using Digital Application-Based Technology | 10.1016/j.arth.2021.04.022 |  | Not at all relevant |  |  |  |
| Incorporating Virtual Reality to Improve Otolaryngology Resident Wellness: One Institution's Experience | 10.1002/lary.29529 |  | Not at all relevant |  |  |  |
| "A link to the outside:" Patient perspectives on a mobile texting program to improve depression self-management | 10.1016/j.pec.2021.02.018 |  | Not at all relevant |  |  |  |
| Changes in work and life patterns associated with depressive symptoms during the COVID-19 pandemic: an observational study of health app (CALO mama) users | 10.1136/oemed-2020-106945 |  | Not at all relevant |  |  |  |
| Ecological momentary assessment to explore fatigue, mood and physical activity levels in people receiving peritoneal dialysis: A study protocol | 10.1177/0896860821992243 |  | Not at all relevant |  |  |  |
| Predicting Non-Suicidal Self-Injury in Young Adults with and without Borderline Personality Disorder: a Multilevel Approach Combining Ecological Momentary Assessment and Self-Report Measures | 10.1007/s11126-020-09875-7 |  | Not at all relevant |  |  |  |
| A Mixed-Method Assessment of a 10-Day Mobile Mindfulness Intervention | 10.3389/fpsyg.2021.722995 |  | Not at all relevant |  |  |  |
| The Effects of a Social Participation App on Seniors | 10.1097/JNR.0000000000000451 |  | Not at all relevant |  |  |  |
| Exercise Management Using a Mobile App in Patients With Parkinsonism: Prospective, Open-Label, Single-Arm Pilot Study | 10.2196/27662 |  | Not at all relevant |  |  |  |
| Erratum: Effects of Smartphone-Delivered Positive-Word Stimulation on Depressed Mood in People with Subthreshold Depression: Protocol for a Pilot Randomized Controlled Trial [Corrigendum] | 10.2147/NDT.S336378 |  | Not at all relevant |  |  |  |
| Automated home-cage monitoring as a potential measure of sickness behaviors and pain-like behaviors in LPS-treated mice | 10.1371/journal.pone.0256706 |  | Not at all relevant |  |  |  |
| Psychoeducational Interventions for Caregivers of Persons With Multiple Sclerosis: Protocol for a Randomized Trial | 10.2196/30617 |  | Not at all relevant |  |  |  |
| Serotonin Transporter Gene Polymorphisms and Maternal Overprotection Regulate Adult Social Expectations on Close Relationships | 10.3390/brainsci11091123 |  | Not at all relevant |  |  |  |
| Automatically Generated Smartphone Data in Young Patients With Newly Diagnosed Bipolar Disorder and Healthy Controls | 10.3389/fpsyt.2021.559954 |  | Not at all relevant |  |  |  |
| Social and Mental Health Impact of Nuclear Disaster in Survivors: A Narrative Review | 10.3390/bs11080113 | Review paper |  |  |  |  |
| Effects of Smartphone-Delivered Positive-Word Stimulation on Depressed Mood in People with Subthreshold Depression: Protocol for a Pilot Randomized Controlled Trial | 10.2147/NDT.S323126 |  | Not at all relevant |  |  |  |
| Exploring the experiences of mental health professionals engaged in the adoption of mobile health technology in Irish mental health services | 10.1186/s12888-021-03426-5 |  | Not at all relevant |  |  |  |
| A randomized clinical trial of an eHealth intervention on anxiety in patients undergoing abdominal aortic aneurysm surgery | 10.1093/bjs/znab151 |  | Not at all relevant |  |  |  |
| Food insecurity, drug resistance and non-disclosure are associated with virologic non-suppression among HIV pregnant women on antiretroviral treatment | 10.1371/journal.pone.0256249 |  | Not at all relevant |  |  |  |
| Optimizing Social-Emotional-Communication Development in Infants of Mothers With Depression: Protocol for a Randomized Controlled Trial of a Mobile Intervention Targeting Depression and Responsive Parenting | 10.2196/31072 |  | Not at all relevant |  |  |  |
| The Roles of General Health and COVID-19 Proximity in Contact Tracing App Usage: Cross-sectional Survey Study | 10.2196/27892 |  | Not at all relevant |  |  |  |
| Development of a Mobile App to Support Self-management of Anxiety and Depression in African American Women: Usability Study | 10.2196/24393 |  |  | App usability |  |  |
| Archaeal tyrosine recombinases | 10.1093/femsre/fuab004 |  | Not at all relevant |  |  |  |
| Commercial Off-The-Shelf Video Games for Reducing Stress and Anxiety: Systematic Review | 10.2196/28150 | Review paper |  |  |  |  |
| Development of Digitally Obtainable 10-Year Risk Scores for Depression and Anxiety in the General Population | 10.3389/fpsyt.2021.689026 |  | Not at all relevant |  |  |  |
| Development of a Mobile Health App (TOGETHERCare) to Reduce Cancer Care Partner Burden: Product Design Study | 10.2196/22608 |  | Not at all relevant |  |  |  |
| Measuring the Change in Health-Related Quality of Life in Patients Using Marijuana for Pain Relief | 10.1159/000517857 |  | Not at all relevant |  |  |  |
| Depressed and Socioeconomically Disadvantaged Mothers' Progression Into a Randomized Controlled Mobile Mental Health and Parenting Intervention: A Descriptive Examination Prior to and During COVID-19 | 10.3389/fpsyg.2021.719149 |  | Not at all relevant |  |  |  |
| Psychometric Validation of the Arabic Fear of Illness and Virus Evaluation | 10.3390/ijerph18168529 |  | Not at all relevant |  |  |  |
| Validity of the Aktibipo Self-rating Questionnaire for the Digital Self-assessment of Mood and Relapse Detection in Patients With Bipolar Disorder: Instrument Validation Study | 10.2196/26348 |  | Not at all relevant |  |  |  |
| Emerging Needs and Viability of Telepsychiatry During and Post COVID-19 Era: A Literature Review | 10.7759/cureus.16974 | Review paper |  |  |  |  |
| A Walk in the Park? Examining the Impact of App-Based Weather Warnings on Affective Reactions and the Search for Information in a Virtual City | 10.3390/ijerph18168353 |  | Not at all relevant |  |  |  |
| Young hospital pharmacists' job stress and career prospects amidst the COVID-19 pandemic in China | 10.1186/s40545-021-00355-2 |  | Not at all relevant |  |  |  |
| Childcare and depression during the coronavirus pandemic in South Africa: A gendered analysis | 10.1371/journal.pone.0255183 |  | Not at all relevant |  |  |  |
| A Compassion-Focused Ecological Momentary Intervention for Enhancing Resilience in Help-Seeking Youth: Uncontrolled Pilot Study | 10.2196/25650 |  | Not at all relevant |  |  |  |
| Smartphone Delivery of Cognitive Behavioral Therapy for Postintensive Care Syndrome-Family: Protocol for a Pilot Study | 10.2196/30813 |  | Not at all relevant |  |  |  |
| Patients' Experiences of Using a Self-help App for Posttraumatic Stress Disorder: Qualitative Study | 10.2196/26852 |  | Not at all relevant |  |  |  |
| Older Adult Use and Outcomes in a Digital Musculoskeletal (MSK) Program, by Generation | 10.3389/fdgth.2021.693170 |  | Not at all relevant |  |  |  |
| Acceptability of a digital return-to-work intervention for common mental disorders: a qualitative study on service user perspectives | 10.1186/s12888-021-03386-w |  | Not at all relevant |  |  |  |
| Web-Based Guidance Through Assisted Reproductive Technology (myFertiCare): Patient-Centered App Development and Qualitative Evaluation | 10.2196/25389 |  | Not at all relevant |  |  |  |
| Ecological Momentary Assessment and mHealth Interventions Among Men Who Have Sex With Men: Scoping Review | 10.2196/27751 | Review paper |  |  |  |  |
| Mobile Technology Improves Adherence to Cardiac Rehabilitation: A Propensity Score-Matched Study | 10.1161/JAHA.120.020482 |  | Not at all relevant |  |  |  |
| Priorities for Medical Marijuana Research from the Perspective of Physicians, Dispensary Owners/Staff, and Patients: A Survey Study | 10.1159/000518105 |  | Not at all relevant |  |  |  |
| Digital interventions for the treatment of depression: A meta-analytic review | 10.1037/bul0000334 | Review paper |  |  |  |  |
| Mobile app helps trainees manage emergencies at the bedside | 10.1002/aet2.10695 |  | Not at all relevant |  |  |  |
| Effects of Smartphone-Based Compensatory Cognitive Training and Physical Activity on Cognition, Depression, and Self-Esteem in Women with Subjective Cognitive Decline | 10.3390/brainsci11081029 |  | Not at all relevant |  |  |  |
| Psychological impacts during the COVID-19 outbreak among adult population in Jordan: A cross-sectional study | 10.1016/j.heliyon.2021.e07826 |  | Not at all relevant |  |  |  |
| Use of Data to Understand the Social Determinants of Depression in Two Middle-Income Countries: the 3-D Commission | 10.1007/s11524-021-00559-6 |  | Not at all relevant |  |  |  |
| Development of a Videogame for the Promotion of Active Aging Through Depression Prevention, Healthy Lifestyle Habits, and Cognitive Stimulation for Middle-to-Older Aged Adults | 10.1089/g4h.2020.0165 |  | Not at all relevant |  |  |  |
| Effectiveness of a smartphone-based, augmented reality exposure app to reduce fear of spiders in real-life: A randomized controlled trial | 10.1016/j.janxdis.2021.102442 |  | Not at all relevant |  |  |  |
| 'Routine' versus 'Smart Phone Application Based - Intense' follow up of patients with acute coronary syndrome undergoing percutaneous coronary intervention: Impact on clinical outcomes and patient satisfaction | 10.1016/j.ijcha.2021.100832 |  | Not at all relevant |  |  |  |
| Ecological momentary assessment of interpersonal theory of suicide constructs in people experiencing psychotic symptoms | 10.1016/j.jpsychires.2021.06.022 |  | Not at all relevant |  |  |  |
| Long-term effectiveness of an mHealth-tailored physical activity intervention in youth with congenital heart disease: A randomized controlled trial | 10.1111/jan.14924 |  | Not at all relevant |  |  |  |
| The Relationship Between Nomophobia and Anxiety Levels in Healthy Young Individuals | 10.3928/02793695-20210324-02 |  | Not at all relevant |  |  |  |
| Salinization depresses soil enzyme activity in metal-polluted soils through increases in metal mobilization and decreases in microbial biomass | 10.1007/s10646-021-02433-2 |  | Not at all relevant |  |  |  |
| Exploring the features of an app-based just-in-time intervention for depression | 10.1016/j.jad.2021.05.021 |  | Not at all relevant |  |  |  |
| The mental health status among nurses from low-risk areas under normalized COVID-19 pandemic prevention and control in China: A cross-sectional study | 10.1111/inm.12852 |  | Not at all relevant |  |  |  |
| Genetic knockdown of Klk8 has sex-specific multi-targeted therapeutic effects on Alzheimer's pathology in mice | 10.1111/nan.12687 |  | Not at all relevant |  |  |  |
| Social media and smartphone app use predicts maintenance of physical activity during Covid-19 enforced isolation in psychiatric outpatients | 10.1038/s41380-020-00963-5 |  | Not at all relevant |  |  |  |
| [Network model of mental disorders: Application and interest in post-stroke depression] | 10.1016/j.encep.2020.08.007 |  | Not at all relevant |  |  |  |
| Coping with COVID-19 in United Nations peacekeeping field hospitals: increased workload and mental stress for military healthcare providers | 10.1136/bmjmilitary-2020-001642 |  | Not at all relevant |  |  |  |
| A qualitative study on the use of mobile-based intervention for perinatal depression among perinatal mothers in rural Bihar, India | 10.1177/0020764020966003 |  | Not at all relevant |  |  |  |
| Sedentary behavior among breast cancer survivors: a longitudinal study using ecological momentary assessments | 10.1007/s11764-020-00948-x |  | Not at all relevant |  |  |  |
| SMARTphone-based, early cardiac REHABilitation in patients with acute coronary syndromes: a randomized controlled trial | 10.1097/MCA.0000000000000938 |  | Not at all relevant |  |  |  |
| Evaluation of a technology-enhanced intervention for older women with HIV infection: a proof of concept study | 10.1080/09540121.2020.1810617 |  | Not at all relevant |  |  |  |
| The Interplay of Tau Protein and β-Amyloid: While Tauopathy Spreads More Profoundly Than Amyloidopathy, Both Processes Are Almost Equally Pathogenic | 10.1007/s10571-020-00906-2 |  | Not at all relevant |  |  |  |
| Suicide Prevention Mobile Apps for Indian Users: An Overview | 10.7759/cureus.16770 |  | Not at all relevant |  |  |  |
| Supporting Patient-Clinician Interaction in Chronic HIV Care: Design and Development of a Patient-Reported Outcomes Software Application | 10.2196/27861 |  | Not at all relevant |  |  |  |
| Predicting Depressive Symptom Severity Through Individuals' Nearby Bluetooth Device Count Data Collected by Mobile Phones: Preliminary Longitudinal Study | 10.2196/29840 |  | Not at all relevant |  |  |  |
| Recommendations for the Design and Implementation of Virtual Reality for Acquired Brain Injury Rehabilitation: Systematic Review | 10.2196/26344 | Review paper |  |  |  |  |
| Self-guided Cognitive Behavioral Therapy Apps for Depression: Systematic Assessment of Features, Functionality, and Congruence With Evidence | 10.2196/27619 |  | Not at all relevant |  |  |  |
[truncated: 821,073 more chars]
