# Supplementary material for: Effectiveness of evidence based mental health apps on user health outcome: A systematic literature review
Source: PLoS One. 2025 Mar 25;20(3):e0319983. doi: 10.1371/journal.pone.0319983 (PMC11936281; doi:10.1371/journal.pone.0319983)
Supplement: Attachment — Submitted filename: report form plos one.docx [file pone.0319983.s005.docx]

This paper provides information about the effectiveness of evidence-based health apps on user mental health outcomes. The article provides information with high clinical relevance in the field of mental health interventions. However, some aspects must be improved to facilitate understanding. In this sense, I would like to add some comments/suggestions to this paper:

- It is recommended to include the protocol registration number (line 103).
- Why have authors limited the objective of the study only to these mental health problems (depression, anxiety, bipolar disorder, psychosis, and suicidal behaviors)?
- Some search terms (depression, anxiety, suicide, mobile, app, and smartphone) do not match the aim of the study (depression, anxiety, bipolar disorder, psychosis, and suicidal behaviors). Bipolar disorder and psychosis are not included as search terms.
- It is recommended to provide more information about the methodology for selecting articles (e.g., if it was done by title and abstract; if, in the event of not reaching an agreement, they included third parties in the decisión; what specific software was used to remove duplicates and for screening abstract).
- Were all the apps based on the cognitive behavioral approach? Did any applications include functions from other approaches? In order to better understand the results, it is recommended to include a table specifying the components of the different apps (e.g., mindfulness, physical activity tracking) for each CBT-function.
- It is recommended to specify the number of articles deleted by title and the number of articles deleted by abstract separately.
- Was any quality assessment carried out on the articles used? For example, through the Effective Public Health Practice Project Quality Assessment Tool (EPHPP)
- In Table 1 it is recommended to indicate the standard deviation of the age of the participants.
- It is recommended to review and unify the uppercase and lowercase letters used in the instruments column of table 1
- On lines 176-177 it is recommended to indicate the number of studies.
- The improvement in mental health outcomes is mentioned in the discussion section. However, few applications address suicidal behaviors. It is recommended that this section be redone, specifying mental health information obtained in more detail.
- Table 3 does not indicate whether the results obtained were short or long term.
