# Supplementary material for: Effectiveness of evidence based mental health apps on user health outcome: A systematic literature review
Source: PLoS One. 2025 Mar 25;20(3):e0319983. doi: 10.1371/journal.pone.0319983 (PMC11936281; doi:10.1371/journal.pone.0319983)
Supplement: Attachment — Submitted filename: Response to Reviewers.docx [file pone.0319983.s007.docx]

**Response to PLOS requirements**

1. Thank you for stating the following financial disclosure: “This work was supported by West Virginia University Internal Seed Grant (#3086)”Please state what role the funders took in the study. If the funders had no role, please state: "The funders had no role in study design, data collection and analysis, decision to publish, or preparation of the manuscript." If this statement is not correct you must amend it as needed. Please include this amended Role of Funder statement in your cover letter; we will change the online submission form on your behalf.

Response: We have stated the following: **"The funders had no role in study design, data collection and analysis, decision to publish, or preparation of the manuscript."**

2. We note that your Data Availability Statement is currently as follows: [All relevant data are within the manuscript and its Supporting Information files.]

Please confirm at this time whether or not your submission contains all raw data required to replicate the results of your study. Authors must share the “minimal data set” for their submission. PLOS defines the minimal data set to consist of the data required to replicate all study findings reported in the article, as well as related metadata and methods (<https://journals.plos.org/plosone/s/data-availability#loc-minimal-data-set-definition>).

**Response: This is a review paper. Datasets do not apply. However, along with reasoning, we have included the names and DOI of all the articles accessed (included and excluded) in the review. Please see the supplement files (S4).**


3. As required by our policy on Data Availability, please ensure your manuscript or supplementary information includes the following:

1.A numbered table of all studies identified in the literature search, including those that were excluded from the analyses. For every excluded study, the table should list the reason(s) for exclusion. If any of the included studies are unpublished, include a link (URL) to the primary source or detailed information about how the content can be accessed.

**Response: We have the tables for the included articles in the manuscript. All excluded articles with reasoning and DOI are provided in S4. We do not and never list excluded studies in the main manuscript. You can read any review paper published in the history of science to verify this.**

2. A table of all data extracted from the primary research sources for the systematic review and/or meta-analysis. The table must include the following information for each study:

-Name of data extractors and date of data extraction --Confirmation that the study was eligible to be included in the review.

-All data extracted from each study for the reported systematic review and/or meta-analysis that would be needed to replicate your analyses. If data or supporting information were obtained from another source (e.g. correspondence with the author of the original research article), please provide the source of data and dates on which the data/information were obtained by your research group.

**Response: This does not apply to any systematic review paper. The authors did all the work. This is not a meta-analysis. Please see some published review papers in PLOS or any other journals and show me which paper has the names of data extractors in a table in the main manuscript.**

4. If applicable for your analysis, a table showing the completed risk of bias and quality/certainty assessments for each study or outcome. Please ensure this is provided for each domain or parameter assessed. For example, if you used the Cochrane risk-of-bias tool for randomized trials, provide answers to each of the signalling questions for each study. If you used GRADE to assess certainty of evidence, provide judgements about each of the quality of evidence factor. This should be provided for each outcome.

**Response: N/A**

5. An explanation of how missing data were handled.

**Response: Missing value in a review paper? N/A.**

**I request that PLOS avoid such irrelevant requests for a satisfactory checklist for the internal team. Please respect our time.**

---------------------------------------------------------------------------------------------------------------------

**Response to Editor**

Dear editor. Thank you for giving us the chance to improve the manuscript. Given the delay, we decided to redo the entire review and update our results. We have addressed all the comments and more. We were asked to include a list of all papers screened, with their DOI/Link and reason for exclusion (which I have never seen in my life before). All this information has been provided in S4 – however, I am unsure what value it will add to our work.

**Response to Reviewers**

**Reviewers**

Reviewer #1: This paper provides information about the effectiveness of evidence-based health apps on user mental health outcomes. The article provides information with high clinical relevance in the field of mental health interventions. However, some aspects must be improved to facilitate understanding. In this sense, I would like to add some comments/suggestions to this paper:

• It is recommended to include the protocol registration number (line 103).

Response: We added the OSF protocol citation.

“The detailed protocol (osf.io/x6m7u) is registered at the Open Science Framework [39].”

• Why have authors limit the objective of the study only to these mental health problems (depression, anxiety, bipolar disorder, psychosis, and suicidal behaviors)?

Response: We agree. However, reviews on other mental health disorders already exist, and for some disorders, there are no apps for us to evaluate. We have clarified this in the manuscript.

“Few existing reviews in this field have focused on the feasibility of various apps designed for agoraphobia, eating disorders, post-traumatic stress disorders (PTSD), substance use disorders, and sleep disorders [34, 35]. However, there is lack of evidence reviewing effectiveness of mobile apps in improving mental health outcomes [36, 37].”

• Some search terms (depression, anxiety, suicide, mobile, app, and smartphone) do not match the aim of the study (depression, anxiety, bipolar disorder, psychosis, and suicidal behaviors). Bipolar disorder and psychosis are not included as search terms.

Response: Thank you for pointing this out. We started with those keywords, but there are studies involving an app that matches our inclusion criteria. We then revised our search term in the manuscript to avoid further discrepancies. Please see S2 for the detailed search query.

• It is recommended to provide more information about the methodology for selecting articles (e.g., if it was done by title and abstract; if, in the event of not reaching an agreement, they included third parties in the decision; what specific software was used to remove duplicates and for screening abstracts).

Response: We have now updated this section to specify that Excel sheets were used for duplicate removal, decisions on conflicts were resolved through discussion between the two authors without the involvement of third parties and how titles and abstracts were screened.

• Were all the apps based on the cognitive behavioral approach? Did any applications include functions from other approaches? To better understand the results, it is recommended to include a table specifying the components of the different apps (e.g., mindfulness, physical activity tracking) for each CBT function.

Response: All the apps included in this study were based on the cognitive behavioral approach, as their core components (e.g., cognitive change, attention change, and context engagement) align with CBT principles. While some functions, such as distress tolerance (BlueIce app) and self-soothing (iBobbly app), share similarities with techniques used in Dialectical Behavior Therapy (DBT), these were integrated within a CBT framework. Additionally, general wellness strategies, such as physical activity tracking and gamified challenges, complement CBT techniques by enhancing behavioral engagement. To address this comment, we have added an extra column to Table III that specifies the components of each app (e.g., mindfulness, physical activity tracking) and their alignment with CBT functions, ensuring comprehensive understanding.

• It is recommended to specify the number of articles deleted by title and the number of articles deleted by abstract separately.

Response: Thank you for your suggestion. We have included the specific numbers of articles deleted at each stage (by title and by abstract) in the manuscript. Also see S4.

• Was any quality assessment carried out on the articles used? For example, through the Effective Public Health Practice Project Quality Assessment Tool (EPHPP)

Response: Yes, we conducted a quality assessment of the included articles using the Mixed Methods Appraisal Tool (MMAT) instead of EPHPP.

Quality Assessment and Risk of Bias:

*“We conducted a quality assessment of the papers following the Mixed Methods Appraisal Tool (MMAT) (see S3) [42]. The MMAT assesses the quality of qualitative, quantitative, and mixed methods studies. It focuses on methodological criteria and includes the nature of the study (randomized or nonrandomized clinical trial) across the performance bias, detection bias, attrition bias, and selection bias. No articles were excluded”.*

• In Table 1 it is recommended to indicate the standard deviation of the age of the participants.

Response: We reviewed and updated Table 1 to include the mean and standard deviation (SD) of participant ages for studies where these values were explicitly reported in the original articles. For other studies that only provided age ranges, we were unable to calculate or include the SD due to the lack of detailed data.

• It is recommended to review and unify the uppercase and lowercase letters used in the instrument’s column of table 1

Response: Thank you for the suggestion. We have reviewed and updated the "Survey Instrument" column in Table 1 to ensure consistency in the use of uppercase and lowercase letters. All survey names now follow a standardized Title Case format with properly formatted acronyms in parentheses.

• On lines 176-177 it is recommended to indicate the number of studies.

Response: We have addressed this by including the number of studies corresponding to each approach in the manuscript.

• The improvement in mental health outcomes is mentioned in the discussion section. However, few applications address suicidal behaviors. It is recommended that this section be redone, specifying mental health information obtained in more detail.

Response: Thank you for your valuable feedback. We have revised the discussion section and provided more detailed information, particularly regarding suicidal behaviors.

Discussion:

*“Our review also highlights a gap in the efficacy of mHealth apps for managing more complex mental health conditions such as suicidal behaviors, where evidence remains scant and less definitive. Only a few apps specifically target suicidal ideation or behaviors. For example, among the apps reviewed, BlueIce and TEC app include features designed to help users manage self-harming and suicidal thoughts. These apps employ distress tolerance techniques, mood tracking, and crisis management features to address suicidal behaviors, but the evidence supporting their efficacy remains preliminary. This mirror concerns in the broader literature about the challenges of addressing high-risk mental health conditions through app-based interventions alone.*

• Table 3 does not indicate whether the results obtained were short or long term.

Response: We have added a new column, "Study duration," to Table 3 to specify the duration of each study (e.g., 3 weeks, 6 weeks).
